# Supplementary material for: Global epidemiology of hepatitis C virus in dialysis patients: A systematic review and meta-analysis
Source: PLoS One. 2024 Feb 8;19(2):e0284169. doi: 10.1371/journal.pone.0284169 (PMC10852299; doi:10.1371/journal.pone.0284169)
Supplement: S2 Table — (PDF) [file pone.0284169.s003.pdf]

S2 Table: Search strategy in databases

		Search	Items
Medline	1	exp Hepatitis C/	69665
	2	exp Hepatitis C, Chronic/	26992
	3	exp Hepacivirus/	36879
	4	Hepatitis C.mp.	89776
	5	Hepacivirus.mp.	36945
	6	HCV.mp.	56609
	7	exp Renal Dialysis/	123807
	8	exp Renal Insufficiency, Chronic/	130587
	9	exp Kidney Failure, Chronic/	99710
	10	exp Hemodialysis Units, Hospital/	1476
	11	hemodialysis.mp.	65715
	12	haemodialysis.mp.	15102
	13	1 or 2 or 3 or 4 or 5 or 6	94743
	14	7 or 8 or 9 or 10 or 11 or 12	212005
	15	13 and 14	3303
Embase	1	exp Hepatitis C/	128905
	2	exp Hepatitis C virus/	72174
	3	exp chronic hepatitis C/	13871
	4	exp Hepacivirus/	74266
	5	Hepatitis C.mp.	171611
	6	Hepatitis C virus.mp.	100358
	7	Hepacivirus.mp.	2435
	8	HCV.mp.	108215
	9	chronic hepatitis C.mp.	38851
	10	exp hemodialysis/	123698
	11	exp Chronic kidney failure/	128416
	12	hemodialysis.mp.	165816
	13	haemodialysis.mp.	22608
	14	Chronic kidney failure.mp.	125698
	15	1 or 2 or 3 or 4 or 5 or 6 or 7 or 8 or 9	189328
	16	10 or 11 or 12 or 13 or 14	272543
	17	15 and 16	6501
Web of Science		(Hepatitis C OR Hepacivirus OR chronic hepatitis C OR HCV) AND (Renal Dialysis OR Hemodialysis OR Haemodialysis OR Chronic kidney failure)	4294
Global Index Medicus		(Hepatitis C OR Hepacivirus OR chronic hepatitis C OR HCV) AND (Renal Dialysis OR Hemodialysis OR Haemodialysis OR Chronic kidney failure)	403
