# Supplementary material for: Global epidemiology of hepatitis C virus in dialysis patients: A systematic review and meta-analysis
Source: PLoS One. 2024 Feb 8;19(2):e0284169. doi: 10.1371/journal.pone.0284169 (PMC10852299; doi:10.1371/journal.pone.0284169)
Supplement: S3 Table — (PDF) [file pone.0284169.s004.pdf]

S3 Table: *Items for risk of bias assessment*

**Hoy et al. tool for cross sectional studies**	**Yes (1)/No (0)**
**External validity**	
1. Was the study's target population a close representation of the national population in relation to HCV prevalence?	**1**
2. Was the sampling frame a true or close representation of the population?	**1**
3. Was some form of random selection used to select the sample, OR was a census undertaken?	**1**
4. Were data collected directly from the subjects (as opposed to a proxy)?	**1**
**Internal validity**	
5. Was an acceptable inclusion criteria definition used in the study?	**1**
6. Did the author calculate and respect the expected sample size?	**1**
7. Was the HCV detection assay shown to have reliability and validity?	**1**
8. Was the same mode type of sample collected for all subjects?	**1**
9. Was the length of the length of the study period > 1 year?	**1**
10. Were the numerator(s) and denominator(s) for the HCV prevalence?	**1**
Total score	**10**
**Interpretation of the risk of bias tool**<br><br>• 7-10: Low risk of bias<br>• 4-6: Moderate risk of bias<br>• 0-3: High risk of bias	

Modified from: Hoy D, Brooks P, Woolf A, Blyth F, March L, Bain C, et al. Assessing risk of bias in prevalence studies: modification of an existing tool and evidence of interrater agreement. J Clin Epidemiol. 2012;65: 934–939. doi:10.1016/j.jclinepi.2011.11.014
