# Supplementary material for: Global epidemiology of hepatitis C virus in dialysis patients: A systematic review and meta-analysis
Source: PLoS One. 2024 Feb 8;19(2):e0284169. doi: 10.1371/journal.pone.0284169 (PMC10852299; doi:10.1371/journal.pone.0284169)
Supplement: S4 Table — (PDF) [file pone.0284169.s005.pdf]

S4 Table: Main reasons of exclusion of eligible studies

N°	Author, Year (Year of publication)	Title	Reason of exclusion
1	Abdalla, 1998	Is the hepatitis C seroconversion rate higher with dialysis catheters than A-V fistula.	No data on HCV prevalence in HD patients
2	Abdalla, 2000	Influence of hepatitis C virus infection upon parenteral iron and erythropoietin responsiveness in regular haemodialysis patients.	No data on HCV prevalence in HD patients
3	Abdel Mohsen, 2013	Fibromyalgia in egyptan patients on haemodialysis. Does hepatitis C viral infection has a role?	No data on HCV prevalence in HD patients
4	Abdel-Maksoud, 2019	Hepatitis B variants among Egyptian patients undergoing hemodialysis. Microbiology and Immunology	Selection of study participants with already HCV result known
5	Abdelsalam, 2019	Efficacy of nutritional support program on anthropometric measurement and subjective global assessment score among hemodialysis patients.	No data on HCV prevalence in HD patients
6	Abene, 2017	Practice of Hemodialysis in a Resource-Poor Setting in Nigeria: A 2-Year Experience.	No data on HCV prevalence in HD patients
7	Abou Rached, 2017	Prevalence of hepatitis C virus genotypes and subtypes in Lebanese population and major high risk groups.	No data on HCV prevalence in HD patients
8	Abutaleb, 2012	Eliminating the chronic problem of false positive HCV testing from hemodialysis units at lowest cost.	No data on HCV prevalence in HD patients
9	Acchiardo, 1991	Is hematologic response to iron and erythropoietin in hemodialysis patients affected by other factors?	No data on HCV prevalence in HD patients
10	Agarwal, 2009	Prevalence and association of hepatitis C viremia in hemodialysis patients at a tertiary care hospital.	No data on HCV prevalence in HD patients
11	Aghakhani, 2012	Viral Hepatitis and HIV Infection in Hemodialysis Patients.	Comment on an article
12	Ahmed Ali, 2009	HBsAg and anti-HBC antibodies among hemodialysis patients Al-Mukalla Hadhramout, Yemen.	No data on HCV prevalence in HD patients
13	Akhan, 2015	An outbreak of acute HCV infection with genotype 1a in a Haemodialysis Unit in Kocaeli, Turkey.	Selection of study participants with already HCV result known
14	Alam, 2018	Incidence of hepatitis-b and hepatitis-c virus in whole blood transfused hemodialysis dependent ckd patient in a tertiary level hospital.	No data on HCV prevalence in HD patients
15	Alavian, 2009	A shield against a monster: Hepatitis C in hemodialysis patients.	Comment on an article
16	Alavian, 2013	Seronegative anti-hepatitis C virus antibody in hemodialysis patients.	No data on HCV prevalence in HD patients
17	Alavian, 2008	Hepatitis B and C in dialysis units in Iran: changing the epidemiology.	Not possible to extract data on HCV prevalence
18	Alavian, 2003	Study of prevalence and risk factors of hepatitis C in hemodialysis patients	Full text or abstract not found
19	Alavian, 2004	Prevalence and risk factors of hepatitis C in dialysis patients in Qazvin (2001).	Full text or abstract not found

20	al-Dhahry, 1992	Antibodies to hepatitis C virus in Omani patients with renal disease.	Full text or abstract not found
21	Alivanis, 1991	Hepatitis C virus antibodies in hemodialysed and in renal transplant patients: correlation with chronic liver disease.	Full text or abstract not found
22	Almroth, 1991	Antibody responses to hepatitis C virus and its modes of transmission in dialysis patients.	Sample size < or = 10 participants
23	Alvarado Esquivel, 2001	[Transmission of hepatitis C virus by dialysis: myth or reality? New perspective with molecular studies].	Article not in English or in French
24	Anees, 2008	Depression in hemodialysis patients.	No data on HCV prevalence in HD patients
25	Anees, 2021	Factors Associated with Seroconversion of Hepatitis C Virus in End Stage Renal Disease Patients.	No data on HCV prevalence in HD patients
26	Angelet, 1998	Absence of hepatitis-C virus seroconversion in hemodialysis.	Selection of study participants with already HCV result known
27	Ansaldi, 2003	An outbreak of hepatitis C virus in a haemodialysis unit: molecular evidence of patient-to-patient transmission.	Selection of study participants with already HCV result known
28	Arduino, 2008	Infections in dialysis patients.	Review
29	Arenas, 2001	Nosocomial transmission of hepatitis C virus: Dialysis, machines, staff or both?	Article not in English or in French
30	Arguillas, 1991	Seroepidemiology of hepatitis C virus infection in the Philippines: a preliminary study and comparison with hepatitis B virus infection among blood donors, medical personnel, and patient groups in Davao, Philippines.	Sample size < or = 10 participants
31	Arora , 2016	Hepatitis C Virus Infection in Patients with End-Stage Renal Disease: A Study from a Tertiary Care Centre in India.	Selection of study participants with already HCV result known
32	Asif, 2020	Frequency Of Hepatitis-B And C In Patients On Haemodialysis For End Stage Renal Disease In Tertiary Care Hospitals: A Multicentre Study.	Not possible to extract data on HCV prevalence
33	Asif, 2019	Frequency of Hepatotropic Viruses Leading To Deranged Liver Function Tests in Renal Transplant Recipients.	No data on HCV prevalence in HD patients
34	Aucella, 1995	[Epidemiology of hepatitis C virus infection in hemodialysis. A study of the Gargano area].	Article not in English or in French
35	Avella, 1992	On the positivity of the test for anti-HCV considerations in dialized patients.	Full text or abstract not found
36	Aydogan, 2012	To study the correlation between carrier status of nasal Staphylococcus aureus in patients on haemodialysis with hepatitis C, hepatitis B and their sociodemographic features.	Not possible to extract data on HCV prevalence
37	Azadegan-Ghomi H, 2007	Prevalence of Hepatitis B, C, HIV in Patients Under Dialysis in Ghom 1383. Tehran: The 3rd Iranian Congress of Virology; 2007.	Full text or abstract not found
38	Azhar, 2011	A prospective study of hepatitis c virus infection in hemodialysis patients in Jeddah, Saudi Arabia.	No baseline data for longitudinal study
39	Babaei, 2004	Comparison of prevalence of hepatitis C virus (HCV) infection among injection drug users (Idus) with other high risk groups: A case– control study of blood donors.	Full text or abstract not found
40	Bahçebaşi, 2004	[Hepatitis C virus seroprevalence in the hemodialysis unit of our hospital and prevention of transmission].	Article not in English or in French

41	Bansky, 1984	[Non-A, non-B hepatitis in patients with chronic hemodialysis].	Case report
42	Barril, 1998	Liver and kidney - Transmission of hepatitis C virus in dialysis units.	Article not in English or in French
43	Barril, 2000	Hepatitis C virus-induced liver disease in dialysis patients.	Review
44	Barril, 2018	Effect of hemodialysis schedules and membranes on hepatocyte growth factor and hepatitis C virus RNA levels.	Selection of study participants with already HCV result known
45	Barril, 2003	Decrease in the hepatitis C virus (HCV) prevalence in hemodialysis patients in Spain: effect of time, initiating HCV prevalence studies and adoption of isolation measures.	No data on HCV prevalence in HD patients
46	Barton, 1998	The seroprevalence of hepatitis and retroviral infection in Jamaican haemodialysis patients.	No baseline data for longitudinal study
47	Bastos, 2012	Transcription-mediated amplification (TMA) for the assessment of viremia in hemodialysis patients with hepatitis C.	Selection of study participants with already HCV result known
48	Bayu, 2020	Assessment of liver fibrosis using APRI score after treatment with elbasvir/grazoprevir in patients with hepatitis C infection and chronic kidney disease on hemodialysis.	Selection of study participants with already HCV result known
49	Beccari, 1996	Anti-HCV prevalence, viraemic status and genotype distribution in an Italian haemodialysis population.	No data on HCV prevalence in HD patients
50	Becker, 2022	Performance of rapid test for antibodies to hepatitis C virus (anti-HCV) in hemodialysis patients and kidney transplant recipients.	Selection of study participants with already HCV result known
51	Bellissima, 1996	[Prevalence of hepatitis C virus infection in different groups of selected populations].	Article not in English or in French
52	Bessa, 2009	Limited evidence of hcv transmission in stable heterosexual couples from Bahia, Brazil.	Selection of study participants with already HCV result known
53	Bhattacharya, 2009	Holiday haemodialysis and imported hepatitis C virus infection: a series of sixteen cases in two large haemodialysis units.	Case report
54	Biamino, 1999	[Prevalence of anti-HCV antibody positivity and seroconversion incidence in hemodialysis patients].	Article not in English or in French
55	Bilgin, 1993	Prevalence of anti-HCV positivity in hemodialysis and renal transplant patients at our center.	Full text or abstract not found
56	Bocşan, 1995	[The markers of hepatitis B, C and D viral infection in multiply transfused patients].	Article not in English or in French
57	Borawski, 2006	[SEN virus infection in maintenance hemodialysis patients].	Article not in English or in French
58	Bosmans, 1997	Prevalence and clinical expression of HCV-genotypes in haemodialysis-patients of two geographically remote countries: Belgium and Saudi-Arabia.	Selection of study participants with already HCV result known
59	Botelho, 2008	Epidemiological aspects of hepatitis C virus infection among renal transplant recipients in Central Brazil.	No data on HCV prevalence in HD patients
60	Bouchardeau, 1993	[Correlation between hepatitis C virus (HVC) RNA and anti-HVC antibodies in a hemodialysis population].	Selection of study participants with

			already HCV result known
61	Bouzgarrou, 2005	Evaluation of a total core antigen assay for the diagnosis of hepatitis C virus infection in hemodialysis patients.	No baseline data for longitudinal study
62	Bozdayi, 2002	[The presence of hepatitis C virus (HCV) infection in hemodialysis patients and determination of HCV genotype distribution].	Article not in English or in French
63	Bozorgi, 2008	Assessment of prevalence and risk factors of hepatitis C virus infection in haemodialysis patients in Ghazvin. SJIBTO (Blood J).	Article not in English or in French
64	Bracho, 2005	Molecular epidemiology of a hepatitis C virus outbreak in a hemodialysis unit.	Selection of study participants with already HCV result known
65	Brncić, 1996	Hepatitis C virus infection in patients on hemodialysis and after renal transplantation in the area of Rijeka-Croatia.	Full text or abstract not found
66	Bruch Igartúa, 1999	[Markers of hepatitis C virus infection in dialysis units. A decreasing problem but still present in Argentina].	Article not in English or in French
67	Bui, 2013	Study of prevalence and clinical features of HBV/HCV carrier on hemodialysis and after kidney transplantation. T	Article not in English or in French
68	Bui, 1999	HCV infection among haemodialysis patients and some subjects who have relationship with blood transfusion in Vietnam.	Article not in English or in French
69	Bukhari, 2020	Predictors of hcv seroconversion among end-stage renal disease patients in hemodialysis unit.	No baseline data for longitudinal study
70	Butsashvili, 2022	Hepatitis B and hepatitis C testing practices and seroconversions among dialysis facilities in Georgia.	No data on HCV prevalence in HD patients
71	Cailliau, 1994	Transmission of hepatitis C virus (HCV) in a haemodialysis unit.	No data on HCV prevalence in HD patients
72	Camps, 1992	[The prevalence of anti-hepatitis virus C antibodies in chronic hemodialysis patients].	Article not in English or in French
73	Cañero-Velasco, 1998	[HCV and HBV prevalence in hemodialyzed pediatric patients. Multicenter study].	Article not in English or in French
74	Cao, 2006	Hepatitis C virus infection in maintenance hemodialysis patients.	Full text or abstract not found
75	Capra, 1995	[Anti-HCV antibodies in chronically dialyzed uremic patients. 1-year follow-up study].	Article not in English or in French
76	Caramelo, 1999	Hand-borne mechanisms of dissemination of hepatitis C virus in dialysis units: basis for new addenda to the present preventive strategies.	Full text or abstract not found
77	Caramelo, 1994	Evidence against transmission of hepatitis C virus through hemodialysis ultrafiltrate and peritoneal fluid.	Selection of study participants with already HCV result known
78	Carneiro, 2005	Decline of hepatitis C infection in hemodialysis patients in Central Brazil: a ten years of surveillance.	No baseline data for longitudinal study
79	Castillo, 1993	[Prevalence of hepatitis C virus antibodies in chronic hemodialysis and kidney transplantation patients].	Article not in English or in French
80	Cendoroglo Neto, 1995	Incidence of and risk factors for hepatitis B virus and hepatitis C virus infection among haemodialysis and CAPD patients: evidence for environmental transmission.	No baseline data for longitudinal study

81	Chan, 1993	Prevalence of hepatitis C virus infection in hemodialysis patients: a longitudinal study comparing the results of RNA and antibody assays.	No baseline data for longitudinal study
82	Chandra, 2004	Prevalence of hepatitis B and hepatitis C viral infections in Indian patients with chronic renal failure.	No data on HCV prevalence in HD patients
83	Chauveau, 1993	Antibodies to hepatitis C virus by second generation test in hemodialyzed patients.	No baseline data for longitudinal study
84	Chebrolu, 2015	Bacteraemia in haemodialysis patients with hepatitis C.	No data on HCV target detected
85	Chen, 2002	Hepatitis C virus infections and genotypes in China.	Not possible to extract data on HCV prevalence
86	Chen, 2013	The risk factors of hepatitis C virus infection in maintenance hemodialysis patients.	Full text or abstract not found
87	Chen, 2002	Hepatitis B and C virus infection in uremia patients on chronic hemodialysis	Full text or abstract not found
88	Chen, 2015	Analysis of related factors of hemodialysis hepatitis c virus infection in patients with type 2 diabetic renal failure.	Full text or abstract not found
89	Cherchiglia, 2016	[Hepatitis C incidence in hemodialysis patients in Brazil from 2000 to 2003].	Article not in English or in French
90	Choi, 2003	[Increased skin pigmentation in patients with chronic renal failure undergoing hemodialysis infected with the hepatitis C virus].	Article not in English or in French
91	Chu, 1994	[Study on serum anti-HCV antibodies in hemodialysis patients].	Article not in English or in French
92	Chuang, 2005	Anticardiolipin antibody and Taiwanese chronic haemodialysis patients with recurrent vascular access thrombosis.	No data on HCV prevalence in HD patients
93	Chuang, 2005	IgM-anticardiolipin antibody and vascular access thrombosis in chronic hemodialysis patients.	No data on HCV prevalence in HD patients
94	Colichon Yerosh, 2004	[Serologic prevalence of HCV antibodies in health personnel in Peru].	Article not in English or in French
95	Colleoni, 1996	Hepatitis C virus genotype in anti-HCV-positive hemodialysed patients.	No baseline data for longitudinal study
96	Colombo, 1997	[Hepatitis C virus and mixed cryoglobulinemia in dialysis].	Article not in English or in French
97	Comty, 1978	Hepatitis in hemodialysis units.	No data on HCV prevalence in HD patients
98	Courault, 1991	[HCV antibodies in selected patient groups and blood donors].	Article not in English or in French
99	Courouce, 1991	Hepatitis C virus infection in a Paris haemodialysis unit [3].	Full text or abstract not found
100	Couroucé, 2000	Efficacy of HCV core antigen detection during the preseroconversion period.	No baseline data for longitudinal study
101	Couroucé, 1994	Anti-hepatitis C virus (anti-HCV) seroconversion in patients undergoing hemodialysis: comparison of second- and third-generation anti-HCV assays.	No baseline data for longitudinal study
102	Crnjaković-Palmović, 2005	[Hepatitis virus infection among hemodialysis patients].	No data on HCV prevalence in HD patients

103	Curciarello, 1996	[Hepatitis C viruses antibodies. Prevalence and their influence on morbidity-mortality in renal transplant recipients, in the last two years of the kidney transplantation program in La Plata city].	No data on HCV prevalence in HD patients
104	Curciarello, 1994	[Viral hepatitis infection and response to the hepatitis B vaccine in hemodialyzed patients].	No data on HCV prevalence in HD patients
105	D' Marco, 2021	SARS-CoV-2 vs. Hepatitis Virus Infection Risk in the Hemodialysis Population: What Should We Expect?	No data on HCV prevalence in HD patients
106	da costa M, 2019	Hepatitis C virus genotypes in hemodialysis patients in Angola.	Selection of study participants with already HCV result known
107	da Costa Marques Borges, 2019	Hepatitis C virus genotypes in hemodialysis patients in Angola. Journal of medical virology	Selection of study participants with already HCV result known
108	Da Porto, 1992	Comparison between first- and second-generation test for anti-hepatitis C virus antibodies in hemodialysis patients.	Duplicates
109	Dadmanesh, 2015	Evaluation of prevalence and risk factors of hepatitis g virus infection among hemodialysis patients referred to Iranian army hospitals in tehran during 2012-2013.	No data on HCV prevalence in HD patients
110	Dadmanesh, 2015	Evaluation of prevalence and risk factors of hepatitis G virus infection among hemodialysis patients referred to Iranian Army Hospitals in Tehran during 2012-2013.	No data on HCV prevalence in HD patients
111	Davis, 1994	Hepatitis C virus in renal disease.	No data on HCV prevalence in HD patients
112	Di Benedetto, 1992	Anti-HCV antibody presence in hemodialyzed patients: assessment after one year regarding some preventive measures chosen.	No data on HCV prevalence in HD patients
113	Di Napoli , 2006	Epidemiology of hepatitis C virus among longterm dialysis patients: a 9-year study in an Italian region. Am	Not possible to extract data on HCV prevalence
114	Diouf, 1999	[Hepatitis C virus genotypes among chronic hemodialysis patients in Dakar].	Full text or abstract not found
115	dos Santos, 1996	Impact of dialysis room and reuse strategies on the incidence of hepatitis C virus infection in haemodialysis units.	No baseline data for longitudinal study
116	Dotta, 2003	Molecular and serological assays in the diagnosis of Hepatitis C in hemodialysis patients.	Full text or abstract not found
117	Duclos, 1995	[Hepatitis C virus viremia and Herpes zoster virus infection in a patient in hemodialysis treated with erythropoietin].	Article not in English or in French
118	Dunford, 2012	A multicentre molecular analysis of hepatitis B and blood-borne virus coinfections in Viet Nam	No data on HCV prevalence in HD patients
119	Duong, 2016	An investigation of an outbreak of hepatitis C virus infections in a low-resourced hemodialysis unit in Vietnam.	No baseline data for longitudinal study
120	Duong, 2016	Shewhart charts and twomonthly screening interval to monitor hepatitis C and hepatitis B virus infections in two-year prospective cohort study of hemodialysis patients in Vietnam.	No baseline data for longitudinal study
121	Dussol, 1995	Hepatitis C virus infection among chronic dialysis patients in the south of France: a collaborative study.	Duplicates
122	Dussol, 1996	Is hepatitis C virus-RNA detection by nested polymerase chain reaction clinically relevant in hemodialysis patients?	Selection of study participants with already HCV result known

123	Dzekova-Vidimliski, 2012	Decreasing prevalence of hepatitis C virus infection in hemodialysis patients:following kdigo guidalines.	Not possible to extract data on HCV prevalence
124	Dzekova-Vidimliski, 2017	Hepatitis C virus infection in maintenance hemodialysis patients: recommendations for diagnostics and treatment.	Review
125	Elsawy, 2005	Serotyping of hepatitis C virus in hemodialysis patients: comparison with a standardized genotyping assay.	Selection of study participants with already HCV result known
126	Engel, 2007	Acute hepatitis C virus infection assessment among chronic hemodialysis patients in the Southwest Parana State, Brazil.	No data on HCV prevalence in HD patients
127	Ergani, 2010	Investigation of hepatitis B virus and hepatitis C virus infections by using serological and molecular methods among the hemodialysis patients.	Article not in English or in French
128	Eslamifar, 2007	Hepatitis G virus exposure in dialysis patients.	No data on HCV prevalence in HD patients
129	Espinosa, 2000	Hepatitis C virus in hemodialysis patients.	Comment on an article
130	Espinosa, 2000	High ALT levels predict viremia in anti-HCV-positive HD patients if a modified normal range of ALT is applied.	No data on HCV prevalence in HD patients
131	Espinosa, 2002	Natural history of acute HCV infection in haemodialysis patients	No baseline data for longitudinal study
132	Fabrizi, 2021	Updated Evidence on the Epidemiology of Hepatitis C Virus in Hemodialysis.	Review
133	Fabrizi, 1995	HEPATITIS-C VIRUS-INFECTION IN DIALYSIS AND CLINICAL NEPHROLOGY.	Review
134	Fabrizi, 1999	Hepatitis C virus in the haemodialysis units: novel insights by new techniques?	Review
135	Fabrizi, 2007	Hepatitis C virus infection and the dialysis patient.	Review
136	Fabrizi, 1996	Hepatitis C virus genotypes in chronic dialysis patients.	Selection of study participants with already HCV result known
137	Fabrizi, 1996	IgM antibody response to hepatitis C virus in end-stage renal disease.	Selection of study participants with already HCV result known
138	Fabrizi, 2022	Hepatitis B virus infection in hemodialysis: Recent discoveries.	Review
139	Fabrizi, 2002	Recent advances in the management of hepatitis C in the dialysis population.	Review
140	Fabrizi, 2002	Genetic variability of hepatitis C virus in dialysis: the implications.	Review
141	Fabrizi, 2003	Peritoneal dialysis and infection by hepatitis B and C virus.	Review
142	Fabrizi, 2010	Health care-associated transmission of hepatitis B and C viruses in hemodialysis units.	Review
143	Fabrizi, 2004	[HCV-related liver disease in hemodialysis population: clinical and biochemical characteristic].	Case report
144	Fabrizi, 2000	Membrane compatibility, flux and HCV infection in dialysis patients: newer evidence.	Review
145	Fabrizi, 2000	Nosocomial transmission of hepatitis C virus infection in hemodialysis patients: clinical perspectives.	Review
146	Fabrizi, 2013	[Control of HCV, HBV and HIV Infections in Hemodialysis].	Review
147	Fabrizi, 2000	Serotyping strip immunoblot assay for assessing hepatitis C virus strains in dialysis patients.	Selection of study participants with already HCV result known

148	Fekrey, 2011	Incidence of acquiring hepatitis B and C virus infection in hemodialysis patients.	No baseline data for longitudinal study
149	Fengyi, 2003	A comprehensive analysis of 135 hemodialysis patients with viral hepatitis b and c infection.	Full text or abstract not found
150	Ferreira Ade, 2011	Acute hepatitis C in Brazil: results of a national survey.	Selection of study participants with already HCV result known
151	Florentino, 2004	Prevalence of hepatitis c virus in patients undergoing hemodialysis in Campina Grande (Paraíba, Brazil).	Full text or abstract not found
152	Fontaine, 2002	[Hepatitis C in certain patient populations: children, patients with hemophilia and thalassemia, the hemodialyzed and kidney transplant recipients].	Full text or abstract not found
153	Fontenele, 2015	Occult hepatitis b among patients with chronic renal failure on hemodialysis from a capital city in northeast Brazil.	Review
154	Frider, 1994	Prevalence of hepatitis C in health care workers investigated by 2nd generation enzyme-linked and line immunoassays.	No data on HCV prevalence in HD patients
155	Froio, 2003	Contamination by hepatitis B and C viruses in the dialysis setting.	No data on HCV prevalence in HD patients
156	Fujiyama, 2000	[Hepatitis C: epidemiology and therapy--with special reference to long-term prognosis after IFN therapy].	No data on HCV prevalence in HD patients
157	Furusyo, 2000	Maintenance hemodialysis decreases serum hepatitis C virus (HCV) RNA levels in hemodialysis patients with chronic HCV infection.	Selection of study participants with already HCV result known
158	Furusyo, 2004	Confirmation of nosocomial hepatitis C virus infection in a hemodialysis unit.	Selection of study participants with already HCV result known
159	Furuta, 1992	[Viral hepatitis in patients on chronic hemodialysis and staff members in hemodialysis centers].	Article not in English or in French
160	Galán, 1992	[Hepatitis C in hemodialyzed patients and with renal transplantation].	Article not in English or in French
161	Galán, 1998	A 3-year follow-up of HCV-RNA viraemia in haemodialysis patients.	Selection of study participants with already HCV result known
162	Gan, 2022	Hepatitis C Prevalence, Incidence, and Treatment in Chinese Hemodialysis Patients: Results From the Dialysis Outcomes and Practice Patterns Study-China (2019-21).	No data on HCV target detected
163	Garassini, 1991	[Antibodies against hepatitis C virus. Experience with a second generation test].	Selection of study participants with already HCV result known
164	Garassini, 1995	[Hepatitis caused by virus C. Risk factors].	No data on HCV prevalence in HD patients
165	García Agudo, 2016	Spanish multicentre PIBHE study: Prevalence and immunization of chronic hepatitis B in haemodialysis patients in Spain.	No data on HCV prevalence in HD patients
166	Garcia-Valdecasas, 1994	Hepatitis C virus RNA in patients with anti-HCV on hemodialysis. Relationship to transaminase levels.	Selection of study participants with already HCV result known

167	Gentile, 2013	Acute hepatitis C in patients undergoing hemodialysis: experience with high-dose interferon therapy.	Full text or abstract not found
168	Giachino, 1991	[Anti-HCV in patients undergoing dialysis in Piedmont and in Valle d'Aosta].	No data on HCV prevalence in HD patients
169	Gilli, 1995	Prevention of hepatitis C virus in dialysis units.	Duplicates
170	Gilli, 1992	Hepatitis C virus infection in haemodialysis patients.	Duplicates
171	Gilli, 1990	Anti-HCV positive patients in dialysis units?	Comment on an article
172	Girou, 2008	Determinant roles of environmental contamination and noncompliance with standard precautions in the risk of hepatitis C virus transmission in a hemodialysis unit.	No data on HCV prevalence in HD patients
173	Goessens, 1997	Hepatitis C virus genotypes in hemodialyzed patients: a multicentric study.	No data on HCV prevalence in HD patients
174	Gold, 1979	Hepatitis in the haemodialysis unit, Baragwanath Hospital, 1973-1977. A cross-sectional and longitudinal survey.	No data on HCV prevalence in HD patients
175	Gómez-Gutiérrez, 2015	Prevalence of hepatitis C virus infection among patients undergoing haemodialysis in Latin America.	Review
176	Gonzalez, 1995	Prevalence of hepatitis C virus RNA in hemodialysis patients: comparison of four antibody assays.	Sample size < or = 10 participants
177	González-Candelas, 2003	Molecular epidemiology and forensic genetics: application to a hepatitis C virus transmission event at a hemodialysis unit.	No data on HCV prevalence in HD patients
178	Gonzalez-Michaca, 2000	Hepatitis C in patients with end stage renal disease. I. Prevalence.	No data on HCV prevalence in HD patients
179	Gonzalez-Michaca, 2001	Viral hepatitis C in patients with end-stage renal disease. III. Viral quantification.	No data on HCV prevalence in HD patients
180	González-Michaca, 2000	[Viral C hepatitis in patients with end stage renal disease. II. Viral genotypes].	No data on HCV prevalence in HD patients
181	González-Michaca, 2001	[Viral hepatitis C in patients with terminal chronic renal insufficiency. III. Viral quantification].	No data on HCV prevalence in HD patients
182	Goodkin, 2013	Hepatitis C infection is very rarely treated among hemodialysis patients.	No baseline data for longitudinal study
183	Grethe, 2000	Molecular epidemiology of an outbreak of HCV in a hemodialysis unit: direct sequencing of HCV-HVR1 as an appropriate tool for phylogenetic analysis.	No data on HCV prevalence in HD patients
184	Griveas, 2017	HCV viraemia in anti-HCV-negative haemodialysis patients: A myth?	No data on HCV prevalence in HD patients
185	Griveas, 2007	Acute hepatitis C in patients receiving hemodialysis.	No data on HCV prevalence in HD patients
186	Guaschino, 1994	Should HCV RNA detection affect the isolation policy in dialysis units?	No data on HCV prevalence in HD patients
187	Guiserix, 1996	Contamination by hepatitis C in a haemodialysis center: preventive measures.	No data on HCV prevalence in HD patients
188	Ha, 2013	Hepatitis C infection among maintenance hemodialysis patients at Viet Duc hospital.	Article not in English or in French

189	Hadiwandowo, 1994	Hepatitis B virus subtypes and hepatitis C virus genotypes in patients with chronic liver disease or on maintenance hemodialysis in Indonesia.	No data on HCV prevalence in HD patients
190	Halfon, 1997	[Clinical significance of detection and quantification of hepatitis C virus RNA in hemodialysis: proposal for a rational diagnostic strategy].	No data on HCV prevalence in HD patients
191	Halle, 2018	Incidence and factors associated with seroconversion to hepatitis C virus seropositivity amongst patients on maintenance hemodialysis, Douala-Cameroon.	No data on HCV prevalence in HD patients
192	Hanci, 2008	[Investigation of hepatitis G virus prevalence in hemodialysis patients and blood donors in Denizli, Turkey].	No data on HCV prevalence in HD patients
193	Hanuka, 2004	Hepatitis C virus infection in dialysis and chronic liver patients: Viraemia dependent anti-E2-antibody response.	No data on HCV prevalence in HD patients
194	Hardy, 2000	Hepatitis C virus in the hemodialysis setting: detecting viral RNA from blood port caps by reverse transcription-polymerase chain reaction.	No data on HCV prevalence in HD patients
195	Harris, 1998	Patients surviving more than 10 years on haemodialysis. The natural history of the complications of treatment.	No data on HCV prevalence in HD patients
196	Hassan, 1993	Prevalence of hepatitis C antibodies in patient groups in Egypt.	No data on HCV prevalence in HD patients
197	Hassoba, 2010	Impact of hepatitis C virus infection on neutrophil oxidative burst function in hemodialysis patients.	No data on HCV prevalence in HD patients
198	Hatatian, 2020	Relative Frequency of Blood-Borne Viruses in Hemodialysis-Dependent and Kidney Transplant Recipients in Iran.	Review
199	Hay, 1992	The prevalence of antibody to hepatitis C virus in a dialysis population.	Full text or abstract not found
200	Hayashi, 1998	Hepatitis G virus in the general population and in patients on hemodialysis.	No data on HCV prevalence in HD patients
201	Hayat, 2010	Hepatitis C and kidney disease.	No data on HCV prevalence in HD patients
202	Heikens, 2019	Hepatitis C virus transmission in a Dutch haemodialysis unit: detailed outbreak investigation using NS5A gene sequencing.	No data on HCV prevalence in HD patients
203	Helaly, 2015	Occult hepatitis B virus infection among chronic hemodialysis patients in Alexandria, Egypt.	No data on HCV prevalence in HD patients
204	Hilzenrat, 2000	[Transmission of hepatitis C in hemodialysis patients--does one-time exposure result in chronic hepatitis?].	No data on HCV prevalence in HD patients
205	Hino, 1999	[Detection of TT virus in hemodialysis patients].	No data on HCV prevalence in HD patients
206	Hmaïed, 2007	Determining the source of nosocomial transmission in hemodialysis units in Tunisia by sequencing NS5B and E2 sequences of HCV.	No data on HCV prevalence in HD patients
207	Hoang, 2009	Prevalence and characteristics of HBV and HCV infections among chronic hemodialysis patients with ESRD. T	Article not in English or in French
208	Holzberger, 1991	The prevalence of anti-HCV in hemodialysis patients and blood donors in Germany.	Full text or abstract not found
209	Holzberger, 1992	Second generation anti-HCV test: seroprevalence in hemodialysis patients and blood donors.	Full text or abstract not found

210	Honegr, 1984	[An epidemic of non-A, non-B hepatitis at a hemodialysis center].	No data on HCV prevalence in HD patients
211	Hosokawa, 2000	Phylogenetic evidence, by multiple clone analysis of hypervariable region 1, for the transmission of hepatitis C virus to chronic haemodialysis patients.	No data on HCV prevalence in HD patients
212	Hosseini-Moghaddam, 2006	Distribution of hepatitis C virus genotypes among hemodialysis patients in Tehran--a multicenter study.	No data on HCV prevalence in HD patients
213	Hosseini-Moghaddam, 2008	GB virus type C infection in hemodialysis patients considering co-infection with hepatitis C virus.	No data on HCV prevalence in HD patients
214	Hsu, 2015	Interferon based treatment of hepatitis C virus infection reduces all-cause mortality in patients with end-stage renal disease	No baseline data for longitudinal study
215	Hu, 2005	Clinical presentation of chronic hepatitis C in patients with end-stage renal disease and on hemodialysis versus those with normal renal function.	Selection of study participants with already HCV result known
216	Huang, 1997	Hepatitis in patients with end-stage renal disease.	Case report
217	Huang, 2002	Hepatitis infection in haemodialysis patients.	No data on HCV prevalence in HD patients
218	Huapaya, 2010	[Re: Factors associated with hepatitis C infection in patients with chronic hemodialysis].	Full text or abstract not found
219	Huraib, 2003	Hepatitis C in dialysis patients.	No data on HCV prevalence in HD patients
220	Iotti, 1997	Hepatitis C and chronic hemodialysis. Liver histopathologic analysis.	Selection of study participants with already HCV result known
221	Ismail, 2016	Seroconversion of Patients Undergoing Haemodialysis from HCV Negative to HCV Positive Status.	Full text or abstract not found
222	Izopet, 2001	[HCV nosocomial infections in hemodialysis].	Article not in English or in French
223	Jadoul, 1996	Transmission routes of HCV infection in dialysis.	Review
224	Jadoul, 2000	Epidemiology and mechanisms of transmission of the hepatitis C virus in haemodialysis.	Review
225	Jadoul, 2012	Hepatitis C in hemodialysis: epidemiology and prevention of hepatitis C virus transmission.	Review
226	Jadoul, 2015	Hepatitis C infection in hemodialysis patients is associated with inferior quality of life in the DOPPS.	No data on HCV prevalence in HD patients
227	Jadoul, 2019	Prevalence, incidence, and risk factors for hepatitis C virus infection in hemodialysis patients.	Not possible to extract data on HCV prevalence
228	Jadoul, 2014	Incidence and risk factors for hepatitis C seroconversion in hemodialysis: a prospective study. The UCL Collaborative Group.	No data on HCV prevalence in HD patients
229	Jadoul, 2014	Epidemiology and prevention of hepatitis C virus transmission in the hemodialysis setting.	No data on HCV prevalence in HD patients
230	Jadoul, 2014	What are the management issues for hepatitis C in dialysis patients?: epidemiology and prevention of hepatitis C virus transmission in the hemodialysis setting.	No data on HCV prevalence in HD patients
231	jadoul, 1997	Prevention of hepatitis C virus transmission in hemodialysis units.	No data on HCV prevalence in HD patients

232	Jadoul, 1997	[Prevention of hepatitis C virus transmission in hemodialysis].	No data on HCV prevalence in HD patients
233	Jadoul, 2004	The changing epidemiology of hepatitis C virus (HCV) infection in haemodialysis: European multicentre study.	Not possible to extract data on HCV prevalence
234	Jadoul, 2005	Patient-to-patient transmission of hepatitis C.	No data on HCV prevalence in HD patients
235	Jahromi, 2008	Prevalence of anti HCV antibody hemodialysis patients refering to hemmodialysis unit of Jahrom.	Article not in English or in French
236	Jakupi, 2019	Phylogenetic analysis confirms hepatitis C virus transmission among hemodialysis patients in Kosovo.	No data on HCV prevalence in HD patients
237	Javaid, 2021	Rate of HCV seroconversion and its associated factors in hemodialysis patients.	No data on HCV prevalence in HD patients
238	Johnson, 2009	Frequencies of hepatitis B and C infections among haemodialysis and peritoneal dialysis patients in Asia-Pacific countries: analysis of registry data.	Not possible to extract data on HCV prevalence
239	Jordan, 1992	[Serological results for hepatitis C in hemodialysis patients].	Article not in English or in French
240	Kalantar-Zadeh, 2007	Hepatitis C infection in dialysis patients: a link to poor clinical outcome?	Review
241	Kalantar-Zadeh, 2005	Diagnostic discordance for hepatitis C virus infection in hemodialysis patients.	No data on HCV prevalence in HD patients
242	Kalantar-Zadeh , 2007	Hepatitis C virus and death risk in haemodialysis patients.	Duplicates
243	KAMAL, 2017	Incidence of hepatitis c virus seroconversion among hemodialysis patients in the Nile Delta of Egypt: A single-center study.	No data on HCV prevalence in HD patients
244	Kamal, 2003	[Hepatitis C infection among patients receiving hemodialysis].	Review
245	kamar, 2003	Hepatitis C virus infection in hemodialysis.	Review
246	kamar, 2008	[Prevalence and incidence of hepatitis C virus in hemodialysis: diagnosis and prevention].	Editorial
247	kaneko, 1993	[A study of HCV infection in patients with chronic hemodialysis without blood transfusion].	Article not in English or in French
248	kao, 2015	Utilization of Signal-to-Cutoff Ratio of Hepatitis C Virus Antibody Assay in Predicting HCV Viremia among Hemodialysis Patients.	No data on HCV prevalence in HD patients
249	Karkar, 2007	Hepatitis C in dialysis units: the Saudi experience.	Review
250	Karkar, 2011	Hepatitis C virus transmission through sharing hemodialysis machines.	No data on HCV prevalence in HD patients
251	Kassaian, 2011	Hepatitis C in patients with multi blood transfusion	Article not in English or in French
252	Katsoulidou, 1999	Molecular epidemiology of a hepatitis C virus outbreak in a haemodialysis unit. Multicentre Haemodialysis Cohort Study on Viral Hepatitis.	Sample size < or = 10 participants
253	Khan, 2002	A study of end stage renal disease patients from southern part of Arabian peninsula. (Aetiology of and hepatitis B & C prevalence in patients on maintenance haemodialysis).	Not possible to extract data on HCV prevalence
254	Kim, 2018	Hepatitis C virus infection in chronic kidney disease: paradigm shift in management.	Review

255	Kiyosawa, 1999	Current problems of hepatitis C virus infection in hemodialysis patients.	Editorial
256	Kokubo, 2002	A phylogenetic-tree analysis elucidating nosocomial transmission of hepatitis C virus in a haemodialysis unit.	No data on HCV prevalence in HD patients
257	Kondili, 2006	Nosocomial transmission in simultaneous outbreaks of hepatitis C and B virus infections in a hemodialysis center.	Selection of study participants with already HCV result known
258	Konstantinidou, 2021	The prevalence of HCV RNA positivity in anti-HCV antibodies-negative hemodialysis patients in Thrace Region. Multicentral study.	Selection of study participants with already HCV antibody result known
259	Kopeć, 2010	[Coincidence of HCV and HGV infections in hemodialysis patients].	Article not in English or in French
260	Korcáková, 1995	[Hepatitis C virus antibodies in hemodialyzed patients].	Article not in English or in French
261	Kosaraju, 2013	Hepatitis viruses in heamodialysis patients: an added insult to injury?	No data on HCV prevalence in HD patients
262	Krautzig, 1994	Hepatitis-C virus (HCV) in peritoneal dialysis.	Full text or abstract not found
263	Ksiaa Cheikhrouhou, 2015	Natural evolution of hepatitis C virus infection in hemodialysis Tunisian patients and CTLA-4 SNP's.	No data on HCV prevalence in HD patients
264	Kühnl, 1990	[Hepatitis C virus antibodies (HCV) in patients treated with chronic hemodialysis].	Article not in English or in French
265	Kulkarni, 1995	Hepatitis C infection in end-stage renal disease patients on maintenance hemodialysis.	Full text or abstract not found
266	Kumagai, 2005	Hepatitis C virus infection in 2,744 hemodialysis patients followed regularly at nine centers in Hiroshima during November 1999 through February 2003.	Not possible to extract data on HCV prevalence
267	Kumar, 1997	Hepatitis C Virus Infection among Hemodialysis Patients in the Najran Region of Saudi Arabia.	No baseline data for longitudinal study
268	Ladino, 2016	Hepatitis C Virus Infection in Chronic Kidney Disease.	Review
269	Ladino, 2018	Hepatitis C Virus Infection in ESKD Patients.	Case report
270	Lampe, 1997	Infection with GB virus C/hepatitis G virus in Brazilian hemodialysis and hepatitis patients and asymptomatic individuals.	No data on HCV prevalence in HD patients
271	Laporte, 2009	Mathematical modeling of hepatitis C virus transmission in hemodialysis.	No data on HCV prevalence in HD patients
272	Laskari, 1995	Hepatitis C: Prevalence and transmission risk in a renal unit.	No data on HCV prevalence in HD patients
273	Latty, 1973	Viral hepatitis in home hemodialysis.	No data on HCV prevalence in HD patients
274	Lazzarini, 2000	[Incidence hepatitis C virus seroconversion after the implementation of a prevention and control program at a hemodialysis unit].	No data on HCV prevalence in HD patients
275	Le Pogam, 1998	Hepatitis C in a hemodialysis unit: molecular evidence for nosocomial transmission.	No data on HCV prevalence in HD patients

276	Leão, 2010	[Infection by hepatitis C virus in patients on hemodialysis: prevalence and risk factors].	Article not in English or in French
277	Leblebicioğlu, 1993	[Hepatitis markers in hemodialysis patients].	Article not in English or in French
278	Lee, 2022	Trends of treated hepatitis B, hepatitis C, and tuberculosis infection in long-term hemodialysis patients in Taiwan: A nationwide survey in 2010-2018.	No data on HCV prevalence in HD patients
279	Leung, 2005	Viral hepatitis C in Hong Kong.	Not possible to extract data on HCV prevalence
280	Li, 1992	[Hepatitis C virus infection in hemodialysis patients].	No data on HCV prevalence in HD patients
281	Li, 2007	Analysis of hepatitis B, C infection in maintenance hemodialysis patients	Full text or abstract not found
282	Lin, 2008	Analysis of hepatitis virus infection in hemodialysis patients.	Full text or abstract not found
283	Ling, 2014	Analysis of HCV infection in hemodialysis patients and its influencing factors.	Full text or abstract not found
284	Liu, 2018	Hepatitis C virus outbreak in a hemodialysis unit of a Primary Hospital: Infection investigation and treatment follow-up.	No data on HCV prevalence in HD patients
285	Liu, 2007	Clinical studies of hemodialysis access through formaldehyde-fixed arterial allografts.	No data on HCV prevalence in HD patients
286	Liu, 2013	Summaries for patients. Pegylated interferon with or without ribavirin for patients with hepatitis C receiving hemodialysis.	No data on HCV prevalence in HD patients
287	Liu, 2022	Hepatitis C virus reinfection in patients on haemodialysis after achieving sustained virologic response with antiviral treatment.	No data on HCV prevalence in HD patients
288	Liu, 1996	Study on HCV infection in hemodialysis patients.	Full text or abstract not found
289	Liu, 1998	Clinical Characteristics of hepatitis B and C viral infection in hemodialysis patients.	Full text or abstract not found
290	Liu, 2005	Clinical study on 120 patients with maintenance hemodialysis complicated with viral hepatitis.	Full text or abstract not found
291	Liu, 2001	Investigation of HBV infection in hemodialysis patients	Full text or abstract not found
292	Liu, 2010	Detection and analysis of hepatitis virus infection in hemodialysis patients.	Full text or abstract not found
293	Liu, 2012	Analysis of viral hepatitis infection in hemodialysis patients	Full text or abstract not found
294	Lok, 1993	Antibody response to core, envelope and nonstructural hepatitis C virus antigens: comparison of immunocompetent and immunosuppressed patients.	No data on HCV prevalence in HD patients
295	Lok, 1991	Overestimation of the prevalence of antibody to hepatitis C virus in retrospective studies on stored sera.	No data on HCV prevalence in HD patients
296	Lombardi, 1995	Is the dialysis membrane a safe barrier against HCV infection?	No data on HCV prevalence in HD patients
297	Lombardi, 1999	Results of a national epidemiological investigation of HCV infection in dialysis patients.	Not possible to extract data on HCV prevalence

298	Loza Munárriz, 2005	[Rate of serological markers of hepatitis B and C viruses in first-time users of the hemodialysis program at Hospital Nacional Cayetano Heredia (HNCH)].	No data on HCV prevalence in HD patients
299	Lugon, 2018	A Web-Based Platform to Collect Data from ESRD Patients Undergoing Dialysis: Methods and Preliminary Results from the Brazilian Dialysis Registry.	Not possible to extract data on HCV prevalence
300	Lusida, 2003	Genotype and subtype analyses of hepatitis B virus (HBV) and possible co-infection of HBV and hepatitis C virus (HCV) or hepatitis D virus (HDV) in blood donors, patients with chronic liver disease and patients on hemodialysis in Surabaya, Indonesia.	No data on HCV prevalence in HD patients
301	Lyon, 2017	Impact of hepatitis C infection in haemodialysis patients is underestimated, finds international study.	Comment on an article
302	Machida, 1992	High incidence of hepatitis C virus antibodies in hemodialysis patients.	Comment on an article
303	Maggi, 1992	Is transfusion the only risk factor for HCV infection among hemodialyzed patients?	Comment on an article
304	Mahaba, 1999	The prevalence of antibodies to hepatitis C virus in Hail region, Saudi Arabia.	Not possible to extract data on HCV prevalence
305	Mahajan, 2017	Utility of hepatitis C virus RNA as the screening test for diagnosing hepatitis C virus infection in hemodialysis patients.	Comment on an article
306	Mahmoud A, 1997	Transmission of HCV among maintenance hemodialysis patients.	Not possible to extract data on HCV prevalence
307	Mahmud, 2014	Hemodialysis patients profile at Dow University of Health Sciences, Karachi. Pakistan.	Selection of study participants with already HCV result known
308	Makhloogh, 2008	Hepatitis C prevalence in hemodialysis patients in Mazandaran, Iran: A survey by polymerase chain reaction and serological methods.	Duplicates
309	Malyszko, 2018	The prevalence of hepatitis C and B among patients on hemodialysis and on renal transplantation waiting list in Poland has significantly decreased during the last 10 years.	Not possible to extract data on HCV prevalence
310	Manescalchi, 1992	Anti-hepatitis C virus epidemiological study in two dialysis centers in Florence.	Not possible to extract data on HCV prevalence
311	Mao, 2013	Investigation and analysis of HCV infection in hemodialysis patients	Full text or abstract not found
312	Marchesi, 2000	Apparent epidemic of HCV infection in a haemodialysis unit [5].	Not possible to extract data on HCV prevalence
313	Marinaki, 2015	Hepatitis C in hemodialysis patients.	Review
314	Martínez, 1991	[Hepatitis C virus infection in different risk groups and among blood donors].	No data on HCV prevalence in HD patients
315	Mashragi, 2014	HIV transmission at a Saudi Arabia hemodialysis unit.	No data on HCV prevalence in HD patients
316	Matar, 1996	Genotyping of hepatitis C virus isolates from Lebanese hemodialysis patients by reverse transcription-PCR and restriction fragment length polymorphism analysis of 5' noncoding region.	No data on HCV prevalence in HD patients
317	Matesanz, 1983	Incidence, diagnosis and evolution of 'non A-non B' hepatitis in haemodialysis units.	No data on HCV prevalence in HD patients
318	Mauro, 2002	[Hepatitis C dissemination by hemodialysis: default isolation versus universal hygienic precautions (reply)].	Full text or abstract not found
319	Mbaeyi, 2013	Hepatitis C virus screening and management of seroconversions in hemodialysis facilities.	No data on HCV prevalence in HD patients

320	McElborough, 2001	Possible cross-infection with hepatitis C virus of an unusual genotype on a haemodialysis unit.	No data on HCV prevalence in HD patients
321	McLaughlin, 1997	Nosocomial transmission of hepatitis C virus within a British dialysis centre.	No data on HCV prevalence in HD patients
322	Meers, 2001	The prevalence and incidence of hepatitis C virus infections among dialysis patients in The Netherlands: a nationwide prospective study.	Full text or abstract not found
323	Mehta, 1999	Hepatitis C virus and hemodialysis.	Full text or abstract not found
324	Mehta, 2022	POS-261 To study the prevalence, biochemical, clinical profile and management of patients with Nephrogenic Ascites; An experience from haemodialysis centre of Northern India.	No data on HCV prevalence in HD patients
325	Menon, 2019	Blood-borne viral infections in pediatric hemodialysis.	Review
326	Mesjasz, 2004	[Does long-term erythropoietin therapy influence the prevalence of serum markers of hepatitis B and C in haemodialysed uraemic patients?].	Article not in English or in French
327	Miedouge, 2010	Analytical evaluation of HCV core antigen and interest for HCV screening in haemodialysis patients.	Selection of study participants with already HCV result known
328	Miller, 2005	Diagnostic discordance for hepatitis C virus infection in hemodialysis: Correlations with clinical and laboratory features - Reply.	Comment on an article
329	Miranda de Menezes , 2021	Brazilian dialysis survey 2019	No data on HCV prevalence in HD patients
330	Miranda de Menezes , 2020	Brazilian Dialysis Census: analysis of data from the 2009–2018 decade.	No data on HCV prevalence in HD patients
331	Mohamed, 2010	Prevention of hepatitis C virus in hemodialysis patients: five years experience from a single center.	No baseline data for longitudinal study
332	Mohammad-Alizadeh, 2002	The frequency of hepatitis in dialyse patients in Hamadan Ekbatan Hospital.	Full text or abstract not found
333	Molares, 1997	Prevalence and seroconversion to HCV in hemodialyzed patients, and epidemiological factors.	Full text or abstract not found
334	Molle, 2002	Hepatitis C infection in children and adolescents with end-stage renal disease.	Not possible to extract data on HCV prevalence
335	Monsalve-Castillo, 2007	Low prevalence of hepatitis C virus infection in Amerindians from Western Venezuela.	No data on HCV prevalence in HD patients
336	Montagnac, 1994	[Hepatitis C in hemodialysis: contribution of a serum bank for the evaluation of risk factors].	Full text or abstract not found
337	Mora Remón, 2012	Decreased prevalence and incidence of HCV markers in haemodialysis units.	Not possible to extract data on HCV prevalence
338	Moukeh, 2009	Epidemiology of hemodialysis patients in Aleppo city.	No data on HCV prevalence in HD patients
339	Moutinho, 2006	Low HBV-DNA levels in end-stage renal disease patients with HBeAg-negative chronic hepatitis B.	No data on HCV prevalence in HD patients
340	Mukhtar, 2019	Epidemiologic features of a large hepatitis C cohort evaluated in a major health system in the western United States.	No data on HCV prevalence in HD patients
341	Mukomolov, 2014	[Molecular epidemiology of hepatitis C in centers of hemodialysis in St. Petersburg].	No data on HCV prevalence in HD patients

342	Munganda, 2017	A prospective observational study on prevalence of hepatitis c virus infection and its risk factors in CKD patients on hemodialysis at Faridabad, Delhi NCR, India.	No data on HCV prevalence in HD patients
343	Nactchigal, 1996	Prevention of hepatitis C virus infection in a haemodialysis centre.	Comment on an article
344	Naman, 1996	[Hepatitis C virus in hemodialysis patients and blood donors in Lebanon].	No data on HCV prevalence in HD patients
345	Naret, 1982	Prevention of hepatitis in a hemodialysis unit.	Full text or abstract not found
346	Nassr, 2014	HCV seroconversion in two Egyptian hemodialysis units: Role of detection method and patients isolation.	No data on HCV prevalence in HD patients
347	Natov, 1996	Hepatitis C in dialysis patients.	Review
348	Natov, 2005	Hepatitis C virus in chronic dialysis patients.	Review
349	Navarro, 1995	Hepatitis B vaccine, hepatitis C virus infection, and haemodialysis.	Review
350	Németh, 1992	[Determination of hepatitis C antibodies, using the Abbott and the Ortho anti-HCV EIA kits, in chronic liver diseases and patients under hemodialysis for chronic renal failure].	Not possible to extract data on HCV prevalence
351	Neves, 2020	Brazilian Dialysis Census: analysis of data from the 2009-2018 decade.	No data on HCV prevalence in HD patients
352	Neves, 1992	Should patients with hepatitis C virus antibodies in chronic hemodialysis be isolated?	Comment on an article
353	Nguyen, 2016	A Large Outbreak of Hepatitis C Virus Infections in a Hemodialysis Clinic.	No data on HCV prevalence in HD patients
354	Nguyen, 2014	Prevalence of hepatitis virus and some clinical features, clinical manifestions of hepatitis in chronic renal failure patients on dialysis cycle.	Article not in English or in French
355	Nguyen, 2013	Effectiveness of modified priming protocol on hepatitis C infection situation among patients receiving maintenance hemodialysis.	Article not in English or in French
356	Nguyen, 2012	Epidemiology of hepatitis B and C virus infection in chronic hemodialysis: a study during 6 years. Y	Article not in English or in French
357	Nguyen, 2008	Hepatitis C virus infection and preventive methods of cross transmission at the Hemodialysis unit, Bach Mai hospital between 2001 and 2006 [PhD dissertation]. Hanoi: Hygiene Sociology and Health Administration, Hanoi Medical University, 2008. (In Vietnamese.)	Article not in English or in French
358	Nguyen, 1195	Primary results of investigating the infection caused by hepatitis C virus in some objects related to blood transfusion.	Article not in English or in French
359	Niemczyk, 1996	[Viral hepatitis type B and C in patients with chronic renal failure treated with dialysis].	Review
360	Niu, 1999	Analysis of infection status and related factors of HBV- HCV in hemodialysis patients.	Full text or abstract not found
361	Nkup, 2018	Prevalence of Hepatitis B Virus Infection Among Patients with Chronic Kidney Disease in Jos University Teaching Hospital (JUTH) Jos, Nigeria. A	No data on HCV prevalence in HD patients
362	No author listeD, 1996	Hepatitis C virus (HCV) infection and disease in nephrology, dialysis and transplantation. Proceedings of the GAMBRO annual symposium. Saint-Etienne, France, September 14-15, 1995.	Full text or abstract not found
363	No author listed, 1999	Hepatitis C and G virus in hemodialysis patients in Syria.	Duplicates
364	No author listed, 2002	Section VI. Haemodialysis-associated infection.	Full text or abstract not found

365	No author listed, 2003	The current state of chronic dialysis treatment in Japan (as of December 31, 2000).	No data on HCV prevalence in HD patients
366	Noh, 1998	Hepatitis G virus infection in hemodialysis and continuous ambulatory peritoneal dialysis patients.	No data on HCV prevalence in HD patients
367	Noiri, 2001	Hepatitis C virus in blood and dialysate in hemodialysis.	No data on HCV prevalence in HD patients
368	Nyirenda M, 2008	Prevalence of infection with hepatitis B and C virus and coinfection with HIV in medical inpatients in Malawi	No data on HCV prevalence in HD patients
369	Okubo, 1996	Hepatitis C virus infection among maintenance hemodialysis patients: a preventable problem of the world.	No data on HCV prevalence in HD patients
370	Okuda, 1998	Acute hepatitis C among renal failure patients on chronic haemodialysis.	No data on HCV prevalence in HD patients
371	Oliveira, 2001	Diferences in HCV antibody patterns in haemodialysis patients infected with the same virus isolate.	Selection of study participants with already HCV result known
372	Oliveira Penido, 2008	[The seroprevalence of HCV in patients submitted to hemodialysis and health professionals in the State of Minas Gerais, southwest of Brazil].	Not possible to extract data on HCV prevalence
373	Olmer, 1997	Transmission of the hepatitis C virus in an hemodialysis unit: evidence for nosocomial infection.	No data on HCV prevalence in HD patients
374	Othman, 2001	Prevalence of hepatitis C virus antibodies among health care workers in Damascus, Syria.	No data on HCV prevalence in HD patients
375	Ozaki, 1999	[Detection of hepatitis C virus antibodies in a pool of 5 sera: their use in seroepidemiologic studies].	No data on HCV prevalence in HD patients
376	Ozdemir, 2005	Relationship between iron replacement and hepatic functions in hepatitis C virus-positive chronic haemodialysis patients.	No data on HCV prevalence in HD patients
377	Ozdoğan, 1997	Histopathological impacts of hepatitis virus infection in hemodialysis patients: should liver biopsy be performed before renal transplantation?	No data on HCV prevalence in HD patients
378	Ozyilkan, 1994	Characteristics of chronic hepatitis C virus infection in patients on maintenance haemodialysis.	No data on HCV prevalence in HD patients
379	Padmanabhan, 1994	Hepatitis C virus infection in haemodialysis patients in saudi arabia.	No data on HCV prevalence in HD patients
380	Paniagua, 2010	Elevated prevalence of hepatitis B in Mexican hemodialysis patients. A multicentric survey.	No data on HCV prevalence in HD patients
381	Papadopoulos, 2018	HCV viraemia in anti-HCV-negative haemodialysis patients: Do we need HCV RNA detection test?	Selection of study participants with already HCV result known
382	Parlati, 2019	HCV-infected dialysis patients: History is changing.	Review
383	Patrice, 2013	Prevalence of Hepatitis B surface antigen and anti-HIV antibodies among patients on maintenance haemodialysis in Douala, Camroon.	No data on HCV prevalence in HD patients
384	Patti, 1998	Nosocomial transmission of HCV in hemodialysis centres.	Full text or abstract not found

385	Patti, 1994	[The prevalence of anti-HCV antibodies in the sera from dialysis patients collected in 1980].	Article not in English or in French
386	Pellerey, 1996	[Epidemiology and clinical course in hepatitis C-positive patients treated with dialysis in Piemonte. Work group of the RPDT].	Selection of study participants with already HCV result known
387	Penido, 2008	The Seroprevalence of HCV in patients submitted to hemodialysis and health professionals in State of Minas Gerais, Southest of Brazil.	Not possible to extract data on HCV prevalence
388	Perboni, 2009	[Blood-borne infections in hemodialysis: facts and opinions].	Review
389	Pereira, 1999	Hepatitis C virus infection in dialysis: a continuing problem.	Review
390	Pereira, 1997	Hepatitis C virus infection in dialysis and renal transplantation.	Review
391	Pereira, 1998	Hepatitis C in dialysis.	Review
392	Pereira , 1179	Effect of hepatitis C infection and renal transplantation on survival in end-stage renal disease.	No data on HCV prevalence in HD patients
393	Perez, 2003	Unexpected distribution of hepatitis C virus genotypes in patients on hemodialysis and kidney transplant recipients.	Selection of study participants with already HCV result known
394	Petrarulo, 1992	HCV infection occupational hazard at dialysis units and virus spread, among relatives of dialyzed patients.	Selection of study participants with already HCV result known
395	Petrosillo, 1993	Hepatitis C transmission in dialysis.	No data on HCV prevalence in HD patients
396	Phan, 1995	The problem of HCV infection at Cho Ray hospital.	Article not in English or in French
397	Piazza, 1999	Long-term persistence of hepatitis C viremia in hemodialysis patients.	Full text or abstract not found
398	Podlasin, 2005	[Hepatitis C infections in dialyzed patients].	No data on HCV prevalence in HD patients
399	Poignet, 1997	[Hepatitis c virus infection in dialysis: necessity of prevention and screening].	Full text or abstract not found
400	Pol, 1995	Hepatitis C virus infection in hemodialyzed patients and kidney allograft recipients.	Selection of study participants with already HCV result known
401	Pol, 1994	[Viral hepatitis in hemodialysis and renal transplantation patients].	Review
402	Pol, 1993	Hepatitis C virus RNA in anti-HCV positive hemodialyzed patients: significance and therapeutic implications.	Selection of study participants with already HCV result known
403	Pol, 2002	HCV infection and hemodialysis.	Review
404	Polakoff, 1972	Hepatitis in dialysis units in the United Kingdom.	No baseline data for longitudinal study
405	Polenakovic, 2007	Hepatitis C in dialysis patients.	Review
406	Polz, 1995	[Infection with HCV in patients hospitalized for various causes. I. Prevalence of anti-HCV antibodies in selected groups of patients].	Article not in English or in French
407	Ponnuvel, 2021	Clinical utility of hepatitis C virus core antigen (HCVcAg) assay to identify active HCV infection in hemodialysis and renal transplant patients.	No data on HCV prevalence in HD patients

408	Ponz, 2001	Hepatitis C virus antibodies in patients on hemodialysis and after kidney transplantation.	Full text or abstract not found
409	Prnjavorac, 2003	[Seroconversion makers of hepatitis in the dialysis center at the General Hospital in Tesanj (10 years' experience)].	No data on HCV prevalence in HD patients
410	Pru, 1994	Hepatitis C transmission through dialysis machines.	No data on HCV prevalence in HD patients
411	Pugliese, 1999	Quantitative assessment of HCV load in chronic hemodialysis patients.	Full text or abstract not found
412	Puttinger, 2002	Hepatitis B and C in peritoneal dialysis patients.	No data on HCV prevalence in HD patients
413	Qadi, 2004	Hepatitis B and hepatitis C virus prevalence among dialysis patients in Bahrain and Saudi Arabia: a survey by serologic and molecular methods.	Duplicates
414	Rabanal, 2010	Impact of hepatitis C in mortality in patients on hemodialysis.	No data on HCV prevalence in HD patients
415	Radović, 1996	[Incidence and clinical manifestations of hepatitis C virus infection in patients on hemodialysis].	Not possible to extract data on HCV prevalence
416	Rahman, 1995	Hepatitis B and hepatitis C virus study in patients suffering from end stage renal failure on maintenance haemodialysis.	Full text or abstract not found
417	Ramatillah, 2018	Hepatitis C Infection Become a Common Issue Among Hemodialysis Patients in a Hemodialysis Center Jakarta, Indonesia, and Survival Comparison of Hemodialysis Patients with Hepatitis Infection between Two Hemodialysis Centers in Jakarta, Indonesia, and Penang, Malaysia.	No baseline data for longitudinal study
418	Ramezani, 2014	Occult HCV infection in hemodialysis patients with elevated liver enzymes.	Selection of study participants with already HCV result known
419	Ramezani, 2010	Hepatitis B prevalence of latent infection in hemodialysis patients.	Full text or abstract not found
420	Rampino, 2000	Hepatitis C virus in hemodialysis patients - Reply from the authors.	Comment on an article
421	Rao, 2013	Outbreak of hepatitis C virus infections at an outpatient hemodialysis facility: the importance of infection control competencies.	Full text or abstract not found
422	Reddy, 2005	Prevalence of HCV infection in patients on haemodialysis: survey by antibody and core antigen detection.	Selection of study participants with already HCV result known
423	Resic, 2001	[A high prevalence of hepatitis C in hemodialysis].	Article not in English or in French
424	Resić, 2003	[Prevalence and incidence of hepatitis C seroconversion in patients on hemodialysis].	Article not in English or in French
425	Rezaee-Zavareh, 2015	Occult hepatitis C virus infection in dialysis patients: does it need special attention?	Comment on an article
426	Robles, 1992	Lack of transmission of hepatitis C virus in a haemodialysis unit.	No baseline data for longitudinal study
427	Robles, 1993	[The low contagiousness of the hepatitis C virus in hemodialysis].	Article not in English or in French
428	Rodríguez, 1993	[Prevalence of hepatitis C virus antibodies in a hemodialysis unit].	Article not in English or in French

429	Rodriguez , 2003	Determinants of survival among HIV-infected chronic dialysis patients.	No data on HCV target detected
430	Roy, 2012	Epidemiology and molecular investigation of hepatitis C infection following holiday haemodialysis.	No data on HCV prevalence in HD patients
431	Saab, 2001	Hepatitis C virus transmission in the hemodialysis community.	No data on HCV prevalence in HD patients
432	Saab, 2001	Hepatitis C screening strategies in hemodialysis patients.	No data on HCV prevalence in HD patients
433	Saboor, 2003	Prevalence and risk factors of hepatitis C infection in hemodialysis patients (Kermanshah, 1999–2000).	Article not in English or in French
434	Saha, 2001	Hepatitis and HIV infection during haemodialysis.	Full text or abstract not found
435	Sahin, 2003	Does hepatitis C virus infection increase hematocrit and hemoglobin levels in hemodialyzed patients?	Selection of study participants with already HCV result known
436	Saifan, 2013	Effect of hepatitis C virus infection on erythropoiesis in patients on hemodialysis.	Selection of study participants with already HCV result known
437	Sakellariou, 2014	Histological features of chronic hepatitis C in haemodialysis patients.	Selection of study participants with already HCV result known
438	Salou, 2019	Prevalence and Factors Associated with Hepatitis B Virus Surface Antigen and Human Immunodeficiency Virus Antibodies in Chronic Hemodialysis Patients Followed-Up in Lome´, To	No data on HCV prevalence in HD patients
439	Sampietro, 1996	Nosocomial hepatitis C in dialysis units.	No data on HCV prevalence in HD patients
440	Sampietro, 1995	Single-tube reverse transcription and heminested polymerase chain reaction of hepatitis C virus RNA to detect viremia in serologically negative hemodialysis patients.	Duplicates
441	Santana, 2001	[Antibodies to hepatitis C virus in patients undergoing hemodialysis in Salvador, BA, Brazil].	Article not in English or in French
442	Santos, 2017	Prevalence and risk factors for human t-lymphotropic virus type 1 (HTLV-1) among maintenance hemodialysis patients	No data on HCV target detected
443	Saracho, 2015	Clinical evolution of chronic renal patients with HIV infection in replacement therapy	Not possible to extract data on HCV prevalence
444	Sarhan, 2015	Prevalence of hepatitis C virus seroconversion among hemodialysis patients in Egypt. E	Full text or abstract not found
445	Sarkari, 2012	High prevalence of hepatitis C infection among high risk groups in Kohgiloyeh and Boyerahmad Province, Southwest Iran.	No data on HCV prevalence in HD patients
446	Sartor, 2004	Transmission of hepatitis C virus between hemodialysis patients sharing the same machine.	No data on HCV prevalence in HD patients
447	Saxena, 2002	Nosocomial transmission of hepatitis C virus: impact of strict isolation on annual seroconversion rate in a hemodialysis unit.	No data on HCV prevalence in HD patients
448	Saxena, 2003	Remarkable resemblance in the mode of transmission of HCV infection among haemodialysis patients and IVDAs.	No data on HCV prevalence in HD patients

449	Schneeberger, 1999	[Nosocomial transmission of hepatitis C virus in a Dutch dialysis center].	No data on HCV prevalence in HD patients
450	Schneeberger, 1995	[Hepatitis-C virus antibody conversion in 3 hemodialysis patients in various dialysis departments].	No data on HCV prevalence in HD patients
451	Scotto, 1999	Hepatitis C virus infection in four haemodialysis units of southern Italy: epidemiological report.	Selection of study participants with already HCV result known
452	Senatore, 2016	Hepatitis C virus outbreak in a haemodialysis unit: learning from failures.	No data on HCV prevalence in HD patients
453	Sesso, 2017	Brazilian chronic dialysis survey 2016	No data on HCV prevalence in HD patients
454	Seyrafian, 2006	Comparison and prevalence of hepatitis B and C infection and hepatitis B vaccination in hemodialysis patients and staffs in 13 hemodialysis centers in Isfahan (Iran).	Full text or abstract not found
455	Sfar, 2009	Prevalence of autoantibodies in a Tunisian cohort of hepatitis C virus infected dialysis patients.	Selection of study participants with already HCV result known
456	ShadAfzar, 2007	Prevalence and Incidence of Hepatitis C in Hemodialysis Patients in Bo-Ali Medical Center in Qazvin in 2003–2004.	Full text or abstract not found
457	Shafi, 2017	Frequency of Hepatitis C in hospitalized patients with chronic kidney disease.	No data on HCV prevalence in HD patients
458	Shafi, 2003	Prevalence and rate of seroconversion of hepatitis C in hemodialysis patients.	No baseline data for longitudinal study
459	Shafiq, 2002	Prevalence of hepatitis C in patients with end-stage renal disease before and during hemodialysis.	Full text or abstract not found
460	Shakhgil'dian, 1994	[Risk of infection with hepatitis B and C viruses of medical workers, patients in the hemodialysis ward, and vaccine prophylaxis of hepatitis B infection in these populations].	No data on HCV prevalence in HD patients
461	Shimokura, 1998	Hepatitis C virus in hemodialysis centers.	Full text or abstract not found
462	Shusterman, 1987	Infectious hepatitis in dialysis patients.	No data on HCV prevalence in HD patients
463	Simon, 1995	Hepatitis C virus infection in hemodialysis patients.	Review
464	Singh, 2011	Seroprevalence of hepatitis B and C in HIV seropositive and chronic renal failure patients in North India.	No data on HCV prevalence in HD patients
465	Smalcelj, 1995	Prevention of hepatitis C infection in haemodialysis units.	Comment on an article
466	Soin, 2015	Hepatitis C virus infection in dialysis patients: a retropspective study from a tertiary care hospital of north India.	Full text or abstract not found
467	Soliman, 2013	Evaluation of an isolation program of hepatitis C virus infected hemodialysis patients in some hemodialysis centers in egypt.	No baseline data for longitudinal study
468	Soresi, 1994	[The prevalence of anti-HCV antibodies in a population of Sicilian hemodialysis patients].	Article not in English or in French
469	Soto-Salgado, 2009	Factors associated to the prevalence of antibodies to hepatitis C virus among patients receiving hemodialysis at selected dialysis centers in Puerto Rico, 2005.	Full text or abstract not found
470	Souqiyyeh, 2001	Dialysis centers in the kingdom of saudi arabia.	Not possible to extract data on HCV prevalence

471	Soyannwo, 1996	Hepatitis C antibodies in haemodialysis and pattern of end-stage renal failure in Gassim, Saudi Arabia.	Full text or abstract not found
472	Spada, 2008	Molecular epidemiology of a hepatitis C virus outbreak in a hemodialysis unit in Italy.	No baseline data for longitudinal study
473	Sterling, 1999	Chronic hepatitis C infection in patients with end stage renal disease: characterization of liver histology and viral load in patients awaiting renal transplantation.	No data on HCV prevalence in HD patients
474	Stradtmann, 1987	[Significance of viral hepatitis in a dialysis center for patients and health personnel--a 5-year review].	Article not in English or in French
475	Stuyver, 1996	Hepatitis C virus in a hemodialysis unit: molecular evidence for nosocomial transmission.	No baseline data for longitudinal study
476	Sud, 2013	Hepatitis C virus infection during haemodialysis on vacation: A series of 3 cases.	Not possible to extract data on HCV prevalence
477	Sułowicz, 2007	Hepatitis C virus infection in dialysis patients.	Review
478	Sungur, 1994	Is HCV a nosocomial infection in haemodialysis patients?	Comment on an article
479	Sungur, 1995	Nosocomial transmission of hepatitis C virus to hemodialysis patients: molecular epidemiology by polymerase chain reaction.	Selection of study participants with already HCV result known
480	Sungur, 1993	Evidence of transmission of hepatitis C by hemodialysis.	No data on HCV prevalence in HD patients
481	Svára, 2001	[Viral hepatitis of patients in a regular haemodialysis programme].	Review
482	Sychev, 1993	[The hepatitis C viral infection of the medical workers and patients at hemodialysis units in Moscow].	Article not in English or in French
483	Takahashi, 1988	[Incidence of non-A, non-B hepatitis at a hemodialysis unit].	Article not in English or in French
484	Tan, 1997	[Chronic hepatitis C in hemodialysis patients].	Review
485	Tanaka, 2004	[Chronic hepatitis C in hemodialysis patients].	Article not in English or in French
486	Tanaka, 2003	[Examination of HCV infection in hemodialysis patients with HCV-RNA assay using a magnetic extraction method (AmpliCap GT HCV MONITOR Test Kit v2.0 and AmpliCap GT HCV Specimen Preparation Kit v2.0)].	Article not in English or in French
487	Tanaka, 2004	Clinical usefulness of a new hepatitis C virus RNA extraction method using specific capture probe and magnetic particle in hemodialysis patients.	Selection of study participants with already HCV result known
488	Taskapan, 2001	Patient to patient transmission of hepatitis C virus in hemodialysis units.	Full text or abstract not found
489	Teles, 1998	Hepatitis B virus infection profile in central Brazilian hemodialysis population.	No data on HCV prevalence in HD patients
490	Teles, 2002	Hepatitis B virus transmission in Brazilian hemodialysis units: Serological and molecular follow-up.	No data on HCV prevalence in HD patients
491	Terrault, 2018	Hepatitis C in Patients With RenalDisease: A Deeper Dive Into the KDIGO Guideline.	Review
492	Teruel, 1992	Importance of nosocomial transmission of hepatitis C virus infection in dialysis units.	Full text or abstract not found
493	Thomé, 2017	Brazilian chronic dialysis survey 2017	Not possible to extract data on HCV prevalence

494	Thompson, 2009	Hepatitis C virus transmission in hemodialysis units: importance of infection control practices and aseptic technique.	Not possible to extract data on HCV prevalence
495	Thomson, 2011	A case of hepatitis C virus transmission acquired through sharing a haemodialysis machine.	Case report
496	Timofte, 2020	Infection with hepatitis C virus in hemodialysis patients: An overview of the diagnosis and prevention rules within a hemodialysis center (Review).	Review
497	Tong, 2006	Hepatitis virus infection in maintenance hemodialysis patients.	Full text or abstract not found
498	Toosi, 2008	Prevalence of viral hepatitis in hemodialysis patients in Tehran, Iran.	Not possible to extract data on HCV prevalence
499	Tran, 2008	Hepatitis B virus infection among hemodialysis patients [Master dissertation]. Ho Chi Minh City: Internal Medicine, University of Medicine and Pharmacy at Ho Chi Minh City, 2008. (In Vietnamese.)	Article not in English or in French
500	Trevizoli, 2008	Hepatitis C is less aggressive in hemodialysis patients than in nonuremic patients.	Selection of study participants with already HCV result known
501	Tsuyuguchi, 1995	[Prevalence of hepatitis C virus infection among chronic hemodialysis patients].	Article not in English or in French
502	Tuyuguchi, 2004	[Prevalence of hepatitis C virus infection among chronic hemodialysis patients].	Article not in English or in French
503	Umlauft, 1997	Patterns of hepatitis C viremia in patients receiving hemodialysis.	Selection of study participants with already HCV result known
504	Umlauft, 1997	Hepatitis G virus infection in hemodialysis patients and the effects of interferon treatment.	Selection of study participants with already HCV result known
505	Utsunomiya, 1999	[The prevalence of TTV infection and the route of TTV transmission in hemodialysis patients--compared with HCV infection].	No data on HCV prevalence in HD patients
506	Vagelli, 1992	Effect of HCV+ patients isolation on HCV infection incidence in a dialysis unit.	Selection of study participants with already HCV result known
507	Vagelli, 1984	Non A-non B hepatitis in a dialysis population: spread by dental surgery?	Full text or abstract not found
508	Vagelli, 1987	[Non-A, non-B viral hepatitis in patients in chronic hemodialysis].	Article not in English or in French
509	Vallet-Pichard, 2013	Hepatitis C virus infection in hemodialysis patients.	Review
510	Valtuille, 2002	Decline of high hepatitis C virus prevalence in a hemodialysis unit with no isolation measures during a 6-year follow-up.	Selection of study participants with already HCV result known
511	Vandelli, 1992	Prevalence of hepatitis C virus (HCV) antibodies in haemodialysis patients.	Duplicates
512	Vasiljević, 2000	[Detection of anti-HCV antibodies in patients on long-term hemodialysis].	Article not in English or in French
513	Vedio, 2009	Hepatitis C, a hazard for haemodialysis patients during international travel.	Sample size < or = 10 participants

514	Vlatkovic, 2009	Malnutrition-inflammation complex syndrome and hepatitis C in maintenance hemodialysis patients.	Selection of study participants with already HCV result known
515	Vu, 2011	HCV-RNA and genotype of hepatitis C virus (HCV) in hemodialysis patients at Bach Mai hospital.	Article not in English or in French
516	Wang, 1999	[Nucleotide sequence analysis for high variance region of hepatitis C virus in patients with hemodialysis].	Selection of study participants with already HCV result known
517	Wang, 2003	Clinical investigation of the maintenance hemodialysis patients complicated with viral hepatitis.	Full text or abstract not found
518	Wang, 2004	Prevalence of hepatitis B and C viral infection in uremia patients treated by chronic hemodialysis	Full text or abstract not found
519	Wang, 2012	Analysis of HCV infection related factors in maintenance hemodialysis patients in a primary hospital.	Full text or abstract not found
520	Wang, 2018	Analysis of hepatitis c infection and influencing factors in 120 dialysis patients with type 2 diabetes mellitus and renal failure.	Full text or abstract not found
521	Wang, 2014	Analysis of related factors of hemodialysis hepatitis b and hepatitis c infection and discussion of preventive measures	Full text or abstract not found
522	Washio, 1997	Hepatitis C virus and human T cell leukemia virus type 1 infection without [corrected] blood transfusion in hemodialysis units and its prevention.	Review
523	Washio, 1998	[Blood-borne viral infection in hemodialysis units: special reference to hepatitis B virus, hepatitis C virus and human T-lymphotropic virus type 1].	Review
524	Weber, 2016	Hepatitis C Virus Outbreaks in Hemodialysis Centers: A Continuing Problem.	Review
525	Webster, 2007	Investigation of hepatitis C transmission in a UK haemodialysis unit: possible role of Schribner shunt vascular access device.	Case report
526	Wiese, 1986	[Viral hepatitis as a nosocomial infection at the dialysis center--a current 5-year study].	Article not in English or in French
527	Wigneswaran, 2018	Hepatitis C virus infection in patients with end-stage renal disease.	Review
528	Winston, 2020	Viral hepatitis in patients on hemodialysis.	Review
529	Wong, 2021	Hepatitis C virus core antigen as alternative diagnostic algorithm for active hepatitis C virus infection among haemodialysis population: Cost implications.	Selection of study participants with already HCV result known
530	Wong, 2020	Hepatitis C core antigen testing to diagnose active hepatitis C infection among haemodialysis patients.	Selection of study participants with already HCV result known
531	Xiao, 1997	Study on HCV infection in hemodialysis patients and dialysis center staff.	Full text or abstract not found
532	Xie, 2007	Comparison of positivity rates of anti-HCV in patients under dialysis	Full text or abstract not found
533	Xu, 2010	Clinical study of viral hepatitis in 150 patients with maintenance hemodialysis.	Full text or abstract not found
534	Yaari, 2006	Detection of HCV salivary antibodies by a simple and rapid test.	Selection of study participants with already HCV result known
535	Yamaguchi, 1994	Seroepidemiology of hepatitis C virus infection in Japan and HCV infection in haemodialysis patients.	Review
536	Yan, 2012	Risk factors of HCV infection in hemodialysis patients in a hospital in Changsha.	Full text or abstract not found

537	Yanai, 2001	[Viral hepatitis in hemodialysis patients].	Review
538	Yang, 2003	Isolation effectively prevents the transmission of hepatitis C virus in the hemodialysis unit.	Selection of study participants with already HCV result known
539	Yang, 2017	[Current status of the prevalence, diagnosis, and treatment of hepatitis C in patients undergoing hemodialysis].	Article not in English or in French
540	Yang, 2012	A multicenter clinical study of HCV infection in hemodialysis patients.	Full text or abstract not found
541	Yespotayeva, 2022	THE EPIDEMIOLOGY OF HEPATITIS B AND C INFECTION IN HEMODIALYSIS PATIENTS IN KAZAKHSTAN.	No data on HCV prevalence in HD patients
542	Yin, 1994	Observation of hepatitis virus infection in hemodialysis patients.	Full text or abstract not found
543	Yoo, 2013	Prevalence of occult hepatitis B virus infection in hemodialysis patients.	No data on HCV prevalence in HD patients
544	Yücel, 2004	Bone mineral density in patients on maintenance hemodialysis and effect of chronic hepatitis C virus infection.	Selection of study participants with already HCV result known
545	Zamani, 2010	Incidence of hepatitis C infection in patients on hemodialysis: a multicenter study of northern part of Iran.	Selection of study participants with already HCV result known
546	Zamir, 2001	Hepatitis C infection in dialysis patients in Israel.	Comment on an article
547	Zampieron, 2006	Sero-conversion of HCV negative patients: a European study on the epidemiology and management of HCV haemodialysis patients.	Selection of study participants with already HCV result known
548	Zeinab, 1994	Prevalence of hepatitis C virus antibodies in hemodialysis patients	Full text or abstract not found
549	Zeng, 2011	Risk factors and prevention of HCV nosocomial infection in hemodialysis patients.	Full text or abstract not found
550	Zeniya, 2015	[Hepatitis C virus infection and hemodialysis: update 2014].	Review
551	Zhang, 1999	Evaluation of the prevalence HCV infection in hemodialysis patients and its infective risk factors.	Full text or abstract not found
552	Zhang, 2013	Serological changes and risk factors of HCV infection in hemodialysis patients: Follow-up data for three years attached.	Full text or abstract not found
553	Zhang, 2010	The occurrence and cause of hepatitis virus infection in hemodialysis patients.	Full text or abstract not found
554	Zhang, 2011	A multicenter clinical study of HCV infection in hemodialysis patients. C	Full text or abstract not found
555	Zhao, 2016	Next Generation Sequencing-Based Investigation of Potential Patient-to-Patient Hepatitis C Virus Transmission during Hemodialytic Treatment.	Selection of study participants with already HCV result known
556	Zhao, 2008	Analysis of hepatitis virus infection in hemodialysis patients.	Full text or abstract not found
557	Zhao, 2010	Investigation of hepatitis c infection in hospital maintenance hemodialysis patients (2008–2009).	Full text or abstract not found
558	Zhu, 2015	Clinical study on HCV infection in hemodialysis patients in changshu district.	Full text or abstract not found
559	Zreiq, 2022	Frequency of Hepatitis C Infection in Hospital Patients in Ha'il, KSA: A Retrospective Analysis.	No data on HCV prevalence in HD patients

560	Zubkin, 2000	[Distribution and features of infection with hepatitis viruses B and C during hemodialysis treatment].	Article not in English or in French
561	Zwolińska, 2001	[Epidemiology of HBV infections and possibilities for therapeutic actions in children and adolescents with end-stage renal failure treated with dialysis].	Article not in English or in French
