# Supplementary material for: Global epidemiology of hepatitis C virus in dialysis patients: A systematic review and meta-analysis
Source: PLoS One. 2024 Feb 8;19(2):e0284169. doi: 10.1371/journal.pone.0284169 (PMC10852299; doi:10.1371/journal.pone.0284169)
Supplement: S5 Table — (PDF) [file pone.0284169.s006.pdf]

S5 Table: Characteristics of included studies

Characteristics	Overall (634)	CFR (8)	Anti-HCV prevalence (559)	Viral RNA prevalence (67)	Viral antigen prevalence (5)	Anti-HCV, Core Ag prevalence (2)
**Year of publication ; range**	1990-2022	1998-2013	1990-2022	1994-2022	2005-2015	2012-2014
**Period of inclusion of participants; range**	1968-2022	1992-2013	1968-2022	1992-2019	2000-2013	2008-2012
**Mean or Median age (years); range**	7.7-72.7	47.3-51.2	7.7-72.7	31.0-70.0	52.7-62.5	NA
**Proportion of male participant; range**	12-95.4	42.6-69.9	12-95.4	30.8-87.2	42-65.2	NA
**Duration since the first dialysis (years); range**	0.2-25.7	3.1-6.0	0.2-25.7	0.5-16.0	1.6-3.0	NA
**Number of dialysis sessions per month; range**	3-16	16	3-16	3.4-12	12	NA
**% Previous blood products transfusion; range**	12.4-100	NA	12.4-100	32.3-89.5	51.4	NA
**% Previous surgical procedure; range**	0-90	NA	0-90	0-12.5	0-8.3	NA
**% Previous dental procedure; range**	0-48.4	NA	0-48.4	0	NA	NA
**% Tattoos; range**	0-41.4	NA	0-41.4	0-2.6	NA	NA
**% Injecting drug users; range**	0-72.7	6.5	0-72.7	0-2.0	NA	NA
**% Piercings; range**	0-41.4	NA	0-41.4	0	NA	NA
**% Scarification; range**	0-17.9	NA	0-17.9	0-15.0	NA	NA
**Study Design**						
Cross-sectional	569 (89.8)	5 (62.5)	504 (90.2)	59 (88.1)	3 (60.0)	2 (100)
Cohort (Baseline data)	60 (9.5)	3 (37.5)	51 (9.1)	7 (10.4)	2 (40.0)	
Case control	4 (0.6)		3 (0.5)	1 (1.5)		
Clinical Trial (Baseline data)	1 (0.2)		1 (0.2)			
**Sampling**						
Non probabilistic	572 (90.2)	7 (87.5)	505 (90.3)	59 (88.1)	5 (100)	2 (100)
Probabilistic	62 (9.8)	1 (12.5)	54 (9.7)	8 (11.9)		
**Setting**						
Hospital-based	634 (100)	8 (100)	559 (100)	67 (100)	5 (100)	2 (100)
**Rural/Urban**						
Urban	367 (57.9)	3 (37.5)	327 (58.5)	37 (55.2)	1 (20.0)	2 (100)
Urban/Rural	32 (5.1)		29 (5.2)	3 (4.5)		
Rural	25 (3.9)		18 (3.2)	7 (10.4)		
Unclear/ Not reported	210 (33.1)	5 (62.5)	185 (33.1)	20 (29.9)	4 (80.0)	
**HCV treatment**						
Antiviral naïve	181 (28.6)		164 (29.3)	17 (25.4)		
Antiviral naïve/ On antiviral	6 (1.0)		5 (0.9)	1 (1.5)		

On antiviral	1 (0.2)		1 (0.2)			
Unclear/ Not reported1	446 (70.4)	8 (100)	389 (69.6)	49 (73.1)	5 (100)	2 (100)
**Timing of samples collection**						
Prospectively	578 (91.2)	6 (75.0)	507 (90.7)	65 (97.0)	5 (100)	1 (50.0)
Retrospectively	55 (8.7)	1 (12.5)	51 (9.1)	2 (3.0)		1 (50.0)
Retroprospectively	1 (0.2)	1 (12.5)	1 (0.2)			
**Countries**						
Italy	70 (11.0)	1 (0.2)	64 (10.1)	5 (0.8)	1 (0.2)	
India	46 (7.3)	1 (0.2)	34 (5.4)	12 (1.9)		
Iran	45 (7.1)	1 (0.2)	38 (6.0)	6 (1.0)	1 (0.2)	
Japan	40 (6.3)	1 (0.2)	35 (5.5)	4 (0.6)	1 (0.2)	
Brazil	37 (5.8)		31 (4.9)	6 (1.0)		
Saudi Arabia	34 (5.4)	1 (0.2)	31 (4.9)	3 (0.5)		
Spain	26 (4.1)		24 (3.8)	2 (0.3)		
United States of America	25 (3.9)	1 (0.2)	25 (3.9)			
Turkey	21 (3.3)		19 (3.0)	2 (0.3)		
Germany	19 (3.0)		16 (2.5)	3 (0.5)		
China	18 (2.8)	1 (0.2)	13 (2.1)	3 (0.5)	1 (0.2)	
Pakistan	17 (2.7)		16 (2.5)	1 (0.2)		
France	14 (2.2)		11 (1.7)	3 (0.5)		
Taiwan	13 (2.1)		12 (1.9)	1 (0.2)		
Morocco	10 (1.6)	1 (0.2)	8 (1.3)	1 (0.2)		1 (0.2)
Egypt	9 (1.4)		8 (1.3)	1 (0.2)		
Tunisia	9 (1.4)		9 (1.4)			
Indonesia	8 (1.3)		8 (1.3)			
Greece	7 (1.1)		4 (0.6)	3 (0.5)		
Poland	7 (1.1)		7 (1.1)			
Romania	7 (1.1)		7 (1.1)			
Jordan	6 (1.0)		6 (1.0)			
Libya	6 (1.0)		6 (1.0)			
Sudan	6 (1.0)		6 (1.0)			
Australia	5 (0.8)		5 (0.8)			
Serbia	5 (0.8)		5 (0.8)			
South Korea	5 (0.8)		5 (0.8)			
Venezuela	5 (0.8)		5 (0.8)			
Argentina	4 (0.6)		3 (0.5)	1 (0.2)		
Netherlands	4 (0.6)		4 (0.6)			

Nigeria	4 (0.6)		4 (0.6)			
Portugal	4 (0.6)		4 (0.6)			
Sweden	4 (0.6)		4 (0.6)			
Thailand	4 (0.6)		2 (0.3)	2 (0.3)		
United Kingdom	4 (0.6)		3 (0.5)	1 (0.2)		
Vietnam	4 (0.6)		1 (0.2)	1 (0.2)	1 (0.2)	1 (0.2)
Yemen	4 (0.6)		4 (0.6)			
Denmark	3 (0.5)		3 (0.5)			
Iraq	3 (0.5)		3 (0.5)			
Israel	3 (0.5)		2 (0.3)	1 (0.2)		
Kosovo	3 (0.5)		3 (0.5)			
Kuwait	3 (0.5)		3 (0.5)			
Peru	3 (0.5)		3 (0.5)			
Senegal	3 (0.5)		3 (0.5)			
South Africa	3 (0.5)		3 (0.5)			
Austria	2 (0.3)		2 (0.3)			
Cameroon	2 (0.3)		2 (0.3)			
Canada	2 (0.3)		2 (0.3)			
Chile	2 (0.3)		2 (0.3)			
Croatia	2 (0.3)		2 (0.3)			
Hungary	2 (0.3)		2 (0.3)			
Mexico	2 (0.3)		1 (0.2)	1 (0.2)		
Palestine	2 (0.3)		1 (0.2)	1 (0.2)		
Singapore	2 (0.3)		2 (0.3)			
Slovenia	2 (0.3)		2 (0.3)			
Syria	2 (0.3)		2 (0.3)			
United Arab Emirates	2 (0.3)		2 (0.3)			
Australia; New Zealand	1 (0.2)		1 (0.2)			
Azerbaijan	1 (0.2)		1 (0.2)			
Bahrain; Saudi Arabia	1 (0.2)			1 (0.2)		
Bangladesh	1 (0.2)		1 (0.2)			
Belgium	1 (0.2)		1 (0.2)			
Bosnia and Herzegovina	1 (0.2)		1 (0.2)			
Botswana	1 (0.2)		1 (0.2)			
Brunei Darussalam	1 (0.2)		1 (0.2)			
Bulgaria	1 (0.2)		1 (0.2)			
Ethiopia	1 (0.2)		1 (0.2)			

Finland	1 (0.2)		1 (0.2)			
French Guiana	1 (0.2)		1 (0.2)			
Kenya	1 (0.2)		1 (0.2)			
Lithuania	1 (0.2)		1 (0.2)			
Malaysia	1 (0.2)			1 (0.2)		
Mali	1 (0.2)		1 (0.2)			
Myanmar	1 (0.2)		1 (0.2)			
Namibia	1 (0.2)		1 (0.2)			
Nepal	1 (0.2)			1 (0.2)		
Philippines	1 (0.2)		1 (0.2)			
Qatar	1 (0.2)		1 (0.2)			
Republic of Ireland	1 (0.2)		1 (0.2)			
Republic of Macedonia	1 (0.2)		1 (0.2)			
Russian Federation	1 (0.2)		1 (0.2)			
Somalia	1 (0.2)		1 (0.2)			
Sri Lanka	1 (0.2)		1 (0.2)			
Switzerland	1 (0.2)		1 (0.2)			
United States Virgin Islands	1 (0.2)		1 (0.2)			
Uzbekistan	1 (0.2)		1 (0.2)			
Yugoslavia	1 (0.2)		1 (0.2)			
**WHO Region**						
Europe	218 (34.4)	1 (0.2)	197 (31.1)	20 (3.2)	1 (0.2)	
Eastern Mediterranean	159 (25.1)	3 (0.5)	144 (22.7)	13 (2.1)	1 (0.2)	1 (0.2)
Western Pacific	91 (14.4)	2 (0.3)	76 (12.0)	10 (1.6)	3 (0.5)	1 (0.2)
America	80 (12.6)	1 (0.2)	72 (11.4)	8 (1.3)		
South-East Asia	62 (9.8)	1 (0.2)	47 (7.4)	15 (2.4)		
Africa	17 (2.7)		17 (2.7)			
Unclear	7 (1.1)		6 (1.0)	1 (0.2)		
**UNSD Region**						
Southern Europe	122 (19.2)	1 (0.2)	111 (17.5)	10 (1.6)	1 (0.2)	
Southern Asia	111 (17.5)	2 (0.3)	90 (14.2)	20 (3.2)	1 (0.2)	
Western Asia	83 (13.1)	1 (0.2)	75 (11.8)	8 (1.3)		
Eastern Asia	76 (12.0)	2 (0.3)	65 (10.3)	8 (1.3)	2 (0.3)	
South America	52 (8.2)		45 (7.1)	7 (1.1)		
Western Europe	41 (6.5)		35 (5.5)	6 (1.0)		
Northern Africa	40 (6.3)	1 (0.2)	37 (5.8)	2 (0.3)		1 (0.2)
Northern America	27 (4.3)	1 (0.2)	27 (4.3)			

Southeastern Asia	22 (3.5)		16 (2.5)	4 (0.6)	1 (0.2)	1 (0.2)
Eastern Europe	18 (2.8)		18 (2.8)			
Northern Europe	14 (2.2)		13 (2.1)	1 (0.2)		
West Africa	8 (1.3)		8 (1.3)			
Oceania	6 (1.0)		6 (1.0)			
Southern Africa	5 (0.8)		5 (0.8)			
Eastern Africa	3 (0.5)		3 (0.5)			
Central Africa	2 (0.3)		2 (0.3)			
Central America	2 (0.3)		1 (0.2)	1 (0.2)		
Caribbean	1 (0.2)		1 (0.2)			
Central Asia	1 (0.2)		1 (0.2)			
**Country income level**						
High-income economies	309 (48.7)	4 (0.6)	281 (44.3)	26 (4.1)	2 (0.3)	
Upper-middle-income economies	181 (28.6)	2 (0.3)	156 (24.6)	22 (3.5)	2 (0.3)	
Lower-middle income economies	111 (17.5)	2 (0.3)	91 (14.4)	17 (2.7)	1 (0.2)	2 (0.3)
Low-income economies	15 (2.4)		15 (2.4)			
High-income countries	13 (2.1)		12 (1.9)	1 (0.2)		
Unclear	5 (0.8)		4 (0.6)	1 (0.2)		
**Age range (Years)**						
Adults: over 18 years	153 (24.1)	1 (12.5)	133 (23.8)	17 (25.4)	2 (40.0)	1 (50.0)
All ages	114 (18.0)	2 (25.0)	104 (18.6)	10 (14.9)		
Children: less 18 years	1 (0.2)		1 (0.2)			
Unclear/ Not reported	366 (57.7)	5 (62.5)	321 (57.4)	40 (59.7)	3 (60.0)	1 (50.0)
**Type of dialysis**						
Hemodialysis	582 (91.8)	5 (62.5)	514 (91.9)	61 (91.0)	5 (100)	2 (100)
Hemodialysis; Peritoneal dialysis	45 (7.1)	1 (12.5)	39 (7.0)	6 (9.0)		
Peritoneal dialysis	6 (1.0)	2 (25.0)	5 (0.9)			
Pre-Dialysis	1 (0.2)		1 (0.2)			
**HCV_diagnostic_method**						
Indirect ELISA	422 (66.6)	3 (37.5)	421 (75.3)			
Classical RT-PCR	52 (8.2)	1 (12.5)		52 (77.6)		
Chemiluminescent immunoassay	25 (3.9)		24 (4.3)		1 (20.0)	
Microparticle enzyme immunoassay	15 (2.4)		15 (2.7)			
Real-time RT-PCR	11 (1.7)			11 (16.4)		
Enzyme immunoassay (EIA)	8 (1.3)	2 (25.0)	7 (1.3)		1 (20.0)	
Rapid Diagnostic test	8 (1.3)		8 (1.4)			
Immunoblot Assay	4 (0.6)		4 (0.7)			

Immunochromatographic test	4 (0.6)		4 (0.7)			
Direct ELISA	3 (0.5)				3 (60.0)	
Direct ELISA, Indirect ELISA	2 (0.3)					2 (100)
Particle Agglutination	2 (0.3)		2 (0.4)			
Radioimmunoassay (RIA)	2 (0.3)		2 (0.4)			
Passive Hemagglutination assay (PHA)	1 (0.2)		1 (0.2)			
Transcription-Mediated Amplification (TMA)	1 (0.2)			1 (1.5)		
Unclear/ Not reported	74 (11.7)	2 (25.0)	71 (12.7)	3 (4.5)		
**Target**						
Anti-HCV	561 (88.5)	7 (87.5)	559 (100)			
Viral RNA	67 (10.6)	1 (12.5)		67 (100)		
Viral antigen	4 (0.6)				5 (100)	
Anti-HCV, Core Ag	2 (0.3)					2 (100)
**Sample types**						
Blood	548 (86.4)	7 (87.5)	480 (85.9)	61 (91.0)	4 (80.0)	2 (100)
Unclear/ Not reported	86 (13.6)	1 (12.5)	79 (14.1)	6 (9.0)	1 (20.0)	
**Risk of bias**						
Moderate risk of bias	418 (65.9)	2 (25.0)	369 (66.0)	46 (68.7)	3 (60.0)	
Low risk of bias	216 (34.1)	6 (75.0)	190 (34.0)	21 (31.3)	2 (40.0)	2 (100)
