# Supplementary material for: Global epidemiology of hepatitis C virus in dialysis patients: A systematic review and meta-analysis
Source: PLoS One. 2024 Feb 8;19(2):e0284169. doi: 10.1371/journal.pone.0284169 (PMC10852299; doi:10.1371/journal.pone.0284169)
Supplement: S6 Table — (PDF) [file pone.0284169.s007.pdf]

S6 Table: Individual characteristics of included studies

ID	Title	Author	Year of publication	Study Design	Sampling	Setting	Number of sites	Rural/Urban	HCV treatment	Timing of samples collection	Countries	Study period	Mean or Median age (Years)	Age range (Years)	Proportion of male participant	Type of dialysis	HCV_diagnostic_method	Target	Sample types
1	Molecular evidence of nosocomial transmission of hepatitis C virus in a haemodialysis unit.	Abacioglu	2000	Cross-sectional	Non probabilistic	Hospital-based	Monocenter	Urban	Unclear/ Not reported	Prospectively	Turkey	Unclear/ Not reported	31	All ages	69.7	Hemodialysis	Classical RT-PCR	Viral RNA	Blood
2	Effectiveness of direct-acting antivirals in Hepatitis C virus infection in haemodialysis patients.	Abad	2017	Cross-sectional	Non probabilistic	Hospital-based	Multicenter	Urban	Unclear/ Not reported	Retrospectively	Spain	Unclear/ Not reported	Unclear/ Not reported	Unclear/ Not reported	72.4	Hemodialysis	Chemiluminescent immunoassay	Anti-HCV	Blood
3	Prevalence of hepatitis C virus antibodies in hospital personnel.	Abb	1991	Cross-sectional	Non probabilistic	Hospital-based	Monocenter	Urban	Unclear/ Not reported	Prospectively	Germany	Unclear/ Not reported	Unclear/ Not reported	Adults: over 18 years	Unclear/ Not reported	Hemodialysis	Indirect ELISA	Anti-HCV	Blood
4	Hepatitis C virus infection in hemodialysis patients in qatar.	Abboud	1995	Cross-sectional	Non probabilistic	Hospital-based	Monocenter	Urban	Unclear/ Not reported	Prospectively	Qatar	Unclear/ Not reported	Unclear/ Not reported	Unclear/ Not reported	Unclear/ Not reported	Hemodialysis	Indirect ELISA	Anti-HCV	Blood
5	Prevalence of hepatitis c virus in haemodialysis patients, in sudan.	Abd Alrahman	2015	Cross-sectional	Non probabilistic	Hospital-based	Monocenter	Unclear/ Not reported	Unclear/ Not reported	Prospectively	Sudan	Jan/2010-Apr/2010	Unclear/ Not reported	All ages	Unclear/ Not reported	Hemodialysis	Indirect ELISA	Anti-HCV	Blood
6	Hepatitis C viral prevalence and seroconversion in Moroccan hemodialysis units: eight year follow up	Abdelaali	2013	Cross-sectional	Non probabilistic	Hospital-based	Multicenter	Urban	Unclear/ Not reported	Prospectively	Morocco	2002-2013	51.2	Unclear/ Not reported	61.3	Hemodialysis	Classical RT-PCR	Viral RNA	Blood
7	Detection of anti-hepatitis C-virus antibodies and hepatitis C-virus RNA in Lebanese hemodialysis patients.	Abdelnour	1997	Cross-sectional	Non probabilistic	Hospital-based	Multicenter	Urban	Unclear/ Not reported	Prospectively	United States of America	Unclear/ Not reported	Unclear/ Not reported	Unclear/ Not reported	Unclear/ Not reported	Hemodialysis	Indirect ELISA	Anti-HCV	Blood
8	Epidemiological and clinical aspects of hepatitis C virus infection in the Russian Republic of Daghestan.	Abdourakhmanov	1998	Cross-sectional	Non probabilistic	Hospital-based	Multicenter	Unclear/ Not reported	Unclear/ Not reported	Prospectively	Russian Federation	Apr/1994-Jun/1996	Unclear/ Not reported	Unclear/ Not reported	Unclear/ Not reported	Hemodialysis	Indirect ELISA	Anti-HCV	Blood
9	Hepatitis C virus genotypes and hepatitis G virus in hemodialysis patients from Syria: identification of two novel hepatitis C virus subtypes.	Abdulkarim	2012	Cross-sectional	Non probabilistic	Hospital-based	Multicenter	Unclear/ Not reported	Unclear/ Not reported	Prospectively	Syria	Unclear/ Not reported	Unclear/ Not reported	Unclear/ Not reported	Unclear/ Not reported	Hemodialysis	Unclear/ Not reported	Anti-HCV	Unclear/ Not reported
10	Current Status of Hepatitis C Virus-Infected Maintenance Hemodialysis Patients in Japan.	Abe	2018	Cross-sectional	Non probabilistic	Hospital-based	Multicenter	Urban	Antiviral naïve	Retrospectively	Japan	2016	Unclear/ Not reported	Unclear/ Not reported	Unclear/ Not reported	Hemodialysis	Unclear/ Not reported	Anti-HCV	Unclear/ Not reported
11	Incidence and prevalence of hepatitis B and hepatitis C viruses in hemodialysis patients in Lebanon.	Abou Rached	2016	Cross-sectional	Non probabilistic	Hospital-based	Multicenter	Unclear/ Not reported	Unclear/ Not reported	Retrospectively	United States of America	Oct/2010-Jul/2012	Unclear/ Not reported	Unclear/ Not reported	Unclear/ Not reported	Hemodialysis	Unclear/ Not reported	Anti-HCV	Unclear/ Not reported
12	Association of conjunctival and corneal calcification with vascular calcification among hepatitis-C-seropositive hemodialysis patients.	AbouSeif	2016	Cross-sectional	Non probabilistic	Hospital-based	Monocenter	Urban	Unclear/ Not reported	Prospectively	Egypt	Unclear/ Not reported	44.5	Unclear/ Not reported	85	Hemodialysis	Indirect ELISA	Anti-HCV	Blood
13	The effect of chemical and heat disinfection of the hemodialysis machines on the spread of hepatitis C virus infection: a prospective study.	Abu-Aisha	1995	Cross-sectional	Non probabilistic	Hospital-based	Monocenter	Urban	Unclear/ Not reported	Prospectively	Saudi Arabia	1992	Unclear/ Not reported	Unclear/ Not reported	Unclear/ Not reported	Hemodialysis; Peritoneal dialysis	Indirect ELISA	Anti-HCV	Blood
14	Correlation of hepatitis C antibody levels in gingival crevicular fluid and saliva of hepatitis C seropositive hemodialysis patients.	Açıkgöz	2009	Cross-sectional	Non probabilistic	Hospital-based	Monocenter	Urban	Unclear/ Not reported	Prospectively	Turkey	Unclear/ Not reported	Unclear/ Not reported	Unclear/ Not reported	Unclear/ Not reported	Hemodialysis	Indirect ELISA	Anti-HCV	Blood
15	Quality of life in hemodialysis patients: hepatitis C virus infection makes sense.	Afsar	2009	Cross-sectional	Non probabilistic	Hospital-based	Monocenter	Rural	Unclear/ Not reported	Prospectively	Turkey	Unclear/ Not reported	49.9	Unclear/ Not reported	Unclear/ Not reported	Hemodialysis	Microparticle enzyme immunoassay	Anti-HCV	Blood
16	Hepatitis C virus infection during haemodialysis in India.	Agarwal	1999	Cross-sectional	Non probabilistic	Hospital-based	Multicenter	Unclear/ Not reported	Unclear/ Not reported	Prospectively	India	Unclear/ Not reported	Unclear/ Not reported	Unclear/ Not reported	Unclear/ Not reported	Hemodialysis	Indirect ELISA	Anti-HCV	Blood
17	HCV infection during renal replacement therapy: should we dialyze all HCV-	Agarwal	1998	Cross-sectional	Non probabilistic	Hospital-based	Multicenter	Unclear/ Not reported	Unclear/ Not reported	Prospectively	India	Unclear/ Not reported	34.7	Unclear/ Not reported	78	Hemodialysis	Indirect ELISA	Anti-HCV	Blood

	positive patients on dedicated machines?																		
18	Hepatitis C virus infection in haemodialysis: the 'no-isolation' policy should not be generalized	Agarwal	2009	Cross-sectional	Non probabilistic	Hospital-based	Monocenter	Urban	Unclear/ Not reported	Prospectively	India	Mar/2003-Feb/2006	42.47	Unclear/ Not reported	70.4	Hemodialysis	Indirect ELISA	Anti-HCV	Blood
19	Prevalence of antibodies against the hepatitis C virus among voluntary blood donors at a makkah hospital.	Ahmad	1995	Cross-sectional	Non probabilistic	Hospital-based	Multicenter	Unclear/ Not reported	Unclear/ Not reported	Prospectively	Saudi Arabia	Unclear/ Not reported	Unclear/ Not reported	Adults: over 18 years	Unclear/ Not reported	Hemodialysis	Indirect ELISA	Anti-HCV	Blood
20	Assessing the prevalence of HBV and HCV infections in children under going hemodialysis and the related risk factors in a children's Medical Center: PS132.	Ahmadi	2017	Cross-sectional	Non probabilistic	Hospital-based	Monocenter	Urban	Antiviral naïve	Retrospectively	Iran	1991-2009	8.8	Unclear/ Not reported	49.6	Hemodialysis	Unclear/ Not reported	Anti-HCV	Unclear/ Not reported
21	Prevalence of hepatitis C virus in maintenance haemodialysis patients - A prospective study.	Ahmed	2003	Cross-sectional	Non probabilistic	Hospital-based	Monocenter	Unclear/ Not reported	Unclear/ Not reported	Prospectively	Bangladesh	Unclear/ Not reported	Unclear/ Not reported	Adults: over 18 years	64.1	Hemodialysis	Radioimmunoassay (RIA)	Anti-HCV	Unclear/ Not reported
22	[Hepatitis C virus infection in hemodialysis patients in General Hospital Gracanica].	Ahmetagić	2016	Cross-sectional	Non probabilistic	Hospital-based	Monocenter	Urban	Unclear/ Not reported	Prospectively	Serbia	2000	Unclear/ Not reported	Unclear/ Not reported	Unclear/ Not reported	Hemodialysis	Unclear/ Not reported	Anti-HCV	Unclear/ Not reported
23	Hepatitis C infection in risk groups.	Ahmetagić	2006	Cross-sectional	Non probabilistic	Hospital-based	Multicenter	Urban	Unclear/ Not reported	Prospectively	Bosnia and Herzegovina	2003-2005	Unclear/ Not reported	Unclear/ Not reported	Unclear/ Not reported	Hemodialysis	Indirect ELISA	Anti-HCV	Blood
24	Seroprevalence and risk factors for hepatitis C.	Akhmouch	2009	Cross-sectional	Non probabilistic	Hospital-based	Multicenter	Urban	Antiviral naïve	Prospectively	Morocco	Unclear/ Not reported	Unclear/ Not reported	Unclear/ Not reported	Unclear/ Not reported	Hemodialysis	Indirect ELISA	Anti-HCV	Blood
25	Prevalence of anti-HCV among haemodialysis patients in Turkey: a multicentre study.	Akpolat	1995	Cross-sectional	Non probabilistic	Hospital-based	Multicenter	Unclear/ Not reported	Unclear/ Not reported	Prospectively	Turkey	Nov/1993-Mar/1994	41.7	All ages	55.1	Hemodialysis	Indirect ELISA	Anti-HCV	Blood
26	Hepatitis C virus infection in hemodialysis patients: comparison of two new hepatitis C antibody assays with a second-generation assay.	al Meshari	1994	Cross-sectional	Non probabilistic	Hospital-based	Monocenter	Urban	Unclear/ Not reported	Prospectively	Saudi Arabia	Unclear/ Not reported	38	All ages	41.8	Hemodialysis	Classical RT-PCR	Viral RNA	Blood
27	Seropositivity to hepatitis C virus in Saudi haemodialysis patients.	al Nasser	1992	Cross-sectional	Non probabilistic	Hospital-based	Monocenter	Urban	Unclear/ Not reported	Prospectively	Saudi Arabia	Unclear/ Not reported	Unclear/ Not reported	Unclear/ Not reported	60.1	Hemodialysis	Indirect ELISA	Anti-HCV	Blood
28	The prevalence of hepatitis C virus antibodies among hemodialysis patients in jeddah area, saudi arabia.	Al Shohaib	1995	Cross-sectional	Non probabilistic	Hospital-based	Monocenter	Urban	Unclear/ Not reported	Prospectively	Saudi Arabia	1992	Unclear/ Not reported	All ages	76.2	Hemodialysis	Indirect ELISA	Anti-HCV	Blood
29	Anti-HCV positive hemodialysis patients: clinical, biochemical, histologic and virologic study and a proposed management scheme.	Al Traif	2000	Cross-sectional	Non probabilistic	Hospital-based	Monocenter	Urban	Unclear/ Not reported	Prospectively	Saudi Arabia	Unclear/ Not reported	Unclear/ Not reported	All ages	Unclear/ Not reported	Hemodialysis	Indirect ELISA	Anti-HCV	Blood
30	Hepatitis B and C prevalence among hemodialysis patients in the West Bank hospitals, Palestine.	Al Zabadi	2016	Cross-sectional	Non probabilistic	Hospital-based	Multicenter	Urban/Rural	Unclear/ Not reported	Prospectively	Palestine	2014	Unclear/ Not reported	Unclear/ Not reported	Unclear/ Not reported	Hemodialysis	Unclear/ Not reported	Anti-HCV	Unclear/ Not reported
31	Hepatitis B and C infection in haemodialysis patients in Libya: prevalence, incidence and risk factors.	Alashek	2012	Cross-sectional	Non probabilistic	Hospital-based	Multicenter	Urban/Rural	Unclear/ Not reported	Prospectively	Libya	May/2009-Oct/2010	49	Adults: over 18 years	58	Hemodialysis	Indirect ELISA	Anti-HCV	Blood
32	Prevalence of hepatitis C virus infection and related risk factors among Iranian haemodialysis patients.	Alavian	2003	Cross-sectional	Probabilistic	Hospital-based	Multicenter	Urban/Rural	Unclear/ Not reported	Prospectively	Iran	Unclear/ Not reported	50.4	Unclear/ Not reported	56.3	Hemodialysis	Indirect ELISA	Anti-HCV	Blood
33	[Spanish extrahospitalary hemodialysis centers survey].	Albalate	2007	Cross-sectional	Non probabilistic	Hospital-based	Monocenter	Urban	Unclear/ Not reported	Prospectively	Spain	Unclear/ Not reported	Unclear/ Not reported	Unclear/ Not reported	Unclear/ Not reported	Hemodialysis	Unclear/ Not reported	Anti-HCV	Unclear/ Not reported
34	Prevalence and risk factors of hepatitis C virus infection in hemodialysis patients from one center in Recife, Brazil.	Albuquerque	2005	Cross-sectional	Non probabilistic	Hospital-based	Monocenter	Urban	Unclear/ Not reported	Prospectively	Brazil	2002	46	All ages	58.8	Hemodialysis	Indirect ELISA	Anti-HCV	Blood
35	Prevalence of antibodies to hepatitis C virus among Omani	al-Dhahry	1993	Cross-sectional	Non probabilistic	Hospital-based	Monocenter	Urban	Unclear/ Not reported	Prospectively	Saudi Arabia	Unclear/ Not reported	42	All ages	45.1	Hemodialysis	Indirect ELISA	Anti-HCV	Blood

	patients with renal disease.																		
36	Hepatitis C virus infection in chronic haemodialysis patients, a clinicopathologic study.	Alfurayh	1992	Cross-sectional	Non probabilistic	Hospital-based	Monocenter	Rural	Unclear/ Not reported	Prospectively	Saudi Arabia	May/1989-Jul/1990	42.2	Adults: over 18 years	46.1	Hemodialysis	Indirect ELISA	Anti-HCV	Blood
37	Hepatitis C Virus Sero-status in Hemodialysis Patients Returning from Holiday: Another Risk Factor for HCV Transmission.	Al-Ghamdi	2001	Cross-sectional	Non probabilistic	Hospital-based	Multicenter	Unclear/ Not reported	Unclear/ Not reported	Prospectively	Saudi Arabia	1992-1995	Unclear/ Not reported	All ages	42.8	Hemodialysis	Indirect ELISA	Anti-HCV	Blood
38	Prevalence and risk factors of hepatitis B and C viruses among haemodialysis patients: a multicentric study.	Ali	2019	Cross-sectional	Non probabilistic	Hospital-based	Multicenter	Urban	Unclear/ Not reported	Prospectively	Pakistan	Jul/2013-Feb/2014	Unclear/ Not reported	Unclear/ Not reported	58.7	Hemodialysis	Classical RT-PCR	Viral RNA	Blood
39	Hepatitis B Virus and Hepatitis C Virus in Hemodialysis Patients: A Prevalence Study from Dialysis Centers in El-Beyda and Almarj-Libya	Ali	2017	Cross-sectional	Non probabilistic	Hospital-based	Multicenter	Urban	Unclear/ Not reported	Prospectively	Libya	Jan/2013-Dec/2016	Unclear/ Not reported	Adults: over 18 years	55.8	Hemodialysis	Indirect ELISA	Anti-HCV	Blood
40	Hepatitis C virus (HCV) infection in hemodialysis patients in the south of Jordan.	Al-Jamal	2009	Cross-sectional	Non probabilistic	Hospital-based	Multicenter	Rural	Unclear/ Not reported	Prospectively	Jordan	Jan/2007-Apr/2008	52.9	All ages	52	Hemodialysis	Indirect ELISA	Anti-HCV	Blood
41	Hepatitis B and C Viral Infections among Dialysis Patients and Related Factors of Dialysis Centres in Saudi Arabia.	Aljarallah	2022	Cross-sectional	Non probabilistic	Hospital-based	Multicenter	Urban	Antiviral naïve	Retrospectively	Saudi Arabia	Aug/2017-Aug/2018	Unclear/ Not reported	Unclear/ Not reported	Unclear/ Not reported	Hemodialysis	Unclear/ Not reported	Anti-HCV	Unclear/ Not reported
42	Hepatitis C virus infection among patients on hemodialysis in jeddah: a single center experience.	Al-Jiffri	2003	Cross-sectional	Probabilistic	Hospital-based	Monocenter	Urban	Antiviral naïve	Retrospectively	Saudi Arabia	Unclear/ Not reported	Unclear/ Not reported	Unclear/ Not reported	Unclear/ Not reported	Hemodialysis	Unclear/ Not reported	Anti-HCV	Unclear/ Not reported
43	Seroprevalence of hepatitis C virus and hepatitis B virus among dialysis patients in Bahrain and Saudi Arabia.	Almawi	2004	Cross-sectional	Non probabilistic	Hospital-based	Multicenter	Urban	Unclear/ Not reported	Prospectively	Bahrain; Saudi Arabia	Unclear/ Not reported	Unclear/ Not reported	Unclear/ Not reported	Unclear/ Not reported	Hemodialysis	Classical RT-PCR	Viral RNA	Blood
44	Monitoring hepatitis C infection in a major Swedish nephrology unit and molecular resolution of a new case of nosocomial transmission.	Almroth	2010	Cross-sectional	Non probabilistic	Hospital-based	Monocenter	Urban	Antiviral naïve/ On antiviral	Retrospectively	Sweden	1997-2007	Unclear/ Not reported	Unclear/ Not reported	Unclear/ Not reported	Hemodialysis; Peritoneal dialysis	Immunoblot Assay	Anti-HCV	Blood
45	Detection and prevention of hepatitis C in dialysis patients and renal transplant recipients. A long-term follow up (1989-January 1997).	Almroth	2002	Cross-sectional	Non probabilistic	Hospital-based	Monocenter	Urban	Unclear/ Not reported	Retrospectively	Sweden	1990-1991	Unclear/ Not reported	Unclear/ Not reported	Unclear/ Not reported	Hemodialysis	Indirect ELISA	Anti-HCV	Blood
46	Seropositivity to hepatitis C virus (HCV) in Saudi children with chronic renal failure maintained on haemodialysis.	al-Mugeiren	1992	Cross-sectional	Non probabilistic	Hospital-based	Multicenter	Urban	Unclear/ Not reported	Prospectively	Saudi Arabia	Unclear/ Not reported	7.7	Adults: over 18 years	60	Hemodialysis	Indirect ELISA	Anti-HCV	Blood
47	Hepatitis C virus infection in two groups of paediatric patients: one maintained on haemodialysis and the other on continuous ambulatory peritoneal dialysis.	al-Mugeiren	1996	Cross-sectional	Non probabilistic	Hospital-based	Multicenter	Urban	Unclear/ Not reported	Prospectively	Saudi Arabia	Unclear/ Not reported	Unclear/ Not reported	Unclear/ Not reported	Unclear/ Not reported	Hemodialysis	Indirect ELISA	Anti-HCV	Blood
48	Hepatitis C virus infection among hemodialysis patients in the eastern region of saudi arabia.	Al-Muhanna	1995	Cross-sectional	Non probabilistic	Hospital-based	Monocenter	Urban	Unclear/ Not reported	Prospectively	Saudi Arabia	Unclear/ Not reported	Unclear/ Not reported	Adults: over 18 years	Unclear/ Not reported	Hemodialysis	Indirect ELISA	Anti-HCV	Blood
49	Prevalence rate of hepatitis c virus (HCV) and hepatitis b virus (HBV) infection in iraqi patients on hemodialysis: Cross sectional study.	Al-Muramdy	2020	Cross-sectional	Non probabilistic	Hospital-based	Monocenter	Urban	Unclear/ Not reported	Prospectively	Iraq	2019	47.65	All ages	51.9	Hemodialysis	Indirect ELISA	Anti-HCV	Blood
50	Antibodies to hepatitis C virus in patients on haemodialysis.	Alonso	1991	Cross-sectional	Non probabilistic	Hospital-based	Multicenter	Urban	Unclear/ Not reported	Prospectively	Spain	Unclear/ Not reported	Unclear/ Not reported	Unclear/ Not reported	Unclear/ Not reported	Hemodialysis	Indirect ELISA	Anti-HCV	Blood
51	Effect of hepatitis C virus on hemoglobin and hematocrit	Alsaran	2009	Cross-sectional	Non probabilistic	Hospital-based	Monocenter	Urban	Unclear/ Not reported	Prospectively	Saudi Arabia	Unclear/ Not reported	52.2	Unclear/ Not reported	53.0	Hemodialysis	Classical RT-PCR	Viral RNA	Blood

	levels in saudi hemodialysis patients.																		
52	Prevalence of blood borne viruses in the dialysis unit, mubarak Al-Kabeer hospital, kuwait.	Altawalah	2015	Cross-sectional	Non probabilistic	Hospital-based	Multicenter	Urban	Antiviral naïve	Prospectively	Kuwait	2012	Unclear/ Not reported	Unclear/ Not reported	57	Hemodialysis	Chemiluminescent immunoassay	Anti-HCV	Blood
53	Human herpesvirus 6 infection in hemodialysis and peritoneal dialysis patients.	Altay	2011	Cross-sectional	Non probabilistic	Hospital-based	Multicenter	Unclear/ Not reported	Unclear/ Not reported	Prospectively	Turkey	Unclear/ Not reported	Unclear/ Not reported	Unclear/ Not reported	Unclear/ Not reported	Hemodialysis; Peritoneal dialysis	Indirect ELISA	Anti-HCV	Blood
54	[Investigation of hemodialysis patients in terms of the presence of occult hepatitis B].	Altindiş	2007	Cross-sectional	Non probabilistic	Hospital-based	Multicenter	Urban	Unclear/ Not reported	Prospectively	Turkey	Unclear/ Not reported	41.3	Unclear/ Not reported	63.4	Hemodialysis	Indirect ELISA	Anti-HCV	Blood
55	Hepatitis C virus in populations at risk for infection.	Alve-Castillo	2007	Cross-sectional	Non probabilistic	Hospital-based	Monocenter	Urban	Antiviral naïve	Prospectively	Venezuela	Unclear/ Not reported	Unclear/ Not reported	Unclear/ Not reported	Unclear/ Not reported	Hemodialysis	Indirect ELISA	Anti-HCV	Unclear/ Not reported
56	Architect hepatitis c virus (HCV) core antigen test: A HCV rna screening alternative in end-stage renal disease (ESRD) and hemodialysis patients?	Alves	2018	Cross-sectional	Non probabilistic	Hospital-based	Monocenter	Urban	Antiviral naïve	Retrospectively	Portugal	Unclear/ Not reported	72.7	Unclear/ Not reported	67.5	Hemodialysis	Chemiluminescent immunoassay	Anti-HCV	Unclear/ Not reported
57	Liver disease in dialysis patients with antibodies to hepatitis C virus.	al-Wakeel	1996	Cross-sectional	Non probabilistic	Hospital-based	Multicenter	Unclear/ Not reported	Unclear/ Not reported	Prospectively	Saudi Arabia	1990	Unclear/ Not reported	Unclear/ Not reported	Unclear/ Not reported	Hemodialysis; Peritoneal dialysis	Indirect ELISA	Anti-HCV	Blood
58	Prevalence and associated factors of hepatitis C virus infection among renal disease patients on maintenance hemodialysis in three health centers in Aden, Yemen: a cross sectional study.	Aman	2015	Cross-sectional	Non probabilistic	Hospital-based	Monocenter	Urban	Unclear/ Not reported	Prospectively	Yemen	2000-2013	47.0	Adults: over 18 years	69.9	Hemodialysis	Unclear/ Not reported	Anti-HCV	Unclear/ Not reported
59	Hepatitis C virus infection in a Moroccan hemodialysis unit: Prevalence and risk factors [1].	Amar	2005	Cross-sectional	Non probabilistic	Hospital-based	Monocenter	Urban	Unclear/ Not reported	Retrospectively	Morocco	Unclear/ Not reported	43.3	All ages	45.9	Hemodialysis	Indirect ELISA	Anti-HCV	Blood
60	Hepatitis C in Lithuania: incidence, prevalence, risk factors and viral genotypes.	Ambrozaitis	1995	Cross-sectional	Non probabilistic	Hospital-based	Monocenter	Urban	Unclear/ Not reported	Prospectively	Lithuania	Nov/1992-Apr/1993	Unclear/ Not reported	Unclear/ Not reported	Unclear/ Not reported	Hemodialysis	Indirect ELISA	Anti-HCV	Blood
61	[Epidemiology of chronic hepatitis B and C among dialysis patients in Switzerland].	Ambühl	2000	Cross-sectional	Probabilistic	Hospital-based	Nationally representative	Urban/Rural	Unclear/ Not reported	Prospectively	Switzerland	Unclear/ Not reported	Unclear/ Not reported	Unclear/ Not reported	Unclear/ Not reported	Hemodialysis; Peritoneal dialysis	Unclear/ Not reported	Anti-HCV	Unclear/ Not reported
62	Hepatitis C virus status in hemodialysis patients in Menoufia Government, Egypt, five years apart: Do we have any improvement?	Amin Elzorkany	2017	Cross-sectional	Non probabilistic	Hospital-based	Multicenter	Urban	Unclear/ Not reported	Prospectively	Egypt	1991; 1996	Unclear/ Not reported	Unclear/ Not reported	Unclear/ Not reported	Hemodialysis	Indirect ELISA	Anti-HCV	Blood
63	Seroprevalence of hepatitis C and risk factors in haemodialysis patients in Guilan, Islamic Republic of Iran.	Amiri	2005	Cross-sectional	Non probabilistic	Hospital-based	Multicenter	Urban	Unclear/ Not reported	Prospectively	Iran	Mar/2001-Apr/2001	52.2	All ages	52.3	Hemodialysis	Indirect ELISA	Anti-HCV	Blood
64	Association of anti-HCV sero-prevalence with blood transfusion and practice of haemodialysis from multiple centres in patients on maintenance haemodialysis.	Amjad	2020	Cross-sectional	Non probabilistic	Hospital-based	Monocenter	Urban	Unclear/ Not reported	Prospectively	Pakistan	Mar/2019-May/2019	50	Adults: over 18 years	64.6	Hemodialysis	Chemiluminescent immunoassay	Anti-HCV	Blood
65	s	Amorim	2010	Cross-sectional	Non probabilistic	Hospital-based	Multicenter	Unclear/ Not reported	Unclear/ Not reported	Prospectively	Brazil	Jan/2002-May/2002	Unclear/ Not reported	Unclear/ Not reported	Unclear/ Not reported	Hemodialysis	Indirect ELISA	Anti-HCV	Blood
66	Prevalence of hepatitis C virus infection in thalassemia and haemodialysis patients in north Iran-Rasht.	Ansar	2002	Cross-sectional	Non probabilistic	Hospital-based	Monocenter	Unclear/ Not reported	Unclear/ Not reported	Prospectively	Iran	Mar/1997-Sep/1998	Unclear/ Not reported	Unclear/ Not reported	Unclear/ Not reported	Hemodialysis	Indirect ELISA	Anti-HCV	Blood
67	Recombinant γ-interferon as adjuvant to hepatitis B vaccine in hemodialysis patients.	Antonio Quiroga	1990	Clinical Trial (Baseline data)	Probabilistic	Hospital-based	Monocenter	Unclear/ Not reported	Unclear/ Not reported	Prospectively	Spain	Jul/1987-Dec/1987	Unclear/ Not reported	Adults: over 18 years	56.7	Hemodialysis	Indirect ELISA	Anti-HCV	Blood
68	Prevalence of Hepatitis B and Hepatitis C Infection	Anwar	2016	Cross-sectional	Non probabilistic	Hospital-based	Monocenter	Urban	Unclear/ Not reported	Prospectively	Pakistan	Unclear/ Not reported	Unclear/ Not reported	Unclear/ Not reported	Unclear/ Not reported	Hemodialysis; Peritoneal dialysis	Immunochromatographic test	Anti-HCV	Blood

	among Patients Undergoing Dialysis.																		
69	HBV-DNA in hemodialysis patients infected by HCV.	Arababadi	2009	Cross-sectional	Non probabilistic	Hospital-based	Monocenter	Urban	Unclear/ Not reported	Prospectively	Iran	Unclear/ Not reported	Unclear/ Not reported	Adults: over 18 years	42.2	Hemodialysis	Classical RT-PCR	Viral RNA	Blood
70	[Nosocomial transmission of the hepatitis C virus in hemodialysis: monitors, personnel, or both?].	Arenas	2001	Cross-sectional	Non probabilistic	Hospital-based	Monocenter	Unclear/ Not reported	Unclear/ Not reported	Prospectively	Spain	Unclear/ Not reported	Unclear/ Not reported	Unclear/ Not reported	Unclear/ Not reported	Hemodialysis	Unclear/ Not reported	Anti-HCV	Blood
71	Peculiarities of the Clinical Course of Chronic Viral Hepatitis C in the Background of End-Stage Chronic Renal Insufficiency.	Aripkhodjayeva	2020	Cross-sectional	Non probabilistic	Hospital-based	Multicenter	Unclear/ Not reported	Unclear/ Not reported	Prospectively	Uzbekistan	Unclear/ Not reported	Unclear/ Not reported	Adults: over 18 years	66.6	Hemodialysis	Unclear/ Not reported	Anti-HCV	Blood
72	Hemodialysis access surgery - is there an increased risk of acquiring hepatitis C virus compared to other elective vascular interventions?	Assadian	2008	Cross-sectional	Non probabilistic	Hospital-based	Monocenter	Unclear/ Not reported	Unclear/ Not reported	Retrospectively	Austria	Unclear/ Not reported	Unclear/ Not reported	Unclear/ Not reported	Unclear/ Not reported	Hemodialysis	Indirect ELISA	Anti-HCV	Blood
73	Prevalence of hepatitis C and B infection and HC V genotypes among hemodialysis patients in Khuzestan province, southwest Iran.	Assarehzadegan	2009	Cross-sectional	Non probabilistic	Hospital-based	Multicenter	Unclear/ Not reported	Unclear/ Not reported	Prospectively	Iran	Mar/2005-Aug/2006	37.3	All ages	63.1	Hemodialysis	Indirect ELISA	Anti-HCV	Blood
74	Effectiveness of universal precautions in limiting nosocomial transmission of hepatitis C virus in haemodialysis units.	Aucella	1996	Cross-sectional	Non probabilistic	Hospital-based	Multicenter	Unclear/ Not reported	Unclear/ Not reported	Prospectively	Italy	Unclear/ Not reported	Unclear/ Not reported	Unclear/ Not reported	Unclear/ Not reported	Hemodialysis	Indirect ELISA	Anti-HCV	Blood
75	Systematic monitor disinfection is effective in limiting HCV spread in hemodialysis.	Aucella	2000	Cohort (Baseline data)	Non probabilistic	Hospital-based	Multicenter	Unclear/ Not reported	Unclear/ Not reported	Prospectively	Italy	Unclear/ Not reported	58	Adults: over 18 years	53.3	Hemodialysis	Indirect ELISA	Anti-HCV	Blood
76	Hepatitis C virus infection in hemodialysis patients from Tunisia: national survey by serologic and molecular methods.	Ayed	2003	Cross-sectional	Probabilistic	Hospital-based	Nationally representative	Unclear/ Not reported	Unclear/ Not reported	Prospectively	Tunisia	Mar/2001-Jun/2001	Unclear/ Not reported	Unclear/ Not reported	Unclear/ Not reported	Hemodialysis; Peritoneal dialysis	Indirect ELISA	Anti-HCV	Blood
77	Prevalence and significance of antibodies to hepatitis C virus among Saudi haemodialysis patients.	Ayoola	1991	Cross-sectional	Non probabilistic	Hospital-based	Monocenter	Unclear/ Not reported	Unclear/ Not reported	Prospectively	Saudi Arabia	Unclear/ Not reported	44.3	Adults: over 18 years	44.5	Hemodialysis	Indirect ELISA	Anti-HCV	Blood
78	[Prevalence and risk factors of hepatitis C virus infection in chronic hemodialysis patients at the University Hospital of Point G, Bamako, Mali].	Baby	2011	Cross-sectional	Non probabilistic	Hospital-based	Monocenter	Unclear/ Not reported	Unclear/ Not reported	Prospectively	Mali	Nov/2008	42.2	Adults: over 18 years	60.6	Hemodialysis	Indirect ELISA	Anti-HCV	Blood
79	Hepatitis C virus antibodies in high-risk Saudi groups.	Bahakim	1991	Cross-sectional	Non probabilistic	Hospital-based	Monocenter	Urban	Antiviral naïve	Prospectively	Saudi Arabia	1990	Unclear/ Not reported	Unclear/ Not reported	73.8	Hemodialysis	Indirect ELISA	Anti-HCV	Blood
80	Seroprevalence of Anti HCV Ab in Healthy Voluntary BloodDonors and in High Risk Individuals.	Baheti	2000	Cross-sectional	Non probabilistic	Hospital-based	Monocenter	Unclear/ Not reported	Unclear/ Not reported	Prospectively	India	Unclear/ Not reported	Unclear/ Not reported	Unclear/ Not reported	Unclear/ Not reported	Hemodialysis	Indirect ELISA	Anti-HCV	Blood
81	Prevalence of occult hepatitis C infection in chronic hemodialysis and kidney transplant patients.	Baid-Agrawal	2014	Cross-sectional	Non probabilistic	Hospital-based	Multicenter	Urban	Unclear/ Not reported	Prospectively	Germany	May/2010-Mar/2011	66.1	Unclear/ Not reported	54	Hemodialysis	Chemiluminescent immunoassay	Anti-HCV	Blood
82	Hepatitis C risk factor for patients submitted to dialysis.	Baldessar	2007	Cross-sectional	Non probabilistic	Hospital-based	Monocenter	Urban	Unclear/ Not reported	Retrospectively	Brazil	Jan/2004-Dec/2004	Unclear/ Not reported	Unclear/ Not reported	Unclear/ Not reported	Hemodialysis	Microparticle enzyme immunoassay	Anti-HCV	Blood
83	Prevalence of HCV Infection Among Hemodialysis Patients in Lanzhou of Northwestern China.	Bao	2022	Cross-sectional	Probabilistic	Hospital-based	Multicenter	Urban	Antiviral naïve	Retrospectively	China	2018	Unclear/ Not reported	All ages	Unclear/ Not reported	Hemodialysis	Unclear/ Not reported	Anti-HCV	Unclear/ Not reported
84	Performance of point of care assays for hepatitis B and C viruses in chronic kidney disease patients.	Barbosa	2018	Cross-sectional	Non probabilistic	Hospital-based	Multicenter	Urban	Unclear/ Not reported	Prospectively	Brazil	Unclear/ Not reported	51.3	Adults: over 18 years	64	Hemodialysis	Indirect ELISA	Anti-HCV	Blood
85	Prevalence of hepatitis C virus in dialysis patients in Spain.	Barril	1995	Cross-sectional	Non probabilistic	Hospital-based	Multicenter	Urban	Unclear/ Not reported	Retrospectively	Spain	Unclear/ Not reported	Unclear/ Not reported	Unclear/ Not reported	Unclear/ Not reported	Hemodialysis; Peritoneal dialysis	Unclear/ Not reported	Anti-HCV	Unclear/ Not reported
86	Detection of HGV in serum and peripheral blood	Basaras	1999	Cross-sectional	Non probabilistic	Hospital-based	Monocenter	Urban	Unclear/ Not reported	Prospectively	Spain	Unclear/ Not reported	61·5	Adults: over 18 years	68.8	Hemodialysis	Indirect ELISA	Anti-HCV	Blood

	mononuclear cells of maintenance haemodialysis patients.																		
87	Prevalence of hepatitis C in patients with renal disease undergoing hemodialysis treatment.	Bastiani	2014	Cross-sectional	Non probabilistic	Hospital-based	Monocenter	Unclear/ Not reported	Unclear/ Not reported	Retrospectively	Brazil	Jan/2012-Dec/2012	60.5	All ages	57.6	Hemodialysis	Unclear/ Not reported	Anti-HCV	Unclear/ Not reported
88	Anti-ENA antibody profile in hepatitis C patients undergoing hemodialysis.	Batchoun	2011	Cross-sectional	Non probabilistic	Hospital-based	Multicenter	Urban	Unclear/ Not reported	Prospectively	Jordan	Unclear/ Not reported	44.2	Unclear/ Not reported	50	Hemodialysis	Indirect ELISA	Anti-HCV	Blood
89	Epidemiology and cost of haemodialysis in Jordan.	Batieha	2007	Cross-sectional	Non probabilistic	Hospital-based	Multicenter	Urban	Unclear/ Not reported	Retrospectively	Jordan	Sep.2003-Oct/2003	48.9	All ages	55.9	Hemodialysis	Unclear/ Not reported	Anti-HCV	Unclear/ Not reported
90	Hepatitis C virus infection in Jordanian haemodialysis units: serological diagnosis and genotyping.	Bdour	2002	Cross-sectional	Non probabilistic	Hospital-based	Multicenter	Urban	Unclear/ Not reported	Prospectively	Jordan	Unclear/ Not reported	Unclear/ Not reported	All ages	61.5	Hemodialysis	Indirect ELISA	Anti-HCV	Blood
91	HGV infection in dialysis patients.	Beccari	1996	Cross-sectional	Non probabilistic	Hospital-based	Monocenter	Urban	Antiviral naïve	Prospectively	Italy	Unclear/ Not reported	Unclear/ Not reported	Unclear/ Not reported	Unclear/ Not reported	Hemodialysis	Indirect ELISA	Anti-HCV	Blood
92	[High prevalence and incidence of hepatitis C virus infections among dialysis patients in the East-Centre of Tunisia].	Ben Othman	2004	Cross-sectional	Non probabilistic	Hospital-based	Multicenter	Urban	Unclear/ Not reported	Prospectively	Tunisia	Mar/2000-Dec/2002	51	All ages	61.6	Hemodialysis	Indirect ELISA	Anti-HCV	Blood
93	Virological markers profile in hemodialysis patients.	Benghanem	2009	Cross-sectional	Non probabilistic	Hospital-based	Monocenter	Urban	Unclear/ Not reported	Prospectively	Morocco	Unclear/ Not reported	Unclear/ Not reported	Unclear/ Not reported	Unclear/ Not reported	Hemodialysis	Indirect ELISA	Anti-HCV	Unclear/ Not reported
94	Prevalence of hepatitis C virus antibodies in hemodialysis patients in madinah Al munawarah.	Bernieh	1995	Cross-sectional	Non probabilistic	Hospital-based	Monocenter	Urban	Unclear/ Not reported	Prospectively	Saudi Arabia	Nov/1991	47.2	Unclear/ Not reported	44.7	Hemodialysis	Indirect ELISA	Anti-HCV	Blood
95	Prevalence of HCV antibodies in a uraemic population undergoing maintenance dialysis therapy and in the staff members of the dialysis unit.	Besso	1992	Cross-sectional	Non probabilistic	Hospital-based	Monocenter	Urban	Unclear/ Not reported	Prospectively	Italy	Jan/1990-Nov/1990	Unclear/ Not reported	Unclear/ Not reported	Unclear/ Not reported	Hemodialysis; Peritoneal dialysis	Indirect ELISA	Anti-HCV	Blood
96	Prevalence of Hepatitis B and C among HemodialysisPatients of Tripura, India.	Bhaumik	2012	Cross-sectional	Non probabilistic	Hospital-based	Multicenter	Urban	Unclear/ Not reported	Prospectively	India	Jan/2011-Jun/2011	48.2	Unclear/ Not reported	72.7	Hemodialysis	Indirect ELISA	Anti-HCV	Blood
97	The seroprevalence of anti-HCV in high-risk dialysis patients.	Bin Selm	2011	Cross-sectional	Non probabilistic	Hospital-based	Monocenter	Urban	Unclear/ Not reported	Prospectively	Yemen	Unclear/ Not reported	55.9	Adults: over 18 years	62.2	Hemodialysis	Indirect ELISA	Anti-HCV	Blood
98	Seroprevalence of Hepatitis B Virus and Hepatitis C Virus in Patients Undergoing Maintenance Hemodialysis.	Bin Shabbir	2022	Cross-sectional	Non probabilistic	Hospital-based	Monocenter	Urban	Antiviral naïve	Prospectively	Pakistan	Unclear/ Not reported	58.2	Unclear/ Not reported	72	Hemodialysis	Indirect ELISA	Anti-HCV	Blood
99	HCV viremia in hemodialysis patients: detection by a DNA enzyme immunoassay for amplified HCV sequences.	Boero	1995	Cross-sectional	Non probabilistic	Hospital-based	Monocenter	Urban	Unclear/ Not reported	Prospectively	Italy	Unclear/ Not reported	63	Adults: over 18 years	53.3	Hemodialysis	Indirect ELISA	Anti-HCV	Blood
100	Does infection by the hepatitis C virus decrease the response of immunization against the hepatitis B virus in individuals undergoing dialysis?	Bonfm Ferreira	2017	Cross-sectional	Non probabilistic	Hospital-based	Multicenter	Urban	Unclear/ Not reported	Prospectively	Brazil	2010-2013	46.1	All ages	54.8	Hemodialysis	Indirect ELISA	Anti-HCV	Blood
101	Occurrence of infectious diseases in dialysed patients.	Borzecki	2004	Cross-sectional	Non probabilistic	Hospital-based	Monocenter	Urban	Antiviral naïve	Prospectively	Poland	Unclear/ Not reported	Unclear/ Not reported	Unclear/ Not reported	Unclear/ Not reported	Hemodialysis	Unclear/ Not reported	Anti-HCV	Unclear/ Not reported
102	Screening for hepatitis B, C and HIV infection among patients on haemodialysis (cross sectional analysis among patients from two dialysis units in the period January to July 2005).	Bosevska	2009	Cross-sectional	Non probabilistic	Hospital-based	Multicenter	Urban	Unclear/ Not reported	Prospectively	Republic of Macedonia	Jan/2005-Jul/2005	54	Adults: over 18 years	Unclear/ Not reported	Hemodialysis	Indirect ELISA	Anti-HCV	Blood
103	[Prevalence of hepatitis C, hepatitis B and HIV infection among haemodialysis patients in Ibn-Rochd university hospital, Casablanca].	Boulaajaj	2005	Cross-sectional	Non probabilistic	Hospital-based	Monocenter	Urban	Unclear/ Not reported	Retrospectively	Morocco	1983-2002	43.1	All ages	49.5	Hemodialysis	Unclear/ Not reported	Anti-HCV	Unclear/ Not reported
104	Presence and significance of TT virus in Danish patients on	Boysen	2003	Cross-sectional	Non probabilistic	Hospital-based	Monocenter	Urban	Unclear/ Not reported	Prospectively	Denmark	Feb/2000-Apr/2000	58.0	Adults: over 18 years	65.7	Hemodialysis	Indirect ELISA	Anti-HCV	Blood

	maintenance hemodialysis.																		
105	Hepatitis C: reality of a renal unit.	Bracchi	1992	Cross-sectional	Non probabilistic	Hospital-based	Monocenter	Unclear/ Not reported	Unclear/ Not reported	Prospectively	Italy	Unclear/ Not reported	Unclear/ Not reported	Unclear/ Not reported	56.0	Hemodialysis	Indirect ELISA	Anti-HCV	Blood
106	Prevalence of hepatitis C infection and its risk factors in hemodialysis patients in tehran: preliminary report from "the effect of dialysis unit isolation on the incidence of hepatitis C in dialysis patients" project.	Broumand	2002	Cross-sectional	Non probabilistic	Hospital-based	Multicenter	Urban	Unclear/ Not reported	Prospectively	Iran	Unclear/ Not reported	45.4	All ages	58	Hemodialysis	Indirect ELISA	Anti-HCV	Blood
107	Antibodies against hepatitis C virus in hemodialysis patients in the central Italian region of Umbria: evaluation of some risk factors.	Brugnano	1992	Cross-sectional	Non probabilistic	Hospital-based	Monocenter	Unclear/ Not reported	Unclear/ Not reported	Prospectively	Italy	Jun/1990-Sep/1990	58.3	Adults: over 18 years	61.7	Hemodialysis; Peritoneal dialysis	Indirect ELISA	Anti-HCV	Blood
108	Seroepidemiology of HBV and HCV infection in Jakarta, Indonesia.	Budihusodo	1999	Cross-sectional	Non probabilistic	Hospital-based	Monocenter	Rural	Unclear/ Not reported	Prospectively	Indonesia	Jun/1990-Sep/1990	Unclear/ Not reported	Unclear/ Not reported	Unclear/ Not reported	Hemodialysis	Indirect ELISA	Anti-HCV	Blood
109	High prevalence of hepatitis C virus (HCV) RNA in dialysis patients: failure of commercially available antibody tests to identify a significant number of patients with HCV infection. Copenhagen Dialysis HCV Study Group.	Bukh	1993	Cross-sectional	Non probabilistic	Hospital-based	Monocenter	Unclear/ Not reported	Unclear/ Not reported	Prospectively	Denmark	Oct/1990-Mar/1991	Unclear/ Not reported	Unclear/ Not reported	Unclear/ Not reported	Hemodialysis	Indirect ELISA	Anti-HCV	Blood
110	Hepatitis C and hepatitis B virus infection in different hemodialysis units in Belo Horizonte, Minas Gerais, Brazil.	Busek	2002	Cross-sectional	Non probabilistic	Hospital-based	Multicenter	Urban	Unclear/ Not reported	Prospectively	Brazil	Mar/2000-Dec/2000	Unclear/ Not reported	Adults: over 18 years	56.3	Hemodialysis	Indirect ELISA	Anti-HCV	Blood
111	Treatment Outcomes for Patients Undergoing Hemodialysis with Chronic Hepatitis C on the Sofosbuvir and Daclatasvi	Butt	2019	Cross-sectional	Non probabilistic	Hospital-based	Multicenter	Urban	On antiviral	Prospectively	Pakistan	Jan/2017-Dec/2018	Unclear/ Not reported	Unclear/ Not reported	Unclear/ Not reported	Hemodialysis	Indirect ELISA	Anti-HCV	Blood
112	Transmission of anti-HCV within the household of haemodialysis patients.	Calabrese	1991	Cross-sectional	Non probabilistic	Hospital-based	Monocenter	Urban	Unclear/ Not reported	Prospectively	Italy	Unclear/ Not reported	Unclear/ Not reported	Adults: over 18 years	Unclear/ Not reported	Hemodialysis	Indirect ELISA	Anti-HCV	Blood
113	TT virus infection in haemodialysis patients.	Campo	2000	Cross-sectional	Non probabilistic	Hospital-based	Monocenter	Urban	Unclear/ Not reported	Prospectively	Italy	Unclear/ Not reported	62.1	Adults: over 18 years	57.6	Hemodialysis	Immunoblot Assay	Anti-HCV	Blood
114	Prevalence of antibodies against hepatitis C virus in a dialysis unit.	Cantù	1992	Cross-sectional	Non probabilistic	Hospital-based	Monocenter	Unclear/ Not reported	Unclear/ Not reported	Prospectively	Italy	Unclear/ Not reported	Unclear/ Not reported	Unclear/ Not reported	Unclear/ Not reported	Hemodialysis; Peritoneal dialysis	Indirect ELISA	Anti-HCV	Blood
115	HCV seroprevalence in dialysis patients, their relatives and medical staff.	Capşa	1991	Cross-sectional	Non probabilistic	Hospital-based	Monocenter	Unclear/ Not reported	Unclear/ Not reported	Prospectively	Romania	Unclear/ Not reported	Unclear/ Not reported	Unclear/ Not reported	Unclear/ Not reported	Hemodialysis	Unclear/ Not reported	Anti-HCV	Unclear/ Not reported
116	Undiagnosed hepatitis C virus infection in hemodialysis patients: value of HCV RNA and liver enzyme levels.	Caramelo	1996	Cross-sectional	Non probabilistic	Hospital-based	Monocenter	Rural	Unclear/ Not reported	Retrospectively	Spain	Unclear/ Not reported	57.3	Unclear/ Not reported	59.8	Hemodialysis	Indirect ELISA	Anti-HCV	Blood
117	Hepatitis C prevalence and risk factors in hemodialysis patients in Central Brazil: a survey by polymerase chain reaction and serological methods.	Carneiro	2001	Cross-sectional	Non probabilistic	Hospital-based	Monocenter	Rural	Unclear/ Not reported	Prospectively	Brazil	Unclear/ Not reported	46.1	All ages	38.6	Hemodialysis	Indirect ELISA	Anti-HCV	Blood
118	Molecular and epidemiological study on nosocomial transmission of HCV in hemodialysis patients in Brazil.	Carneiro	2007	Cross-sectional	Non probabilistic	Hospital-based	Multicenter	Rural	Unclear/ Not reported	Prospectively	Brazil	Apr/2002-Dec/2002	49.3	All ages	59	Hemodialysis	Indirect ELISA	Anti-HCV	Blood
119	Persistence of antibodies to hepatitis C virus in a chronic hemodialysis population.	Carrera	1994	Cohort (Baseline data)	Non probabilistic	Hospital-based	Monocenter	Rural	Unclear/ Not reported	Prospectively	Portugal	Unclear/ Not reported	58.5	Unclear/ Not reported	Unclear/ Not reported	Hemodialysis	Indirect ELISA	Anti-HCV	Blood
120	Prevalence of non-A non-B hepatitis and anti-HCV antibodies in a Portuguese dialysis population.	Carrera	1992	Cross-sectional	Non probabilistic	Hospital-based	Monocenter	Unclear/ Not reported	Unclear/ Not reported	Prospectively	Portugal	Jan/1990	56	Adults: over 18 years	Unclear/ Not reported	Hemodialysis	Indirect ELISA	Anti-HCV	Blood
121	High Prevalence of Hepatitis C Virus Infection in Chronic Hemodialysis Patients.	Carvalho	1999	Cross-sectional	Non probabilistic	Hospital-based	Monocenter	Unclear/ Not reported	Unclear/ Not reported	Prospectively	Brazil	Unclear/ Not reported	Unclear/ Not reported	Unclear/ Not reported	Unclear/ Not reported	Hemodialysis	Indirect ELISA	Anti-HCV	Blood

122	The prevalence of antibodies to hepatitis C virus at two haemodialysis units in South Africa.	Cassidy	1995	Cross-sectional	Non probabilistic	Hospital-based	Monocenter	Unclear/ Not reported	Unclear/ Not reported	Prospectively	South Africa	Unclear/ Not reported	Unclear/ Not reported	Unclear/ Not reported	Unclear/ Not reported	Hemodialysis	Indirect ELISA	Anti-HCV	Blood
123	Comparison of three different tests for assessment of hepatitis C virus in dialysis patients.	Castelnovo	1995	Cross-sectional	Non probabilistic	Hospital-based	Monocenter	Rural	Unclear/ Not reported	Prospectively	Italy	Nov/1992-Dec/1992	57	Unclear/ Not reported	55.1	Hemodialysis; Peritoneal dialysis	Classical RT-PCR	Viral RNA	Blood
124	Prevalence of HBV, HDV, HCV infection and alleged risk factors in the Pistoia (Italy) haemodialysis population.	Catalani	2008	Cross-sectional	Non probabilistic	Hospital-based	Monocenter	Urban	Antiviral naïve	Prospectively	Italy	2007	Unclear/ Not reported	Unclear/ Not reported	Unclear/ Not reported	Hemodialysis	Indirect ELISA	Anti-HCV	Blood
125	Environmental transmission of hepatitis B and hepatitis C viruses within the hemodialysis unit.	Cendoroglo Neto	1995	Cross-sectional	Non probabilistic	Hospital-based	Monocenter	Urban	Unclear/ Not reported	Prospectively	Brazil	Jan/1987-Jan/1990	43	Adults: over 18 years	54.6	Hemodialysis; Peritoneal dialysis	Indirect ELISA	Anti-HCV	Blood
126	Prevalence of hepatitis B and C virus infections among haemodialysis patients in Pune (western India).	Chadha	1993	Cross-sectional	Non probabilistic	Hospital-based	Multicenter	Unclear/ Not reported	Unclear/ Not reported	Prospectively	India	Unclear/ Not reported	Unclear/ Not reported	Unclear/ Not reported	Unclear/ Not reported	Hemodialysis	Indirect ELISA	Anti-HCV	Blood
127	Hepatitis C infection among dialysis patients: a comparison between patients on maintenance haemodialysis and continuous ambulatory peritoneal dialysis.	Chan	1991	Cross-sectional	Non probabilistic	Hospital-based	Multicenter	Unclear/ Not reported	Unclear/ Not reported	Prospectively	China	Unclear/ Not reported	42.9	Unclear/ Not reported	46.6	Hemodialysis; Peritoneal dialysis	Indirect ELISA	Anti-HCV	Blood
128	Discrepancy between serological and virological analysis of viral hepatitis in hemodialysis patients.	Chang	2014	Cross-sectional	Non probabilistic	Hospital-based	Multicenter	Urban	Unclear/ Not reported	Prospectively	China	Jan/2011-Jan/2012	Unclear/ Not reported	Unclear/ Not reported	Unclear/ Not reported	Hemodialysis	Indirect ELISA	Anti-HCV	Blood
129	Hepatitis C among child transfusion and adult renal dialysis patients in Indonesia.	Chanpong	2002	Cross-sectional	Non probabilistic	Hospital-based	Multicenter	Rural	Unclear/ Not reported	Prospectively	Indonesia	Jan/1994-Oct/1994	Unclear/ Not reported	Adults: over 18 years	Unclear/ Not reported	Hemodialysis	Indirect ELISA	Anti-HCV	Blood
130	Prevalence of transfusion-transmitted virus infection in patients on maintenance hemodialysis from New Delhi, India.	Chattopadhyay	2005	Case control	Non probabilistic	Hospital-based	Monocenter	Urban	Unclear/ Not reported	Prospectively	India	2001-2002	Unclear/ Not reported	Unclear/ Not reported	Unclear/ Not reported	Hemodialysis	Indirect ELISA	Anti-HCV	Blood
131	Antibody to hepatitis C virus in risk groups in Canada.	Chaudhary	1992	Cross-sectional	Non probabilistic	Hospital-based	Monocenter	Urban	Unclear/ Not reported	Prospectively	Namibia	1980-1982;1990	Unclear/ Not reported	Unclear/ Not reported	Unclear/ Not reported	Hemodialysis	Indirect ELISA	Anti-HCV	Blood
132	Hepatitis B and C Virus infections associated withrenal replacement therapy in patients with endstage renal disease in a tertiary care hospital inIndia – prevalence, risk factors and outcome.	Chawla	2005	Cross-sectional	Non probabilistic	Hospital-based	Monocenter	Unclear/ Not reported	Unclear/ Not reported	Retroprospectively	India	Unclear/ Not reported	Unclear/ Not reported	Unclear/ Not reported	69.9	Peritoneal dialysis	Indirect ELISA	Anti-HCV	Blood
133	Occult hepatitis B and C in hemodialysis patients in a hyper-endemic area in southern Taiwan.	Chen	2002	Cross-sectional	Non probabilistic	Hospital-based	Monocenter	Urban	Unclear/ Not reported	Prospectively	China	Unclear/ Not reported	Unclear/ Not reported	Unclear/ Not reported	Unclear/ Not reported	Hemodialysis	Classical RT-PCR	Viral RNA	Unclear/ Not reported
134	Superinfection with hepatitis C virus in hemodialysis patients with hepatitis B surface antigenemia: its prevalence and clinical significance in Taiwan.	Chen	1996	Cross-sectional	Non probabilistic	Hospital-based	Monocenter	Urban	Unclear/ Not reported	Prospectively	China	Feb/1993	54	All ages	45.0	Hemodialysis	Indirect ELISA	Anti-HCV	Blood
135	Full-length sequence analysis of a new subtype of hepatitis C virus genotype 1 starting its spread in a hemodialysis unit.	Chevaliez	2009	Cross-sectional	Non probabilistic	Hospital-based	Monocenter	Unclear/ Not reported	Unclear/ Not reported	Prospectively	France	Unclear/ Not reported	Unclear/ Not reported	Unclear/ Not reported	Unclear/ Not reported	Hemodialysis	Unclear/ Not reported	Viral RNA	Unclear/ Not reported
136	Serological monitoring of HCV marker in hemodialysis patients from tertiary care hospitals of Karachi. M	Chishti	2015	Cross-sectional	Non probabilistic	Hospital-based	Multicenter	Unclear/ Not reported	Unclear/ Not reported	Prospectively	Pakistan	Jul/2010-Apr/2011	56.7	Unclear/ Not reported	62.5	Hemodialysis	Enzyme immunoassay (EIA)	Anti-HCV	Blood
137	Association of uraemic pruritus with inflammation and hepatitis infection in	Chiu	2008	Cross-sectional	Non probabilistic	Hospital-based	Monocenter	Unclear/ Not reported	Unclear/ Not reported	Prospectively	China	Unclear/ Not reported	59.7	Unclear/ Not reported	49.5	Hemodialysis	Indirect ELISA	Anti-HCV	Blood

	haemodialysis patients.																		
138	Duration of dialysis increases risk of hepatitis C virus infections among hemodialysis patients in Anambra state, Nigeria.	Chizoba	2018	Cross-sectional	Non probabilistic	Hospital-based	Multicenter	Unclear/ Not reported	Unclear/ Not reported	Prospectively	Nigeria	Feb/2016-Jul/2016	Unclear/ Not reported	Adults: over 18 years	57.8	Hemodialysis	Immunochromatographic test	Anti-HCV	Blood
139	Hepatitis B and C status according to dialysis modality in a Korean Single Center.	Choi	2009	Cross-sectional	Non probabilistic	Hospital-based	Monocenter	Unclear/ Not reported	Unclear/ Not reported	Prospectively	South Korea	Jan/2004-Dec/2008	57.3	Unclear/ Not reported	Unclear/ Not reported	Hemodialysis	Unclear/ Not reported	Anti-HCV	Blood
140	Hepatitis C virus infection and haemodialysis: experience of a district general hospital in Brunei Darussalam.	Chong	2008	Cross-sectional	Non probabilistic	Hospital-based	Monocenter	Urban	Unclear/ Not reported	Prospectively	Brunei Darussalam	Unclear/ Not reported	48.0	Unclear/ Not reported	52.1	Hemodialysis	Indirect ELISA	Anti-HCV	Blood
141	Hepatitis C Virus Infection in Haemodialysis Patients: "Wolf in Sheep's Clothing".	Chopra	2005	Cross-sectional	Non probabilistic	Hospital-based	Monocenter	Unclear/ Not reported	Unclear/ Not reported	Prospectively	India	Unclear/ Not reported	Unclear/ Not reported	Unclear/ Not reported	41.6	Hemodialysis	Classical RT-PCR	Viral RNA	Blood
142	Prevalence and genotype distribution of hepatitis C virus within hemodialysis units in Thailand: Role of HCV core antigen in the assessment of viremia.	Chuaypen	2022	Cross-sectional	Non probabilistic	Hospital-based	Multicenter	Urban	Antiviral naïve	Prospectively	Thailand	2019	Unclear/ Not reported	Unclear/ Not reported	Unclear/ Not reported	Hemodialysis	Indirect ELISA	Anti-HCV	Blood
143	Is hepatitis G virus a real risk for haemodialysis patients?	Cocco	1998	Cross-sectional	Non probabilistic	Hospital-based	Monocenter	Unclear/ Not reported	Unclear/ Not reported	Prospectively	Italy	Unclear/ Not reported	Unclear/ Not reported	Unclear/ Not reported	Unclear/ Not reported	Hemodialysis	Unclear/ Not reported	Anti-HCV	Blood
144	Prevalence of hepatitis C infection in a hemodialysis unit.	Colombo	1992	Cross-sectional	Non probabilistic	Hospital-based	Monocenter	Unclear/ Not reported	Unclear/ Not reported	Prospectively	Italy	Unclear/ Not reported	Unclear/ Not reported	Adults: over 18 years	52.9	Hemodialysis	Indirect ELISA	Anti-HCV	Blood
145	Lower prevalence of anti-hepatitis C antibody in dialysis and renal transplant patients in Ireland.	Conlon	1993	Cross-sectional	Non probabilistic	Hospital-based	Multicenter	Unclear/ Not reported	Unclear/ Not reported	Prospectively	Republic of Ireland	Jan/1990-Mar/1990	49	All ages	Unclear/ Not reported	Hemodialysis	Indirect ELISA	Anti-HCV	Blood
146	Successful use of two rapid HCV assays in a high prevalence Romanian population.	Constantine	1994	Cross-sectional	Non probabilistic	Hospital-based	Monocenter	Unclear/ Not reported	Unclear/ Not reported	Prospectively	Romania	Jul/1993	Unclear/ Not reported	Unclear/ Not reported	Unclear/ Not reported	Hemodialysis	Indirect ELISA	Anti-HCV	Blood
147	Prevalence of antibodies to hepatitis C in dialysis patients and transplant recipients with possible routes of transmission.	Conway	1992	Cross-sectional	Non probabilistic	Hospital-based	Monocenter	Urban	Unclear/ Not reported	Prospectively	United Kingdom	Unclear/ Not reported	Unclear/ Not reported	Unclear/ Not reported	Unclear/ Not reported	Hemodialysis	Indirect ELISA	Anti-HCV	Blood
148	Hepatitis C virus infection in haemodialysis patients: a clinical and virological study.	Corcoran	1994	Cross-sectional	Non probabilistic	Hospital-based	Monocenter	Unclear/ Not reported	Unclear/ Not reported	Retrospectively	United Kingdom	Unclear/ Not reported	Unclear/ Not reported	Unclear/ Not reported	Unclear/ Not reported	Hemodialysis	Indirect ELISA	Anti-HCV	Blood
149	Decline in hepatitis B and C prevalence among hemodialysis patients in Tocantins, Northern Brazil.	Cordeiro	2018	Cross-sectional	Non probabilistic	Hospital-based	Monocenter	Unclear/ Not reported	Unclear/ Not reported	Prospectively	Brazil	Oct/2014-Feb/2015	53.4	Adults: over 18 years	58.6	Hemodialysis	Indirect ELISA	Anti-HCV	Blood
150	Hepatitis C virus (HCV) infection in haemodialysed patients: HCV-RNA and anti-HCV antibodies (third-generation assays).	Couroucé	1995	Cross-sectional	Non probabilistic	Hospital-based	Monocenter	Unclear/ Not reported	Unclear/ Not reported	Prospectively	France	1993	Unclear/ Not reported	Unclear/ Not reported	Unclear/ Not reported	Hemodialysis	Indirect ELISA	Anti-HCV	Blood
151	Hepatitis virus infection in haemodialysis patients from Moldavia.	Covic	1999	Cross-sectional	Non probabilistic	Hospital-based	Monocenter	Urban	Unclear/ Not reported	Prospectively	Romania	Unclear/ Not reported	41.3	Adults: over 18 years	59.1	Hemodialysis	Indirect ELISA	Anti-HCV	Blood
152	A survey of hepatitis C virus infection in haemodialysis patients over a 7-year follow-up.	Cristina	1997	Cross-sectional	Non probabilistic	Hospital-based	Monocenter	Unclear/ Not reported	Unclear/ Not reported	Prospectively	Italy	Nov/1989-Dec/1996	Unclear/ Not reported	Adults: over 18 years	Unclear/ Not reported	Hemodialysis	Indirect ELISA	Anti-HCV	Blood
153	Hepatitis C virus in dialysis units: a multicenter study.	Da Porto	1992	Cross-sectional	Probabilistic	Hospital-based	Multicenter	Urban/Rural	Unclear/ Not reported	Prospectively	Italy	Unclear/ Not reported	Unclear/ Not reported	Unclear/ Not reported	Unclear/ Not reported	Hemodialysis	Indirect ELISA	Anti-HCV	Blood
154	Evidence of association between hepatitis C virus genotype 2b and nosocomial transmissions in hemodialysis centers from southern Brazil.	da Silva	2013	Cross-sectional	Non probabilistic	Hospital-based	Monocenter	Unclear/ Not reported	Unclear/ Not reported	Prospectively	Brazil	Jan/2009-Aug/2010	56.9	Adults: over 18 years	57.2	Hemodialysis	Indirect ELISA	Anti-HCV	Blood
155	Prevalence of HCV-RNA-positive patients in a dialysis unit in Germany.	Da silva cardoso	1994	Cross-sectional	Probabilistic	Hospital-based	Monocenter	Urban/Rural	Unclear/ Not reported	Prospectively	Germany	Unclear/ Not reported	Unclear/ Not reported	Unclear/ Not reported	Unclear/ Not reported	Hemodialysis	Indirect ELISA	Anti-HCV	Blood

156	Prevalence and risk factors of hepatitis C virus among hemodialysis patients.	Dadgaran	2005	Cross-sectional	Non probabilistic	Hospital-based	Multicenter	Unclear/ Not reported	Unclear/ Not reported	Prospectively	Iran	Unclear/ Not reported	Unclear/ Not reported	Unclear/ Not reported	Unclear/ Not reported	Hemodialysis	Indirect ELISA	Anti-HCV	Blood
157	[Investigation of hepatitis B and hepatitis C virus infections by serological and molecular methods in hemodialysis patients].	Dağlar	2014	Cross-sectional	Probabilistic	Hospital-based	Multicenter	Urban/Rural	Unclear/ Not reported	Retrospectively	Turkey	2006	51.1	All ages	60	Hemodialysis	Indirect ELISA	Anti-HCV	Blood
158	SEN virus infection among patients on maintenance hemodialysis in southern Taiwan.	Dai	2005	Cross-sectional	Probabilistic	Hospital-based	Monocenter	Urban/Rural	Antiviral naïve/ On antiviral	Prospectively	China	Unclear/ Not reported	57.4	Adults: over 18 years	80	Hemodialysis	Indirect ELISA	Anti-HCV	Blood
159	Epidemiology and clinical significance of chronic hepatitis-related viruses infection in hemodialysis patients from Taiwan.	Dai	2002	Cross-sectional	Probabilistic	Hospital-based	Monocenter	Urban/Rural	Unclear/ Not reported	Prospectively	China	Unclear/ Not reported	58.1	Adults: over 18 years	48.2	Hemodialysis	Indirect ELISA	Anti-HCV	Blood
160	Absence of HCV viraemia in anti-HCV-negative haemodialysis patients.	Dalekos	1998	Cross-sectional	Probabilistic	Hospital-based	Monocenter	Urban/Rural	Unclear/ Not reported	Retrospectively	Greece	Unclear/ Not reported	61	All ages	66.7	Hemodialysis	Classical RT-PCR	Viral RNA	Blood
161	Prevalence of hepatitis B and C virus infections and immunity among hemodialysis patients in the Mazandaran province, Northern Iran.	Darrudi	2022	Cross-sectional	Non probabilistic	Hospital-based	Multicenter	Urban	Antiviral naïve	Retrospectively	Iran	2019-2020	62.25	All ages	50.9	Hemodialysis	Indirect ELISA	Anti-HCV	Blood
162	Utility of routine real time quantitative PCR monitoring of HCV infection in haemodialysis patients.	Datta	2015	Cross-sectional	Non probabilistic	Hospital-based	Multicenter	Urban	Antiviral naïve	Prospectively	India	2010-2012	47.6	Unclear/ Not reported	66.6	Hemodialysis	Real-time RT-PCR	Viral RNA	Blood
163	[Natural history of HCV infection and risk of death in a cohort of patients on long-term hemodialysis].	Dattolo	2006	Cohort (Baseline data)	Probabilistic	Hospital-based	Monocenter	Urban	Unclear/ Not reported	Prospectively	Italy	Unclear/ Not reported	Unclear/ Not reported	Unclear/ Not reported	Unclear/ Not reported	Hemodialysis	Unclear/ Not reported	Anti-HCV	Unclear/ Not reported
164	Blood-borne viruses in the haemodialysis-dependent population attending Top End Northern Territory facilities 2000-2009.	Davies	2012	Cross-sectional	Probabilistic	Hospital-based	Monocenter	Urban/Rural	Unclear/ Not reported	Retrospectively	Australia	2000-2009	50	All ages	46.6	Hemodialysis	Chemiluminescent immunoassay	Anti-HCV	Blood
165	Prevalence of hepatitis C virus antibodies among different populations of relative and attributable risk.	Daw	2002	Cross-sectional	Probabilistic	Hospital-based	Monocenter	Urban	Unclear/ Not reported	Prospectively	Libya	1999-2001	Unclear/ Not reported	Unclear/ Not reported	Unclear/ Not reported	Hemodialysis	Indirect ELISA	Anti-HCV	Blood
166	Nosocomial transmission of hepatitis C virus in haemodialysis patients.	de Lamballerie	1996	Cross-sectional	Probabilistic	Hospital-based	Monocenter	Urban	Unclear/ Not reported	Prospectively	France	Dec/1994	58	Adults: over 18 years	51	Hemodialysis	Indirect ELISA	Anti-HCV	Blood
167	[Prevalence of anti hepatitis C antibodies in patients with chronic renal failure receiving conservative therapy].	de los Rios	2006	Cross-sectional	Probabilistic	Hospital-based	Monocenter	Urban/Rural	Unclear/ Not reported	Prospectively	Peru	Feb/2003-Feb/2004	56.6	Adults: over 18 years	54.1	Hemodialysis	Indirect ELISA	Anti-HCV	Blood
168	Prevalence of hepatitis C and G virus infection in chronic hemodialysis patients.	de medina	1998	Cross-sectional	Probabilistic	Hospital-based	Monocenter	Urban	Unclear/ Not reported	Prospectively	United States of America	Unclear/ Not reported	Unclear/ Not reported	Adults: over 18 years	61.9	Hemodialysis	Indirect ELISA	Anti-HCV	Blood
169	Quantitative detection of hepatitis C virus RNA in patients undergoing hemodialysis.	de Medina	1997	Cross-sectional	Probabilistic	Hospital-based	Monocenter	Urban	Unclear/ Not reported	Prospectively	United States of America	Jul/1992-Dec/1992	52	Adults: over 18 years	75	Hemodialysis	Indirect ELISA	Anti-HCV	Blood
170	Improved detection of antibodies to hepatitis C virus in dialysis patients using a second-generation enzyme immunoassay.	de Medina	1992	Cross-sectional	Probabilistic	Hospital-based	Monocenter	Urban	Unclear/ Not reported	Prospectively	United States of America	Unclear/ Not reported	54.5	Adults: over 18 years	58.6	Hemodialysis	Indirect ELISA	Anti-HCV	Blood
171	Hepatitis C, HCV genotypes and hepatic siderosis in patients with chronic renal failure on haemodialysis in Brazil.	de Paula Farah	2007	Cohort (Baseline data)	Probabilistic	Hospital-based	Monocenter	Urban	Unclear/ Not reported	Prospectively	Brazil	May/2000-Dec/2003	43.6	Adults: over 18 years	Unclear/ Not reported	Hemodialysis	Classical RT-PCR	Viral RNA	Blood
172	Prevalence of viral hepatitis (Hep-B and Hep-C) infection in	Deep	2022	Cross-sectional	Non probabilistic	Hospital-based	Monocenter	Urban	Antiviral naïve	Prospectively	India	Jun/2018-Jun/2020	Unclear/ Not reported	Unclear/ Not reported	Unclear/ Not reported	Hemodialysis	Indirect ELISA	Anti-HCV	Unclear/ Not reported

	advanced renal failure patients: a tertiary care centre study.																		
173	Outbreak of hepatitis C virus infection in a hemodialysis unit: potential transmission by the hemodialysis machine?	Delarocque-Astagneau	2002	Case control	Probabilistic	Hospital-based	Monocenter	Urban	Unclear/ Not reported	Prospectively	France	Jan/1994-Jul/1997	57	Adults: over 18 years	55.5	Hemodialysis	Indirect ELISA	Anti-HCV	Blood
174	Prevalence and incidence of hepatitis C virus (HCV) in hemodialysis patients: study of risk factors.	Dentico	1992	Cross-sectional	Probabilistic	Hospital-based	Monocenter	Urban/Rural	Unclear/ Not reported	Prospectively	Italy	1984-1990	Unclear/ Not reported	Unclear/ Not reported	Unclear/ Not reported	Hemodialysis	Indirect ELISA	Anti-HCV	Blood
175	Hepatitis C virus-RNA, immunoglobulin M anti-HCV and risk factors in haemodialysis patients.	Dentico	1999	Cross-sectional	Probabilistic	Hospital-based	Monocenter	Urban/Rural	Unclear/ Not reported	Prospectively	Italy	Unclear/ Not reported	Unclear/ Not reported	Unclear/ Not reported	Unclear/ Not reported	Hemodialysis	Unclear/ Not reported	Anti-HCV	Unclear/ Not reported
176	Hepatitis C virus serotypes in haemodialysis patients in South-East Italy.	Dentico	2000	Cohort (Baseline data)	Probabilistic	Hospital-based	Monocenter	Urban/Rural	Unclear/ Not reported	Prospectively	Italy	Dec/1994	43	Adults: over 18 years	56.5	Hemodialysis	Indirect ELISA	Anti-HCV	Blood
177	Hepatitis C virus in hemodialysis patients.	Dentico	1992	Cross-sectional	Probabilistic	Hospital-based	Monocenter	Urban/Rural	Unclear/ Not reported	Prospectively	Italy	Unclear/ Not reported	Unclear/ Not reported	Adults: over 18 years	Unclear/ Not reported	Hemodialysis	Indirect ELISA	Anti-HCV	Blood
178	Detection of antibodies to HCV in haemodialysis patients using two second generation ELISA tests.	Dentico	1993	Cross-sectional	Probabilistic	Hospital-based	Monocenter	Urban/Rural	Unclear/ Not reported	Prospectively	Italy	Unclear/ Not reported	Unclear/ Not reported	Unclear/ Not reported	Unclear/ Not reported	Hemodialysis	Indirect ELISA	Anti-HCV	Blood
179	HCV third generation test in hemodialysis patients.	Dentico	1995	Cross-sectional	Probabilistic	Hospital-based	Monocenter	Urban/Rural	Unclear/ Not reported	Prospectively	Italy	Unclear/ Not reported	Unclear/ Not reported	Unclear/ Not reported	Unclear/ Not reported	Hemodialysis	Classical RT-PCR	Viral RNA	Blood
180	Reduced antibody reactivity to hepatitis C virus antigens in hemodialysis patients coinfected with hepatitis B virus.	Devesa	1997	Cross-sectional	Probabilistic	Hospital-based	Multicenter	Urban/Rural	Unclear/ Not reported	Prospectively	United States of America	Apr/1994-Apr/1995	48	Unclear/ Not reported	Unclear/ Not reported	Hemodialysis	Indirect ELISA	Anti-HCV	Blood
181	Risk factors of hepatitis C virus infection in patients on hemodialysis: a multivariate analysis based on a dialysis register in Central Italy.	Di lallo	1999	Cross-sectional	Probabilistic	Hospital-based	Multicenter	Urban	Unclear/ Not reported	Prospectively	Italy	Jun/1994-Jun/1996	61.6	All ages	59.3	Hemodialysis	Unclear/ Not reported	Anti-HCV	Unclear/ Not reported
182	Hepatitis C virus antibodies in dialysis pediatric patients.	Di loreto	1992	Cross-sectional	Probabilistic	Hospital-based	Monocenter	Urban/Rural	Unclear/ Not reported	Prospectively	Italy	Unclear/ Not reported	Unclear/ Not reported	Unclear/ Not reported	Unclear/ Not reported	Hemodialysis	Indirect ELISA	Anti-HCV	Blood
183	Confirmation of high prevalence of hepatitis C antibodies in hemodialysis patients by second generation immunoblot assay.	Di maggio	1992	Cross-sectional	Probabilistic	Hospital-based	Monocenter	Urban/Rural	Unclear/ Not reported	Prospectively	Italy	Unclear/ Not reported	54.2	Unclear/ Not reported	51.6	Hemodialysis	Indirect ELISA	Anti-HCV	Blood
184	Epidemiology of hepatitis C virus among long-term dialysis patients: a 9-year study in an Italian region.	Di Napoli	2006	Cohort (Baseline data)	Probabilistic	Hospital-based	Monocenter	Urban/Rural	Unclear/ Not reported	Prospectively	Italy	Jan/1995-Dec/2003	61.6	Unclear/ Not reported	61.6	Hemodialysis	Indirect ELISA	Anti-HCV	Blood
185	Further evidence for nosocomial spread of hepatitis C virus infection in hemodialysis units.	Dimković	1996	Cross-sectional	Probabilistic	Hospital-based	Monocenter	Urban/Rural	Unclear/ Not reported	Prospectively	Serbia	1991-1994	54.3	Unclear/ Not reported	58.8	Hemodialysis	Indirect ELISA	Anti-HCV	Blood
186	[Prevalence of hepatitis B and C viruses in a chronic hemodialysis center in Dakar].	Diouf	2000	Cross-sectional	Probabilistic	Hospital-based	Monocenter	Urban	Unclear/ Not reported	Prospectively	Senegal	Jun/1996-Jun/1997	48	Unclear/ Not reported	Unclear/ Not reported	Hemodialysis	Unclear/ Not reported	Anti-HCV	Unclear/ Not reported
187	Prevention of nosocomial transmission of hepatitis C infection in a hemodialysis unit. A prospective study	Djordjevic	2000	Cohort (Baseline data)	Non probabilistic	Hospital-based	Monocenter	Unclear/ Not reported	Unclear/ Not reported	Prospectively	Yugoslavia	Unclear/ Not reported	Unclear/ Not reported	Unclear/ Not reported	Unclear/ Not reported	Hemodialysis	Unclear/ Not reported	Anti-HCV	Unclear/ Not reported
188	Hepatitis C virus infection in patients on peritoneal dialysis, hemodialysis and in dialysis staff members in south Serbia.	Djordjević	1996	Cross-sectional	Probabilistic	Hospital-based	Monocenter	Urban	Unclear/ Not reported	Prospectively	Serbia	Sept/1994-Oct/1994	Unclear/ Not reported	Unclear/ Not reported	Unclear/ Not reported	Hemodialysis; Peritoneal dialysis	Indirect ELISA	Anti-HCV	Blood
189	[Prevalence and risk factors of hepatitis C virus infection in patients on hemodialysis: results of a Moroccan study].	Doblali	2014	Cross-sectional	Probabilistic	Hospital-based	Multicenter	Urban	Unclear/ Not reported	Retrospectively	Morocco	April/2010-Sept2012	52.6	Unclear/ Not reported	56.7	Hemodialysis	Indirect ELISA	Anti-HCV	Blood

190	[Seroprevalence of hepatitis C at the Military Hospital Mohammed V of Rabat].	Doblali	2014	Cross-sectional	Non probabilistic	Hospital-based	Monocenter	Urban	Unclear/ Not reported	Retrospectively	Morocco	Jan/2010-Dec/2012	Unclear/ Not reported	Adults: over 18 years	Unclear/ Not reported	Hemodialysis	Direct ELISA, Indirect ELISA	Anti-HCV, Core Ag	Blood
191	Quantitation of hepatitis C viral RNA in sera of hemodialysis patients: gender-related differences in viral load.	DuBois	1994	Cross-sectional	Non probabilistic	Hospital-based	Monocenter	Unclear/ Not reported	Unclear/ Not reported	Prospectively	United States of America	Unclear/ Not reported	63	Adults: over 18 years	68.2	Hemodialysis	Indirect ELISA	Anti-HCV	Blood
192	Hepatitis C virus in Vietnam: high prevalence of infection in dialysis and multi-transfused patients involving diverse and novel virus variants.	Dunford	2012	Cross-sectional	Non probabilistic	Hospital-based	Multicenter	Urban	Unclear/ Not reported	Prospectively	Vietnam	2008-2009	Unclear/ Not reported	Unclear/ Not reported	Unclear/ Not reported	Hemodialysis	Direct ELISA, Indirect ELISA	Anti-HCV, Core Ag	Blood
193	Hepatitis B and C virus infections among patients with end stage renal disease in a low-resourced hemodialysis center in Vietnam: a cross-sectional study.	Duong	2015	Cross-sectional	Non probabilistic	Hospital-based	Multicenter	Unclear/ Not reported	Unclear/ Not reported	Prospectively	Vietnam	Oct/2012-Jan/2013	53	Adults: over 18 years	48	Hemodialysis	Chemiluminescent immunoassay	Viral antigen	Blood
194	Screening haemodialysis patients for hepatitis C in Vietnam: The inconsistency between common hepatitis C virus serological and virological tests.	Duong	2019	Cross-sectional	Non probabilistic	Hospital-based	Multicenter	Rural	Unclear/ Not reported	Prospectively	Vietnam	Oct/2012-Oct2014	57	Adults: over 18 years	48	Hemodialysis	Real-time RT-PCR	Viral RNA	Blood
195	Hepatitis C virus infection among chronic dialysis patients in the southeast of France. Provence-Alpes-Côte d'Azur Nephrologists Group.	Dussol	1995	Cross-sectional	Non probabilistic	Hospital-based	Multicenter	Rural	Unclear/ Not reported	Prospectively	France	Unclear/ Not reported	59	Adults: over 18 years	20	Hemodialysis; Peritoneal dialysis	Indirect ELISA	Anti-HCV	Blood
196	Detection of hepatitis C infection by polymerase chain reaction among hemodialysis patients.	Dussol	1993	Cross-sectional	Non probabilistic	Hospital-based	Monocenter	Unclear/ Not reported	Unclear/ Not reported	Prospectively	France	1992	56	Adults: over 18 years	56.5	Hemodialysis	Indirect ELISA	Anti-HCV	Blood
197	Hepatitis C virus infection among dialysis patients in United arab emirates.	El Shahat	1995	Cross-sectional	Non probabilistic	Hospital-based	Monocenter	Unclear/ Not reported	Unclear/ Not reported	Prospectively	United Arab Emirates	Jan/1991-Dec/1993	41.8	Adults: over 18 years	67.5	Hemodialysis	Indirect ELISA	Anti-HCV	Blood
198	Hepatitis B and C Infections in Patients With Prolonged Hemodialysis Secondary to Chronic Renal Failure.	Elahi	2020	Cross-sectional	Non probabilistic	Hospital-based	Monocenter	Urban	Unclear/ Not reported	Prospectively	United Arab Emirates	Jan/1991-Dec/1993	41.8	Unclear/ Not reported	Unclear/ Not reported	Hemodialysis	Unclear/ Not reported	Anti-HCV	Unclear/ Not reported
199	Hepatitis C virus infection in hemodialysis patients in Sudan: two centers' report.	El-Amin	2007	Cross-sectional	Non probabilistic	Hospital-based	Multicenter	Unclear/ Not reported	Unclear/ Not reported	Prospectively	Sudan	Jan/2005-Mar/2005	43.6	All ages	71.6	Hemodialysis	Indirect ELISA	Anti-HCV	Blood
200	Diagnostic Performance of an Immunoassay for Simultaneous Detection of Hcv Core Antigen and Antibodies among Haemodialysis Patients.	El-Emshaty	2011	Cross-sectional	Non probabilistic	Hospital-based	Monocenter	Unclear/ Not reported	Unclear/ Not reported	Prospectively	Egypt	Unclear/ Not reported	49.9	Adults: over 18 years	30.8	Hemodialysis	Classical RT-PCR	Viral RNA	Blood
201	Antibodies against hepatitis C virus (anti-HCV) in haemodialysis patients: association with hepatitis B serological markers.	Elisaf	1991	Cross-sectional	Non probabilistic	Hospital-based	Monocenter	Unclear/ Not reported	Unclear/ Not reported	Prospectively	Greece	Unclear/ Not reported	52	All ages	60.7	Hemodialysis	Indirect ELISA	Anti-HCV	Blood
202	Prevalence and risk factors of hepatitis B and C viruses among haemodialysis patients in Gaza strip, Palestine.	El-kader	2010	Cross-sectional	Non probabilistic	Hospital-based	Multicenter	Unclear/ Not reported	Unclear/ Not reported	Prospectively	Palestine	Aug/2007-Sep/2007	46.7	All ages	53.2	Hemodialysis	Real-time RT-PCR	Viral RNA	Blood
203	Hepatitis C virus infection in patients on maintenance dialysis in kuwait: epidemiological profile and efficacy of prophylaxis.	El-Reshaid	1995	Cross-sectional	Non probabilistic	Hospital-based	Multicenter	Unclear/ Not reported	Unclear/ Not reported	Retrospectively	Kuwait	Dec/1994	50	All ages	48.7	Hemodialysis	Indirect ELISA	Anti-HCV	Blood
204	PREVALENCE OF ANTI-HEPATITIS-C VIRUS-ANTIBODIES AND HEPATITIS-C VIRUS VIREMIA IN CHRONIC-HEMODIALYSIS PATIENTS IN LIBYA.	Elzouki	1995	Cross-sectional	Non probabilistic	Hospital-based	Monocenter	Urban	Antiviral naïve	Prospectively	Libya	Unclear/ Not reported	41	All ages	57.5	Hemodialysis	Indirect ELISA	Anti-HCV	Blood

205	HCV in hemodialysis patients in Benghazi, Libya.	El-Zouki	1993	Cross-sectional	Non probabilistic	Hospital-based	Monocenter	Urban	Unclear/ Not reported	Prospectively	Libya	Unclear/ Not reported	Unclear/ Not reported	Unclear/ Not reported	Unclear/ Not reported	Hemodialysis	Indirect ELISA	Anti-HCV	Blood
206	Marked reduction in the prevalence of hepatitis C virus infection in hemodialysis patients: causes and consequences.	Espinosa	2004	Cross-sectional	Non probabilistic	Hospital-based	Multicenter	Urban	Antiviral naïve	Prospectively	Spain	Jan/1992-Dec/2002	52.1	Unclear/ Not reported	58.8	Hemodialysis	Indirect ELISA	Anti-HCV	Blood
207	Risk of death and liver cirrhosis in anti-HCV positive long-term haemodialysis patients.	Espinosa	2001	Cohort (Baseline data)	Non probabilistic	Hospital-based	Multicenter	Unclear/ Not reported	Unclear/ Not reported	Prospectively	Spain	Jan/1992-Dec/1999	Unclear/ Not reported	Unclear/ Not reported	53.1	Hemodialysis	Indirect ELISA	Anti-HCV	Blood
208	Genotyping hepatitis C virus from hemodialysis patients in Central Brazil by line probe assay and sequence analysis.	Espírito-Santo	2007	Cross-sectional	Non probabilistic	Hospital-based	Multicenter	Urban	Unclear/ Not reported	Prospectively	Brazil	Apr/2002-Dec/2002	Unclear/ Not reported	Unclear/ Not reported	Unclear/ Not reported	Hemodialysis	Classical RT-PCR	Viral RNA	Blood
209	De novo HCV infection among dialysis patients: a prospective study by HCV core antigen ELISA assay.	Fabrizi	2005	Cohort (Baseline data)	Non probabilistic	Hospital-based	Monocenter	Urban	Unclear/ Not reported	Prospectively	Italy	Aug/2000-Jul/2003	62.5	Unclear/ Not reported	42	Hemodialysis	Direct ELISA	Viral antigen	Blood
210	Hepatitis C virus infection and hepatic function in chronic hemodialysis patients.	Fabrizi	1992	Cross-sectional	Non probabilistic	Hospital-based	Monocenter	Urban	Unclear/ Not reported	Prospectively	Italy	Unclear/ Not reported	Unclear/ Not reported	Unclear/ Not reported	Unclear/ Not reported	Hemodialysis	Indirect ELISA	Anti-HCV	Blood
211	Influence of hepatitis C virus (HCV) viraemia upon serum aminotransferase activity in chronic dialysis patients.	Fabrizi	1997	Cross-sectional	Non probabilistic	Hospital-based	Multicenter	Urban	Antiviral naïve	Prospectively	Italy	Unclear/ Not reported	61.9	Adults: over 18 years	53.5	Hemodialysis; Peritoneal dialysis	Indirect ELISA	Anti-HCV	Blood
212	Hepatitis E virus infection in haemodialysis patients: a seroepidemiological survey.	Fabrizi	1997	Cross-sectional	Non probabilistic	Hospital-based	Monocenter	Urban	Antiviral naïve	Prospectively	Italy	Feb/1995	61.1	Adults: over 18 years	55.4	Hemodialysis	Indirect ELISA	Anti-HCV	Blood
213	Incidence of seroconversion for hepatitis C virus in chronic haemodialysis patients: a prospective study.	Fabrizi	1994	Cohort (Baseline data)	Non probabilistic	Hospital-based	Monocenter	Urban	Antiviral naïve	Prospectively	Italy	Feb/1990-Mar/1994	59.8	Adults: over 18 years	59	Hemodialysis	Indirect ELISA	Anti-HCV	Blood
214	Virological characteristics of hepatitis C virus infection in chronic hemodialysis patients: a cross-sectional study.	Fabrizi	1995	Cross-sectional	Non probabilistic	Hospital-based	Monocenter	Urban	Antiviral naïve	Prospectively	Italy	Unclear/ Not reported	Unclear/ Not reported	Unclear/ Not reported	Unclear/ Not reported	Hemodialysis	Indirect ELISA	Anti-HCV	Blood
215	Molecular epidemiology of hepatitis C virus infection in dialysis patients.	Fabrizi	1997	Cross-sectional	Non probabilistic	Hospital-based	Multicenter	Rural	Antiviral naïve	Prospectively	Italy	Feb/1996	62.3	Adults: over 18 years	44	Hemodialysis	Classical RT-PCR	Viral RNA	Blood
216	Serologic survey for control of hepatitis C in haemodialysis patients: third-generation assays and analysis of costs.	Fabrizi	1997	Cross-sectional	Non probabilistic	Hospital-based	Monocenter	Urban	Antiviral naïve	Prospectively	Italy	May1994	62.6	Adults: over 18 years	58.4	Hemodialysis	Indirect ELISA	Anti-HCV	Blood
217	Acquisition of hepatitis C virus in hemodialysis patients: a prospective study by branched DNA signal amplification assay.	Fabrizi	1998	Cross-sectional	Non probabilistic	Hospital-based	Multicenter	Urban	Antiviral naïve	Prospectively	United States of America	Apr/1994-Mar/1995	60.7	Unclear/ Not reported	56	Hemodialysis	Indirect ELISA	Anti-HCV	Blood
218	Antibodies to hepatitis C virus (HCV) and transaminase concentration in chronic haemodialysis patients: a study with second-generation assays.	Fabrizi	1993	Cross-sectional	Non probabilistic	Hospital-based	Multicenter	Rural	Antiviral naïve	Prospectively	Italy	Unclear/ Not reported	60.6	All ages	55.3	Hemodialysis	Indirect ELISA	Anti-HCV	Blood
219	Conflicting results of hepatitis C virus serological tests in haemodialysis patients.	Fabrizi	1993	Cross-sectional	Non probabilistic	Hospital-based	Multicenter	Urban	Unclear/ Not reported	Prospectively	Italy	Unclear/ Not reported	Unclear/ Not reported	Unclear/ Not reported	Unclear/ Not reported	Hemodialysis	Indirect ELISA	Anti-HCV	Blood
220	Low risk of sexual transmission of hepatitis C virus to spouses of infected hemodialysis patients.	Fadil-Romao	2006	Cross-sectional	Non probabilistic	Hospital-based	Monocenter	Urban	Antiviral naïve	Prospectively	Brazil	Unclear/ Not reported	35.7	Adults: over 18 years	57.6	Hemodialysis	Indirect ELISA	Anti-HCV	Blood
221	Prevalence of antibodies to hepatitis C virus in hemodialysis patients in Riyadh.	Fakunle	1991	Cross-sectional	Non probabilistic	Hospital-based	Monocenter	Urban	Antiviral naïve	Prospectively	Saudi Arabia	Unclear/ Not reported	40.4	All ages	63.1	Hemodialysis	Indirect ELISA	Anti-HCV	Blood
222	Hepatitis C virus RNA (HCV RNA) and viral types in dialysis	Falasca	1999	Cross-sectional	Non probabilistic	Hospital-based	Multicenter	Urban	Antiviral naïve	Prospectively	Senegal	Unclear/ Not reported	47	Adults: over 18 years	95.4	Hemodialysis	Indirect ELISA	Anti-HCV	Blood

	patients in Dakar, Senegal.																		
223	Hepatitis C and haemodialysis.	Faoagali	1990	Cross-sectional	Non probabilistic	Hospital-based	Monocenter	Rural	Antiviral naïve	Prospectively	Australia	1989	Unclear/ Not reported	Unclear/ Not reported	Unclear/ Not reported	Hemodialysis; Peritoneal dialysis	Unclear/ Not reported	Anti-HCV	Blood
224	Hepatitis C, HCV genotypes and hepatic chronic renal failure on haemodialysis in plantation siderosis in patients with Brazil.	Farah	2007	Cross-sectional	Non probabilistic	Hospital-based	Multicenter	Urban	Antiviral naïve	Prospectively	Brazil	May/2000-Dec/2003	43.6	Adults: over 18 years	Unclear/ Not reported	Hemodialysis	Classical RT-PCR	Viral RNA	Blood
225	Hepatic and extra-hepatic sequelae, and prevalence of viral hepatitis C infection estimated from routine data in at-risk groups.	Faustini	2010	Cross-sectional	Non probabilistic	Hospital-based	Multicenter	Urban/Rural	Antiviral naïve/ On antiviral	Prospectively	Italy	1997-2001	64	Unclear/ Not reported	60.1	Hemodialysis	Indirect ELISA	Anti-HCV	Blood
226	Antibodies to hepatitis C virus in patients with chronic renal disease in Sri Lanka.	Fernando	2002	Cross-sectional	Non probabilistic	Hospital-based	Monocenter	Urban	Antiviral naïve	Prospectively	Sri Lanka	Jul/1997-Dec/1999	Unclear/ Not reported	Unclear/ Not reported	Unclear/ Not reported	Hemodialysis	Indirect ELISA	Anti-HCV	Blood
227	Hepatitis G virus infection in a haemodialysis unit: prevalence and clinical implications.	Forns	1997	Cross-sectional	Non probabilistic	Hospital-based	Monocenter	Urban	Antiviral naïve	Prospectively	Spain	1996	58	Unclear/ Not reported	67	Hemodialysis	Indirect ELISA	Anti-HCV	Blood
228	Incidence and risk factors of hepatitis C virus infection in a haemodialysis unit.	Forns	1997	Cross-sectional	Non probabilistic	Hospital-based	Monocenter	Urban	Antiviral naïve	Prospectively	Spain	1991-1995	58	Unclear/ Not reported	61	Hemodialysis	Indirect ELISA	Anti-HCV	Blood
229	High prevalence of TT virus (TTV) infection in patients on maintenance hemodialysis: frequent mixed infections with different genotypes and lack of evidence of associated liver disease.	Forns	1999	Cross-sectional	Non probabilistic	Hospital-based	Monocenter	Urban	Antiviral naïve	Prospectively	Spain	Unclear/ Not reported	58	Unclear/ Not reported	Unclear/ Not reported	Hemodialysis	Unclear/ Not reported	Anti-HCV	Blood
230	Hepatitis C in the health care setting. II. Seroprevalence among hemodialysis staff and patients in suburban New York City.	Forseter	1993	Cross-sectional	Non probabilistic	Hospital-based	Monocenter	Urban	Unclear/ Not reported	Prospectively	United States of America	1991	Unclear/ Not reported	Unclear/ Not reported	Unclear/ Not reported	Hemodialysis	Indirect ELISA	Anti-HCV	Blood
231	Epidemiological and virological study of hepatitis c virus infection in hemodialysis	Foullous	2015	Cross-sectional	Non probabilistic	Hospital-based	Multicenter	Urban	Antiviral naïve	Prospectively	Morocco	Unclear/ Not reported	48	Unclear/ Not reported	Unclear/ Not reported	Hemodialysis	Indirect ELISA	Anti-HCV	Blood
232	Prevalence, genotypes and risk factors associated with hepatitis C virus infection in hemodialysis patients in Campo Grande, MS, Brazil.	Freitas	2008	Cross-sectional	Non probabilistic	Hospital-based	Multicenter	Urban	Unclear/ Not reported	Prospectively	Brazil	2003	48	All ages	59.5	Hemodialysis	Indirect ELISA	Anti-HCV	Blood
233	The prevalence of anti-HCV antibodies in hemodialysis patients.	Fujiyama	1991	Cross-sectional	Non probabilistic	Hospital-based	Monocenter	Urban	Unclear/ Not reported	Prospectively	Japan	Unclear/ Not reported	56.8	Adults: over 18 years	56.2	Hemodialysis	Indirect ELISA	Anti-HCV	Blood
234	Changes in prevalence of anti-HCV antibodies associated with preventive measures among hemodialysis patients and dialysis staff.	Fujiyama	1995	Cross-sectional	Non probabilistic	Hospital-based	Multicenter	Urban	Antiviral naïve	Prospectively	Japan	Unclear/ Not reported	Unclear/ Not reported	Unclear/ Not reported	Unclear/ Not reported	Hemodialysis	Indirect ELISA	Anti-HCV	Unclear/ Not reported
235	Prevalence of hepatitis C virus antibodies in hemodialysis patients and dialysis staff.	Fujiyama	1992	Cross-sectional	Non probabilistic	Hospital-based	Multicenter	Urban	Antiviral naïve	Prospectively	Japan	Unclear/ Not reported	Unclear/ Not reported	Unclear/ Not reported	Unclear/ Not reported	Hemodialysis	Indirect ELISA	Anti-HCV	Unclear/ Not reported
236	Lower hepatitis G virus infection prevalence compared to hepatitis B and C virus infection prevalences.	Furusyo	2000	Cross-sectional	Non probabilistic	Hospital-based	Monocenter	Urban	Unclear/ Not reported	Prospectively	Japan	1989-1992	Unclear/ Not reported	All ages	57.8	Hemodialysis	Indirect ELISA	Anti-HCV	Blood
237	Acute hepatitis C among Japanese hemodialysis patients: a prospective 9-year study.	Furusyo	2001	Cross-sectional	Non probabilistic	Hospital-based	Monocenter	Urban	Unclear/ Not reported	Prospectively	Japan	Jan/1990-Dec/1998	54.9	Unclear/ Not reported	60.9	Hemodialysis	Indirect ELISA	Anti-HCV	Blood
238	Effect of isolation measures on the incidence and prevalence of hepatitis C virus infection in hernodialysis.	Gallego	2006	Cross-sectional	Non probabilistic	Hospital-based	Multicenter	Urban	Antiviral naïve	Prospectively	Spain	Dec/1992-Dec/2003	Unclear/ Not reported	Unclear/ Not reported	Unclear/ Not reported	Hemodialysis; Peritoneal dialysis	Indirect ELISA	Anti-HCV	Unclear/ Not reported
239	Hepatitis C in hemodialysis: the	Galperim	2010	Cross-sectional	Non probabilistic	Hospital-based	Multicenter	Urban	Unclear/ Not reported	Prospectively	Brazil	2005-2006	54.4	Adults: over 18 years	58.2	Hemodialysis	Chemiluminescent immunoassay	Anti-HCV	Blood

	contribution of injection drug use.																		
240	[Antibodies against hepatitis C virus in patients with liver diseases and in risk subjects. Preliminary report].	Garassini	1990	Cross-sectional	Non probabilistic	Hospital-based	Monocenter	Urban	Antiviral naïve	Prospectively	Venezuela	Unclear/ Not reported	Unclear/ Not reported	Unclear/ Not reported	Unclear/ Not reported	Hemodialysis	Indirect ELISA	Anti-HCV	Unclear/ Not reported
241	Relevance of investigating the presence of hepatitis C virus RNA in HCV antibody-negative hemodialysis patients.	García	2000	Cross-sectional	Non probabilistic	Hospital-based	Multicenter	Urban	Unclear/ Not reported	Prospectively	Spain	Dec/1997-Dec/1998	Unclear/ Not reported	Unclear/ Not reported	Unclear/ Not reported	Hemodialysis	Indirect ELISA	Anti-HCV	Blood
242	Comparison of the enzyme-linked immunosorbant assay III, recombinant immunoblot third generation assay, and polymerase chain reaction method in the detection of hepatitis C virus infection in haemodialysis patients.	Garinis	1999	Cross-sectional	Non probabilistic	Hospital-based	Monocenter	Unclear/ Not reported	Unclear/ Not reported	Prospectively	Greece	Unclear/ Not reported	43.3	Unclear/ Not reported	51.5	Hemodialysis	Classical RT-PCR	Viral RNA	Blood
243	High prevalence of hepatitis G virus (HGV) infections in dialysis staff.	Gärtner	1999	Cross-sectional	Non probabilistic	Hospital-based	Monocenter	Unclear/ Not reported	Unclear/ Not reported	Prospectively	Germany	Unclear/ Not reported	Unclear/ Not reported	Unclear/ Not reported	Unclear/ Not reported	Hemodialysis	Microparticle enzyme immunoassay	Anti-HCV	Blood
244	Epidemiology of hepatitis B and hepatitis C virus infections among hemodialysis patients in Khartoum, Sudan.	Gasim	2012	Cross-sectional	Non probabilistic	Hospital-based	Monocenter	Urban	Unclear/ Not reported	Prospectively	Sudan	2010	46.2	Unclear/ Not reported	67.4	Hemodialysis	Indirect ELISA	Anti-HCV	Blood
245	HCV infection in hemodialyzed patients: incidence and correlation with dialytic age.	Giammaria	1992	Cross-sectional	Non probabilistic	Hospital-based	Multicenter	Unclear/ Not reported	Unclear/ Not reported	Prospectively	Italy	Unclear/ Not reported	Unclear/ Not reported	Children: less 18 years	Unclear/ Not reported	Hemodialysis	Indirect ELISA	Anti-HCV	Blood
246	Non-A, non-B hepatitis and anti-HCV antibodies in dialysis patients.	Gilli	1990	Cross-sectional	Non probabilistic	Hospital-based	Monocenter	Unclear/ Not reported	Unclear/ Not reported	Prospectively	Italy	Unclear/ Not reported	60.4	Unclear/ Not reported	50	Hemodialysis	Indirect ELISA	Anti-HCV	Blood
247	Prevalence of antibodies to hepatitis C virus in patients on peritoneal dialysis--a multicenter study.	Gladziwa	1993	Cross-sectional	Non probabilistic	Hospital-based	Multicenter	Urban	Antiviral naïve	Prospectively	Germany	Unclear/ Not reported	Unclear/ Not reported	Unclear/ Not reported	Unclear/ Not reported	Peritoneal dialysis	Indirect ELISA	Anti-HCV	Blood
248	Prevalence of antibodies to hepatitis C virus in hemodialysis patients and renal transplant recipients.	Gohar	1995	Cross-sectional	Non probabilistic	Hospital-based	Monocenter	Urban	Antiviral naïve	Prospectively	Egypt	Unclear/ Not reported	Unclear/ Not reported	Unclear/ Not reported	Unclear/ Not reported	Hemodialysis	Indirect ELISA	Anti-HCV	Blood
249	[Anti-HCV seropositivity in dialysis patients].	Gomes	2006	Cohort (Baseline data)	Non probabilistic	Hospital-based	Multicenter	Urban	Unclear/ Not reported	Retrospectively	Portugal	Aug/2003-Dec/2003	54.3	All ages	58	Hemodialysis	Unclear/ Not reported	Anti-HCV	Unclear/ Not reported
250	[Hepatitis C viral in patients with terminal chronic kidney failure. I. Prevalence].	González-Michaca	2000	Cross-sectional	Non probabilistic	Hospital-based	Multicenter	Urban	Antiviral naïve	Prospectively	Mexico	Unclear/ Not reported	Unclear/ Not reported	Unclear/ Not reported	Unclear/ Not reported	Hemodialysis; Peritoneal dialysis	Classical RT-PCR	Viral RNA	Blood
251	Prevalence and risk factors for hepatitis C virus infection in continuous ambulatory peritoneal dialysis patients.	Górriz	1996	Cross-sectional	Probabilistic	Hospital-based	Multicenter	Unclear/ Not reported	Unclear/ Not reported	Prospectively	Spain	Unclear/ Not reported	Unclear/ Not reported	All ages	57	Peritoneal dialysis	Indirect ELISA	Anti-HCV	Blood
252	Prevalence of hepatitis C virus antibodies in hemodialysis patients in the area of Milan.	Gubertini	1992	Cross-sectional	Non probabilistic	Hospital-based	Multicenter	Urban	Unclear/ Not reported	Prospectively	Italy	Unclear/ Not reported	Unclear/ Not reported	All ages	Unclear/ Not reported	Hemodialysis	Indirect ELISA	Anti-HCV	Blood
253	Prevalence of hepatitis C in patients on maintenance haemodialysis.	Gul	2003	Cross-sectional	Non probabilistic	Hospital-based	Monocenter	Urban	Antiviral naïve	Prospectively	Pakistan	1999	Unclear/ Not reported	Unclear/ Not reported	Unclear/ Not reported	Hemodialysis	Unclear/ Not reported	Anti-HCV	Unclear/ Not reported
254	Prevalence of hepatitis C infection in hemodialysis patients: Report from 37 hemodialysis centers in Libya	Gusbi	2019	Cross-sectional	Non probabilistic	Hospital-based	Multicenter	Unclear/ Not reported	Unclear/ Not reported	Prospectively	Libya	Unclear/ Not reported	53.40	Unclear/ Not reported	55.8	Hemodialysis	Indirect ELISA	Anti-HCV	Blood
255	Hepatitis C infection in hemodialysis units.	Ha	1990	Cross-sectional	Non probabilistic	Hospital-based	Multicenter	Urban	Antiviral naïve	Prospectively	South Korea	1990	49.1	Unclear/ Not reported	Unclear/ Not reported	Hemodialysis	Unclear/ Not reported	Anti-HCV	Blood
256	[Viral hepatitis C in chronic hemodialyzed patients in southern Tunisia. Prevalence and risk factors].	Hachicha	1995	Cross-sectional	Non probabilistic	Hospital-based	Multicenter	Urban	Antiviral naïve	Prospectively	Tunisia	Unclear/ Not reported	Unclear/ Not reported	Unclear/ Not reported	Unclear/ Not reported	Hemodialysis	Indirect ELISA	Anti-HCV	Unclear/ Not reported

257	Hepatitis B virus subtypes and hepatitis C virus genotypes in patients with chronic liver disease or on maintenance hemodialysis in Indonesia	Hadiwandowo	1994	Cross-sectional	Non probabilistic	Hospital-based	Monocenter	Urban	Antiviral naïve	Prospectively	Indonesia	Unclear/ Not reported	Unclear/ Not reported	Unclear/ Not reported	Unclear/ Not reported	Hemodialysis	Indirect ELISA	Anti-HCV	Blood
258	Prevalence of hepatitis B and hepatitis C in blood donors and high risk groups in Hajjah, Yemen Republic.	Haidar	2002	Cross-sectional	Non probabilistic	Hospital-based	Monocenter	Urban	Antiviral naïve	Prospectively	Yemen	Apr/1997-Sep/1999	Unclear/ Not reported	Unclear/ Not reported	Unclear/ Not reported	Hemodialysis	Indirect ELISA	Anti-HCV	Blood
259	National Epidemiological Study about Hepatitis C Virus Infection among Dialysis Patients.	Hajji	2021	Cross-sectional	Non probabilistic	Hospital-based	Nationally representative	Urban	Antiviral naïve	Prospectively	Tunisia	2019	Unclear/ Not reported	Unclear/ Not reported	Unclear/ Not reported	Hemodialysis; Peritoneal dialysis	Indirect ELISA	Anti-HCV	Unclear/ Not reported
260	Prospective virological follow-up of hepatitis C infection in a haemodialysis unit.	Halfon	1998	Cross-sectional	Non probabilistic	Hospital-based	Multicenter	Unclear/ Not reported	Unclear/ Not reported	Prospectively	France	Unclear/ Not reported	Unclear/ Not reported	All ages	61	Hemodialysis	Indirect ELISA	Anti-HCV	Blood
261	Hepatitis C virus transmission at an outpatient hemodialysis unit--New York, 2001-2008.	Hallack	2009	Cohort (Baseline data)	Non probabilistic	Hospital-based	Monocenter	Unclear/ Not reported	Unclear/ Not reported	Retrospectively	United States of America	2001-2008	Unclear/ Not reported	Unclear/ Not reported	Unclear/ Not reported	Hemodialysis	Indirect ELISA	Anti-HCV	Blood
262	Hepatitis B, Hepatitis C, and Human Immune deficiency Virus Seroconversion Positivity Rates and Their Potential Risk Factors Among Patients on Maintenance Hemodialysis in Cameroon.	Halle	2016	Cross-sectional	Non probabilistic	Hospital-based	Monocenter	Rural	Unclear/ Not reported	Prospectively	Cameroon	Jun/2012-Sep/2012	51	Unclear/ Not reported	66	Hemodialysis	Indirect ELISA	Anti-HCV	Blood
263	Occurrence of hepatitis B and C infection among hemodialyzed patients with chronic renal failure in Qazvin, Iran: A preliminary study	Hamissi	2011	Cross-sectional	Non probabilistic	Hospital-based	Multicenter	Unclear/ Not reported	Unclear/ Not reported	Prospectively	Iran	Jul/2009-Oct/2009	53.62	All ages	61.5	Hemodialysis	Indirect ELISA	Anti-HCV	Blood
264	Hepatitis B virus (HBV) and Hepatitis C virus (HCV) infections among hemodialysis patients at OMTH.	Hammad	2016	Cross-sectional	Non probabilistic	Hospital-based	Monocenter	Unclear/ Not reported	Unclear/ Not reported	Prospectively	Sudan	May/2015-Jun/2015	50	Adults: over 18 years	52	Hemodialysis	Indirect ELISA	Anti-HCV	Blood
265	Hepatitis C virus infection in renal failure patients in the absence of anti-hepatitis C virus antibodies.	Hanuka	2002	Cross-sectional	Non probabilistic	Hospital-based	Monocenter	Unclear/ Not reported	Unclear/ Not reported	Prospectively	Israel	Unclear/ Not reported	Unclear/ Not reported	Unclear/ Not reported	Unclear/ Not reported	Hemodialysis	Indirect ELISA	Anti-HCV	Blood
266	Antibody to hepatitis C virus increases with time on hemodialysis.	Hardy	1992	Cross-sectional	Non probabilistic	Hospital-based	Monocenter	Urban	Antiviral naïve	Prospectively	United States Virgin Islands	Unclear/ Not reported	Unclear/ Not reported	Unclear/ Not reported	Unclear/ Not reported	Hemodialysis	Indirect ELISA	Anti-HCV	Unclear/ Not reported
267	Low prevalence of hepatitis C virus infection in hemodialysis units: effect of isolation?	Harmankaya	2002	Cross-sectional	Non probabilistic	Hospital-based	Monocenter	Unclear/ Not reported	Antiviral naïve	Prospectively	Turkey	Mar/1992-Aug/2000	54	All ages	54.7	Hemodialysis	Indirect ELISA	Anti-HCV	Blood
268	Epidemiological Aspects of Hepatitis B and C and Human Immunodeficiency Viruses Among Hemodialysis Patients in Mazandaran Province, Iran.	Hasanjani Roushan	2016	Cross-sectional	Non probabilistic	Hospital-based	Monocenter	Urban/Rural	Unclear/ Not reported	Prospectively	Iran	2012-2013	51.6	All ages	52.5	Hemodialysis	Unclear/ Not reported	Anti-HCV	Unclear/ Not reported
269	Post-transfusion-transmitted hepatitis C virus infection: a study on thalassemia and hemodialysis patients in southeastern Iran.	Hassanshahi	2011	Cross-sectional	Non probabilistic	Hospital-based	Multicenter	Unclear/ Not reported	Unclear/ Not reported	Prospectively	Iran	Jun/2006-Mar/2007	51.1	All ages	42	Hemodialysis	Classical RT-PCR	Viral RNA	Blood
270	Prevalence of antibody to hepatitis C virus in hemodialysis patients.	Hayashi	1991	Cross-sectional	Non probabilistic	Hospital-based	Multicenter	Urban	Unclear/ Not reported	Prospectively	Japan	Unclear/ Not reported	50.4	All ages	57	Hemodialysis	Indirect ELISA	Anti-HCV	Blood
271	Prevalence and role of hepatitis C viraemia in haemodialysis patients in Japan.	Hayashi	1994	Cross-sectional	Non probabilistic	Hospital-based	Multicenter	Unclear/ Not reported	Unclear/ Not reported	Prospectively	Japan	Unclear/ Not reported	Unclear/ Not reported	Unclear/ Not reported	Unclear/ Not reported	Hemodialysis	Indirect ELISA	Anti-HCV	Blood
272	Seroepidemiology of hepatitis C virus infection in hemodialysis patients and the general population in Fukuoka and Okinawa, Japan.	Hayashi	1994	Cross-sectional	Non probabilistic	Hospital-based	Multicenter	Unclear/ Not reported	Unclear/ Not reported	Prospectively	Japan	Unclear/ Not reported	Unclear/ Not reported	All ages	57.8	Hemodialysis	Passive Hemagglutination assay (PHA)	Anti-HCV	Blood

273	Prevalence and risk factors of hepatitis C virus infection in haemodialysis patients: a multicentre study in 2796 patients.	Hinrichsen	2002	Cross-sectional	Non probabilistic	Hospital-based	Multicenter	Unclear/ Not reported	Unclear/ Not reported	Prospectively	Germany	Oct/1996-Mar/1997	61	All ages	53	Hemodialysis	Indirect ELISA	Anti-HCV	Blood
274	Hepatitis C virus infection among dialysis patients in Tunisia: incidence and molecular evidence for nosocomial transmission.	Hmaied	2006	Cross-sectional	Non probabilistic	Hospital-based	Multicenter	Unclear/ Not reported	Unclear/ Not reported	Prospectively	Tunisia	Nov/2001–May/2002	54	All ages	Unclear/ Not reported	Hemodialysis	Indirect ELISA	Anti-HCV	Blood
275	[HCV antibodies in hemodialyzed patients in Tunisia].	Hmida	1995	Cross-sectional	Non probabilistic	Hospital-based	Multicenter	Urban	Antiviral naïve	Prospectively	Tunisia	Unclear/ Not reported	Unclear/ Not reported	Unclear/ Not reported	Unclear/ Not reported	Hemodialysis	Unclear/ Not reported	Anti-HCV	Blood
276	Intrafamilial transmission of hepatitis C virus in hemodialysis patients.	Hou	1995	Cross-sectional	Non probabilistic	Hospital-based	Multicenter	Unclear/ Not reported	Unclear/ Not reported	Prospectively	China	Unclear/ Not reported	53.4	All ages	53	Hemodialysis	Indirect ELISA	Anti-HCV	Blood
277	High prevalence of antibodies to hepatitis C virus in three haemodialysis centres in south-western Poland.	Hruby	1993	Cross-sectional	Non probabilistic	Hospital-based	Multicenter	Unclear/ Not reported	Unclear/ Not reported	Prospectively	Poland	Unclear/ Not reported	44.7	All ages	43	Hemodialysis	Microparticle enzyme immunoassay	Anti-HCV	Blood
278	TT virus infection in patients on peritoneal dialysis in Taiwan.	Hsu	2007	Cross-sectional	Non probabilistic	Hospital-based	Monocenter	Unclear/ Not reported	Unclear/ Not reported	Prospectively	China	Dec/2004	53.6	Unclear/ Not reported	32	Peritoneal dialysis	Indirect ELISA	Anti-HCV	Blood
279	Hepatitis C markers in hemodialysis patients.	Huang	1993	Cross-sectional	Non probabilistic	Hospital-based	Monocenter	Urban	Antiviral naïve	Prospectively	China	Unclear/ Not reported	52.9	Adults: over 18 years	57	Hemodialysis	Indirect ELISA	Anti-HCV	Blood
280	Hepatitis C virus--does it penetrate the haemodialysis membrane? PCR analysis of haemodialysis ultrafiltrate and whole blood.	Hubmann	1995	Cross-sectional	Non probabilistic	Hospital-based	Monocenter	Urban	Antiviral naïve	Prospectively	Austria	Unclear/ Not reported	53.5	Unclear/ Not reported	58.6	Hemodialysis	Indirect ELISA	Anti-HCV	Unclear/ Not reported
281	High prevalence of and risk factors for hepatitis C in haemodialysis patients in Saudi Arabia: a need for new dialysis strategies.	Huraib	1995	Cross-sectional	Non probabilistic	Hospital-based	Multicenter	Urban	Antiviral naïve	Prospectively	Saudi Arabia	Unclear/ Not reported	43.4	All ages	50.5	Hemodialysis	Indirect ELISA	Anti-HCV	Blood
282	Hepatitis-C and it's seroconversion in end stage kidney disease patients on maintenance hemodialysis and factors affecting it.	Hussain	2019	Cross-sectional	Non probabilistic	Hospital-based	Monocenter	Rural	Antiviral naïve	Prospectively	Pakistan	2017	Unclear/ Not reported	All ages	57.82	Hemodialysis	Unclear/ Not reported	Anti-HCV	Unclear/ Not reported
283	Methods used to reduce the prevalence of hepatitis C in a dialysis unit.	Hussein	2010	Cross-sectional	Non probabilistic	Hospital-based	Monocenter	Urban	Antiviral naïve	Prospectively	Saudi Arabia	Jul/2003-Mar/2009	54.6	All ages	47.7	Hemodialysis	Classical RT-PCR	Viral RNA	Unclear/ Not reported
284	The impact of polymerase chain reaction assays for the detection of hepatitis C virus infection in a hemodialysis unit.	Hussein	2007	Cross-sectional	Non probabilistic	Hospital-based	Monocenter	Urban	Antiviral naïve	Prospectively	Saudi Arabia	2003-2005	54.6	All ages	47.7	Hemodialysis	Microparticle enzyme immunoassay	Anti-HCV	Blood
285	[The determination of the prevalence of antibodies to the hepatitis C virus (anti-HCV) and of the serum levels of C-reactive protein in chronic dialysis patients].	Iancu	1995	Cross-sectional	Non probabilistic	Hospital-based	Monocenter	Urban	Antiviral naïve	Prospectively	Spain	Unclear/ Not reported	Unclear/ Not reported	Unclear/ Not reported	Unclear/ Not reported	Hemodialysis	Unclear/ Not reported	Anti-HCV	Unclear/ Not reported
286	[Hepatitis C virus: results of detection in several high risk groups in the X region of Chile].	Ibarra	1995	Cross-sectional	Non probabilistic	Hospital-based	Multicenter	Urban	Antiviral naïve	Prospectively	Chile	Unclear/ Not reported	Unclear/ Not reported	Unclear/ Not reported	Unclear/ Not reported	Hemodialysis	Indirect ELISA	Anti-HCV	Unclear/ Not reported
287	Hepatitis C Virus Seroconversion Among Hemodialysis Patients and the Role of Hepatitis C Virus Positive Patient's Isolation in Benha, Egypt.	Ibrahim	2017	Cohort (Baseline data)	Non probabilistic	Hospital-based	Monocenter	Urban	Unclear/ Not reported	Prospectively	Egypt	Unclear/ Not reported	54.41	Adults: over 18 years	56.7	Hemodialysis	Indirect ELISA	Anti-HCV	Blood
288	Clinical significance of hepatitis G virus infection in patients on long-term haemodialysis.	Ideura	1997	Cross-sectional	Non probabilistic	Hospital-based	Monocenter	Urban	Antiviral naïve	Prospectively	Japan	1995	57.9	All ages	56.4	Hemodialysis	Indirect ELISA	Anti-HCV	Blood
289	Superinfection of TT virus and hepatitis C virus among chronic haemodialysis patients.	Ikeuchi	1999	Cross-sectional	Non probabilistic	Hospital-based	Monocenter	Urban	Antiviral naïve	Prospectively	Japan	1995	57.9	All ages	56.4	Hemodialysis	Indirect ELISA	Anti-HCV	Blood
290	[Hepatitis B virus markers and anti-HCV antibodies in	Illés	1992	Cross-sectional	Non probabilistic	Hospital-based	Monocenter	Urban	Antiviral naïve	Prospectively	Hungary	Unclear/ Not reported	Unclear/ Not reported	Unclear/ Not reported	Unclear/ Not reported	Hemodialysis	Indirect ELISA	Anti-HCV	Blood

	hemodialyzed patients].																		
291	Comparison of anti-hepatitis C virus detection with ELISA assay and RIBA 4 in dialysis patients: our experience.	Innocenti	1992	Cross-sectional	Non probabilistic	Hospital-based	Monocenter	Urban	Antiviral naïve	Prospectively	Italy	Unclear/ Not reported	Unclear/ Not reported	Unclear/ Not reported	Unclear/ Not reported	Hemodialysis	Indirect ELISA	Anti-HCV	Blood
292	HCV incidence in a dialysis center: preliminary reports.	Ippolito	1992	Cross-sectional	Non probabilistic	Hospital-based	Monocenter	Urban	Antiviral naïve	Prospectively	Italy	Dec/1989-May/1991	Unclear/ Not reported	Adults: over 18 years	Unclear/ Not reported	Hemodialysis	Indirect ELISA	Anti-HCV	Blood
293	Hepatitis C infection unrelated to blood transfusion in hemodialysis patients.	Irie	1994	Cross-sectional	Non probabilistic	Hospital-based	Monocenter	Urban	Antiviral naïve	Prospectively	Japan	Jul/1995	Unclear/ Not reported	All ages	Unclear/ Not reported	Hemodialysis	Indirect ELISA	Anti-HCV	Blood
294	Identification of hepatitis C virus seroconversion resulting from nosocomial transmission on a haemodialysis unit: implications for infection control and laboratory screening.	Irish	1999	Cross-sectional	Non probabilistic	Hospital-based	Multicenter	Rural	Antiviral naïve/ On antiviral	Prospectively	United Kingdom	1996	Unclear/ Not reported	Unclear/ Not reported	Unclear/ Not reported	Hemodialysis	Classical RT-PCR	Viral RNA	Blood
295	Torque teno virus infection in hemodialysis patients in North India.	Irshad	2010	Cross-sectional	Non probabilistic	Hospital-based	Monocenter	Urban	Antiviral naïve	Prospectively	India	May/2007-Apr/2009	43	Unclear/ Not reported	77	Hemodialysis	Indirect ELISA	Anti-HCV	Blood
296	HCV infection in Delhi, India.	Irshad	1998	Cross-sectional	Non probabilistic	Hospital-based	Monocenter	Urban	Unclear/ Not reported	Prospectively	India	1992-1996	Unclear/ Not reported	Unclear/ Not reported	Unclear/ Not reported	Hemodialysis	Indirect ELISA	Anti-HCV	Blood
297	Viral hepatitis in multiple blood transfused patients treated at a referral hospital of Delhi, India.	Irshad	2002	Cross-sectional	Non probabilistic	Hospital-based	Monocenter	Urban	Unclear/ Not reported	Prospectively	India	Unclear/ Not reported	Unclear/ Not reported	Unclear/ Not reported	Unclear/ Not reported	Hemodialysis	Unclear/ Not reported	Anti-HCV	Blood
298	Epidemiology update for hepatitis C virus and hepatitis B virus in end-stage renal disease in France.	Isnard Bagnis	2017	Cross-sectional	Probabilistic	Hospital-based	Nationally representative	Urban/Rural	Unclear/ Not reported	Prospectively	France	Jan/2005-Dec/2013	67	Unclear/ Not reported	62.5	Hemodialysis	Unclear/ Not reported	Viral RNA	Unclear/ Not reported
299	Hepatitis C virus infection in hemodialysis patients: First-generation enzyme immunoassay.	Ivankovic	1994	Cross-sectional	Non probabilistic	Hospital-based	Monocenter	Urban	Antiviral naïve	Prospectively	Croatia	Unclear/ Not reported	Unclear/ Not reported	Unclear/ Not reported	Unclear/ Not reported	Hemodialysis	Indirect ELISA	Anti-HCV	Blood
300	Patterns in the prevalence of hepatitis C virus infection at the start of hemodialysis in Japan.	Iwasa	2008	Cross-sectional	Non probabilistic	Hospital-based	Monocenter	Urban	Antiviral naïve	Prospectively	Japan	Feb/2003-Jun/2007	61	Adults: over 18 years	67.7	Hemodialysis	Unclear/ Not reported	Anti-HCV	Unclear/ Not reported
301	Occasional infection of hepatitis C virus occurring in haemodialysis units identified by serial monitoring of the virus infection.	Iwasaki	2000	Cross-sectional	Non probabilistic	Hospital-based	Monocenter	Urban	Unclear/ Not reported	Prospectively	Japan	1992-1997	57.55	Adults: over 18 years	54.22	Hemodialysis	Classical RT-PCR	Viral RNA	Blood
302	Molecular evidence for nosocomial transmission of hepatitis C virus in a French hemodialysis unit.	Izopet	1999	Cross-sectional	Non probabilistic	Hospital-based	Monocenter	Urban	Antiviral naïve	Prospectively	France	1994-1995	Unclear/ Not reported	Unclear/ Not reported	Unclear/ Not reported	Hemodialysis	Indirect ELISA	Anti-HCV	Blood
303	Incidence of HCV infection in French hemodialysis units: a prospective study.	Izopet	2005	Cross-sectional	Non probabilistic	Hospital-based	Multicenter	Urban	Antiviral naïve	Prospectively	France	1997-2000	Unclear/ Not reported	Unclear/ Not reported	Unclear/ Not reported	Hemodialysis	Indirect ELISA	Anti-HCV	Blood
304	Prevalence and predisposing factors for hepatitis C virus in haemodialysis unit universiti kebangsaan malaysia medical centre.	Jaafar	2011	Cross-sectional	Non probabilistic	Hospital-based	Multicenter	Urban	Antiviral naïve	Prospectively	Malaysia	Jun/2009-May/2010	Unclear/ Not reported	Unclear/ Not reported	Unclear/ Not reported	Hemodialysis	Classical RT-PCR	Viral RNA	Blood
305	Hepatitis C in hemodialysis centers of golestan province, northeast of Iran (2005).	Jabbari	2008	Cross-sectional	Non probabilistic	Hospital-based	Multicenter	Rural	Antiviral naïve	Prospectively	Iran	2005	47.3	Unclear/ Not reported	54.8	Hemodialysis	Indirect ELISA	Anti-HCV	Blood
306	Incidence and risk factors for hepatitis C seroconversion in hemodialysis: A prospective study.	Jadoul	1993	Cross-sectional	Non probabilistic	Hospital-based	Multicenter	Urban	Antiviral naïve	Prospectively	Belgium	May/1991-Nov/1992	61	All ages	Unclear/ Not reported	Hemodialysis	Indirect ELISA	Anti-HCV	Blood
307	Occult hepatitis C virus infection is more common than hepatitis B infection in maintenance hemodialysis patients.	Jain	2008	Cross-sectional	Non probabilistic	Hospital-based	Monocenter	Urban	Unclear/ Not reported	Prospectively	India	Jun/2006-Jun/2007	41.4	Adults: over 18 years	66.67	Hemodialysis	Classical RT-PCR	Viral RNA	Blood
308	Prevalence of hepatitis viruses among chronic renal failure patients on hemodialysis in central India.	jaiswal	2002	Cross-sectional	Non probabilistic	Hospital-based	Monocenter	Urban	Antiviral naïve	Prospectively	India	1992-2000	Unclear/ Not reported	Unclear/ Not reported	Unclear/ Not reported	Hemodialysis	Unclear/ Not reported	Anti-HCV	Unclear/ Not reported

309	A very high prevalence of hepatitis C virus infection among patients undergoing hemodialysis in Kosovo: a nationwide study.	Jakupi	2018	Cross-sectional	Probabilistic	Hospital-based	Nationally representative	Urban	Unclear/ Not reported	Prospectively	Kosovo	Jan/2013-Mar/2013	56.3	Unclear/ Not reported	47.8	Hemodialysis	Indirect ELISA	Anti-HCV	Blood
310	Prevalence of Hepatitis C Infection and its Genotypes in Suspected Hemodialysis Patients, Southwest of Iran.	Jamalidoust	2021	Cross-sectional	Non probabilistic	Hospital-based	Monocenter	Urban	Antiviral naïve	Prospectively	Iran	2011-2018	46.26	Unclear/ Not reported	63.4	Hemodialysis	Indirect ELISA	Anti-HCV	Unclear/ Not reported
311	Hepatitis C and hepatitis B virus infection in hemodialysis patients and staff: a two year follow-up.	Jankovic	1994	Cross-sectional	Non probabilistic	Hospital-based	Monocenter	Urban	Antiviral naïve	Prospectively	Croatia	May/1991-Apr/1993	49	Unclear/ Not reported	57	Hemodialysis	Indirect ELISA	Anti-HCV	Blood
312	Prevalence and associations of hepatitis C viremia in hemodialysis patients at a tertiary care hospital.	Jasuja	2009	Cross-sectional	Non probabilistic	Hospital-based	Monocenter	Urban	Unclear/ Not reported	Prospectively	India	Unclear/ Not reported	Unclear/ Not reported	Unclear/ Not reported	64.7	Hemodialysis	Classical RT-PCR	Viral RNA	Blood
313	Prevalence and Risk Factors Associated with Hepatitis B and Hepatitis C Infections among Patients Undergoing Hemodialysis: A Single-Centre Study in Somalia.	jeele	2021	Cross-sectional	Non probabilistic	Hospital-based	Monocenter	Urban	Unclear/ Not reported	Prospectively	Somalia	Jan/2021-Jun/2021	52.7	Unclear/ Not reported	51.4	Hemodialysis	Indirect ELISA	Anti-HCV	Blood
314	Hepatitis C infection in two urban hemodialysis units.	Jeffers	1990	Cross-sectional	Non probabilistic	Hospital-based	Multicenter	Urban	Unclear/ Not reported	Prospectively	United States of America	Mar/1989-Apr/1989	54	Unclear/ Not reported	75.6	Hemodialysis	Unclear/ Not reported	Anti-HCV	Unclear/ Not reported
315	Seropositivity to hepatitis C virus in Tunisian haemodialysis patients.	Jemni	1994	Cross-sectional	Non probabilistic	Hospital-based	Monocenter	Urban	Unclear/ Not reported	Prospectively	Tunisia	Unclear/ Not reported	Unclear/ Not reported	Unclear/ Not reported	Unclear/ Not reported	Hemodialysis	Indirect ELISA	Anti-HCV	Unclear/ Not reported
316	Hepatitis C Virus (HCV) Infection among Seronegative Patients undergoing Haemodialysis in a Remotely Located Tertiary Care Hospital of Northern India: Value of HCV-RNA and Genotypes.	Jindal	2015	Cross-sectional	Non probabilistic	Hospital-based	Monocenter	Urban	Unclear/ Not reported	Prospectively	India	Mar/2012-Jun/2013	Unclear/ Not reported	Unclear/ Not reported	Unclear/ Not reported	Hemodialysis	Indirect ELISA	Anti-HCV	Blood
317	HCV infection among healthy blood donors and risk groups in north India.	Jindal	2009	Cross-sectional	Non probabilistic	Hospital-based	Monocenter	Urban	Unclear/ Not reported	Prospectively	India	2004–2005	Unclear/ Not reported	Unclear/ Not reported	Unclear/ Not reported	Hemodialysis	Indirect ELISA	Anti-HCV	Blood
318	Hepatitis C infection in a pediatric dialysis population.	Jonas	1992	Cross-sectional	Non probabilistic	Hospital-based	Multicenter	Unclear/ Not reported	Unclear/ Not reported	Prospectively	United States of America	Unclear/ Not reported	20.9	All ages	44.4	Hemodialysis	Indirect ELISA	Anti-HCV	Blood
319	Hepatitis C and hepatitis B seroprevalence and associated risk factors in hemodialysis patients in Guilan province, north of Iran: HCV and HBV seroprevalence in hemodialysis patients.	Joukar	2011	Cross-sectional	Non probabilistic	Hospital-based	Multicenter	Urban	Unclear/ Not reported	Prospectively	Iran	May/2009-Sep/2009	54.8	All ages	55.6	Hemodialysis	Indirect ELISA	Anti-HCV	Blood
320	Occult hepatitis B infection in a hemodialysis population in Guilan Province, Northern Iran.	Joukar	2012	Cross-sectional	Non probabilistic	Hospital-based	Multicenter	Urban	Unclear/ Not reported	Prospectively	Iran	May/2009-Sep/2009	Unclear/ Not reported	Unclear/ Not reported	Unclear/ Not reported	Hemodialysis	Indirect ELISA	Anti-HCV	Blood
321	Prevalence of hepatitis B and C viruses infections among hemodialysis patients in Addis Ababa, Ethiopia.	Juhar	2018	Cross-sectional	Non probabilistic	Hospital-based	Multicenter	Urban/Rural	Unclear/ Not reported	Prospectively	Ethiopia	Apr/2016-May/2016	48.94	Adults: over 18 years	74.3	Hemodialysis	Chemiluminescent immunoassay	Anti-HCV	Blood
322	[Anti-HCV antibodies among hemodialysed and kidney-transplanted patients].	Juszczyk	1994	Cross-sectional	Non probabilistic	Hospital-based	Multicenter	Unclear/ Not reported	Unclear/ Not reported	Prospectively	Poland	Unclear/ Not reported	Unclear/ Not reported	Unclear/ Not reported	Unclear/ Not reported	Hemodialysis	Indirect ELISA	Anti-HCV	Blood
323	Prevalence and risk factors of hepatitis B and C viruses among hemodialysis patients in Isfahan, Iran.	KALANTARI	2014	Cross-sectional	Probabilistic	Hospital-based	Multicenter	Urban	Antiviral naïve	Prospectively	Iran	Oct/2010-Mar/2011	52.3	Adults: over 18 years	60.7	Hemodialysis	Indirect ELISA	Anti-HCV	Blood
324	Clinical characteristics and mortality in hepatitis C positive haemodialysis patients: a population based study.	Kalantar-Zadeh	2005	Cohort (Baseline data)	Non probabilistic	Hospital-based	Nationally representative	Unclear/ Not reported	Unclear/ Not reported	Prospectively	United States of America	July/2001-Sep/2001	60.74	Unclear/ Not reported	53	Hemodialysis	Enzyme immunoassay (EIA)	Anti-HCV	Blood

325	Circulation of an atypical hepatitis C virus (HCV) strain at a dialysis unit in northeast India.	kalita	2021	Cross-sectional	Non probabilistic	Hospital-based	Monocenter	Urban	Unclear/ Not reported	Prospectively	India	Jan/2016-Dec/2016	51	Adults: over 18 years	87.2	Hemodialysis	Real-time RT-PCR	Viral RNA	Blood
326	Laboratory Evaluation of Hepatitis C Virus Infection in Patients Undergoing Hemodialysis from North East India.	Kalita	2022	Cross-sectional	Non probabilistic	Hospital-based	Monocenter	Urban	Antiviral naïve	Prospectively	India	Unclear/ Not reported	Unclear/ Not reported	Adults: over 18 years	Unclear/ Not reported	Hemodialysis	Real-time RT-PCR	Viral RNA	Blood
327	Significance of hepatitis B, hepatitis C and GBV-C in ANCA-positive hemodialysis patients.	Kallinowski	1997	Cross-sectional	Non probabilistic	Hospital-based	Monocenter	Urban	Antiviral naïve	Prospectively	Germany	Unclear/ Not reported	64	Unclear/ Not reported	52	Hemodialysis	Classical RT-PCR	Viral RNA	Blood
328	Prevalence of antibodies to hepatitis C virus in hemodialysis patients.	Kallinowski	1991	Cross-sectional	Non probabilistic	Hospital-based	Monocenter	Urban	Unclear/ Not reported	Prospectively	Germany	Unclear/ Not reported	56	Unclear/ Not reported	51.1	Hemodialysis	Indirect ELISA	Anti-HCV	Blood
329	Seroprevalence occurrence of viral hepatitis and HIV among hemodialysis patients.	kamal	2018	Cross-sectional	Non probabilistic	Hospital-based	Monocenter	Urban	Unclear/ Not reported	Retrospectively	Iraq	Jan/2015-Jan/2-18	Unclear/ Not reported	All ages	62.6	Hemodialysis	Indirect ELISA	Anti-HCV	Blood
330	Seroprevalence of human immunodeficiency virus, hepatitis B virus, and hepatitis C virus among hemodialysis patients in a Tertiary Care Teaching Hospital in a developing country	Kansay	2019	Cross-sectional	Non probabilistic	Hospital-based	Monocenter	Unclear/ Not reported	Unclear/ Not reported	Prospectively	India	Jan/2011-Dec/2015	50.45	Adults: over 18 years	64.7	Hemodialysis	Rapid Diagnostic test	Anti-HCV	Blood
331	Is dialysis environment more important than blood transfusion in transmission of hepatitis C virus during hemodialysis?	kapoor	1993	Cross-sectional	Non probabilistic	Hospital-based	Monocenter	Urban	Antiviral naïve	Prospectively	Kuwait	1992	Unclear/ Not reported	Unclear/ Not reported	Unclear/ Not reported	Hemodialysis	Indirect ELISA	Anti-HCV	Blood
332	Seroprevalence and risk factors of HCV in dialysis patients in a university hemodialysis, center of southeast Anatolia, Turkey.	kara	2001	Cohort (Baseline data)	Non probabilistic	Hospital-based	Monocenter	Urban	Unclear/ Not reported	Prospectively	Turkey	Jan/1999-Dec/1999	44.6	Unclear/ Not reported	56.7	Hemodialysis; Peritoneal dialysis	Indirect ELISA	Anti-HCV	Blood
333	Hepatitis C and G Virus Infection Prevalence Among Hemodialysis Patients and Associated Risk Factors in the Hormozgan Province of Southern Iran.	Kargar Kheirabad	2016	Cross-sectional	Probabilistic	Hospital-based	Monocenter	Urban	Unclear/ Not reported	Prospectively	Iran	Jan/2015-Mar/2015	56.23	Adults: over 18 years	61.7	Hemodialysis	Real-time RT-PCR	Viral RNA	Blood
334	Hepatitis C Virus Among Hemodialysis Patients in Najran: Prevalence is More Among Multi-Center Visitors.	Kashem	2003	Cross-sectional	Non probabilistic	Hospital-based	Multicenter	Urban	Antiviral naïve/ On antiviral	Prospectively	Saudi Arabia	2002	45	Adults: over 18 years	52.2	Hemodialysis	Indirect ELISA	Anti-HCV	Blood
335	Incidence and Risk Factors for Hepatitis C Virus and Hepatitis B Virus Seroconversion in End-Stage Renal Failure Patients on Maintenance Hemodialysis.	Kataruka	2020	Cohort (Baseline data)	Non probabilistic	Hospital-based	Monocenter	Urban	Unclear/ Not reported	Prospectively	India	Jan/2015-Oct/2015	Unclear/ Not reported	Unclear/ Not reported	Unclear/ Not reported	Hemodialysis	Real-time RT-PCR	Viral RNA	Blood
336	Association of HCV core antigen seropositivity with long-term mortality in patients on regular hemodialysis.	Kato	2012	Cross-sectional	Non probabilistic	Hospital-based	Multicenter	Urban	Antiviral naïve/ On antiviral	Prospectively	Japan	Jun/2002	62	Adults: over 18 years	67.2	Hemodialysis	Chemiluminescent immunoassay	Anti-HCV	Blood
337	Quantitation and genotyping of hepatitis C virus RNA in sera of hemodialysis and AIDS patients.	Kessler	1996	Cross-sectional	Non probabilistic	Hospital-based	Monocenter	Urban	Unclear/ Not reported	Prospectively	Australia	Unclear/ Not reported	54.2	Adults: over 18 years	Unclear/ Not reported	Hemodialysis	Indirect ELISA	Anti-HCV	Blood
338	Risk factors for HCV infection in two haemodialysis units in The Netherlands.	Keur	1997	Cross-sectional	Non probabilistic	Hospital-based	Multicenter	Urban	Unclear/ Not reported	Retrospectively	Netherlands	Dec/1991-Jan/1992	51.4	Unclear/ Not reported	61.4	Hemodialysis	Indirect ELISA	Anti-HCV	Blood
339	Evaluation of diagnostic value of ELISA method (EIA) & PCR in diagnosis of hepatitis C virus in hemodialysis patients.	Khadem-Ansari	2006	Cross-sectional	Non probabilistic	Hospital-based	Monocenter	Urban	Unclear/ Not reported	Prospectively	Azerbaijan	2005-2006	Unclear/ Not reported	Unclear/ Not reported	Unclear/ Not reported	Hemodialysis	Enzyme immunoassay (EIA)	Anti-HCV	Blood
340	High prevalence of hepatitis C virus infection and genotype distribution among general population,	Khaja	2006	Cross-sectional	Non probabilistic	Hospital-based	Multicenter	Unclear/ Not reported	Unclear/ Not reported	Prospectively	India	Unclear/ Not reported	Unclear/ Not reported	Unclear/ Not reported	Unclear/ Not reported	Hemodialysis	Classical RT-PCR	Viral RNA	Blood

	blood donors and risk groups.																		
341	Prevalence of hepatitis B and C markers in patients on maintenance hemodialysis in Najran.	Khan	2001	Cross-sectional	Non probabilistic	Hospital-based	Monocenter	Urban	Unclear/ Not reported	Prospectively	Saudi Arabia	Jun/2000	23.6	All ages	53.6	Hemodialysis	Indirect ELISA	Anti-HCV	Blood
342	Rising burden of Hepatitis C Virus in hemodialysis patients.	Khan	2011	Cross-sectional	Probabilistic	Hospital-based	Multicenter	Urban	Unclear/ Not reported	Prospectively	Pakistan	Jan/2010-Apr/2010	40.9	All ages	63.5	Hemodialysis	Indirect ELISA	Anti-HCV	Blood
343	Prevalence and risk factors for hepatitis C virus infection in hemodialysis patients in an Iraqi renal transplant center.	Khattab	2008	Cross-sectional	Non probabilistic	Hospital-based	Monocenter	Urban	Unclear/ Not reported	Prospectively	Iraq	Sep/2003-Sep/2005	36	All ages	60.4	Hemodialysis	Indirect ELISA	Anti-HCV	Blood
344	Presence and significance of transfusion-transmitted virus infection in Iranian patients on maintenance hemodialysis.	Kheradpezhouh	2013	Cross-sectional	Non probabilistic	Hospital-based	Multicenter	Urban	Unclear/ Not reported	Prospectively	Iran	Mar/2005	Unclear/ Not reported	Unclear/ Not reported	Unclear/ Not reported	Hemodialysis	Unclear/ Not reported	Anti-HCV	Blood
345	Prevalence of HCV infections among hemodialysis patients in Al Gharbiyah Governorate, Egypt.	Khodir	2012	Cohort (Baseline data)	Non probabilistic	Hospital-based	Multicenter	Urban	Unclear/ Not reported	Prospectively	Egypt	Apr/2011-Nov/2011	52	All ages	61	Hemodialysis	Microparticle enzyme immunoassay	Anti-HCV	Blood
346	Risk factors for hepatitis C virus infection in patients on long-term hemodialysis.	Khokhar	2005	Cross-sectional	Non probabilistic	Hospital-based	Monocenter	Urban	Unclear/ Not reported	Retrospectively	Pakistan	Jan/2002-Jun/2003	55	Unclear/ Not reported	Unclear/ Not reported	Hemodialysis	Chemiluminescent immunoassay	Anti-HCV	Blood
347	HCV-RNA Pcr Positivity In Hcv Antibody Negative Patients Undergoing Haemodialysis.	Kiani	2018	Cross-sectional	Non probabilistic	Hospital-based	Monocenter	Urban	Antiviral naïve	Prospectively	Pakistan	2016	49.7	Unclear/ Not reported	56.5	Hemodialysis	Indirect ELISA	Anti-HCV	Blood
348	Prevalence of hepatitis E virus infection in regular hemodialysis patients.	Kikuchi	2006	Cross-sectional	Probabilistic	Hospital-based	Multicenter	Urban	Unclear/ Not reported	Prospectively	Japan	Unclear/ Not reported	60.1	Unclear/ Not reported	62.7	Hemodialysis	Chemiluminescent immunoassay	Anti-HCV	Blood
349	Hepatitis C virus genotypes in chronic hemodialysis patients.	Kiliç	2000	Cross-sectional	Non probabilistic	Hospital-based	Monocenter	Urban	Unclear/ Not reported	Prospectively	Turkey	Unclear/ Not reported	40.6	Unclear/ Not reported	56.6	Hemodialysis	Chemiluminescent immunoassay	Anti-HCV	Blood
350	Hepatitis C in dialysis patients: relationship to blood transfusions, dialysis and liver disease.	Knudsen	1993	Cross-sectional	Non probabilistic	Hospital-based	Multicenter	Urban	Unclear/ Not reported	Prospectively	Denmark	Oct/1990-Mar/1991	54	All ages	61	Hemodialysis	Indirect ELISA	Anti-HCV	Blood
351	Eighteen-year follow-up cohort study on hepatitis B and C virus infections related long-term prognosis among hemodialysis patients in Hiroshima.	Ko	2020	Cross-sectional	Non probabilistic	Hospital-based	Multicenter	Urban	Unclear/ Not reported	Prospectively	Japan	1999-2017	Unclear/ Not reported	Unclear/ Not reported	60.4	Hemodialysis	Chemiluminescent immunoassay	Anti-HCV	Blood
352	Prospective follow-up study of hepatitis C virus infection in patients undergoing maintenance haemodialysis: comparison among haemodialysis units.	Kobayashi	1998	Cohort (Baseline data)	Non probabilistic	Hospital-based	Multicenter	Urban	Unclear/ Not reported	Prospectively	Japan	Apr/1990-May/1995	54.7	Adults: over 18 years	69.9	Hemodialysis	Indirect ELISA	Anti-HCV	Blood
353	Detection of hepatitis B and C infection by polymerase chain reaction among hemodialysis patients.	Kocabaş	2002	Cross-sectional	Non probabilistic	Hospital-based	Monocenter	Urban	Unclear/ Not reported	Prospectively	Turkey	Unclear/ Not reported	40.9	All ages	62.3	Hemodialysis	Classical RT-PCR	Viral RNA	Blood
354	[Studies on hepatitis C virus infection in haemodialysis patients].	Koda	1992	Cross-sectional	Non probabilistic	Hospital-based	Monocenter	Urban	Unclear/ Not reported	Prospectively	Japan	Unclear/ Not reported	52.2	Unclear/ Not reported	59	Hemodialysis	Indirect ELISA	Anti-HCV	Blood
355	Hepatitis C antibodies in dialysis patients and patients with leukaemia.	Kolho	1993	Cross-sectional	Non probabilistic	Hospital-based	Monocenter	Urban	Unclear/ Not reported	Prospectively	Finland	May/1991	51	Adults: over 18 years	56.1	Hemodialysis; Peritoneal dialysis	Indirect ELISA	Anti-HCV	Blood
356	Factors associated with the severity of pruritus in patients with terminal chronic kidney disease undergoing hemodialysis in Lima, Peru.	Kossuth-Cabrejos	2020	Cross-sectional	Non probabilistic	Hospital-based	Monocenter	Unclear/ Not reported	Unclear/ Not reported	Prospectively	Peru	Jul/2017-Aug/2017	52.17	Unclear/ Not reported	59.9	Hemodialysis	Unclear/ Not reported	Anti-HCV	Blood
357	Detection of hepatitis C virus RNA in hemodialysis patients.	Kuhns	1994	Cross-sectional	Non probabilistic	Hospital-based	Monocenter	Urban	Unclear/ Not reported	Prospectively	United States of America	Apr/1990-May/1990	55	Adults: over 18 years	77.8	Hemodialysis	Indirect ELISA	Anti-HCV	Blood
358	A cross-sectional study of dialysis practice-patterns in patients with chronic kidney disease on	Kulkarni	2015	Cross-sectional	Non probabilistic	Hospital-based	Multicenter	Urban	Unclear/ Not reported	Prospectively	India	Apr/2012-Jun/2012	47.2	Unclear/ Not reported	68	Hemodialysis	Unclear/ Not reported	Anti-HCV	Unclear/ Not reported

	maintenance hemodialysis.																		
359	Hepatitis G virus infection in hemodialysis patients from urban Delhi.	Kumar	2005	Cross-sectional	Non probabilistic	Hospital-based	Monocenter	Urban	Unclear/ Not reported	Prospectively	India	Unclear/ Not reported	Unclear/ Not reported	Unclear/ Not reported	Unclear/ Not reported	Hemodialysis	Indirect ELISA	Anti-HCV	Blood
360	Hepatitis-C virus antibodies (anti HCV) in haemodialyzed vs non-dialyzed patients.	Kumar	1994	Cross-sectional	Non probabilistic	Hospital-based	Monocenter	Urban	Unclear/ Not reported	Prospectively	Pakistan	Mar/1992-Sep/1992	Unclear/ Not reported	Unclear/ Not reported	Unclear/ Not reported	Hemodialysis	Indirect ELISA	Anti-HCV	Blood
361	Prevalence and risk factors of Hepatitis C among maintenance hemodialysis patients at a tertiary-care hospital in Coimbatore, India.	Kumar	2011	Cross-sectional	Non probabilistic	Hospital-based	Monocenter	Unclear/ Not reported	Unclear/ Not reported	Prospectively	India	Unclear/ Not reported	45	Adults: over 18 years	70.3	Hemodialysis	Chemiluminescent immunoassay	Anti-HCV	Blood
362	Differential effect of viral hepatitis infection on mortality among Korean maintenance dialysis patients: a prospective multicenter cohort study	Kwon	2015	Cohort (Baseline data)	Non probabilistic	Hospital-based	Multicenter	Unclear/ Not reported	Unclear/ Not reported	Prospectively	South Korea	Unclear/ Not reported	54.71	Unclear/ Not reported	57.91	Hemodialysis; Peritoneal dialysis	Chemiluminescent immunoassay	Anti-HCV	Blood
363	Evaluation of commercially available third-generation anti-hepatitis C virus enzyme-linked immunosorbent assay in patients on haemodialysis.	Lakshmi	2007	Cross-sectional	Non probabilistic	Hospital-based	Monocenter	Unclear/ Not reported	Unclear/ Not reported	Prospectively	India	May/2003-Dec/2003	Unclear/ Not reported	Unclear/ Not reported	Unclear/ Not reported	Hemodialysis	Indirect ELISA	Anti-HCV	Blood
364	Nosocomal transmission of hepatitis C viruses (HCV) in a hemodialysis unit: Molecular evidence.	Lampe	2018	Cross-sectional	Non probabilistic	Hospital-based	Monocenter	Urban	Unclear/ Not reported	Prospectively	Brazil	Dec/2016-Mar/2017	Unclear/ Not reported	Unclear/ Not reported	Unclear/ Not reported	Hemodialysis	Real-time RT-PCR	Viral RNA	Unclear/ Not reported
365	Molecular analysis and patterns of ALT and hepatitis C virus seroconversion in haemodialysis patients with acute hepatitis.	Lampe	2008	Cohort (Baseline data)	Non probabilistic	Hospital-based	Monocenter	Urban	Unclear/ Not reported	Prospectively	Brazil	Unclear/ Not reported	Unclear/ Not reported	Unclear/ Not reported	Unclear/ Not reported	Hemodialysis	Indirect ELISA	Anti-HCV	Blood
366	Molecular epidemiology of a hepatitis C virus epidemic in a haemodialysis unit: outbreak investigation and infection outcome.	Lanini	2010	Cohort (Baseline data)	Non probabilistic	Hospital-based	Monocenter	Urban	Unclear/ Not reported	Retrospectively	Italy	Feb/2005-Sep/2005	63	Unclear/ Not reported	72	Hemodialysis	Indirect ELISA	Anti-HCV	Blood
367	Significance of anti-E2 in the diagnosis of HCV infection in patients on maintenance hemodialysis: anti-E2 is frequently detected among anti-HCV antibody-negative patients.	Lee	1996	Cross-sectional	Non probabilistic	Hospital-based	Monocenter	Urban	Unclear/ Not reported	Retrospectively	South Korea	Unclear/ Not reported	Unclear/ Not reported	Unclear/ Not reported	Unclear/ Not reported	Hemodialysis	Indirect ELISA	Anti-HCV	Blood
368	Hepatitis C antibodies in patients on peritoneal dialysis: prevalence and risk factors.	Lee	1996	Cross-sectional	Non probabilistic	Hospital-based	Monocenter	Urban	Unclear/ Not reported	Prospectively	Singapore	Unclear/ Not reported	Unclear/ Not reported	Unclear/ Not reported	Unclear/ Not reported	Peritoneal dialysis	Indirect ELISA	Anti-HCV	Blood
369	Comparative study of hepatitis C virus antibody between hemodialysis and continuous ambulatory peritoneal dialysis patients.	Lee	1993	Cross-sectional	Non probabilistic	Hospital-based	Monocenter	Urban	Unclear/ Not reported	Prospectively	South Korea	Jul/1992-Aug.1992	46.9	Unclear/ Not reported	60.14	Hemodialysis; Peritoneal dialysis	Indirect ELISA	Anti-HCV	Blood
370	Seroepidemiology of hepatitis C virus infection in Taiwan.	Lee	1991	Cross-sectional	Non probabilistic	Hospital-based	Monocenter	Urban	Unclear/ Not reported	Prospectively	China	1990	52.7	Adults: over 18 years	58.3	Hemodialysis	Indirect ELISA	Anti-HCV	Blood
371	Hepatitis C viral infection in a Chinese hemodialysis unit.	Li	2010	Cross-sectional	Non probabilistic	Hospital-based	Monocenter	Urban	Unclear/ Not reported	Prospectively	China	May/2009	53.1	Adults: over 18 years	47.9	Hemodialysis	Indirect ELISA	Anti-HCV	Blood
372	Hepatitis C virus core antigen in virological monitoring of dialysis patients.	Li Cavoli	2010	Cross-sectional	Non probabilistic	Hospital-based	Monocenter	Urban	Unclear/ Not reported	Prospectively	Italy	Sep/2009-Feb/2010	68.6	Unclear/ Not reported	58.7	Hemodialysis; Peritoneal dialysis	Chemiluminescent immunoassay	Anti-HCV	Blood
373	High incidence of hepatitis C virus infection in hemodialysis patients in Taiwan.	Lin	1993	Cohort (Baseline data)	Non probabilistic	Hospital-based	Monocenter	Urban	Unclear/ Not reported	Prospectively	China	Unclear/ Not reported	45	All ages	61.4	Hemodialysis	Indirect ELISA	Anti-HCV	Blood
374	Prevalence of antibodies to hepatitis C virus in the hemodialysis unit.	Lin	1991	Cross-sectional	Non probabilistic	Hospital-based	Monocenter	Urban	Unclear/ Not reported	Prospectively	China	Unclear/ Not reported	45.9	Unclear/ Not reported	54.4	Hemodialysis	Indirect ELISA	Anti-HCV	Blood
375	Viral hepatitis C and B among dialysis patients at the Rabat	Lioussfi	2014	Cross-sectional	Non probabilistic	Hospital-based	Monocenter	Urban	Unclear/ Not reported	Prospectively	Morocco	Jul/2009	44.8	Unclear/ Not reported	52.4	Hemodialysis; Peritoneal dialysis	Indirect ELISA	Anti-HCV	Blood

	University Hospital: prevalence and risk factors.																		
376	Profile and predictors of hepatitis and HIV infection in patients on hemodialysis of Quetta, Pakistan.	Lodhi	2019	Cross-sectional	Non probabilistic	Hospital-based	Multicenter	Urban	Unclear/ Not reported	Prospectively	Pakistan	Jun/2018-Sep/2018	43	Adults: over 18 years	60.1	Hemodialysis	Rapid Diagnostic test	Anti-HCV	Blood
377	Determination of the cut-off value of serum alanine aminotransferase in patients undergoing hemodialysis, to identify biochemical activity in patients with hepatitis C viremia.	Lopes	2006	Cross-sectional	Probabilistic	Hospital-based	Monocenter	Unclear/ Not reported	Unclear/ Not reported	Retrospectively	Brazil	Jan/2002-Oct-2002	51.2	All ages	60	Hemodialysis	Indirect ELISA	Anti-HCV	Blood
378	Prevalence of hepatitis B, hepatitis C, GB virus C/hepatitis G and TT viruses in predialysis and hemodialysis patients.	López-Alcorocho	2001	Cross-sectional	Probabilistic	Hospital-based	Monocenter	Unclear/ Not reported	Unclear/ Not reported	Prospectively	Spain	Unclear/ Not reported	65.5	Unclear/ Not reported	Unclear/ Not reported	Hemodialysis	Classical RT-PCR	Viral RNA	Blood
379	Prevalence of hepatitis C virus infection at three hemodialysis units in the western region of Puerto Rico.	López-Navedo	1999	Cross-sectional	Probabilistic	Hospital-based	Multicenter	Unclear/ Not reported	Unclear/ Not reported	Prospectively	United States of America	Dec/1997-Mar/1998	Unclear/ Not reported	Unclear/ Not reported	Unclear/ Not reported	Hemodialysis	Indirect ELISA	Anti-HCV	Blood
380	[Hepatitis C virus infection among long-term hemodialysis patients].	Lu	1997	Cross-sectional	Probabilistic	Hospital-based	Monocenter	Rural	Unclear/ Not reported	Prospectively	China	Dec/1994-Jun/1995	Unclear/ Not reported	Unclear/ Not reported	Unclear/ Not reported	Hemodialysis	Classical RT-PCR	Viral RNA	Blood
381	Hepatitis C virus infection in patients with chronic liver disease or chronic renal failure and blood donors in Thailand.	Luengrojanakul	1994	Cross-sectional	Probabilistic	Hospital-based	Monocenter	Rural	Unclear/ Not reported	Prospectively	Thailand	Unclear/ Not reported	44	Unclear/ Not reported	Unclear/ Not reported	Hemodialysis; Peritoneal dialysis	Classical RT-PCR	Viral RNA	Blood
382	Seroprevalence of human immunodeficiency virus, hepatitis B and C viruses among haemodialysis patients in two newly opened centres in Cameroon.	Luma	2017	Cross-sectional	Probabilistic	Hospital-based	Multicenter	Rural	Unclear/ Not reported	Prospectively	Cameroon	Feb/2013-Apr/2013	48	All ages	23.5	Hemodialysis	Indirect ELISA	Anti-HCV	Blood
383	Hepatitis B and C viral infections among hemodialysis patients attending 500 bedded Yangon specialty hospital, Myanmar	Lwin	2018	Cross-sectional	Non probabilistic	Hospital-based	Monocenter	Urban	Antiviral naïve	Prospectively	Myanmar	2016	48	Unclear/ Not reported	57	Hemodialysis	Immunochromatographic test	Anti-HCV	Blood
384	Prevalence of hepatitis C among haemodialysis patients in a tertiary care hospital in south India.	Madhavan	2020	Cross-sectional	Non probabilistic	Hospital-based	Multicenter	Rural	Unclear/ Not reported	Prospectively	India	Jan/2018-May/2018	58.2	Adults: over 18 years	62.5	Hemodialysis	Classical RT-PCR	Viral RNA	Blood
385	Seronegative occult hepatitis C infection among hemodialysis patients: A prevalence study.	Mahmoudvand	2021	Cross-sectional	Non probabilistic	Hospital-based	Monocenter	Urban	Antiviral naïve	Prospectively	Iran	Apr/2019-Jul/2019	57.2	Adults: over 18 years	57	Hemodialysis	Indirect ELISA	Anti-HCV	Blood
386	Prevalence and risk factors for hepatitis b and c among end-stage renal disease patients on hemodialysis in Gaborone, Botswana.	Mahupe	2021	Cross-sectional	Non probabilistic	Hospital-based	Multicenter	Urban	Unclear/ Not reported	Retrospectively	Botswana	May/2016-Jul/2016	50.5	Adults: over 18 years	Unclear/ Not reported	Hemodialysis	Microparticle enzyme immunoassay	Anti-HCV	Blood
387	Hepatitis C virus screening and clinical monitoring of biomarkers in patients undergoing hemodialysis.	Maia	2009	Cross-sectional	Non probabilistic	Hospital-based	Monocenter	Urban	Antiviral naïve	Retrospectively	Brazil	Mar/2006-Jan/2007	43	Adults: over 18 years	52.5	Hemodialysis	Indirect ELISA	Anti-HCV	Blood
388	Hepatitis C prevalence studied by polymerase chain reaction and serological methods in haemodialysis patients in Mazandaran, Iran.	Makhlough	2008	Cross-sectional	Probabilistic	Hospital-based	Multicenter	Urban	Antiviral naïve	Prospectively	Iran	Jun/2006-Aug/2006	58.8	Adults: over 18 years	60.8	Hemodialysis	Indirect ELISA	Anti-HCV	Blood
389	Antibodies to hepatitis C virus (anti-HCV): prevalence in the same geographical area in dialysis patients, staff members, and blood donors.	Malaguti	1992	Cross-sectional	Probabilistic	Hospital-based	Monocenter	Urban/Rural	Unclear/ Not reported	Prospectively	Italy	Unclear/ Not reported	Unclear/ Not reported	Adults: over 18 years	Unclear/ Not reported	Hemodialysis	Indirect ELISA	Anti-HCV	Unclear/ Not reported
390	Hepatitis B virus and hepatitis C virus co-infection in	Malhotra	2016	Cross-sectional	Probabilistic	Hospital-based	Monocenter	Urban	Unclear/ Not reported	Retrospectively	India	Jan/2013-Dec/2014	Unclear/ Not reported	Unclear/ Not reported	Unclear/ Not reported	Hemodialysis	Indirect ELISA	Anti-HCV	Unclear/ Not reported

	hemodialysis patients: A retrospective study from a tertiary care hospital of North India.																		
391	Prevalence of hepatitis B and C infection in hemodialysis patients of Rasht (Center of Guilan Province, Northern part of Iran).	Mansour-Ghanael	2009	Cross-sectional	Probabilistic	Hospital-based	Monocenter	Unclear/ Not reported	Unclear/ Not reported	Prospectively	Iran	Jun/2007-Aug/2007	42.3	Unclear/ Not reported	66	Hemodialysis	Indirect ELISA	Anti-HCV	Blood
392	HEPATITIS VIRUS INFECTION IN END-STAGE KIDNEY DISEASE PATIENTS TREATED BY HAEMODIALYSIS IN ROMANIA AFTER 5 YEARS OF ACTIVE ANTIVIRAL THERAPY, REVISITED.	Marc	2022	Cross-sectional	Non probabilistic	Hospital-based	Multicenter	Urban	Antiviral naïve	Prospectively	Romania	Unclear/ Not reported	Unclear/ Not reported	Unclear/ Not reported	Unclear/ Not reported	Hemodialysis	Unclear/ Not reported	Anti-HCV	Unclear/ Not reported
393	Antibodies against hepatitis C virus in hemodialysis patients.	Mas	1996	Cross-sectional	Non probabilistic	Hospital-based	Monocenter	Urban	Antiviral naïve	Prospectively	Turkey	Unclear/ Not reported	Unclear/ Not reported	Unclear/ Not reported	Unclear/ Not reported	Hemodialysis	Unclear/ Not reported	Anti-HCV	Unclear/ Not reported
394	Sero Conversion of Viral Hepatitis among End Stage Renal Disease Patients on Hemodialysis in Kashmir: Results of a Prospective Study.	Masoodi	2019	Cross-sectional	Non probabilistic	Hospital-based	Monocenter	Urban	Antiviral naïve	Prospectively	India	Jan/2009-Apr/2018	62.4	Adults: over 18 years	63.2	Hemodialysis	Indirect ELISA	Anti-HCV	Blood
395	Hepatitis C virus antibodies, viral RNA and genotypes in sera from patients on maintenance haemodialysis.	Masuko	1994	Cross-sectional	Non probabilistic	Hospital-based	Multicenter	Urban	Antiviral naïve	Prospectively	Japan	Unclear/ Not reported	55.5	Unclear/ Not reported	51.6	Hemodialysis	Indirect ELISA	Anti-HCV	Blood
396	Hepatitis C virus infection in renal dialysis patients in Glasgow.	McIntyre	1994	Cross-sectional	Non probabilistic	Hospital-based	Multicenter	Urban	Antiviral naïve	Prospectively	United Kingdom	1991	50.7	All ages	55.1	Hemodialysis; Peritoneal dialysis	Indirect ELISA	Anti-HCV	Blood
397	[Prevalence and associated factors to hepatitis C in hemodialysis patients in Brazil].	Medeiros	2004	Cross-sectional	Non probabilistic	Hospital-based	Multicenter	Urban	Antiviral naïve	Prospectively	Brazil	1997	Unclear/ Not reported	Unclear/ Not reported	55	Hemodialysis	Indirect ELISA	Anti-HCV	Blood
398	Different kinetics of HBV and HCV during haemodialysis and absence of seronegative viral hepatitis in patients with end-stage renal disease.	Mederacke	2011	Cross-sectional	Non probabilistic	Hospital-based	Monocenter	Unclear/ Not reported	Antiviral naïve	Prospectively	Germany	Unclear/ Not reported	Unclear/ Not reported	Unclear/ Not reported	57.0	Hemodialysis	Chemiluminescent immunoassay	Anti-HCV	Blood
399	Diagnostic utility of hepatitis C virus core antigen in hemodialysis patients.	Medhi	2008	Cohort (Baseline data)	Non probabilistic	Hospital-based	Monocenter	Unclear/ Not reported	Unclear/ Not reported	Prospectively	India	Aug/2005-Dec/2006	45.4	Adults: over 18 years	60	Hemodialysis	Classical RT-PCR	Viral RNA	Blood
400	Anti-hepatitis C virus positivity and clinical correlations in hemodialyzed patients.	Medici	1992	Cross-sectional	Non probabilistic	Hospital-based	Monocenter	Unclear/ Not reported	Unclear/ Not reported	Prospectively	Italy	Unclear/ Not reported	57.2	Unclear/ Not reported	64.2	Hemodialysis	Indirect ELISA	Anti-HCV	Blood
401	Seroconversion to hepatitis C virus in dialysis patients: a retrospective and prospective study.	Medin	1993	Cohort (Baseline data)	Non probabilistic	Hospital-based	Multicenter	Unclear/ Not reported	Unclear/ Not reported	Prospectively	Sweden	Oct/1990-Dec/1991	Unclear/ Not reported	Unclear/ Not reported	Unclear/ Not reported	Hemodialysis; Peritoneal dialysis	Indirect ELISA	Anti-HCV	Blood
402	[Hepatitis C serum prevalence in hemodialyzed patients].	Mello Lde	2007	Cross-sectional	Non probabilistic	Hospital-based	Monocenter	Unclear/ Not reported	Unclear/ Not reported	Prospectively	Brazil	Unclear/ Not reported	Unclear/ Not reported	Adults: over 18 years	Unclear/ Not reported	Hemodialysis	Indirect ELISA	Anti-HCV	Blood
403	[Risk factors for hepatitis C in hemodialysis and its impact on the waiting list for kidney transplantation].	Méndez Chacón	2005	Cross-sectional	Non probabilistic	Hospital-based	Monocenter	Unclear/ Not reported	Unclear/ Not reported	Prospectively	Peru	Dec/2000	Unclear/ Not reported	Unclear/ Not reported	Unclear/ Not reported	Hemodialysis	Unclear/ Not reported	Anti-HCV	Unclear/ Not reported
404	Prevalence of hepatitis C virus infection among hemodialysis patients at a tertiary-care hospital in Mexico City, Mexico.	Méndez-Sánchez	2004	Cross-sectional	Non probabilistic	Hospital-based	Monocenter	Unclear/ Not reported	Unclear/ Not reported	Prospectively	Mexico	Unclear/ Not reported	51	Unclear/ Not reported	53	Hemodialysis	Microparticle enzyme immunoassay	Anti-HCV	Blood
405	Prevalence and risk factors of hepatitis B and C among hemodialysis patients in Tunisia.	Mhalla	2018	Cross-sectional	Non probabilistic	Hospital-based	Monocenter	Unclear/ Not reported	Unclear/ Not reported	Prospectively	Tunisia	Jul/2013-Jun/2014	50	Adults: over 18 years	68.8	Hemodialysis	Chemiluminescent immunoassay	Anti-HCV	Blood
406	Profile of hepatitis B virus, hepatitis C virus, hepatitis d virus and human immunodeficiency virus infections in hemodialysis patients of a tertiary	Mittal	2013	Cohort (Baseline data)	Non probabilistic	Hospital-based	Monocenter	Unclear/ Not reported	Unclear/ Not reported	Prospectively	India	Unclear/ Not reported	50.0	Adults: over 18 years	66.9	Hemodialysis	Indirect ELISA	Anti-HCV	Blood

	care hospital in uttarakhand.																		
407	Hepatitis C in chronic renal failure patients.	Mitwalli	1992	Cohort (Baseline data)	Non probabilistic	Hospital-based	Monocenter	Unclear/ Not reported	Unclear/ Not reported	Prospectively	Saudi Arabia	Unclear/ Not reported	51.5	Adults: over 18 years	Unclear/ Not reported	Hemodialysis	Indirect ELISA	Anti-HCV	Blood
408	Hepatitis G virus (HGV) infection in Saudi dialysis patients and healthy controls.	Mitwalli	2000	Cross-sectional	Non probabilistic	Hospital-based	Multicenter	Unclear/ Not reported	Unclear/ Not reported	Prospectively	Saudi Arabia	Jan/1997-Dec/1997	Unclear/ Not reported	Unclear/ Not reported	53	Hemodialysis	Indirect ELISA	Anti-HCV	Blood
409	[Hepatitis C in hemodialysis patients].	Miyano	2001	Cross-sectional	Non probabilistic	Hospital-based	Monocenter	Unclear/ Not reported	Unclear/ Not reported	Prospectively	Japan	Unclear/ Not reported	Unclear/ Not reported	Unclear/ Not reported	Unclear/ Not reported	Hemodialysis	Unclear/ Not reported	Viral RNA	Unclear/ Not reported
410	[Infection of hepatitis C virus in patients with chronic renal failure undergoing hemodialysis therapy and staff members].	Miyasaka	1991	Cross-sectional	Non probabilistic	Hospital-based	Monocenter	Unclear/ Not reported	Unclear/ Not reported	Prospectively	Japan	Unclear/ Not reported	Unclear/ Not reported	Unclear/ Not reported	Unclear/ Not reported	Hemodialysis	Indirect ELISA	Anti-HCV	Blood
411	Genetic and serological evidence for multiple instances of unrecognized transmission of hepatitis C virus in hemodialysis units.	Mizuno	1998	Cohort (Baseline data)	Non probabilistic	Hospital-based	Multicenter	Unclear/ Not reported	Unclear/ Not reported	Prospectively	Japan	Unclear/ Not reported	Unclear/ Not reported	Unclear/ Not reported	Unclear/ Not reported	Hemodialysis	Classical RT-PCR	Viral RNA	Blood
412	Molecular evaluation of hepatitis G virus and hepatitis C virus in patients with chronic renal failure in Iran.	Mohsenzadeh	2012	Cross-sectional	Non probabilistic	Hospital-based	Monocenter	Urban	Antiviral naïve	Prospectively	Iran	Unclear/ Not reported	Unclear/ Not reported	Unclear/ Not reported	67.72	Hemodialysis	Indirect ELISA	Anti-HCV	Blood
413	Hepatitis C virus (HCV) Infection Rate among Seronegative Hemodialysis Patients Screened by Two Methods; HCV Core Antigen and Polymerase Chain Reaction.	Moini	2013	Cross-sectional	Non probabilistic	Hospital-based	Monocenter	Unclear/ Not reported	Unclear/ Not reported	Prospectively	Iran	Mar/2009	52.7	Unclear/ Not reported	65.2	Hemodialysis	Direct ELISA	Viral antigen	Blood
414	High prevalence of antibodies to hepatitis C virus in hemodialysis units using a second generation assay.	Mondelli	1992	Cross-sectional	Non probabilistic	Hospital-based	Multicenter	Unclear/ Not reported	Unclear/ Not reported	Prospectively	Italy	Unclear/ Not reported	Unclear/ Not reported	Unclear/ Not reported	Unclear/ Not reported	Hemodialysis	Indirect ELISA	Anti-HCV	Blood
415	Abnormal alanine aminotransferase activity reflects exposure to hepatitis C virus in haemodialysis patients.	Mondelli	1991	Cross-sectional	Non probabilistic	Hospital-based	Multicenter	Unclear/ Not reported	Unclear/ Not reported	Prospectively	Italy	1968-1990	59	All ages	54.2	Hemodialysis	Indirect ELISA	Anti-HCV	Blood
416	Hepatitis C virus infection in hemodialysis patients in Maracaibo, Venezuela.	Monsalve-Castillo	2012	Cohort (Baseline data)	Non probabilistic	Hospital-based	Monocenter	Unclear/ Not reported	Unclear/ Not reported	Prospectively	Venezuela	2007-2009	45	Adults: over 18 years	Unclear/ Not reported	Hemodialysis	Indirect ELISA	Anti-HCV	Blood
417	Epidemiology of hepatitis C virus infection in hemodialysis patients of Sicily.	Montalto	2008	Cross-sectional	Non probabilistic	Hospital-based	Multicenter	Unclear/ Not reported	Unclear/ Not reported	Prospectively	Italy	Unclear/ Not reported	65.2	Adults: over 18 years	55.2	Hemodialysis	Unclear/ Not reported	Anti-HCV	Unclear/ Not reported
418	Impact of prophylactic measures on prevalence of anti HCV and viral genotypes in a dialysis unit [13].	Morales	1997	Cross-sectional	Non probabilistic	Hospital-based	Monocenter	Urban	Antiviral naïve	Prospectively	Chile	1993–1996	Unclear/ Not reported	All ages	59.5	Hemodialysis	Indirect ELISA	Anti-HCV	Blood
419	Prospective study of hepatitis C virus infection in hemodialysis patients by monthly analysis of HCV RNA and antibodies.	Moreira	2003	Cohort (Baseline data)	Non probabilistic	Hospital-based	Multicenter	Urban	Unclear/ Not reported	Prospectively	Brazil	Mar/1997-Apr/1998	Unclear/ Not reported	Adults: over 18 years	Unclear/ Not reported	Hemodialysis	Indirect ELISA	Anti-HCV	Blood
420	Prevalence and characterization of hepatitis C virus in hemodialysis patients.	Morikawa	1999	Cohort (Baseline data)	Non probabilistic	Hospital-based	Monocenter	Rural	Unclear/ Not reported	Prospectively	Japan	Unclear/ Not reported	55	Adults: over 18 years	48.8	Hemodialysis	Indirect ELISA	Anti-HCV	Blood
421	Epidemiology of hepatitis C in a population of hemodialysis patients.	Mosconi	1992	Cross-sectional	Non probabilistic	Hospital-based	Monocenter	Urban	Unclear/ Not reported	Prospectively	Italy	Unclear/ Not reported	Unclear/ Not reported	Unclear/ Not reported	Unclear/ Not reported	Hemodialysis	Indirect ELISA	Anti-HCV	Blood
422	[The prevalence of serum anti-hepatitis C virus antibodies in hemodialyzed patients].	Motta	1991	Cross-sectional	Non probabilistic	Hospital-based	Monocenter	Urban	Unclear/ Not reported	Prospectively	Italy	Unclear/ Not reported	Unclear/ Not reported	Unclear/ Not reported	Unclear/ Not reported	Hemodialysis	Indirect ELISA	Anti-HCV	Blood
423	Epidemiology of hepatitis C virus infection in ESRD patients in Khuzestan Province, Iran.	Mousavi	2012	Cross-sectional	Non probabilistic	Hospital-based	Monocenter	Urban	Antiviral naïve	Prospectively	Iran	Dec/2010-Mar/2011	Unclear/ Not reported	Unclear/ Not reported	Unclear/ Not reported	Hemodialysis; Peritoneal dialysis	Indirect ELISA	Anti-HCV	Blood
424	Prevalence of HCV antibodies in haemodialysis	Muhammad	1997	Cross-sectional	Non probabilistic	Hospital-based	Monocenter	Unclear/ Not reported	Unclear/ Not reported	Prospectively	Pakistan	Unclear/ Not reported	Unclear/ Not reported	Unclear/ Not reported	Unclear/ Not reported	Hemodialysis	Indirect ELISA	Anti-HCV	Blood

	population of Multan - Pakistan.																		
425	Risk factors for dialysis-associated hepatitis C in Venezuela.	Muller	1992	Cross-sectional	Non probabilistic	Hospital-based	Multicenter	Urban/Rural	Unclear/ Not reported	Prospectively	Venezuela	Unclear/ Not reported	45	All ages	58.4	Hemodialysis	Indirect ELISA	Anti-HCV	Blood
426	Profile of hepatitis B and hepatitis C virus infections in dialysis and renal transplant patients 1997-2001: CMCH Vellore	Murthy	2003	Cross-sectional	Non probabilistic	Hospital-based	Monocenter	Urban	Antiviral naïve	Prospectively	India	Jun/1997-Dec/2001	33.7	Unclear/ Not reported	83	Hemodialysis	Rapid Diagnostic test	Anti-HCV	Blood
427	Incidence of antibodies to hepatitis C virus in patients undergoing chronic dialysis and CAPD.	Nakashima	1993	Cross-sectional	Non probabilistic	Hospital-based	Monocenter	Urban	Unclear/ Not reported	Prospectively	Japan	Apr/1991	53.7	Unclear/ Not reported	63.9	Hemodialysis; Peritoneal dialysis	Radioimmunoassay (RIA)	Anti-HCV	Blood
428	Hepatitis C and B virus infections in populations at low or high risk in Ho Chi Minh and Hanoi, Vietnam	Nakata	1994	Cross-sectional	Non probabilistic	Hospital-based	Multicenter	Urban	Antiviral naïve	Prospectively	Vietnam	1993	Unclear/ Not reported	Unclear/ Not reported	Unclear/ Not reported	Hemodialysis	Indirect ELISA	Anti-HCV	Blood
429	Prognosis of anti-hepatitis C virus antibody-positive patients on regular hemodialysis therapy.	Nakayama	2000	Cohort (Baseline data)	Non probabilistic	Hospital-based	Multicenter	Urban	Unclear/ Not reported	Prospectively	Japan	1993-1999	Unclear/ Not reported	All ages	12.0	Hemodialysis	Indirect ELISA	Anti-HCV	Blood
430	Low prevalence of anti-hepatitis C virus antibodies in female hemodialysis patients without blood transfusion: a multicenter analysis.	Nakayama	1996	Cohort (Baseline data)	Non probabilistic	Hospital-based	Multicenter	Urban	Unclear/ Not reported	Prospectively	Japan	Nov/1989	56.2	Unclear/ Not reported	59.8	Hemodialysis	Indirect ELISA	Anti-HCV	Blood
431	Prevalence of hepatitis b &c in patients of end stage renal disease (ESRD) on treatment of chronic/long-term hemodialysis.	Nasir	2019	Cross-sectional	Non probabilistic	Hospital-based	Monocenter	Urban	Antiviral naïve	Retrospectively	Pakistan	2014-2017	43.8	Unclear/ Not reported	60.2	Hemodialysis	Rapid Diagnostic test	Anti-HCV	Blood
432	Hepatitis C virus infection among patients on hemodialysis: a report from a single center in Iran.	Nemati	2009	Cross-sectional	Non probabilistic	Hospital-based	Monocenter	Urban	Unclear/ Not reported	Prospectively	Iran	Unclear/ Not reported	Unclear/ Not reported	Unclear/ Not reported	Unclear/ Not reported	Hemodialysis	Indirect ELISA	Anti-HCV	Blood
433	ENVIRONMENTAL TRANSMISSION OF HEPATITIS-B AND HEPATITIS-C VIRUSES WITHIN THE HEMODIALYSIS UNIT.	Neto	1995	Cross-sectional	Non probabilistic	Hospital-based	Monocenter	Urban	Unclear/ Not reported	Prospectively	Brazil	Jan/1987-Jan/1990	Unclear/ Not reported	Unclear/ Not reported	Unclear/ Not reported	Hemodialysis; Peritoneal dialysis	Indirect ELISA	Anti-HCV	Blood
434	Prevalence of hepati[1]tis C virus infection according to the year of birth: identifcation of risk groups.	Neukam	2018	Cross-sectional	Non probabilistic	Hospital-based	Monocenter	Urban	Antiviral naïve	Prospectively	Argentina	Jan/2001-Dec/2015	Unclear/ Not reported	Unclear/ Not reported	Unclear/ Not reported	Hemodialysis	Indirect ELISA	Anti-HCV	Blood
435	Outbreak of hemodialysis-associated non-A, non-B hepatitis and correlation with antibody to hepatitis C virus.	Niu	1992	Cohort (Baseline data)	Non probabilistic	Hospital-based	Monocenter	Urban	Unclear/ Not reported	Prospectively	United States of America	Jan/1987-Oct/1988	50	Unclear/ Not reported	Unclear/ Not reported	Hemodialysis	Indirect ELISA	Anti-HCV	Blood
436	Multicenter study of hepatitis C virus infection in chronic hemodialysis patients and hemodialysis center staff members.	Niu	1993	Cohort (Baseline data)	Probabilistic	Hospital-based	Multicenter	Urban	Unclear/ Not reported	Prospectively	United States of America	Apr/1990-Dec/1991	Unclear/ Not reported	Adults: over 18 years	Unclear/ Not reported	Hemodialysis	Indirect ELISA	Anti-HCV	Blood
437	Prevalence of hepatitis C virus antibody in patients on chronic hemodialysis.	Nomiyama	1998	Cross-sectional	Non probabilistic	Hospital-based	Multicenter	Unclear/ Not reported	Unclear/ Not reported	Prospectively	Japan	Unclear/ Not reported	Unclear/ Not reported	Unclear/ Not reported	Unclear/ Not reported	Hemodialysis	Indirect ELISA	Anti-HCV	Unclear/ Not reported
438	Hepatitis C virus infection in hemodialysis patients in southern Sweden: epidemiological, clinical, and diagnostic aspects.	Nordenfelt	1993	Cohort (Baseline data)	Non probabilistic	Hospital-based	Multicenter	Urban	Unclear/ Not reported	Prospectively	Sweden	Unclear/ Not reported	Unclear/ Not reported	Unclear/ Not reported	Unclear/ Not reported	Hemodialysis	Indirect ELISA	Anti-HCV	Blood
439	Seroprevalence of hepatitis C in patients with type 2 diabetes mellitus and non-diabetic on haemodialysis.	ocak	2006	Cross-sectional	Non probabilistic	Hospital-based	Multicenter	Urban	Antiviral naïve	Prospectively	Turkey	Unclear/ Not reported	52.1	All ages	68.1	Hemodialysis	Chemiluminescent immunoassay	Anti-HCV	Blood
440	Hepatitis virus infection (HBV and HCV) in eleven Japanese hemodialysis units.	Oguchi	1992	Cross-sectional	Non probabilistic	Hospital-based	Monocenter	Urban	Unclear/ Not reported	Prospectively	Japan	Unclear/ Not reported	Unclear/ Not reported	Unclear/ Not reported	Unclear/ Not reported	Hemodialysis	Indirect ELISA	Anti-HCV	Blood
441	Prevalence of anti-HCV in patients on long-term hemodialysis.	Oguchi	1990	Cross-sectional	Non probabilistic	Hospital-based	Multicenter	Unclear/ Not reported	Unclear/ Not reported	Prospectively	Japan	Unclear/ Not reported	Unclear/ Not reported	Unclear/ Not reported	Unclear/ Not reported	Hemodialysis	Indirect ELISA	Anti-HCV	Blood

442	Standardized prevalence ratios for chronic hepatitis C virus infection among adult Japanese hemodialysis patients.	Ohsawa	2010	Cohort (Baseline data)	Non probabilistic	Hospital-based	Multicenter	Urban	Unclear/ Not reported	Prospectively	Japan	Jun/2003-Mar/2004	Unclear/ Not reported	Adults: over 18 years	64.2	Hemodialysis	Chemiluminescent immunoassay	Anti-HCV	Blood
443	Seropositivity for anti-HCV core antigen is independently associated with increased all[1]cause, cardiovascular, and liver disease-related mortality in haemodialysis patients.	Ohsawa	2010	Cohort (Baseline data)	Non probabilistic	Hospital-based	Monocenter	Unclear/ Not reported	Unclear/ Not reported	Prospectively	Japan	Apr/2003-Mar/2008	Unclear/ Not reported	Adults: over 18 years	64.1	Hemodialysis	Enzyme immunoassay (EIA)	Viral antigen	Blood
444	Sero-prevalence of Hepatitis B and C infection among patients with end stage renal disease at haemodialysis initiation. African Journal of Medical and Health Sciences.	Okoye	2020	Cross-sectional	Non probabilistic	Hospital-based	Monocenter	Urban	Antiviral naïve	Prospectively	Nigeria	Jan/2012-Jan/2016	Unclear/ Not reported	Unclear/ Not reported	Unclear/ Not reported	Hemodialysis	Rapid Diagnostic test	Anti-HCV	Unclear/ Not reported
445	Epidemiological Survey of Patients With Hemodialysis Complicated by Hepatitis C in Japan.	Okubo	2019	Cross-sectional	Non probabilistic	Hospital-based	Multicenter	Urban	Antiviral naïve	Prospectively	Jordan	Jun/2016-Sep/2017	Unclear/ Not reported	Unclear/ Not reported	Unclear/ Not reported	Hemodialysis	Unclear/ Not reported	Anti-HCV	Unclear/ Not reported
446	Markers of hepatitis C infection among hemodialysis patients with acute and chronic infection: implications for infection control strategies in hemodialysis units.	Oliva	1995	Cohort (Baseline data)	Non probabilistic	Hospital-based	Multicenter	Unclear/ Not reported	Unclear/ Not reported	Prospectively	Spain	Unclear/ Not reported	Unclear/ Not reported	Unclear/ Not reported	Unclear/ Not reported	Hemodialysis	Classical RT-PCR	Viral RNA	Blood
447	Late seroconversion of C virus markers in hemodialysis patients.	Oliva	1993	Cohort (Baseline data)	Non probabilistic	Hospital-based	Multicenter	Unclear/ Not reported	Unclear/ Not reported	Prospectively	Spain	1988-1990	Unclear/ Not reported	Unclear/ Not reported	Unclear/ Not reported	Hemodialysis	Unclear/ Not reported	Anti-HCV	Unclear/ Not reported
448	Seroprevalence of hepatitis C virus infection and evaluation of serum aminotransferase levels among haemodialysis patients in Izmir, Turkey.	Olut	2005	Cohort (Baseline data)	Non probabilistic	Hospital-based	Multicenter	Urban	Unclear/ Not reported	Prospectively	Turkey	Jan/2004-Jun/2004	51.3	Unclear/ Not reported	54.9	Hemodialysis	Indirect ELISA	Anti-HCV	Blood
449	Liver enzymes and protein electrophoretic patterns in hemodialysis patients with antibodies against the hepatitis C virus.	Omar	1995	Cohort (Baseline data)	Non probabilistic	Hospital-based	Monocenter	Urban	Unclear/ Not reported	Prospectively	Saudi Arabia	Unclear/ Not reported	48	Unclear/ Not reported	58.4	Hemodialysis	Indirect ELISA	Anti-HCV	Blood
450	A prospective study of the prevalence of hepatitis B and C virus co-infection among patients with chronic renal disease under hemodialysis.	Ono-nita	2004	Cross-sectional	Non probabilistic	Hospital-based	Multicenter	Urban	Antiviral naïve	Prospectively	Brazil	1996-1998	Unclear/ Not reported	Unclear/ Not reported	Unclear/ Not reported	Hemodialysis	Unclear/ Not reported	Anti-HCV	Unclear/ Not reported
451	Seroprevalence of hepatitis B and C in maintenance dialysis in a public hospital in a developing country.	Otedo	2003	Cross-sectional	Non probabilistic	Hospital-based	Monocenter	Urban	Unclear/ Not reported	Prospectively	Kenya	Mar/1998-Nov/1998	43.3	Unclear/ Not reported	69.5	Hemodialysis; Peritoneal dialysis	Microparticle enzyme immunoassay	Anti-HCV	Blood
452	Prevalence of antibodies to hepatitis C virus among hemodialysis patients in Damascus, Syria.	Othman	2001	Cross-sectional	Non probabilistic	Hospital-based	Multicenter	Urban	Unclear/ Not reported	Prospectively	Syria	Jan/1996-Mar/1996	38.9	All ages	57.6	Hemodialysis	Indirect ELISA	Anti-HCV	Blood
453	Revelance of hepatitis C virus RNA detection, quantitation and genotypes in a hemodialysis unit.	Ouzan	1997	Cross-sectional	Non probabilistic	Hospital-based	Monocenter	Urban	Antiviral naïve	Prospectively	French Guiana	Unclear/ Not reported	Unclear/ Not reported	Unclear/ Not reported	Unclear/ Not reported	Hemodialysis	Indirect ELISA	Anti-HCV	Blood
454	[Anti HCV in hemodialyzed patients: reduction of the prevalence and association with epidemiological variables].	Padrone	1999	Cross-sectional	Non probabilistic	Hospital-based	Multicenter	Urban	Unclear/ Not reported	Prospectively	Argentina	Unclear/ Not reported	Unclear/ Not reported	Unclear/ Not reported	Unclear/ Not reported	Hemodialysis	Unclear/ Not reported	Anti-HCV	Unclear/ Not reported
455	Prevalence of HCV, HBV and HIV infections in patients and staff of haemodialysis unit.	Palanisamy	2012	Cross-sectional	Non probabilistic	Hospital-based	Multicenter	Unclear/ Not reported	Unclear/ Not reported	Prospectively	India	Unclear/ Not reported	Unclear/ Not reported	Unclear/ Not reported	Unclear/ Not reported	Hemodialysis	Rapid Diagnostic test	Anti-HCV	Blood
456	Risk factors and clinical expression of HCV infection in	Pauri	1992	Cross-sectional	Non probabilistic	Hospital-based	Monocenter	Unclear/ Not reported	Unclear/ Not reported	Prospectively	Italy	1990-1991	50	Unclear/ Not reported	Unclear/ Not reported	Hemodialysis	Indirect ELISA	Anti-HCV	Blood

	hemodialysis patients.																		
457	Hepatitis C virus seropositivity and TNF superfamily receptors: sCD40, sFas--the new putative determinants of endothelial dysfunction in haemodialysis patients.	Pawlak	2010	Cross-sectional	Non probabilistic	Hospital-based	Monocenter	Unclear/ Not reported	Unclear/ Not reported	Prospectively	Poland	Unclear/ Not reported	Unclear/ Not reported	Adults: over 18 years	62.3	Hemodialysis	Microparticle enzyme immunoassay	Anti-HCV	Blood
458	The prevalence of anti-HCV positivity in patients undergoing haemodialysis or with malignant disease.	Paydas	1994	Cross-sectional	Non probabilistic	Hospital-based	Monocenter	Urban	Antiviral naïve	Prospectively	Turkey	Unclear/ Not reported	Unclear/ Not reported	Unclear/ Not reported	Unclear/ Not reported	Hemodialysis	Indirect ELISA	Anti-HCV	Blood
459	[Hepatitis C virus antibodies in persons on dialysis].	Pazdiora	1993	Cross-sectional	Non probabilistic	Hospital-based	Monocenter	Urban	Antiviral naïve	Prospectively	Germany	Unclear/ Not reported	Unclear/ Not reported	Unclear/ Not reported	Unclear/ Not reported	Hemodialysis	Indirect ELISA	Anti-HCV	Blood
460	[Viral hepatitis C--a problem in the treatment of children with renal insufficiency on hemodialysis].	Peco-Antić	1993	Cross-sectional	Non probabilistic	Hospital-based	Monocenter	Urban	Antiviral naïve	Prospectively	Serbia	Nov/1990-Sep/1992	Unclear/ Not reported	Unclear/ Not reported	58.3	Hemodialysis	Indirect ELISA	Anti-HCV	Blood
461	[Epidemiologic study of infection by hepatitis C virus in a hemodialysis unit].	Pena	2000	Cross-sectional	Non probabilistic	Hospital-based	Monocenter	Urban	Antiviral naïve	Prospectively	Spain	Unclear/ Not reported	Unclear/ Not reported	Unclear/ Not reported	Unclear/ Not reported	Hemodialysis	Indirect ELISA	Anti-HCV	Blood
462	Influence of hepatitis C virus infection on soluble cellular adhesion molecules in hemodialysis patients.	Peng	2005	Cross-sectional	Non probabilistic	Hospital-based	Monocenter	Urban	Antiviral naïve	Prospectively	China	Unclear/ Not reported	60	Unclear/ Not reported	35.6	Hemodialysis	Indirect ELISA	Anti-HCV	Blood
463	Seroprevalence of hepatitis B, hepatitis C and HIV infection among patients undergoing haemodialysis in Buenos Aires, Argentina	Pereson	2021	Cross-sectional	Non probabilistic	Hospital-based	Monocenter	Unclear/ Not reported	Unclear/ Not reported	Retrospectively	Argentina	Jan/2001-Dec/2018	59	Unclear/ Not reported	54.8	Hemodialysis	Enzyme immunoassay (EIA)	Anti-HCV	Blood
464	Prevalence of antihepatitis C antibodies in patients treated with continuous ambulatory peritoneal dialysis and hemodialysis.	Pérez-Fontán	1991	Cross-sectional	Non probabilistic	Hospital-based	Monocenter	Urban	Antiviral naïve	Prospectively	Spain	Mar/1990	51	Unclear/ Not reported	61	Hemodialysis; Peritoneal dialysis	Indirect ELISA	Anti-HCV	Blood
465	Prevalence of infected patients and understaffing have a role in hepatitis C virus transmission in dialysis.	Petrosillo	2001	Cross-sectional	Non probabilistic	Hospital-based	Multicenter	Urban	Antiviral naïve	Prospectively	Italy	Jan/1997-Jan/1998	69	Unclear/ Not reported	55	Hemodialysis	Indirect ELISA	Anti-HCV	Blood
466	Prevalence of human immunodeficiency virus, hepatitis B virus and hepatitis C virus among dialysis patients. The Italian Multicentric Study on Nosocomial and Occupational Risk of Blood-Borne Infections in Dialysis.	Petrosillo	1993	Cross-sectional	Non probabilistic	Hospital-based	Multicenter	Urban	Antiviral naïve	Prospectively	Italy	1991	58	Adults: over 18 years	56.8	Hemodialysis	Indirect ELISA	Anti-HCV	Blood
467	The risks of occupational exposure and infection by human immunodeficiency virus, hepatitis B virus, and hepatitis C virus in the dialysis setting. Italian Multicenter Study on Nosocomial and Occupational Risk of Infections in Dialysis.	Petrosillo	1995	Cross-sectional	Non probabilistic	Hospital-based	Multicenter	Urban	Antiviral naïve	Prospectively	Italy	Feb/1993-Jan/1994	Unclear/ Not reported	Adults: over 18 years	Unclear/ Not reported	Hemodialysis	Indirect ELISA	Anti-HCV	Blood
468	Anti-hepatitis C virus antibodies and hepatitis C virus viraemia in haemodialysis patients.	Picciotto	1993	Cross-sectional	Non probabilistic	Hospital-based	Monocenter	Urban	Antiviral naïve	Prospectively	Italy	Unclear/ Not reported	57.7	Adults: over 18 years	56.1	Hemodialysis	Immunoblot Assay	Anti-HCV	Blood
469	[Hepatitis C virus infection in patients on chronic hemodialysis].	Pljesa	1996	Cross-sectional	Non probabilistic	Hospital-based	Monocenter	Urban	Antiviral naïve	Prospectively	Serbia	Unclear/ Not reported	52.6	Unclear/ Not reported	50.9	Hemodialysis	Indirect ELISA	Anti-HCV	Blood
470	Hepatitis C virus-related acute and chronic hepatitis in hemodialysis patients.	Pluvio	1992	Cross-sectional	Non probabilistic	Hospital-based	Monocenter	Urban	Antiviral naïve	Prospectively	Italy	Unclear/ Not reported	Unclear/ Not reported	Unclear/ Not reported	44.7	Hemodialysis	Indirect ELISA	Anti-HCV	Blood
471	Serprevalence of hepatitis-C virus in blood donors and high risk individuals.	Poddar	2012	Case control	Non probabilistic	Hospital-based	Monocenter	Unclear/ Not reported	Unclear/ Not reported	Prospectively	India	Mar/2005-Jun/2006	Unclear/ Not reported	Unclear/ Not reported	Unclear/ Not reported	Hemodialysis	Indirect ELISA	Anti-HCV	Blood
472	Prevalence of hepatitis B & C viruses among	Prakash	2014	Cross-sectional	Non probabilistic	Hospital-based	Multicenter	Unclear/ Not reported	Unclear/ Not reported	Prospectively	India	Unclear/ Not reported	45.1	All ages	62.9	Hemodialysis	Indirect ELISA	Anti-HCV	Blood

	patients on hemodialysis in Lucknow, Uttar Pradesh.																		
473	Comparison of third generation ELISA and conventional nested RT-PCR for detection of HCV among hemodialysis patients.	Prakash	2014	Cross-sectional	Non probabilistic	Hospital-based	Monocenter	Unclear/ Not reported	Unclear/ Not reported	Prospectively	India	Unclear/ Not reported	45.7	All ages	64.5	Hemodialysis	Indirect ELISA	Anti-HCV	Blood
474	Hepatitis E virus (HEV) infection in haemodialysis patients.	Psichogiou	1996	Cross-sectional	Non probabilistic	Hospital-based	Multicenter	Urban	Unclear/ Not reported	Prospectively	Greece	Unclear/ Not reported	57.3	All ages	55.9	Hemodialysis	Indirect ELISA	Anti-HCV	Blood
475	High incidence of hepatitis C virus infection in hemodialysis patients in units with high prevalence.	Pujol	1996	Cross-sectional	Non probabilistic	Hospital-based	Multicenter	Urban	Unclear/ Not reported	Retrospectively	Venezuela	1994	Unclear/ Not reported	Adults: over 18 years	56.8	Hemodialysis	Indirect ELISA	Anti-HCV	Blood
476	Prevalence of hepatitides in our hemodialyzed population.	Raccosta	1992	Cross-sectional	Non probabilistic	Hospital-based	Multicenter	Unclear/ Not reported	Unclear/ Not reported	Retrospectively	Italy	Unclear/ Not reported	61.6	Adults: over 18 years	56.1	Hemodialysis	Indirect ELISA	Anti-HCV	Blood
477	Seroprevalence and risk factors for Hepatitis C virus among maintenance hemodialysis patients at a Tertiary Care Hospital in Puducherry, India.	Ragunathan	2022	Cross-sectional	Non probabilistic	Hospital-based	Monocenter	Urban	Antiviral naïve	Retrospectively	India	Jan/2016–Dec/2019	50.45	Unclear/ Not reported	81	Hemodialysis	Real-time RT-PCR	Viral RNA	Blood
478	Prevalence of Hepatitis B and Hepatitis C in Patients undergoing hemodialysis at a teaching hospital in Uttarakhand.	Raina	2022	Cross-sectional	Non probabilistic	Hospital-based	Multicenter	Urban	Antiviral naïve	Prospectively	India	2019	Unclear/ Not reported	Unclear/ Not reported	Unclear/ Not reported	Hemodialysis	Chemiluminescent immunoassay	Anti-HCV	Blood
479	Anti-HCV seropositivity among haemodialysis patients of Iranian origin.	Rais-Jalali	1999	Cross-sectional	Non probabilistic	Hospital-based	Multicenter	Unclear/ Not reported	Unclear/ Not reported	Prospectively	Iran	Unclear/ Not reported	Unclear/ Not reported	Adults: over 18 years	60.9	Hemodialysis	Indirect ELISA	Anti-HCV	Blood
480	Serological pattern of anti-HBc alone infers occult hepatitis B virus infection in high-risk individuals in Iran.	Ramezani	2013	Cross-sectional	Non probabilistic	Hospital-based	Monocenter	Unclear/ Not reported	Unclear/ Not reported	Prospectively	Iran	Jan/2008-Dec/2008	55	Unclear/ Not reported	60.2	Hemodialysis	Indirect ELISA	Anti-HCV	Blood
481	Improved detection of hepatitis C virus infection by transcription-mediated amplification technology in dialysis population.	Rao	2010	Cross-sectional	Non probabilistic	Hospital-based	Monocenter	Unclear/ Not reported	Antiviral naïve	Prospectively	United States of America	Unclear/ Not reported	54.8	Adults: over 18 years	64.3	Hemodialysis	Indirect ELISA	Anti-HCV	Blood
482	Hepatitis C in several risk groups of Kosovo.	Rashiti-Bytyci	2022	Cross-sectional	Non probabilistic	Hospital-based	Multicenter	Unclear/ Not reported	Unclear/ Not reported	Prospectively	Kosovo	2019	Unclear/ Not reported	Unclear/ Not reported	Unclear/ Not reported	Hemodialysis	Chemiluminescent immunoassay	Anti-HCV	Blood
483	Utility of HCV core antigen ELISA in the screening for hepatitis C virus infection in patients on hemodialysis.	Reddy	2006	Cross-sectional	Non probabilistic	Hospital-based	Monocenter	Unclear/ Not reported	Unclear/ Not reported	Prospectively	India	May/2003-Oct/2004	Unclear/ Not reported	Unclear/ Not reported	Unclear/ Not reported	Hemodialysis	Real-time RT-PCR	Viral RNA	Blood
484	Prevalence of HBV and HCV dual infection in patients on haemodialysis.	Reddy	2005	Cross-sectional	Non probabilistic	Hospital-based	Monocenter	Unclear/ Not reported	Unclear/ Not reported	Prospectively	India	May/2003-Apr/2004	Unclear/ Not reported	Unclear/ Not reported	Unclear/ Not reported	Hemodialysis	Indirect ELISA	Anti-HCV	Blood
485	Hepatitis C infection and the risk of bacteremia in hemodialysis patients with tunneled vascular access catheters.	Reddy	2009	Cross-sectional	Non probabilistic	Hospital-based	Monocenter	Unclear/ Not reported	Unclear/ Not reported	Prospectively	United States of America	Apr/2006	Unclear/ Not reported	Unclear/ Not reported	Unclear/ Not reported	Hemodialysis	Rapid Diagnostic test	Anti-HCV	Blood
486	Cross-Sectional Study to Determine the Prevalence of Hepatitis B and C Virus Infection in High Risk Groups in the Northeast Region of Brazil.	Ribeiro	2017	Cross-sectional	Non probabilistic	Hospital-based	Multicenter	Unclear/ Not reported	Unclear/ Not reported	Prospectively	Brazil	Jun/2014-Mar/2015	Unclear/ Not reported	Adults: over 18 years	68.5	Hemodialysis	Indirect ELISA	Anti-HCV	Blood
487	HCV-RNA qualitative assay based on transcription mediated amplification improves the detection of hepatitis C virus infection in patients on hemodialysis: results from five hemodialysis units in central Greece.	Rigopoulou	2005	Cross-sectional	Non probabilistic	Hospital-based	Multicenter	Unclear/ Not reported	Antiviral naïve	Prospectively	Greece	Unclear/ Not reported	60.5	Unclear/ Not reported	66.3	Hemodialysis	Transcription-Mediated Amplification (TMA)	Viral RNA	Blood
488	Hepatitis B and C virus infection among hemodialysis patients in Yogyakarta, Indonesia:	Rinonce	2013	Cross-sectional	Probabilistic	Hospital-based	Monocenter	Unclear/ Not reported	Unclear/ Not reported	Prospectively	Indonesia	Jan/2010-Feb/2010	48	Unclear/ Not reported	58	Hemodialysis	Particle Agglutination	Anti-HCV	Blood

	Prevalence and molecular evidence for nosocomial transmission.																		
489	Detection of antibodies to hepatitis C virus in dialysis patients.	Rivanera	1993	Cross-sectional	Non probabilistic	Hospital-based	Monocenter	Unclear/ Not reported	Unclear/ Not reported	Prospectively	Italy	Jan/1990-Sep/1991	Unclear/ Not reported	Adults: over 18 years	61.9	Hemodialysis	Indirect ELISA	Anti-HCV	Blood
490	Prevalence of TT virus infection in Italian-dialysis patients.	Rivanera	2009	Cross-sectional	Non probabilistic	Hospital-based	Monocenter	Unclear/ Not reported	Unclear/ Not reported	Prospectively	Italy	Unclear/ Not reported	Unclear/ Not reported	Unclear/ Not reported	71.7	Hemodialysis	Classical RT-PCR	Viral RNA	Blood
491	Prevalence of hepatitis C virus infection and genotypes in patient with chronic kidney disease undergoing hemodialysis.	Rodrigues de Freitas	2013	Cross-sectional	Non probabilistic	Hospital-based	Multicenter	Unclear/ Not reported	Unclear/ Not reported	Prospectively	Brazil	Jan/2011-Dec/2011	49	Adults: over 18 years	66	Hemodialysis	Indirect ELISA	Anti-HCV	Blood
492	Hepatitis C virus infection in haemodialysis patients.	Roger	1991	Cross-sectional	Non probabilistic	Hospital-based	Monocenter	Unclear/ Not reported	Unclear/ Not reported	Prospectively	Australia	Unclear/ Not reported	50	Unclear/ Not reported	60	Hemodialysis	Indirect ELISA	Anti-HCV	Blood
493	Health related quality of life in Iranian hemodialysis patients with viral hepatitis: changing epidemiology.	Rostami	2013	Cross-sectional	Non probabilistic	Hospital-based	Multicenter	Unclear/ Not reported	Unclear/ Not reported	Prospectively	Iran	Oct/2010-Aug/2011	55	Adults: over 18 years	57	Hemodialysis	Indirect ELISA	Anti-HCV	Blood
494	Hemodialysis as predisposing factor for viral hepatitis B and C.	Roşu	2015	Cross-sectional	Non probabilistic	Hospital-based	Multicenter	Urban	Antiviral naïve	Prospectively	Spain	Unclear/ Not reported	Unclear/ Not reported	Adults: over 18 years	Unclear/ Not reported	Hemodialysis	Unclear/ Not reported	Anti-HCV	Unclear/ Not reported
495	Prevalence and genotyping pattern of hepatitis C virus among patients on maintenance hemodialysis at five centers in Pune, India.	Roy	2019	Cross-sectional	Non probabilistic	Hospital-based	Multicenter	Unclear/ Not reported	Unclear/ Not reported	Prospectively	India	Feb/2014-Jan/2015	47.3	Unclear/ Not reported	82.8	Hemodialysis	Enzyme immunoassay (EIA)	Anti-HCV	Blood
496	Hepatitis C virus infection in hemodialyzed patients detected by first and second generation assays.	Ruffatti	1992	Cross-sectional	Non probabilistic	Hospital-based	Monocenter	Unclear/ Not reported	Antiviral naïve	Prospectively	Italy	1987-1990	58	Adults: over 18 years	60	Hemodialysis	Indirect ELISA	Anti-HCV	Blood
497	Serum alanine aminotransferase in hepatitis c screening of patients on hemodialysis.	Saab	2001	Cross-sectional	Non probabilistic	Hospital-based	Multicenter	Unclear/ Not reported	Antiviral naïve	Prospectively	United States of America	Jan/1998-Dec/1998	Unclear/ Not reported	Unclear/ Not reported	Unclear/ Not reported	Hemodialysis	Indirect ELISA	Anti-HCV	Blood
498	Effect of HCV infection on hematocrit and hemoglobin level in Egyptian hemodialysis patients.	Sabry	2009	Cross-sectional	Non probabilistic	Hospital-based	Monocenter	Unclear/ Not reported	Antiviral naïve	Retrospectively	Egypt	Unclear/ Not reported	40.4	Unclear/ Not reported	68.69	Hemodialysis	Indirect ELISA	Anti-HCV	Blood
499	Hepatitis C virus infection in hemodialysis patients in jordan.	Said	1995	Cross-sectional	Non probabilistic	Hospital-based	Monocenter	Urban	Antiviral naïve	Prospectively	Jordan	1994	Unclear/ Not reported	All ages	Unclear/ Not reported	Hemodialysis	Indirect ELISA	Anti-HCV	Blood
500	Prevalence of hepatitis C virus infection among long-term hemodialysis patients: detection of hepatitis C virus RNA in plasma.	Sakamoto	1993	Cross-sectional	Non probabilistic	Hospital-based	Monocenter	Urban	Antiviral naïve	Prospectively	Japan	Unclear/ Not reported	53	Adults: over 18 years	59.8	Hemodialysis	Indirect ELISA	Anti-HCV	Blood
501	Prevalence of hepatitis C virus infection among haemodialysis patients in West Java, Indonesia.	Saketi	2003	Cross-sectional	Non probabilistic	Hospital-based	Multicenter	Urban	Antiviral naïve	Prospectively	Indonesia	1999	49	Adults: over 18 years	60.8	Hemodialysis	Indirect ELISA	Anti-HCV	Blood
502	Hepatitis C virus infection in French hemodialysis units: a multicenter study.	Salama	2000	Cross-sectional	Non probabilistic	Hospital-based	Multicenter	Urban	Antiviral naïve	Prospectively	France	Unclear/ Not reported	65	Adults: over 18 years	59.9	Hemodialysis	Indirect ELISA	Anti-HCV	Blood
503	Hepatitis G virus exposure in dialysis patients and blood donors in Isfahan-Iran.	Salehi	2014	Cross-sectional	Probabilistic	Hospital-based	Multicenter	Urban	Antiviral naïve	Prospectively	Iran	2008	49	Unclear/ Not reported	47.5	Hemodialysis	Indirect ELISA	Anti-HCV	Blood
504	Prevalence of antibodies to hepatitis C virus in HBsAg negative hemodialysis patients.	Salunkhe	1992	Cross-sectional	Non probabilistic	Hospital-based	Monocenter	Urban	Antiviral naïve	Prospectively	India	Unclear/ Not reported	Unclear/ Not reported	Unclear/ Not reported	Unclear/ Not reported	Hemodialysis	Unclear/ Not reported	Anti-HCV	Unclear/ Not reported
505	Prevalence of hepatitis C virus antibody and related risk factors among hemodialysis patients in Markazi province (2004).	Samimi rad	2006	Cross-sectional	Non probabilistic	Hospital-based	Monocenter	Unclear/ Not reported	Unclear/ Not reported	Prospectively	Iran	Unclear/ Not reported	Unclear/ Not reported	Unclear/ Not reported	Unclear/ Not reported	Hemodialysis	Enzyme immunoassay (EIA)	Anti-HCV	Blood
506	Hepatitis C virus infection among multi-transfused patients and personnel in haemodialysis units	Samimi Rad	2012	Cross-sectional	Non probabilistic	Hospital-based	Multicenter	Unclear/ Not reported	Unclear/ Not reported	Prospectively	Iran	Apr/2006-Jun/2006	57.0	Unclear/ Not reported	65.6	Hemodialysis	Indirect ELISA	Anti-HCV	Blood

	in central Islamic Republic of Iran.																		
507	Hepatitis C virus infection and HCV genotypes of hemodialysis patients.	Samimi Rad	2008	Cross-sectional	Non probabilistic	Hospital-based	Multicenter	Unclear/ Not reported	Unclear/ Not reported	Prospectively	Iran	Mar/2005-Jul/2005	53.7	All ages	49.5	Hemodialysis	Indirect ELISA	Anti-HCV	Blood
508	High prevalence of a rare hepatitis C virus in patients treated in the same hemodialysis unit: evidence for nosocomial transmission of HCV.	Sampietro	1995	Cross-sectional	Non probabilistic	Hospital-based	Multicenter	Urban	Antiviral naïve	Prospectively	Italy	1993	55	Adults: over 18 years	56.2	Hemodialysis	Classical RT-PCR	Viral RNA	Blood
509	Hepatitis C virus antibodies in patients on hemodialysis.	San Miguel	1992	Cross-sectional	Non probabilistic	Hospital-based	Multicenter	Urban	Antiviral naïve	Prospectively	Spain	Unclear/ Not reported	44	Unclear/ Not reported	31.5	Hemodialysis	Indirect ELISA	Anti-HCV	Blood
510	Hepatitis C prevalence and risk factors in the northern Alberta dialysis population.	Sandhu	1999	Cross-sectional	Non probabilistic	Hospital-based	Multicenter	Urban	Antiviral naïve	Prospectively	Canada	1997	57.4	Unclear/ Not reported	60.7	Hemodialysis	Indirect ELISA	Anti-HCV	Blood
511	Hepatitis status and mortality in hemodialysis population.	Santoro	2009	Cross-sectional	Non probabilistic	Hospital-based	Multicenter	Urban	Antiviral naïve	Prospectively	Italy	Jan/1999-Dec/2000	71.1	Unclear/ Not reported	58.8	Hemodialysis	Indirect ELISA	Anti-HCV	Blood
512	Infection by the hepatitis C virus in chronic renal failure patients undergoing hemodialysis in Mato Grosso state, central Brazil: a cohort study.	Santos	2007	Cross-sectional	Non probabilistic	Hospital-based	Multicenter	Urban	Antiviral naïve	Prospectively	Brazil	Jan/2002-May/2005	50	Unclear/ Not reported	61	Hemodialysis	Microparticle enzyme immunoassay	Anti-HCV	Blood
513	Prevalence of hepatitis B and hepatitis C in haemodialysis patients.	Santos	1998	Cross-sectional	Non probabilistic	Hospital-based	Multicenter	Urban	Antiviral naïve	Retrospectively	Philippines	Aug/1994-Feb/1995	44.9	Unclear/ Not reported	48.8	Hemodialysis	Indirect ELISA	Anti-HCV	Blood
514	Hepatitis C virus and hepatitis B virus infection in patients and staff of haemodialysis unit - A report from Chennai.	Saraswathy	2013	Cross-sectional	Non probabilistic	Hospital-based	Monocenter	Unclear/ Not reported	Antiviral naïve	Prospectively	India	2011	Unclear/ Not reported	Unclear/ Not reported	Unclear/ Not reported	Hemodialysis	Rapid Diagnostic test	Anti-HCV	Blood
515	Hepatitis C virus antibodies in dialysis patients in Tunisia: a single center study.	Sassi	2000	Cross-sectional	Non probabilistic	Hospital-based	Monocenter	Urban	Antiviral naïve	Prospectively	Tunisia	Unclear/ Not reported	48	All ages	60.3	Hemodialysis	Indirect ELISA	Anti-HCV	Blood
516	Decreased prevalence and incidence of HCV markers in haemodialysis units: a multicentric French survey.	Sauné	2011	Cross-sectional	Non probabilistic	Hospital-based	Multicenter	Urban	Antiviral naïve	Prospectively	France	Unclear/ Not reported	72	Adults: over 18 years	61	Hemodialysis	Indirect ELISA	Anti-HCV	Blood
517	A large nosocomial outbreak of hepatitis C virus infections at a hemodialysis center.	Savey	2005	Cross-sectional	Non probabilistic	Hospital-based	Multicenter	Urban	Antiviral naïve	Prospectively	France	2001	70	Unclear/ Not reported	68.2	Hemodialysis	Classical RT-PCR	Viral RNA	Blood
518	The impact of nurse understaffing on the transmission of hepatitis C virus in a hospital-based hemodialysis unit.	Saxena	2004	Cross-sectional	Non probabilistic	Hospital-based	Multicenter	Urban	Antiviral naïve	Prospectively	Saudi Arabia	Aug/1995-Aug/2000	47.0	All ages	54	Hemodialysis	Indirect ELISA	Anti-HCV	Blood
519	Prevalence of hepatitis C antibodies among hemodialysis patients in Al-hasa region of saudi arabia.	Saxena	2001	Cross-sectional	Non probabilistic	Hospital-based	Monocenter	Urban	Antiviral naïve	Prospectively	Saudi Arabia	Unclear/ Not reported	47.5	All ages	48.1	Hemodialysis	Indirect ELISA	Anti-HCV	Blood
520	The role the type of vascular access plays in the transmission of hepatitis C virus in a high prevalence hemodialysis unit.	Saxena	2002	Cross-sectional	Non probabilistic	Hospital-based	Monocenter	Urban	Antiviral naïve	Retrospectively	Saudi Arabia	Nov/1995-Nov/2000	47.0	All ages	54.0	Hemodialysis	Indirect ELISA	Anti-HCV	Blood
521	Impact of dedicated space, dialysis equipment, and nursing staff on the transmission of hepatitis C virus in a hemodialysis unit of the middle east.	Saxena	2003	Cross-sectional	Non probabilistic	Hospital-based	Monocenter	Urban	Antiviral naïve	Retrospectively	Saudi Arabia	Dec/1995-Dec/2000	47.5	All ages	48.2	Hemodialysis	Indirect ELISA	Anti-HCV	Blood
522	HCV infection in haemodialysis and CAPD patients.	Sayiner	1999	Cross-sectional	Non probabilistic	Hospital-based	Monocenter	Urban	Antiviral naïve	Prospectively	Turkey	Unclear/ Not reported	Unclear/ Not reported	Unclear/ Not reported	Unclear/ Not reported	Hemodialysis; Peritoneal dialysis	Indirect ELISA	Anti-HCV	Blood
523	Hepatitis B and C virus infection in the hemodialysis population from three romanian regions.	Schiller	2015	Cross-sectional	Non probabilistic	Hospital-based	Multicenter	Urban	Antiviral naïve	Prospectively	Romania	2010	56	Unclear/ Not reported	55.3	Hemodialysis	Chemiluminescent immunoassay	Anti-HCV	Blood
524	Hepatitis C virus antibodies in haemodialysis patients.	Schlipköter	1990	Cross-sectional	Non probabilistic	Hospital-based	Multicenter	Urban	Antiviral naïve	Prospectively	Germany	Unclear/ Not reported	Unclear/ Not reported	Unclear/ Not reported	Unclear/ Not reported	Hemodialysis	Indirect ELISA	Anti-HCV	Blood
525	Prevalence of hepatitis C virus infections in dialysis	Schlipköter	1992	Cross-sectional	Non probabilistic	Hospital-based	Multicenter	Urban	Antiviral naïve	Prospectively	Germany	Unclear/ Not reported	60	All ages	57.4	Hemodialysis	Indirect ELISA	Anti-HCV	Blood

	patients and their contacts using a second generation enzymed-linked immunosorbent assay.																		
526	Hepatitis C virus infections in dialysis centers in The Netherlands: a national survey by serological and molecular methods.	Schneeberger	1998	Cross-sectional	Non probabilistic	Hospital-based	Multicenter	Urban	Antiviral naïve	Prospectively	Netherlands	Sep/1995-Jul/1996	Unclear/ Not reported	Unclear/ Not reported	Unclear/ Not reported	Hemodialysis; Peritoneal dialysis	Indirect ELISA	Anti-HCV	Blood
527	The prevalence and incidence of hepatitis C virus infections among dialysis patients in the Netherlands: a nationwide prospective study.	Schneeberger	2000	Cross-sectional	Non probabilistic	Hospital-based	Multicenter	Urban	Antiviral naïve	Prospectively	Netherlands	Sep/1995-Aug/1998	Unclear/ Not reported	Unclear/ Not reported	Unclear/ Not reported	Hemodialysis; Peritoneal dialysis	Indirect ELISA	Anti-HCV	Blood
528	Prevalence of antibodies to hepatitis C virus in a Dutch group of haemodialysis patients related to risk factors.	Schneeberger	1993	Cross-sectional	Non probabilistic	Hospital-based	Monocenter	Urban	Antiviral naïve	Prospectively	Netherlands	Dec/1991-Jan/1992	52.7	Unclear/ Not reported	60.6	Hemodialysis	Indirect ELISA	Anti-HCV	Blood
529	Prolonged time until seroconversion among hemodialysis patients: the need for HCV PCR.	Schroeter	2005	Cross-sectional	Non probabilistic	Hospital-based	Monocenter	Urban	Antiviral naïve	Prospectively	Germany	1995-2002	62	All ages	Unclear/ Not reported	Hemodialysis	Microparticle enzyme immunoassay	Anti-HCV	Blood
530	GB virus C hepatitis G virus infection in hemodialysis patients: Determination of seroprevalence by a four-antigen recombinant immunoblot assay.	Schroter	1999	Cross-sectional	Non probabilistic	Hospital-based	Monocenter	Urban	Antiviral naïve	Prospectively	Germany	Unclear/ Not reported	59	Adults: over 18 years	59.7	Hemodialysis	Classical RT-PCR	Viral RNA	Blood
531	High percentage of seronegative HCV infections in hemodialysis patients: the need for PCR.	Schröter	1997	Cross-sectional	Non probabilistic	Hospital-based	Multicenter	Urban	Antiviral naïve	Prospectively	Germany	Unclear/ Not reported	Unclear/ Not reported	Unclear/ Not reported	Unclear/ Not reported	Hemodialysis	Indirect ELISA	Anti-HCV	Blood
532	HCV in a group of chronic hemodialysis patients.	Scipioni	1992	Cross-sectional	Non probabilistic	Hospital-based	Monocenter	Urban	Antiviral naïve	Prospectively	Italy	Unclear/ Not reported	59.5	Unclear/ Not reported	57.7	Hemodialysis	Indirect ELISA	Anti-HCV	Blood
533	Adverse impact of hepatitis C virus infection on renal replacement therapy and renal transplant patients in Australia and New Zealand	Scott	2010	Cohort (Baseline data)	Non probabilistic	Hospital-based	Multicenter	Unclear/ Not reported	Unclear/ Not reported	Prospectively	Australia; New Zealand	Jan/1996-Dec/2007	Unclear/ Not reported	Unclear/ Not reported	Unclear/ Not reported	Hemodialysis	Indirect ELISA	Anti-HCV	Blood
534	HCV infections in dialysis patients.	Scotto	1992	Cross-sectional	Non probabilistic	Hospital-based	Multicenter	Urban	Antiviral naïve	Prospectively	Italy	Unclear/ Not reported	Unclear/ Not reported	Unclear/ Not reported	51.4	Hemodialysis	Unclear/ Not reported	Anti-HCV	Unclear/ Not reported
535	Trends in hepatitis C infection among hemodialysis patients in Senegal: results of a decade of prevention.	Seck	2014	Cross-sectional	Non probabilistic	Hospital-based	Multicenter	Urban	Antiviral naïve	Retrospectively	Senegal	2011	43.4	Adults: over 18 years	52.8	Hemodialysis	Indirect ELISA	Anti-HCV	Blood
536	Hepatitis C virus infections in dialysis units: prevalence of HCV-RNA and antibodies to HCV.	Seelig	1994	Cross-sectional	Non probabilistic	Hospital-based	Multicenter	Urban	Antiviral naïve	Prospectively	Germany	Unclear/ Not reported	50.1	Unclear/ Not reported	53.8	Hemodialysis	Classical RT-PCR	Viral RNA	Blood
537	[Prevalence of anti-HCV antibodies and seroconversion incidence in five haemodialysis units in Morocco].	Sekkat	2008	Cross-sectional	Non probabilistic	Hospital-based	Multicenter	Urban	Antiviral naïve	Prospectively	Morocco	Sep/2003-Sep/2004	49	All ages	51.1	Hemodialysis	Indirect ELISA	Anti-HCV	Blood
538	Prevalence of hepatitis C antibodies (HCV) in a dialysis population at one center.	Selgas	1992	Cross-sectional	Non probabilistic	Hospital-based	Multicenter	Urban	Antiviral naïve	Prospectively	Spain	Unclear/ Not reported	Unclear/ Not reported	Unclear/ Not reported	Unclear/ Not reported	Hemodialysis; Peritoneal dialysis	Indirect ELISA	Anti-HCV	Blood
539	Prevalence of hepatitis C virus infection among hemodialysis patients in a single center in Yemen.	Selm	2010	Cross-sectional	Non probabilistic	Hospital-based	Monocenter	Urban	Antiviral naïve	Retrospectively	Yemen	2007	66.5	Unclear/ Not reported	64.7	Hemodialysis	Unclear/ Not reported	Anti-HCV	Unclear/ Not reported
540	Molecular evidence for nosocomial spread of two different hepatitis C virus strains in one hemodialysis unit.	Seme	1997	Cross-sectional	Non probabilistic	Hospital-based	Monocenter	Urban	Antiviral naïve	Prospectively	Slovenia	Dec/1990-Jun/1993	55	Unclear/ Not reported	56.5	Hemodialysis	Indirect ELISA	Anti-HCV	Blood
541	High prevalence of hepatitis C virus infection in hemodialysis patients from one dialysis unit in Slovenia.	Seme	1995	Cross-sectional	Non probabilistic	Hospital-based	Monocenter	Urban	Antiviral naïve	Prospectively	Slovenia	Unclear/ Not reported	Unclear/ Not reported	Unclear/ Not reported	Unclear/ Not reported	Hemodialysis	Indirect ELISA	Anti-HCV	Blood
542	Hepatitis C virus in patients on regular hemodialysis in Beni-	Senosy	2016	Cross-sectional	Non probabilistic	Hospital-based	Multicenter	Unclear/ Not reported	Unclear/ Not reported	Retrospectively	Egypt	May/2015-Jun/2015	46.1	All ages	57.4	Hemodialysis	Unclear/ Not reported	Anti-HCV	Unclear/ Not reported

	Suef Governorate, Egypt																		
543	Hepatitis C infection in hemodialysis patients: Protective against oxidative stress?	Sezer	2006	Cross-sectional	Non probabilistic	Hospital-based	Monocenter	Urban	Antiviral naïve	Prospectively	Turkey	Unclear/ Not reported	49.3	Unclear/ Not reported	60.3	Hemodialysis	Microparticle enzyme immunoassay	Anti-HCV	Blood
544	Seroprevalence of Hepatitis B Virus and Hepatitis C Virus in Patients Undergoing Maintenance Hemodialysis.	Shabbir	2022	Cross-sectional	Non probabilistic	Hospital-based	Monocenter	Urban	Antiviral naïve	Prospectively	Pakistan	Unclear/ Not reported	Unclear/ Not reported	Unclear/ Not reported	Unclear/ Not reported	Hemodialysis	Indirect ELISA	Anti-HCV	Unclear/ Not reported
545	Prevalence of Hepatitis C in Dialysis Patients in Khyber Teaching Hospital Peshawar.	Shah	2022	Cross-sectional	Non probabilistic	Hospital-based	Monocenter	Urban	Antiviral naïve	Prospectively	Pakistan	2017	Unclear/ Not reported	All ages	52.63	Hemodialysis	Immunochromatographic test	Anti-HCV	Blood
546	Prevalence of hepatitis C antibodies among hemodialysis patients in the Western province of saudi arabia.	Shaheen	1995	Cross-sectional	Non probabilistic	Hospital-based	Multicenter	Urban	Antiviral naïve	Prospectively	Saudi Arabia	Unclear/ Not reported	42.9	All ages	52	Hemodialysis	Indirect ELISA	Anti-HCV	Blood
547	Prevalence of HBV, HCV, and HIV Infections among Patients Undergoing Hemodialysis in Fasa, Iran: A Six-Year Follow-up Study.	Shamsdin	2022	Cross-sectional	Non probabilistic	Hospital-based	Monocenter	Urban	Antiviral naïve	Prospectively	Iran	Apr/2014-Mar/2020	56.2	Unclear/ Not reported	62	Hemodialysis	Indirect ELISA	Anti-HCV	Blood
548	Evaluation of immunogenicity of hepatitis B vaccine in hemodialysis patients at Mazandaran Heart Center, Iran.	Shamshirian	2017	Cross-sectional	Non probabilistic	Hospital-based	Multicenter	Urban	Antiviral naïve	Retrospectively	Iran	2015	54.7	All ages	59	Hemodialysis	Unclear/ Not reported	Anti-HCV	Unclear/ Not reported
549	The role of hemodialysis machines dedication in reducing Hepatitis C transmission in the dialysis setting in Iran: a multicenter prospective interventional study.	Shamshirsaz	2004	Cross-sectional	Non probabilistic	Hospital-based	Multicenter	Urban	Antiviral naïve	Prospectively	Iran	Unclear/ Not reported	49.5	All ages	58.7	Hemodialysis	Classical RT-PCR	Viral RNA	Blood
550	High prevalence of hepatitis G virus infection compared with hepatitis C virus infection in patients undergoing chronic hemodialysis.	Sheng	1998	Cross-sectional	Non probabilistic	Hospital-based	Monocenter	Urban	Antiviral naïve	Prospectively	Germany	Unclear/ Not reported	61.5	Adults: over 18 years	58.9	Hemodialysis	Indirect ELISA	Anti-HCV	Blood
551	Prevalence of anti-HCV and HCV viremia in hemodialysis patients in Taiwan.	Sheu	1992	Cross-sectional	Non probabilistic	Hospital-based	Monocenter	Urban	Antiviral naïve	Prospectively	China	Unclear/ Not reported	51	Adults: over 18 years	56.8	Hemodialysis	Indirect ELISA	Anti-HCV	Blood
552	Patient-care practices associated with an increased prevalence of hepatitis C virus infection among chronic hemodialysis patients.	Shimokura	2011	Cross-sectional	Non probabilistic	Hospital-based	Multicenter	Urban	Antiviral naïve	Prospectively	United States of America	Jul/2000-Nov/2001	Unclear/ Not reported	Adults: over 18 years	55.1	Hemodialysis	Indirect ELISA	Anti-HCV	Blood
553	Infection with GB virus C and hepatitis C virus in drug addicts, patients on maintenance hemodialysis, or with chronic liver disease in Nepal.	Shrestha	1997	Cross-sectional	Non probabilistic	Hospital-based	Monocenter	Urban	Antiviral naïve	Prospectively	Nepal	1996	Unclear/ Not reported	Unclear/ Not reported	Unclear/ Not reported	Hemodialysis	Classical RT-PCR	Viral RNA	Blood
554	Virological features of hepatitis C virus infection in hemodialysis patients.	Silini	1993	Cross-sectional	Non probabilistic	Hospital-based	Monocenter	Unclear/ Not reported	Unclear/ Not reported	Prospectively	Italy	Unclear/ Not reported	Unclear/ Not reported	Adults: over 18 years	44.1	Hemodialysis	Indirect ELISA	Anti-HCV	Blood
555	Prevalence of hepatitis C virus (HCV) infection and HCV genotypes of hemodialysis patients in Salvador, Northeastern Brazil.	Silva	2006	Cross-sectional	Non probabilistic	Hospital-based	Multicenter	Unclear/ Not reported	Unclear/ Not reported	Retrospectively	Brazil	Apr/2002-Jul/2002	Unclear/ Not reported	Unclear/ Not reported	Unclear/ Not reported	Hemodialysis	Indirect ELISA	Anti-HCV	Blood
556	A twelve year natural history of hepatitis C virus infection in hemodialyzed patients.	Simon	1994	Cohort (Baseline data)	Non probabilistic	Hospital-based	Monocenter	Unclear/ Not reported	Unclear/ Not reported	Prospectively	France	Jan/1980-Jan/1992	Unclear/ Not reported	Unclear/ Not reported	Unclear/ Not reported	Hemodialysis	Indirect ELISA	Anti-HCV	Blood
557	Seroprevalence of hepatitis B and C viruses in patients with chronic kidney disease in the predialysis stage at a university hospital in Turkey.	Sit	2007	Cross-sectional	Non probabilistic	Hospital-based	Monocenter	Unclear/ Not reported	Unclear/ Not reported	Prospectively	Turkey	Mar/2004-Feb/2005	50.9	Adults: over 18 years	44.4	Pre-Dialysis	Indirect ELISA	Anti-HCV	Blood
558	High prevalence of hepatitis C infection among patients receiving	Sivapalasingam	2002	Cross-sectional	Non probabilistic	Hospital-based	Monocenter	Urban	Unclear/ Not reported	Prospectively	United States of America	Dec/1998	Unclear/ Not reported	Unclear/ Not reported	50	Hemodialysis	Indirect ELISA	Anti-HCV	Blood

	hemodialysis at an urban dialysis center.																		
559	Prevalence of anti-HCV positivity in hemodialysis patients.	Sivrel Arisoy	2000	Cross-sectional	Non probabilistic	Hospital-based	Monocenter	Unclear/ Not reported	Unclear/ Not reported	Prospectively	Turkey	Unclear/ Not reported	49	Adults: over 18 years	38.8	Hemodialysis	Indirect ELISA	Anti-HCV	Blood
560	[Hepatitis B (HBV) and C (HCV) virus infections as an eventual cause of chronic hepatic damage in patients undergoing maintenance hemodialysis].	Slizien	1995	Cross-sectional	Non probabilistic	Hospital-based	Monocenter	Unclear/ Not reported	Unclear/ Not reported	Prospectively	Poland	Unclear/ Not reported	Unclear/ Not reported	Unclear/ Not reported	Unclear/ Not reported	Hemodialysis	Indirect ELISA	Anti-HCV	Blood
561	Differential prevalence of hepatitis C virus subtypes in healthy blood donors, patients on maintenance hemodialysis, and patients with hepatocellular carcinoma in Surabaya, Indonesia	Soetjipto	1996	Cross-sectional	Non probabilistic	Hospital-based	Multicenter	Unclear/ Not reported	Unclear/ Not reported	Prospectively	Indonesia	Unclear/ Not reported	Unclear/ Not reported	Unclear/ Not reported	Unclear/ Not reported	Hemodialysis	Indirect ELISA	Anti-HCV	Blood
562	Risk factors of HCV seroconversion in hemodialysis patients in tabriz, iran.	Somi	2014	Cohort (Baseline data)	Non probabilistic	Hospital-based	Multicenter	Unclear/ Not reported	Unclear/ Not reported	Retrospectively	Iran	Dec/2011-Jan/2012	55.9	All ages	60.4	Hemodialysis	Indirect ELISA	Anti-HCV	Blood
563	Hepatitis C virus genotypes in patients with end-stage renal disease in East Azerbaijan, Iran.	Somi	2008	Cross-sectional	Non probabilistic	Hospital-based	Multicenter	Unclear/ Not reported	Unclear/ Not reported	Prospectively	Iran	Jan/2006-Mar/2006	52.1	All ages	54.3	Hemodialysis; Peritoneal dialysis	Classical RT-PCR	Viral RNA	Blood
564	A cost-identification analysis of screening and surveillance of hepatitis C infection in a prospective cohort of dialysis patients.	Somsouk	2008	Cohort (Baseline data)	Non probabilistic	Hospital-based	Multicenter	Unclear/ Not reported	Unclear/ Not reported	Prospectively	United States of America	Jan/1997-Aug/2004	Unclear/ Not reported	Unclear/ Not reported	Unclear/ Not reported	Hemodialysis	Indirect ELISA	Anti-HCV	Blood
565	Hepatitis C virus antibodies among risk groups in a South African area endemic for hepatitis B virus.	Soni	1993	Cross-sectional	Non probabilistic	Hospital-based	Monocenter	Unclear/ Not reported	Unclear/ Not reported	Prospectively	South Africa	Sep/1991-Nov/1991	Unclear/ Not reported	Unclear/ Not reported	Unclear/ Not reported	Hemodialysis	Indirect ELISA	Anti-HCV	Blood
566	The annual incidence of seroconversion of antibodies to the hepatitis C virus in the hemodialysis population in saudi arabia.	Souqiyyeh	1995	Cross-sectional	Non probabilistic	Hospital-based	Multicenter	Unclear/ Not reported	Unclear/ Not reported	Prospectively	Saudi Arabia	Jan/1993-Dec/1994	44.5	All ages	Unclear/ Not reported	Hemodialysis	Indirect ELISA	Anti-HCV	Blood
567	Hepatitis B and C in the hemodialysis unit of Tocantins, Brazil: serological and molecular profiles.	Souza	2003	Cross-sectional	Non probabilistic	Hospital-based	Monocenter	Unclear/ Not reported	Unclear/ Not reported	Prospectively	Brazil	Jan/2001-Mar/2001	47.6	Adults: over 18 years	62	Hemodialysis	Classical RT-PCR	Viral RNA	Blood
568	Risk of death among chronic dialysis patients infected with hepatitis C virus	Stehman-Breen	1998	Cohort (Baseline data)	Non probabilistic	Hospital-based	Monocenter	Unclear/ Not reported	Unclear/ Not reported	Prospectively	United States of America	1992-1997	Unclear/ Not reported	All ages	58	Hemodialysis; Peritoneal dialysis	Enzyme immunoassay (EIA)	Anti-HCV	Blood
569	Prevalence and risk factors of hepatitis C and B virus infections in hemodialysis patients and their spouses: a multicenter study in Beijing, China.	Su	2013	Cross-sectional	Probabilistic	Hospital-based	Multicenter	Unclear/ Not reported	Unclear/ Not reported	Prospectively	China	Aug/2011-Nov/2011	59.8	Adults: over 18 years	52.6	Hemodialysis	Indirect ELISA	Anti-HCV	Blood
570	Prevalence of hepatitis C virus infection in hemodialysis patients in Sudan.	Suliman	1995	Cross-sectional	Non probabilistic	Hospital-based	Monocenter	Unclear/ Not reported	Unclear/ Not reported	Prospectively	Sudan	Dec/1994	Unclear/ Not reported	Unclear/ Not reported	73.9	Hemodialysis	Indirect ELISA	Anti-HCV	Blood
571	[Occurrence of antibodies against hepatitis C virus (anti-HCV) in patients on long-term hemodialysis].	Sułowicz	1992	Cross-sectional	Non probabilistic	Hospital-based	Monocenter	Unclear/ Not reported	Unclear/ Not reported	Prospectively	Poland	Unclear/ Not reported	Unclear/ Not reported	Unclear/ Not reported	Unclear/ Not reported	Hemodialysis	Unclear/ Not reported	Anti-HCV	Unclear/ Not reported
572	Incidence and patterns of hepatitis C virus seroconversion in a cohort of hemodialysis patients.	Sypsa	2005	Cross-sectional	Non probabilistic	Hospital-based	Multicenter	Unclear/ Not reported	Unclear/ Not reported	Prospectively	Greece	Feb/1993-May/1995	57.2	Unclear/ Not reported	57.7	Hemodialysis	Indirect ELISA	Anti-HCV	Blood
573	An archived serum sample as a clue for identifying the primary source of a nosocomial hepatitis C virus outbreak in a haemodialysis unit.	Szücs	2014	Cross-sectional	Non probabilistic	Hospital-based	Monocenter	Unclear/ Not reported	Unclear/ Not reported	Prospectively	Hungary	Nov/2007-Jan/2008	Unclear/ Not reported	Unclear/ Not reported	53	Hemodialysis	Indirect ELISA	Anti-HCV	Blood
574	Hepatitis C virus infection in chronic haemodialysis patients--	Taal	2000	Cross-sectional	Non probabilistic	Hospital-based	Monocenter	Unclear/ Not reported	Unclear/ Not reported	Prospectively	South Africa	Dec/1992;Dec/1995	Unclear/ Not reported	Unclear/ Not reported	44.6	Hemodialysis	Indirect ELISA	Anti-HCV	Blood

	relationship to blood transfusions and dialyser re-use.																		
575	Prevalence of hepatitis C and B virus infections among hemodialysis patients in Karaj, Iran.	Tajbakhsh	2015	Cross-sectional	Non probabilistic	Hospital-based	Multicenter	Unclear/ Not reported	Unclear/ Not reported	Prospectively	Iran	Feb/2010-Jun/2010	54.8	All ages	56.2	Hemodialysis	Indirect ELISA	Anti-HCV	Blood
576	Prevalence of four blood-borne viruses (HBV, HCV, HTLV-I, HIV-1) among haemodialysis patients in Japan.	Tamura	1992	Cross-sectional	Non probabilistic	Hospital-based	Multicenter	Unclear/ Not reported	Unclear/ Not reported	Prospectively	Japan	May/1990-Oct/1990	52.1	Adults: over 18 years	Unclear/ Not reported	Hemodialysis	Indirect ELISA	Anti-HCV	Blood
577	[Detection of TT virus (TTV) in Japanese hemodialysis (HD) patients].	Tanaka	1999	Cross-sectional	Non probabilistic	Hospital-based	Monocenter	Unclear/ Not reported	Unclear/ Not reported	Prospectively	Japan	Unclear/ Not reported	Unclear/ Not reported	Unclear/ Not reported	Unclear/ Not reported	Hemodialysis	Unclear/ Not reported	Anti-HCV	Unclear/ Not reported
578	Hepatitis E virus infection in hemodialysis patients: a seroepidemiological survey in Iran.	Taremi	2005	Cross-sectional	Non probabilistic	Hospital-based	Multicenter	Unclear/ Not reported	Unclear/ Not reported	Prospectively	Iran	Aug/2004	53.5	Unclear/ Not reported	59	Hemodialysis	Indirect ELISA	Anti-HCV	Blood
579	Prevalence of hepatitis C virus infection in hemodialysis patients.	Taziki	2008	Cross-sectional	Non probabilistic	Hospital-based	Monocenter	Unclear/ Not reported	Unclear/ Not reported	Prospectively	Iran	2001-2006	47.3	Unclear/ Not reported	52.3	Hemodialysis	Unclear/ Not reported	Anti-HCV	Blood
580	Hepatitis B and C in dialysis units in Kosova.	Telaku	2009	Cross-sectional	Non probabilistic	Hospital-based	Multicenter	Unclear/ Not reported	Unclear/ Not reported	Prospectively	Kosovo	Jan/2008-Dec/2008	Unclear/ Not reported	All ages	38.6	Hemodialysis	Microparticle enzyme immunoassay	Anti-HCV	Blood
581	[Hepatitis C virus infection in patients treated with hemodialysis].	Teruel	1990	Cross-sectional	Non probabilistic	Hospital-based	Monocenter	Unclear/ Not reported	Unclear/ Not reported	Prospectively	Spain	Unclear/ Not reported	Unclear/ Not reported	Unclear/ Not reported	Unclear/ Not reported	Hemodialysis	Indirect ELISA	Anti-HCV	Blood
582	Viral hepatitis infections among dialysis patients: Thailand registry report.	Thanachartwet	2007	Cross-sectional	Non probabilistic	Hospital-based	Multicenter	Unclear/ Not reported	Unclear/ Not reported	Retrospectively	Thailand	Jan/2002-Dec/2003	Unclear/ Not reported	All ages	50.8	Hemodialysis; Peritoneal dialysis	Indirect ELISA	Anti-HCV	Blood
583	Occult hepatitis C virus infection during an outbreak in a hemodialysis unit in Thailand.	Thongsawat	2008	Cohort (Baseline data)	Non probabilistic	Hospital-based	Multicenter	Unclear/ Not reported	Unclear/ Not reported	Prospectively	Thailand	Unclear/ Not reported	59	Unclear/ Not reported	55	Hemodialysis	Classical RT-PCR	Viral RNA	Blood
584	The role of dedicated reuse machine for anti HCV positive patients in reducing the incidence of hepatitis C infection in hemodialysis unit: A two year prospective study.	Tjiang	2016	Cohort (Baseline data)	Non probabilistic	Hospital-based	Monocenter	Unclear/ Not reported	Unclear/ Not reported	Prospectively	Indonesia	Dec/2013-Dec/2015	52	Unclear/ Not reported	58.6	Hemodialysis	Unclear/ Not reported	Anti-HCV	Blood
585	High prevalence of hepatitis C virus infection in one dialysis center in Bulgaria.	Todorov	1998	Cross-sectional	Non probabilistic	Hospital-based	Monocenter	Unclear/ Not reported	Unclear/ Not reported	Prospectively	Bulgaria	Unclear/ Not reported	Unclear/ Not reported	Unclear/ Not reported	Unclear/ Not reported	Hemodialysis	Indirect ELISA	Anti-HCV	Blood
586	Risk factors and seroprevalence of hepatitis B and C infections among hemodialysis patients in Tehran	Toosi	2007	Cross-sectional	Non probabilistic	Hospital-based	Multicenter	Unclear/ Not reported	Unclear/ Not reported	Prospectively	Iran	Unclear/ Not reported	52.9	Adults: over 18 years	60	Hemodialysis	Unclear/ Not reported	Anti-HCV	Blood
587	High frequency of antibodies to Hantaan virus and hepatitis C virus in chronic haemodialysis patients. Coincidence or cross-reaction?	Tsianos	1993	Cross-sectional	Non probabilistic	Hospital-based	Monocenter	Unclear/ Not reported	Unclear/ Not reported	Prospectively	Greece	Unclear/ Not reported	Unclear/ Not reported	Adults: over 18 years	59.6	Hemodialysis	Indirect ELISA	Anti-HCV	Blood
588	[Prevalence of hepatitis C virus infection among chronic hemodialysis patients].	Tsuyuguchi	1994	Cross-sectional	Non probabilistic	Hospital-based	Monocenter	Unclear/ Not reported	Unclear/ Not reported	Prospectively	Japan	Unclear/ Not reported	Unclear/ Not reported	Unclear/ Not reported	64.7	Hemodialysis	Indirect ELISA	Anti-HCV	Blood
589	Prevalence and incidence of hepatitis C virus in hemodialysis patients in British Columbia: Follow-up after a possible breach in hemodialysis machines.	Tu	2009	Cohort (Baseline data)	Non probabilistic	Hospital-based	Monocenter	Unclear/ Not reported	Unclear/ Not reported	Prospectively	Canada	Dec/2003-Jun/2004	63	Unclear/ Not reported	59.4	Hemodialysis	Microparticle enzyme immunoassay	Anti-HCV	Blood
590	[Hepatitis E virus seropositivity in hemodialysis patients in Hatay province, Turkey].	Uçar	2009	Cross-sectional	Non probabilistic	Hospital-based	Monocenter	Unclear/ Not reported	Unclear/ Not reported	Prospectively	Turkey	Unclear/ Not reported	55	Adults: over 18 years	58.6	Hemodialysis	Indirect ELISA	Anti-HCV	Blood
591	Risk factors for hepatitis C virus sero-positivity among haemodialysis patients receiving care at kidney centre in a tertiary health	Ummate	2014	Cross-sectional	Non probabilistic	Hospital-based	Monocenter	Urban	Unclear/ Not reported	Prospectively	Nigeria	Unclear/ Not reported	39.9	All ages	68	Hemodialysis	Indirect ELISA	Anti-HCV	Blood

	facility in Maiduguri, Nigeria.																		
592	Prevalence of Hepatitis C virus infection among haemodialysis patients in North-Eastern Nigeria.	Ummate	2013	Cross-sectional	Non probabilistic	Hospital-based	Monocenter	Unclear/ Not reported	Unclear/ Not reported	Prospectively	Nigeria	Unclear/ Not reported	39.9	Adults: over 18 years	68	Hemodialysis	Unclear/ Not reported	Anti-HCV	Blood
593	HBV and HCV infections in relation to the chosen genetic features in hemodialyzed patients.	Urbanowicz	2000	Cross-sectional	Non probabilistic	Hospital-based	Multicenter	Unclear/ Not reported	Unclear/ Not reported	Prospectively	Poland	Unclear/ Not reported	43	All ages	51.5	Hemodialysis	Unclear/ Not reported	Anti-HCV	Unclear/ Not reported
594	Screening of relatives of anti-hepatitis-C virus positive hemodialysed patients (preliminary data).	Urso	1996	Cross-sectional	Non probabilistic	Hospital-based	Monocenter	Urban	Unclear/ Not reported	Prospectively	Italy	Unclear/ Not reported	62.4	Adults: over 18 years	Unclear/ Not reported	Hemodialysis	Immunoblot Assay	Anti-HCV	Unclear/ Not reported
595	PREVALENCE AND RISK FACTORS OF HEPATITIS B AND C VIRUS INFECTIONS AMONG HEMODIALYSIS PATIENTS FROM PRIVATE HEMODIALYSIS UNITS IN SURABAYA, INDONESIA.	Utsumi	2016	Cohort (Baseline data)	Non probabilistic	Hospital-based	Multicenter	Unclear/ Not reported	Unclear/ Not reported	Prospectively	Indonesia	Jul/2012-Apr/2014	50	Adults: over 18 years	63.3	Hemodialysis	Particle Agglutination	Anti-HCV	Blood
596	TT virus infection in hemodialysis patients.	Utsunomiya	1999	Cross-sectional	Probabilistic	Hospital-based	Monocenter	Urban	Unclear/ Not reported	Prospectively	Japan	Mar/1998	62	Adults: over 18 years	42.6	Hemodialysis	Indirect ELISA	Anti-HCV	Blood
597	The role of transfusion-transmitted virus in patients undergoing hemodialysis.	Valtuille	2002	Cross-sectional	Non probabilistic	Hospital-based	Monocenter	Urban	Unclear/ Not reported	Prospectively	Argentina	Unclear/ Not reported	55	Adults: over 18 years	61.33	Hemodialysis	Classical RT-PCR	Viral RNA	Blood
598	Behavior of antibody profile against hepatitis C virus in patients on maintenance hemodialysis.	Vandelli	1992	Cohort (Baseline data)	Non probabilistic	Hospital-based	Multicenter	Urban	Unclear/ Not reported	Prospectively	Italy	Unclear/ Not reported	62	Adults: over 18 years	57.3	Hemodialysis	Indirect ELISA	Anti-HCV	Blood
599	High prevalence of hepatitis C infection among Brazilian hemodialysis patients in Rio de Janeiro: a one-year follow-up study.	Vanderborght	1995	Cohort (Baseline data)	Non probabilistic	Hospital-based	Multicenter	Urban	Unclear/ Not reported	Prospectively	Brazil	1991	Unclear/ Not reported	Unclear/ Not reported	Unclear/ Not reported	Hemodialysis; Peritoneal dialysis	Indirect ELISA	Anti-HCV	Blood
600	Prevalence of isolated hepatitis B core antibody and occult hepatitis B among adult patients initiating hemodialysis at the Philippine General Hospital (PGH) Dialysis Unit.	Velasquez	2011	Cross-sectional	Non probabilistic	Hospital-based	Monocenter	Urban	Antiviral naïve	Prospectively	Australia	Unclear/ Not reported	51.8	Unclear/ Not reported	54	Hemodialysis	Unclear/ Not reported	Anti-HCV	Unclear/ Not reported
601	Detection of hepatitis C virus in patients with terminal renal disease undergoing dialysis in southern Brazil: prevalence, risk factors, genotypes, and viral load dynamics in hemodialysis patients.	Vidales-Braz	2015	Cross-sectional	Non probabilistic	Hospital-based	Multicenter	Urban	Unclear/ Not reported	Prospectively	Brazil	Mar/2012-Aug/2013	Unclear/ Not reported	Unclear/ Not reported	Unclear/ Not reported	Hemodialysis; Peritoneal dialysis	Classical RT-PCR	Viral RNA	Blood
602	Seroprevalence of hepatitis C infection in patients undergoing haemodialysis in a tertiary care centre.	Vinayakumar	2020	Cross-sectional	Non probabilistic	Hospital-based	Monocenter	Unclear/ Not reported	Unclear/ Not reported	Prospectively	India	Unclear/ Not reported	Unclear/ Not reported	Unclear/ Not reported	Unclear/ Not reported	Hemodialysis	Indirect ELISA	Anti-HCV	Blood
603	Epidemiology of hepatitis C virus infection in dialysis units: first-versus second-generation assays.	Vitale	1993	Cross-sectional	Non probabilistic	Hospital-based	Multicenter	Unclear/ Not reported	Unclear/ Not reported	Prospectively	Italy	Unclear/ Not reported	Unclear/ Not reported	Unclear/ Not reported	Unclear/ Not reported	Hemodialysis	Indirect ELISA	Anti-HCV	Blood
604	Infections with hepatitis B and C viruses in patients on maintenance dialysis in Romania and in former communist countries: yellow spots on a blank map?	Vladutiu	2000	Cross-sectional	Non probabilistic	Hospital-based	Monocenter	Urban	Unclear/ Not reported	Prospectively	Romania	1993-1998	Unclear/ Not reported	Adults: over 18 years	Unclear/ Not reported	Hemodialysis	Indirect ELISA	Anti-HCV	Blood
605	A cross-sectional epidemiological study of HBV, HCV, HDV and HEV prevalence in the SubCarpathian and South-Eastern regions of Romania.	Voiculescu	2010	Cross-sectional	Non probabilistic	Hospital-based	Multicenter	Urban/Rural	Unclear/ Not reported	Prospectively	Romania	Oct/2008-Mar/2009	53.7	All ages	48.8	Hemodialysis	Indirect ELISA	Anti-HCV	Blood

606	Follow-up study of hepatitis C virus infection in uremic patients on maintenance hemodialysis for 30 months.	Wang	2000	Cohort (Baseline data)	Non probabilistic	Hospital-based	Monocenter	Urban	Unclear/ Not reported	Prospectively	China	1995-1998	Unclear/ Not reported	All ages	Unclear/ Not reported	Hemodialysis	Classical RT-PCR	Viral RNA	Blood
607	Infection with GB virus C and hepatitis C virus in hemodialysis patients and blood donors in Beijing.	Wang	1997	Cross-sectional	Non probabilistic	Hospital-based	Multicenter	Urban	Unclear/ Not reported	Prospectively	China	Jan/1994-Feb/1994	51	Unclear/ Not reported	60.8	Hemodialysis	Classical RT-PCR	Viral RNA	Blood
608	Mortality in hepatitis C-positive patients treated with peritoneal dialysis	Wang	2008	Cross-sectional	Non probabilistic	Hospital-based	Monocenter	Unclear/ Not reported	Unclear/ Not reported	Retrospectively	China	1996-2005	Unclear/ Not reported	Unclear/ Not reported	42.6	Peritoneal dialysis	Indirect ELISA	Anti-HCV	Blood
609	GB virus C and hepatitis C virus infections in hemodialysis patients in eight Japanese centers.	Watanabe	1997	Case control	Non probabilistic	Hospital-based	Multicenter	Unclear/ Not reported	Unclear/ Not reported	Prospectively	Japan	May/1995-Sep/1995	57	Unclear/ Not reported	62.6	Hemodialysis	Classical RT-PCR	Viral RNA	Blood
610	Seroprevalence of HCV, HAV, HBV, HDV, HCMV and HIV in high risk groups/Frankfurt a.M., Germany.	Weber	1995	Cross-sectional	Non probabilistic	Hospital-based	Multicenter	Urban	Unclear/ Not reported	Prospectively	Germany	Aug/1991-Apr/1993	51.5	Adults: over 18 years	58.5	Hemodialysis	Indirect ELISA	Anti-HCV	Blood
611	[Detection of serum HCV RNA and HBV DNA in patients on hemodialysis].	Wei	1996	Cross-sectional	Non probabilistic	Hospital-based	Multicenter	Urban	Unclear/ Not reported	Prospectively	China	Unclear/ Not reported	Unclear/ Not reported	Unclear/ Not reported	Unclear/ Not reported	Hemodialysis	Indirect ELISA	Anti-HCV	Blood
612	Evolutionary seroepidemiology of viral hepatitis and the gap in hepatitis C care cascades among uraemic patients receiving haemodialysis in Taiwan-the Formosa-Like Group.	Wei	2021	Cross-sectional	Non probabilistic	Hospital-based	Multicenter	Urban/Rural	Unclear/ Not reported	Prospectively	China	2019	64.3	Unclear/ Not reported	52.9	Hemodialysis	Indirect ELISA	Anti-HCV	Blood
613	Prevalence of antibodies to hepatitis C virus (anti-HCV) in different populations in Taiwan.	Wu	1991	Cross-sectional	Non probabilistic	Hospital-based	Multicenter	Unclear/ Not reported	Unclear/ Not reported	Prospectively	China	Unclear/ Not reported	Unclear/ Not reported	Unclear/ Not reported	Unclear/ Not reported	Hemodialysis	Indirect ELISA	Anti-HCV	Blood
614	[Detection of hepatitis C virus antigen in hemodialysis patients].	Wu	2009	Cross-sectional	Non probabilistic	Hospital-based	Multicenter	Unclear/ Not reported	Unclear/ Not reported	Prospectively	China	Unclear/ Not reported	Unclear/ Not reported	Unclear/ Not reported	Unclear/ Not reported	Hemodialysis	Direct ELISA	Viral antigen	Unclear/ Not reported
615	Seroprevalence of hepatitis B, C and D viral among hemodialysis patients in Tehran.	Yadegarynia	2017	Cross-sectional	Non probabilistic	Hospital-based	Multicenter	Urban/Rural	Unclear/ Not reported	Prospectively	Iran	2016	53.4	All ages	59.1	Hemodialysis	Indirect ELISA	Anti-HCV	Blood
616	Prevalence of occult hepatitis B and hepatitis C virus infections in Turkish hemodialysis patients.	Yakaryilmaz	2006	Cross-sectional	Non probabilistic	Hospital-based	Monocenter	Urban	Unclear/ Not reported	Prospectively	Turkey	Unclear/ Not reported	49	All ages	54.3	Hemodialysis	Indirect ELISA	Anti-HCV	Blood
617	Long term survey of hepatitis C virus infection in hemodialysis units in Fukuoka, Japan.	Yamaji	1996	Cohort (Baseline data)	Non probabilistic	Hospital-based	Multicenter	Urban	Unclear/ Not reported	Prospectively	Japan	1989-1994	53	Adults: over 18 years	54.4	Hemodialysis	Indirect ELISA	Anti-HCV	Blood
618	Seroprevalence of antibodies to the hepatitis C virus in Singapore.	Yap	1991	Cross-sectional	Non probabilistic	Hospital-based	Multicenter	Unclear/ Not reported	Unclear/ Not reported	Prospectively	Singapore	Unclear/ Not reported	44	All ages	Unclear/ Not reported	Hemodialysis	Indirect ELISA	Anti-HCV	Blood
619	High prevalence of hepatitis C virus antibody in patients with chronic renal failure at the start of hemodialysis therapy.	Yonemura	1996	Cross-sectional	Non probabilistic	Hospital-based	Multicenter	Unclear/ Not reported	Unclear/ Not reported	Prospectively	Japan	Unclear/ Not reported	63.3	Unclear/ Not reported	65.4	Hemodialysis	Unclear/ Not reported	Anti-HCV	Unclear/ Not reported
620	Hepatitis C virus in chronic hemodialysis patients with non-A, non-B hepatitis.	Yoshida	1992	Cohort (Baseline data)	Probabilistic	Hospital-based	Multicenter	Unclear/ Not reported	Unclear/ Not reported	Prospectively	Brazil	1989	46	Unclear/ Not reported	31.3	Hemodialysis	Indirect ELISA	Anti-HCV	Unclear/ Not reported
621	Antibodies against non-structural c100/3 and structural core antigen of hepatitis C virus (HCV) in hemodialysis patients.	Yoshida	1993	Cohort (Baseline data)	Probabilistic	Hospital-based	Monocenter	Urban	Unclear/ Not reported	Prospectively	Brazil	Jun/1990-Dec/1990	Unclear/ Not reported	All ages	55.1	Hemodialysis	Indirect ELISA	Anti-HCV	Blood
622	Nosocomial Transmission of Hepatitis B Surface Antigen and Anti-Hepatitis C Virus among Hemodialysis Patients.	Yousif	2022	Cross-sectional	Non probabilistic	Hospital-based	Monocenter	Urban	Unclear/ Not reported	Retrospectively	Sudan	Aug/2021-Jan/2022	Unclear/ Not reported	Adults: over 18 years	Unclear/ Not reported	Hemodialysis	Indirect ELISA	Anti-HCV	Blood
623	Hepatitis C virus and carpal tunnel syndrome in hemodialysis patients: a single	Yu	2020	Cross-sectional	Non probabilistic	Hospital-based	Monocenter	Urban	Unclear/ Not reported	Prospectively	China	Jul/2017-Sep/2017	58.5	Adults: over 18 years	61.9	Hemodialysis	Chemiluminescent immunoassay	Anti-HCV	Blood

	center cross-sectional study.																		
624	Establishment of an outreach, grouping healthcare system to achieve microelimination of HCV for uremic patients in haemodialysis centres (ERASE-C).	Yu	2021	Cross-sectional	Non probabilistic	Hospital-based	Monocenter	Urban	Antiviral naïve	Prospectively	China	Unclear/ Not reported	Unclear/ Not reported	Unclear/ Not reported	Unclear/ Not reported	Hemodialysis	Unclear/ Not reported	Anti-HCV	Unclear/ Not reported
625	Quality control measures for lowering the seroconversion rate of hemodialysis patients with hepatitis B or C virus.	Yuan	2012	Cohort (Baseline data)	Non probabilistic	Hospital-based	Multicenter	Unclear/ Not reported	Unclear/ Not reported	Prospectively	China	2007-2010	54.6	Adults: over 18 years	59.72	Hemodialysis	Unclear/ Not reported	Anti-HCV	Unclear/ Not reported
626	Seroprevalence of Hepatitis Viruses B, C, D and HIV Infection Among Hemodialysis Patients in Kerman Province, South-East Iran.	Zahedi	2012	Cross-sectional	Non probabilistic	Hospital-based	Multicenter	Unclear/ Not reported	Unclear/ Not reported	Prospectively	Iran	2010	51	Unclear/ Not reported	59.6	Hemodialysis	Indirect ELISA	Anti-HCV	Blood
627	Prevalence of seroconversion of hepatitis C virus among hemodialysis patients in Menoufia Governorate, Egypt.	Zahran	2014	Cohort (Baseline data)	Non probabilistic	Hospital-based	Multicenter	Unclear/ Not reported	Unclear/ Not reported	Prospectively	Egypt	Unclear/ Not reported	Unclear/ Not reported	Unclear/ Not reported	Unclear/ Not reported	Hemodialysis	Unclear/ Not reported	Anti-HCV	Unclear/ Not reported
628	[Low prevalence of hepatitis G infection in dialysis patients in Israel].	Zamir	1999	Cross-sectional	Non probabilistic	Hospital-based	Multicenter	Unclear/ Not reported	Unclear/ Not reported	Prospectively	Israel	Unclear/ Not reported	Unclear/ Not reported	Unclear/ Not reported	Unclear/ Not reported	Hemodialysis; Peritoneal dialysis	Classical RT-PCR	Viral RNA	Blood
629	Hepatitis C virus seroconversion and genotype prevalence in patients and staff on chronic hemodialysis.	Zamir	1999	Cohort (Baseline data)	Non probabilistic	Hospital-based	Monocenter	Unclear/ Not reported	Unclear/ Not reported	Prospectively	Israel	Unclear/ Not reported	Unclear/ Not reported	Unclear/ Not reported	Unclear/ Not reported	Hemodialysis	Indirect ELISA	Anti-HCV	Blood
630	The prevalence of hepatitis C virus antibodies among hemodialysis patients.	Zeldis	1990	Cross-sectional	Non probabilistic	Hospital-based	Multicenter	Urban	Unclear/ Not reported	Prospectively	United States of America	Unclear/ Not reported	Unclear/ Not reported	Unclear/ Not reported	Unclear/ Not reported	Hemodialysis	Indirect ELISA	Anti-HCV	Blood
631	Phylogenetic analysis of hepatitis C virus isolates from hemodialysis patients.	Zeuzem	1996	Cross-sectional	Non probabilistic	Hospital-based	Monocenter	Rural	Unclear/ Not reported	Prospectively	Germany	Unclear/ Not reported	56.7	Adults: over 18 years	56.3	Hemodialysis	Indirect ELISA	Anti-HCV	Blood
632	Tumor Necrosis Factor-alpha Promoter Gene Polymorphisms Are Not Associated with Hepatitis C Virus Infection in Chinese Hemodialysis Patients.	Zhang	2011	Cross-sectional	Non probabilistic	Hospital-based	Multicenter	Unclear/ Not reported	Antiviral naïve	Prospectively	China	Unclear/ Not reported	52.6	Unclear/ Not reported	62.9	Hemodialysis	Indirect ELISA	Anti-HCV	Blood
633	Baseline data report of the China Dialysis Outcomes and Practice Patterns Study (DOPPS).	Zhao	2021	Cohort (Baseline data)	Probabilistic	Hospital-based	Multicenter	Unclear/ Not reported	Unclear/ Not reported	Prospectively	China	Unclear/ Not reported	58.7	Adults: over 18 years	54.6	Hemodialysis	Unclear/ Not reported	Anti-HCV	Unclear/ Not reported
634	Prevalence of HCV infection in hemodialysis patients of South Khorasan in comparison with HBV, HDV, HTLV I/II, And HIV infection	Ziaee	2014	Cross-sectional	Non probabilistic	Hospital-based	Monocenter	Urban	Antiviral naïve	Prospectively	Iran	2010	54.93	Unclear/ Not reported	68.3	Hemodialysis	Real-time RT-PCR	Viral RNA	Blood
