# Supplementary material for: Global epidemiology of hepatitis C virus in dialysis patients: A systematic review and meta-analysis
Source: PLoS One. 2024 Feb 8;19(2):e0284169. doi: 10.1371/journal.pone.0284169 (PMC10852299; doi:10.1371/journal.pone.0284169)
Supplement: S7 Table — (PDF) [file pone.0284169.s008.pdf]

S7 Table: Risk of bias assessment

ID	Title	Author	Year of publication	Was the study's target population a close representation of the national population in relation to HCV prevalence?	Was the sampling frame a true or close representation of the target population?	Was some form of random selection used to select the sample, OR was acensus undertaken?	Were data collected directly from the subjects (as opposed to a proxy)?	Was an acceptable inclusion criteria definition used in the study?	Did the author calculate and respect the expected sample size?	Was the HCV detection assay shown to have reliability and validity?	Was the same mode of data collection used for all subjects?	Was the length of the study period > or = 1 year?	Were the numerator(s) and denominator(s) for the HCV prevalence?	Score	Risk of bias
1	Molecular evidence of nosocomial transmission of hepatitis C virus in a haemodialysis unit.	Abacioglu	2000	No	Yes	No	Yes	Yes	No	Yes	Yes	Unclear	Yes	6	Moderate risk of bias
2	Effectiveness of direct-acting antivirals in Hepatitis C virus infection in haemodialysis patients.	Abad	2017	No	Yes	No	Yes	Yes	No	Yes	Yes	Unclear	Yes	6	Moderate risk of bias
3	Prevalence of hepatitis C virus antibodies in hospital personnel.	Abb	1991	No	Yes	No	Yes	Yes	No	Yes	Yes	Unclear	Yes	6	Moderate risk of bias
4	Hepatitis C virus infection in hemodialysis patients in qatar.	Abboud	1995	No	Yes	No	Yes	Yes	No	Yes	Yes	Unclear	Yes	6	Moderate risk of bias
5	Prevalence of hepatitis c virus in haemodialysis patients, in sudan.	Abd Alrahman	2015	No	Yes	No	Yes	Yes	No	Yes	Yes	No	Yes	6	Moderate risk of bias
6	Hepatitis C viral prevalence and seroconversion in Moroccan hemodialysis units: eight year follow up	Abdelaali	2013	No	Yes	No	Yes	Yes	No	Yes	Yes	Yes	Yes	7	Low risk of bias
7	Detection of anti-hepatitis C-virus antibodies and hepatitis C-virus RNA in Lebanese hemodialysis patients.	Abdelnour	1997	No	Yes	No	Yes	Yes	No	Yes	Yes	Unclear	Yes	6	Moderate risk of bias
8	Epidemiological and clinical aspects of hepatitis C virus infection in the Russian Republic of Daghestan.	Abdourakhmanov	1998	No	Yes	No	Yes	Yes	No	Yes	Yes	Yes	Yes	7	Low risk of bias
9	Hepatitis C virus genotypes and hepatitis G virus in hemodialysis patients from Syria: identification of two novel hepatitis C virus subtypes.	Abdulkarim	2012	No	Yes	No	Yes	Yes	No	Yes	Yes	Unclear	Yes	6	Moderate risk of bias
10	Current Status of Hepatitis C Virus-Infected Maintenance Hemodialysis Patients in Japan.	Abe	2018	No	Yes	No	Yes	Yes	No	Yes	Yes	Unclear	Yes	6	Moderate risk of bias
11	Incidence and prevalence of hepatitis B and hepatitis C viruses in hemodialysis patients in Lebanon.	Abou Rached	2016	No	Yes	No	Yes	Yes	No	Yes	Yes	Yes	Yes	7	Low risk of bias
12	Association of conjunctival and corneal calcification with vascular calcification among hepatitis-C-seropositive hemodialysis patients.	AbouSeif	2016	No	Yes	No	Yes	Yes	No	Yes	Yes	Unclear	Yes	6	Moderate risk of bias
13	The effect of chemical and heat disinfection of the hemodialysis machines on the spread of hepatitis C virus infection: a prospective study.	Abu-Aisha	1995	No	Yes	No	Yes	Yes	No	Yes	Yes	Unclear	Yes	6	Moderate risk of bias
14	Correlation of hepatitis C antibody levels in gingival crevicular fluid and saliva of hepatitis C seropositive hemodialysis patients.	Açıkgöz	2009	No	Yes	No	Yes	Yes	No	Yes	Yes	Unclear	Yes	6	Moderate risk of bias
15	Quality of life in hemodialysis patients: hepatitis C virus infection makes sense.	Afsar	2009	No	Yes	No	Yes	Yes	No	Yes	Yes	Unclear	Yes	6	Moderate risk of bias
16	Hepatitis C virus infection during haemodialysis in India.	Agarwal	1999	No	Yes	No	Yes	Yes	No	Yes	Yes	Unclear	Yes	6	Moderate risk of bias
17	HCV infection during renal replacement therapy: should we dialyze all HCV-positive patients on dedicated machines?	Agarwal	1998	No	Yes	No	Yes	Yes	No	Yes	Yes	Unclear	Yes	6	Moderate risk of bias
18	Hepatitis C virus infection in haemodialysis: the 'no-isolation' policy should not be generalized	Agarwal	2009	No	Yes	No	Yes	Yes	Yes	Yes	Yes	Yes	Yes	8	Low risk of bias
19	Prevalence of antibodies against the hepatitis C virus among voluntary blood donors at a makkah hospital.	Ahmad	1995	No	Yes	No	Yes	Yes	Yes	Yes	Yes	Unclear	Yes	7	Low risk of bias
20	Assessing the prevalence of HBV and HCV infections in children under going hemodialysis and the related risk factors in a children's Medical Center: PS132.	Ahmadi	2017	No	Yes	No	Yes	Yes	No	Yes	Yes	Yes	Yes	7	Low risk of bias
21	Prevalence of hepatitis C virus in maintenance haemodialysis patients - A prospective study.	Ahmed	2003	No	Yes	Unclear	Yes	Yes	Unclear	Yes	Yes	Unclear	Yes	6	Moderate risk of bias
22	[Hepatitis C virus infection in hemodialysis patients in General Hospital Gracanica].	Ahmetagić	2016	No	Yes	No	Yes	Yes	No	Yes	Yes	Unclear	Yes	6	Moderate risk of bias
23	Hepatitis C infection in risk groups.	Ahmetagić	2006	No	Yes	No	Yes	Yes	No	Yes	Yes	Yes	Yes	7	Low risk of bias
24	Seroprevalence and risk factors for hepatitis C.	Akhmouch	2009	No	Yes	No	Yes	Yes	No	Yes	Yes	Unclear	Yes	6	Moderate risk of bias
25	Prevalence of anti-HCV among haemodialysis patients in Turkey: a multicentre study.	Akpolat	1995	No	Yes	No	Yes	Yes	No	Yes	Yes	No	Yes	6	Moderate risk of bias
26	Hepatitis C virus infection in hemodialysis patients: comparison of two new hepatitis C antibody assays with a second-generation assay.	al Meshari	1994	No	Yes	No	Yes	Yes	No	Yes	Yes	Unclear	Yes	6	Moderate risk of bias
27	Seropositivity to hepatitis C virus in Saudi haemodialysis patients.	al Nasser	1992	No	Yes	No	Yes	Yes	No	Yes	Yes	Unclear	Yes	6	Moderate risk of bias
28	The prevalence of hepatitis C virus antibodies among hemodialysis patients in jeddah area, saudi arabia.	Al Shohaib	1995	No	Yes	No	Yes	Yes	No	Yes	Yes	Unclear	Yes	6	Moderate risk of bias
29	Anti-HCV positive hemodialysis patients: clinical, biochemical, histologic and virologic study and a proposed management scheme.	Al Traif	2000	No	Yes	No	Yes	Yes	No	Yes	Yes	Unclear	Yes	6	Moderate risk of bias
30	Hepatitis B and C prevalence among hemodialysis patients in the West Bank hospitals, Palestine.	Al Zabadi	2016	No	Yes	No	Yes	Yes	No	Yes	Yes	Unclear	Yes	6	Moderate risk of bias
31	Hepatitis B and C infection in haemodialysis patients in Libya: prevalence, incidence and risk factors.	Alashek	2012	No	Yes	No	Yes	Yes	No	Yes	Yes	No	Yes	6	Moderate risk of bias
32	Prevalence of hepatitis C virus infection and related risk factors among Iranian haemodialysis patients.	Alavian	2003	No	Yes	Yes	Yes	Yes	No	Yes	Yes	Unclear	Yes	7	Low risk of bias
33	[Spanish extrahospitalary hemodialysis centers survey].	Albalate	2007	No	Yes	No	Yes	Yes	No	Yes	Yes	Unclear	Yes	6	Moderate risk of bias
34	Prevalence and risk factors of hepatitis C virus infection in hemodialysis patients from one center in Recife, Brazil.	Albuquerque	2005	No	Yes	No	Yes	Yes	No	Yes	Yes	Unclear	Yes	6	Moderate risk of bias

35	Prevalence of antibodies to hepatitis C virus among Omani patients with renal disease.	al-Dhahry	1993	No	Yes	No	Yes	Yes	No	Yes	Yes	Unclear	Yes	6	Moderate risk of bias
36	Hepatitis C virus infection in chronic haemodialysis patients, a clinicopathologic study.	Alfurayh	1992	No	Yes	No	Yes	Yes	No	Yes	Yes	Yes	Yes	7	Low risk of bias
37	Hepatitis C Virus Sero-status in Hemodialysis Patients Returning from Holiday: Another Risk Factor for HCV Transmission.	Al-Ghamdi	2001	No	Yes	No	Yes	Yes	No	Yes	Yes	Yes	Yes	7	Low risk of bias
38	Prevalence and risk factors of hepatitis B and C viruses among haemodialysis patients: a multicentric study.	Ali	2019	No	Yes	No	Yes	Yes	No	Yes	Yes	No	Yes	6	Moderate risk of bias
39	Hepatitis B Virus and Hepatitis C Virus in Hemodialysis Patients: A Prevalence Study from Dialysis Centers in El-Beyda and Almarj-Libya	Ali	2017	No	Yes	No	Yes	Yes	No	Yes	Yes	Yes	Yes	7	Low risk of bias
40	Hepatitis C virus (HCV) infection in hemodialysis patients in the south of Jordan.	Al-Jamal	2009	No	Yes	No	Yes	Yes	No	Yes	Yes	No	Yes	6	Moderate risk of bias
41	Hepatitis B and C Viral Infections among Dialysis Patients and Related Factors of Dialysis Centres in Saudi Arabia.	Aljarallah	2022	No	Yes	No	Yes	Yes	No	Unclear	Yes	Yes	Yes	6	Moderate risk of bias
42	Hepatitis C virus infection among patients on hemodialysis in jeddah: a single center experience.	Al-Jiffri	2003	No	Yes	Yes	Yes	Yes	No	Yes	Yes	Unclear	Yes	7	Low risk of bias
43	Seroprevalence of hepatitis C virus and hepatitis B virus among dialysis patients in Bahrain and Saudi Arabia.	Almawi	2004	No	Yes	No	Yes	Yes	No	Yes	Yes	Unclear	Yes	6	Moderate risk of bias
44	Monitoring hepatitis C infection in a major Swedish nephrology unit and molecular resolution of a new case of nosocomial transmission.	Almroth	2010	No	Yes	No	Yes	Yes	No	Yes	Yes	Yes	Yes	7	Low risk of bias
45	Detection and prevention of hepatitis C in dialysis patients and renal transplant recipients. A long-term follow up (1989-January 1997).	Almroth	2002	No	Yes	No	Yes	Yes	No	Yes	Yes	Yes	Yes	7	Low risk of bias
46	Seropositivity to hepatitis C virus (HCV) in Saudi children with chronic renal failure maintained on haemodialysis.	al-Mugeiren	1992	No	Yes	No	Yes	Yes	No	Yes	Yes	Unclear	Yes	6	Moderate risk of bias
47	Hepatitis C virus infection in two groups of paediatric patients: one maintained on haemodialysis and the other on continuous ambulatory peritoneal dialysis.	al-Mugeiren	1996	No	Yes	No	Yes	Yes	No	Yes	Yes	Unclear	Yes	6	Moderate risk of bias
48	Hepatitis C virus infection among hemodialysis patients in the eastern region of saudi arabia.	Al-Muhanna	1995	No	Yes	No	Yes	Yes	No	Yes	Yes	Unclear	Yes	6	Moderate risk of bias
49	Prevalence rate of hepatitis c virus (HCV) and hepatitis b virus (HBV) infection in iraqi patients on hemodialysis: Cross sectional study.	Al-Muramdy	2020	No	Yes	No	Yes	Yes	No	Yes	Yes	Unclear	Yes	6	Moderate risk of bias
50	Antibodies to hepatitis C virus in patients on haemodialysis.	Alonso	1991	No	Yes	No	Yes	Yes	No	Yes	Yes	Unclear	Yes	6	Moderate risk of bias
51	Effect of hepatitis C virus on hemoglobin and hematocrit levels in saudi hemodialysis patients.	Alsaran	2009	No	Yes	No	Yes	Yes	No	Yes	Yes	Unclear	Yes	6	Moderate risk of bias
52	Prevalence of blood borne viruses in the dialysis unit, mubarak Al-Kabeer hospital, kuwait.	Altawalah	2015	No	Yes	No	Yes	Yes	No	Yes	Yes	Unclear	Yes	6	Moderate risk of bias
53	Human herpesvirus 6 infection in hemodialysis and peritoneal dialysis patients.	Altay	2011	No	Yes	No	Yes	Yes	No	Yes	Yes	Unclear	Yes	6	Moderate risk of bias
54	[Investigation of hemodialysis patients in terms of the presence of occult hepatitis B].	Altindiş	2007	No	Yes	No	Yes	Yes	No	Yes	Yes	Unclear	Yes	6	Moderate risk of bias
55	Hepatitis C virus in populations at risk for infection.	Alve-Castillo	2007	No	Yes	No	Yes	Yes	No	Yes	Yes	Unclear	Yes	6	Moderate risk of bias
56	Architect hepatitis c virus (HCV) core antigen test: A HCV rna screening alternative in end-stage renal disease (ESRD) and hemodialysis patients?	Alves	2018	No	Yes	No	Yes	Yes	No	Yes	Yes	Unclear	Yes	6	Moderate risk of bias
57	Liver disease in dialysis patients with antibodies to hepatitis C virus.	al-Wakeel	1996	No	Yes	No	Yes	Yes	No	Yes	Yes	Unclear	Yes	6	Moderate risk of bias
58	Prevalence and associated factors of hepatitis C virus infection among renal disease patients on maintenance hemodialysis in three health centers in Aden, Yemen: a cross sectional study.	Aman	2015	No	Yes	No	Yes	Yes	No	Yes	Yes	Yes	Yes	7	Low risk of bias
59	Hepatitis C virus infection in a Moroccan hemodialysis unit: Prevalence and risk factors [1].	Amar	2005	No	Yes	No	Yes	Yes	Yes	Yes	Yes	Unclear	Yes	7	Low risk of bias
60	Hepatitis C in Lithuania: incidence, prevalence, risk factors and viral genotypes.	Ambrozaitis	1995	No	Yes	No	Yes	Yes	No	Yes	Yes	No	Yes	6	Moderate risk of bias
61	[Epidemiology of chronic hepatitis B and C among dialysis patients in Switzerland].	Ambühl	2000	Yes	Yes	Yes	Yes	Yes	No	Yes	Yes	Unclear	Yes	8	Low risk of bias
62	Hepatitis C virus status in hemodialysis patients in Menoufia Government, Egypt, five years apart: Do we have any improvement?	Amin Elzorkany	2017	No	Yes	No	Yes	Yes	No	Yes	Yes	No	No	5	Moderate risk of bias
63	Seroprevalence of hepatitis C and risk factors in haemodialysis patients in Guilan, Islamic Republic of Iran.	Amiri	2005	No	Yes	No	Yes	Yes	No	Yes	Yes	No	No	5	Moderate risk of bias
64	Association of anti-HCV sero-prevalence with blood transfusion and practice of haemodialysis from multiple centres in patients on maintenance haemodialysis.	Amjad	2020	No	Yes	No	Yes	Yes	No	Yes	Yes	No	No	5	Moderate risk of bias
65	s	Amorim	2010	No	Yes	No	Yes	Yes	No	Yes	Yes	No	Yes	6	Moderate risk of bias
66	Prevalence of hepatitis C virus infection in thalassemia and haemodialysis patients in north Iran-Rasht.	Ansar	2002	No	Yes	No	Yes	Yes	No	Yes	Yes	Yes	Yes	7	Low risk of bias
67	Recombinant γ-interferon as adjuvant to hepatitis B vaccine in hemodialysis patients.	Antonio Quiroga	1990	No	Yes	Yes	Yes	Yes	No	Yes	Yes	No	Yes	7	Low risk of bias
68	Prevalence of Hepatitis B and Hepatitis C Infection among Patients Undergoing Dialysis.	Anwar	2016	No	Yes	No	Yes	Yes	No	Yes	Yes	Unclear	Yes	6	Moderate risk of bias
69	HBV-DNA in hemodialysis patients infected by HCV.	Arababadi	2009	No	Yes	No	Yes	Yes	No	Yes	Yes	Unclear	Yes	6	Moderate risk of bias
70	[Nosocomial transmission of the hepatitis C virus in hemodialysis: monitors, personnel, or both?].	Arenas	2001	No	Yes	No	Yes	Yes	Unclear	Yes	Yes	Unclear	Yes	6	Moderate risk of bias

71	Peculiarities of the Clinical Course of Chronic Viral Hepatitis C in the Background of End-Stage Chronic Renal Insufficiency.	Aripkhodjayeva	2020	No	Yes	No	Yes	Yes	No	Yes	Yes	Unclear	Yes	6	Moderate risk of bias
72	Hemodialysis access surgery - is there an increased risk of acquiring hepatitis C virus compared to other elective vascular interventions?	Assadian	2008	No	Yes	No	Yes	Yes	Unclear	Yes	Yes	Unclear	Yes	6	Moderate risk of bias
73	Prevalence of hepatitis C and B infection and HC V genotypes among hemodialysis patients in Khuzestan province, southwest Iran.	Assarehzadegan	2009	No	Yes	No	Yes	Yes	No	Yes	Yes	Yes	Yes	7	Low risk of bias
74	Effectiveness of universal precautions in limiting nosocomial transmission of hepatitis C virus in haemodialysis units.	Aucella	1996	No	Yes	No	Yes	Yes	No	Yes	Yes	Yes	Yes	7	Low risk of bias
75	Systematic monitor disinfection is effective in limiting HCV spread in hemodialysis.	Aucella	2000	No	Yes	No	Yes	Yes	No	Yes	Yes	Yes	Yes	7	Low risk of bias
76	Hepatitis C virus infection in hemodialysis patients from Tunisia: national survey by serologic and molecular methods.	Ayed	2003	Yes	Yes	Yes	Yes	Yes	Yes	Yes	Yes	No	Yes	9	Low risk of bias
77	Prevalence and significance of antibodies to hepatitis C virus among Saudi haemodialysis patients.	Ayoola	1991	No	Yes	No	Yes	Yes	No	Yes	Yes	Unclear	Yes	6	Moderate risk of bias
78	[Prevalence and risk factors of hepatitis C virus infection in chronic hemodialysis patients at the University Hospital of Point G, Bamako, Mali].	Baby	2011	No	Yes	No	Yes	Yes	No	Yes	Yes	No	Yes	6	Moderate risk of bias
79	Hepatitis C virus antibodies in high-risk Saudi groups.	Bahakim	1991	No	Yes	No	Yes	Yes	No	Yes	Yes	Unclear	Yes	6	Moderate risk of bias
80	Seroprevalence of Anti HCV Ab in Healthy Voluntary BloodDonors and in High Risk Individuals.	Baheti	2000	No	Yes	Unclear	Yes	Yes	Unclear	Yes	Yes	Unclear	Yes	6	Moderate risk of bias
81	Prevalence of occult hepatitis C infection in chronic hemodialysis and kidney transplant patients.	Baid-Agrawal	2014	No	Yes	No	Yes	Yes	No	Yes	Yes	No	Yes	6	Moderate risk of bias
82	Hepatitis C risk factor for patients submitted to dialysis.	Baldessar	2007	No	Yes	No	Yes	Yes	No	Yes	Yes	No	Yes	6	Moderate risk of bias
83	Prevalence of HCV Infection Among Hemodialysis Patients in Lanzhou of Northwestern China.	Bao	2022	No	Yes	Yes	Yes	Yes	No	Unclear	Yes	No	Yes	6	Moderate risk of bias
84	Performance of point of care assays for hepatitis B and C viruses in chronic kidney disease patients.	Barbosa	2018	No	Yes	No	Yes	Yes	No	Yes	Yes	Unclear	Yes	6	Moderate risk of bias
85	Prevalence of hepatitis C virus in dialysis patients in Spain.	Barril	1995	No	Yes	No	Yes	Yes	No	Yes	Yes	Unclear	Yes	6	Moderate risk of bias
86	Detection of HGV in serum and peripheral blood mononuclear cells of maintenance haemodialysis patients.	Basaras	1999	No	Yes	No	Yes	Yes	No	Yes	Yes	Unclear	Yes	6	Moderate risk of bias
87	Prevalence of hepatitis C in patients with renal disease undergoing hemodialysis treatment.	Bastiani	2014	No	Yes	No	Yes	Yes	No	Unclear	Unclear	Yes	Yes	5	Moderate risk of bias
88	Anti-ENA antibody profile in hepatitis C patients undergoing hemodialysis.	Batchoun	2011	No	Yes	No	Yes	Yes	No	Yes	Yes	Unclear	Yes	6	Moderate risk of bias
89	Epidemiology and cost of haemodialysis in Jordan.	Batieha	2007	No	Yes	No	Yes	Yes	No	Yes	Yes	No	Yes	6	Moderate risk of bias
90	Hepatitis C virus infection in Jordanian haemodialysis units: serological diagnosis and genotyping.	Bdour	2002	No	Yes	No	Yes	Yes	No	Yes	Yes	Unclear	Yes	6	Moderate risk of bias
91	HGV infection in dialysis patients.	Beccari	1996	No	Yes	No	Yes	Yes	No	Yes	Yes	Unclear	Yes	6	Moderate risk of bias
92	[High prevalence and incidence of hepatitis C virus infections among dialysis patients in the East-Centre of Tunisia].	Ben Othman	2004	No	Yes	No	Yes	Yes	No	Yes	Yes	Yes	Yes	7	Low risk of bias
93	Virological markers profile in hemodialysis patients.	Benghanem	2009	No	Yes	No	Yes	Yes	No	Yes	Yes	Unclear	Yes	6	Moderate risk of bias
94	Prevalence of hepatitis C virus antibodies in hemodialysis patients in madinah Al munawarah.	Bernieh	1995	No	Yes	No	Yes	Yes	No	Yes	Yes	No	Yes	6	Moderate risk of bias
95	Prevalence of HCV antibodies in a uraemic population undergoing maintenance dialysis therapy and in the staff members of the dialysis unit.	Besso	1992	No	Yes	No	Yes	Yes	No	Yes	Yes	No	Yes	6	Moderate risk of bias
96	Prevalence of Hepatitis B and C among HemodialysisPatients of Tripura, India.	Bhaumik	2012	No	Yes	No	Yes	Yes	No	Yes	Yes	No	Yes	6	Moderate risk of bias
97	The seroprevalence of anti-HCV in high-risk dialysis patients.	Bin Selm	2011	No	Yes	No	Yes	Yes	No	Yes	Yes	Unclear	Yes	6	Moderate risk of bias
98	Seroprevalence of Hepatitis B Virus and Hepatitis C Virus in Patients Undergoing Maintenance Hemodialysis.	Bin Shabbir	2022	No	Yes	No	Yes	Yes	No	Yes	Yes	Unclear	Yes	6	Moderate risk of bias
99	HCV viremia in hemodialysis patients: detection by a DNA enzyme immunoassay for amplified HCV sequences.	Boero	1995	No	Yes	No	Yes	Yes	No	Yes	Yes	Unclear	Yes	6	Moderate risk of bias
100	Does infection by the hepatitis C virus decrease the response of immunization against the hepatitis B virus in individuals undergoing dialysis?	Bonfm Ferreira	2017	No	Yes	No	Yes	Yes	No	Yes	Yes	Yes	Yes	7	Low risk of bias
101	Occurrence of infectious diseases in dialysed patients.	Borzecki	2004	No	Yes	No	Yes	Yes	No	Yes	Yes	Unclear	Yes	6	Moderate risk of bias
102	Screening for hepatitis B, C and HIV infection among patients on haemodialysis (cross sectional analysis among patients from two dialysis units in the period January to July 2005).	Bosevska	2009	No	Yes	No	Yes	Yes	No	Yes	Yes	No	No	5	Moderate risk of bias
103	[Prevalence of hepatitis C, hepatitis B and HIV infection among haemodialysis patients in Ibn-Rochd university hospital, Casablanca].	Boulaajaj	2005	No	Yes	No	Yes	Yes	No	Yes	Yes	Yes	Yes	7	Low risk of bias
104	Presence and significance of TT virus in Danish patients on maintenance hemodialysis.	Boysen	2003	No	Yes	No	Yes	Yes	No	Yes	Yes	No	Yes	6	Moderate risk of bias
105	Hepatitis C: reality of a renal unit.	Bracchi	1992	No	Yes	No	Yes	Yes	No	Yes	Yes	Unclear	Yes	6	Moderate risk of bias

106	Prevalence of hepatitis C infection and its risk factors in hemodialysis patients in tehran: preliminary report from "the effect of dialysis unit isolation on the incidence of hepatitis C in dialysis patients" project.	Broumand	2002	No	Yes	No	Yes	Yes	No	Yes	Yes	Unclear	No	5	Moderate risk of bias
107	Antibodies against hepatitis C virus in hemodialysis patients in the central Italian region of Umbria: evaluation of some risk factors.	Brugnano	1992	No	Yes	No	Yes	Yes	No	Yes	Yes	No	Yes	6	Moderate risk of bias
108	Seroepidemiology of HBV and HCV infection in Jakarta, Indonesia.	Budihusodo	1999	No	Yes	No	Yes	Yes	No	Yes	Yes	No	Yes	6	Moderate risk of bias
109	High prevalence of hepatitis C virus (HCV) RNA in dialysis patients: failure of commercially available antibody tests to identify a significant number of patients with HCV infection. Copenhagen Dialysis HCV Study Group.	Bukh	1993	No	Yes	No	Yes	Yes	No	Yes	Yes	No	Yes	6	Moderate risk of bias
110	Hepatitis C and hepatitis B virus infection in different hemodialysis units in Belo Horizonte, Minas Gerais, Brazil.	Busek	2002	No	Yes	No	Yes	Yes	No	Yes	Yes	No	Yes	6	Moderate risk of bias
111	Treatment Outcomes for Patients Undergoing Hemodialysis with Chronic Hepatitis C on the Sofosbuvir and Daclatasvi	Butt	2019	No	Yes	No	Yes	Yes	No	Yes	Yes	No	Yes	6	Moderate risk of bias
112	Transmission of anti-HCV within the household of haemodialysis patients.	Calabrese	1991	No	Yes	No	Yes	Yes	No	Yes	Yes	Unclear	Yes	6	Moderate risk of bias
113	TT virus infection in haemodialysis patients.	Campo	2000	No	Yes	No	Yes	Yes	No	Yes	Yes	Unclear	Yes	6	Moderate risk of bias
114	Prevalence of antibodies against hepatitis C virus in a dialysis unit.	Cantù	1992	No	Yes	No	Yes	Yes	No	Yes	Yes	Unclear	Yes	6	Moderate risk of bias
115	HCV seroprevalence in dialysis patients, their relatives and medical staff.	Capşa	1991	No	Yes	No	Yes	Yes	Unclear	Yes	Yes	Unclear	No	5	Moderate risk of bias
116	Undiagnosed hepatitis C virus infection in hemodialysis patients: value of HCV RNA and liver enzyme levels.	Caramelo	1996	No	Yes	No	Yes	Yes	No	Yes	Yes	Unclear	Yes	6	Moderate risk of bias
117	Hepatitis C prevalence and risk factors in hemodialysis patients in Central Brazil: a survey by polymerase chain reaction and serological methods.	Carneiro	2001	No	Yes	No	Yes	Yes	No	Yes	Yes	Unclear	Yes	6	Moderate risk of bias
118	Molecular and epidemiological study on nosocomial transmission of HCV in hemodialysis patients in Brazil.	Carneiro	2007	No	Yes	No	Yes	Yes	No	Yes	Yes	No	Yes	6	Moderate risk of bias
119	Persistence of antibodies to hepatitis C virus in a chronic hemodialysis population.	Carrera	1994	No	Yes	No	Yes	Yes	No	Yes	Yes	Unclear	Yes	6	Moderate risk of bias
120	Prevalence of non-A non-B hepatitis and anti-HCV antibodies in a Portuguese dialysis population.	Carrera	1992	No	Yes	No	Yes	Yes	No	Yes	Yes	No	Yes	6	Moderate risk of bias
121	High Prevalence of Hepatitis C Virus Infection in Chronic Hemodialysis Patients.	Carvalho	1999	No	Yes	No	Yes	Yes	No	Yes	Yes	Unclear	Yes	6	Moderate risk of bias
122	The prevalence of antibodies to hepatitis C virus at two haemodialysis units in South Africa.	Cassidy	1995	No	Yes	No	Yes	Yes	No	Yes	Yes	Unclear	Yes	6	Moderate risk of bias
123	Comparison of three different tests for assessment of hepatitis C virus in dialysis patients.	Castelnovo	1995	No	Yes	No	Yes	Yes	No	Yes	Yes	No	Yes	6	Moderate risk of bias
124	Prevalence of HBV, HDV, HCV infection and alleged risk factors in the Pistoia (Italy) haemodialysis population.	Catalani	2008	No	Yes	No	Yes	Yes	No	Yes	Yes	Unclear	Yes	6	Moderate risk of bias
125	Environmental transmission of hepatitis B and hepatitis C viruses within the hemodialysis unit.	Cendoroglo Neto	1995	No	Yes	No	Yes	Yes	No	Yes	Yes	Yes	Yes	7	Low risk of bias
126	Prevalence of hepatitis B and C virus infections among haemodialysis patients in Pune (western India).	Chadha	1993	No	Yes	No	Yes	Yes	No	Yes	Yes	Yes	Yes	7	Low risk of bias
127	Hepatitis C infection among dialysis patients: a comparison between patients on maintenance haemodialysis and continuous ambulatory peritoneal dialysis.	Chan	1991	No	Yes	No	Yes	Yes	No	Yes	Yes	Unclear	Yes	6	Moderate risk of bias
128	Discrepancy between serological and virological analysis of viral hepatitis in hemodialysis patients.	Chang	2014	No	Yes	No	Yes	Yes	No	Yes	Yes	Yes	Yes	7	Low risk of bias
129	Hepatitis C among child transfusion and adult renal dialysis patients in Indonesia.	Chanpong	2002	No	Yes	No	Yes	Yes	No	Yes	Yes	No	Yes	6	Moderate risk of bias
130	Prevalence of transfusion-transmitted virus infection in patients on maintenance hemodialysis from New Delhi, India.	Chattopadhyay	2005	No	Yes	No	Yes	Yes	No	Yes	Yes	Yes	Yes	7	Low risk of bias
131	Antibody to hepatitis C virus in risk groups in Canada.	Chaudhary	1992	No	Yes	No	Yes	Yes	No	Yes	Yes	Yes	Yes	7	Low risk of bias
132	Hepatitis B and C Virus infections associated withrenal replacement therapy in patients with endstage renal disease in a tertiary care hospital inIndia – prevalence, risk factors and outcome.	Chawla	2005	No	Yes	No	Yes	Yes	No	Yes	Yes	Unclear	Yes	6	Moderate risk of bias
133	Occult hepatitis B and C in hemodialysis patients in a hyper-endemic area in southern Taiwan.	Chen	2002	No	Yes	No	Yes	Yes	No	Yes	Yes	Unclear	Yes	6	Moderate risk of bias
134	Superinfection with hepatitis C virus in hemodialysis patients with hepatitis B surface antigenemia: its prevalence and clinical significance in Taiwan.	Chen	1996	No	Yes	No	Yes	Yes	No	Yes	Yes	No	Yes	6	Moderate risk of bias
135	Full-length sequence analysis of a new subtype of hepatitis C virus genotype 1 starting its spread in a hemodialysis unit.	Chevaliez	2009	No	Yes	No	Yes	Yes	No	Yes	Yes	Unclear	Yes	6	Moderate risk of bias
136	Serological monitoring of HCV marker in hemodialysis patients from tertiary care hospitals of Karachi. M	Chishti	2015	No	Yes	No	Yes	Yes	No	Yes	Yes	No	Yes	6	Moderate risk of bias
137	Association of uraemic pruritus with inflammation and hepatitis infection in haemodialysis patients.	Chiu	2008	No	Yes	No	Yes	Yes	No	Yes	Yes	Unclear	Yes	6	Moderate risk of bias
138	Duration of dialysis increases risk of hepatitis C virus infections among hemodialysis patients in Anambra state, Nigeria.	Chizoba	2018	No	Yes	No	Yes	Yes	No	Yes	Yes	No	Yes	6	Moderate risk of bias
139	Hepatitis B and C status according to dialysis modality in a Korean Single Center.	Choi	2009	No	Yes	No	Yes	Yes	No	Yes	Yes	Yes	No	6	Moderate risk of bias
140	Hepatitis C virus infection and haemodialysis: experience of a district general hospital in Brunei Darussalam.	Chong	2008	No	Yes	No	Yes	Yes	No	Yes	Yes	Unclear	Yes	6	Moderate risk of bias

141	Hepatitis C Virus Infection in Haemodialysis Patients: "Wolf in Sheep's Clothing".	Chopra	2005	No	Yes	No	Yes	Yes	No	Yes	Yes	Unclear	Yes	6	Moderate risk of bias
142	Prevalence and genotype distribution of hepatitis C virus within hemodialysis units in Thailand: Role of HCV core antigen in the assessment of viremia.	Chuaypen	2022	No	Yes	No	Yes	Yes	No	Yes	Yes	No	Yes	6	Moderate risk of bias
143	Is hepatitis G virus a real risk for haemodialysis patients?	Cocco	1998	No	Yes	No	Yes	Yes	No	Yes	Yes	Unclear	Yes	6	Moderate risk of bias
144	Prevalence of hepatitis C infection in a hemodialysis unit.	Colombo	1992	No	Yes	No	Yes	Yes	No	Yes	Yes	Unclear	Yes	6	Moderate risk of bias
145	Lower prevalence of anti-hepatitis C antibody in dialysis and renal transplant patients in Ireland.	Conlon	1993	No	Yes	No	Yes	Yes	No	Yes	Yes	No	Yes	6	Moderate risk of bias
146	Successful use of two rapid HCV assays in a high prevalence Romanian population.	Constantine	1994	No	Yes	No	Yes	Yes	No	Yes	Yes	No	Yes	6	Moderate risk of bias
147	Prevalence of antibodies to hepatitis C in dialysis patients and transplant recipients with possible routes of transmission.	Conway	1992	No	Yes	No	Yes	Yes	No	Yes	Yes	Unclear	Yes	6	Moderate risk of bias
148	Hepatitis C virus infection in haemodialysis patients: a clinical and virological study.	Corcoran	1994	No	Yes	No	Yes	Yes	No	Yes	Yes	Unclear	Yes	6	Moderate risk of bias
149	Decline in hepatitis B and C prevalence among hemodialysis patients in Tocantins, Northern Brazil.	Cordeiro	2018	No	Yes	No	Yes	Yes	No	Yes	Yes	No	No	5	Moderate risk of bias
150	Hepatitis C virus (HCV) infection in haemodialysed patients: HCV-RNA and anti-HCV antibodies (third-generation assays).	Courouсé	1995	No	Yes	No	Yes	Yes	No	Yes	Yes	Unclear	Yes	6	Moderate risk of bias
151	Hepatitis virus infection in haemodialysis patients from Moldavia.	Covic	1999	No	Yes	No	Yes	Yes	No	Yes	Yes	Unclear	Yes	6	Moderate risk of bias
152	A survey of hepatitis C virus infection in haemodialysis patients over a 7-year follow-up.	Cristina	1997	No	Yes	No	Yes	Yes	No	Yes	Yes	Yes	Yes	7	Low risk of bias
153	Hepatitis C virus in dialysis units: a multicenter study.	Da Porto	1992	No	Yes	Yes	Yes	Yes	No	Yes	Yes	Unclear	Yes	7	Low risk of bias
154	Evidence of association between hepatitis C virus genotype 2b and nosocomial transmissions in hemodialysis centers from southern Brazil.	da Silva	2013	No	Yes	No	Yes	Yes	No	Yes	Yes	Yes	Yes	7	Low risk of bias
155	Prevalence of HCV-RNA-positive patients in a dialysis unit in Germany.	Da silva cardoso	1994	No	Yes	Yes	Yes	Yes	No	Yes	Yes	Unclear	Yes	7	Low risk of bias
156	Prevalence and risk factors of hepatitis C virus among hemodialysis patients.	Dadgaran	2005	No	Yes	No	Yes	Yes	No	Yes	Yes	Unclear	Yes	6	Moderate risk of bias
157	[Investigation of hepatitis B and hepatitis C virus infections by serological and molecular methods in hemodialysis patients].	Dağlar	2014	No	Yes	Yes	Yes	Yes	Yes	Yes	Yes	Unclear	Yes	8	Low risk of bias
158	SEN virus infection among patients on maintenance hemodialysis in southern Taiwan.	Dai	2005	No	Yes	Yes	Yes	Yes	No	Yes	Yes	Unclear	Yes	7	Low risk of bias
159	Epidemiology and clinical significance of chronic hepatitis-related viruses infection in hemodialysis patients from Taiwan.	Dai	2002	No	Yes	Yes	Yes	Yes	No	Yes	Yes	Unclear	Yes	7	Low risk of bias
160	Absence of HCV viraemia in anti-HCV-negative haemodialysis patients.	Dalekos	1998	No	Yes	Yes	Yes	Yes	No	Yes	Yes	Unclear	Yes	7	Low risk of bias
161	Prevalence of hepatitis B and C virus infections and immunity among hemodialysis patients in the Mazandaran province, Northern Iran.	Darrudi	2022	No	Yes	No	Yes	Yes	No	Yes	Yes	Yes	Yes	7	Low risk of bias
162	Utility of routine real time quantitative PCR monitoring of HCV infection in haemodialysis patients.	Datta	2015	No	Yes	No	Yes	Yes	No	Yes	Yes	Yes	Yes	7	Low risk of bias
163	[Natural history of HCV infection and risk of death in a cohort of patients on long-term hemodialysis].	Dattolo	2006	No	Yes	Yes	Yes	Yes	No	Yes	Yes	Unclear	Yes	7	Low risk of bias
164	Blood-borne viruses in the haemodialysis-dependent population attending Top End Northern Territory facilities 2000-2009.	Davies	2012	No	Yes	Yes	Yes	Yes	No	Yes	Yes	Yes	Yes	8	Low risk of bias
165	Prevalence of hepatitis C virus antibodies among different populations of relative and attributable risk.	Daw	2002	No	Yes	Yes	Yes	Yes	No	Yes	Yes	Yes	Yes	8	Low risk of bias
166	Nosocomial transmission of hepatitis C virus in haemodialysis patients.	de Lamballerie	1996	No	Yes	Yes	Yes	Yes	No	Yes	Yes	No	Yes	7	Low risk of bias
167	[Prevalence of anti hepatitis C antibodies in patients with chronic renal failure receiving conservative therapy].	de los Rios	2006	No	Yes	Yes	Yes	Yes	No	Yes	Yes	Yes	Yes	8	Low risk of bias
168	Prevalence of hepatitis C and G virus infection in chronic hemodialysis patients.	de medina	1998	No	Yes	Yes	Yes	Yes	No	Yes	Yes	Unclear	Yes	7	Low risk of bias
169	Quantitative detection of hepatitis C virus RNA in patients undergoing hemodialysis.	de Medina	1997	No	Yes	Yes	Yes	Yes	No	Yes	Yes	No	Yes	7	Low risk of bias
170	Improved detection of antibodies to hepatitis C virus in dialysis patients using a second-generation enzyme immunoassay.	de Medina	1992	No	Yes	Yes	Yes	Yes	No	Yes	Yes	Unclear	Yes	7	Low risk of bias
171	Hepatitis C, HCV genotypes and hepatic siderosis in patients with chronic renal failure on haemodialysis in Brazil.	de Paula Farah	2007	No	Yes	Yes	Yes	Yes	No	Yes	Yes	Yes	Yes	8	Low risk of bias
172	Prevalence of viral hepatitis (Hep-B and Hep-C) infection in advanced renal failure patients: a tertiary care centre study.	Deep	2022	No	Yes	No	Yes	Yes	No	Yes	Yes	Yes	Yes	7	Low risk of bias
173	Outbreak of hepatitis C virus infection in a hemodialysis unit: potential transmission by the hemodialysis machine?	Delarocque-Astagneau	2002	No	Yes	Yes	Yes	Yes	No	Yes	Yes	Yes	Yes	8	Low risk of bias
174	Prevalence and incidence of hepatitis C virus (HCV) in hemodialysis patients: study of risk factors.	Dentico	1992	No	Yes	Yes	Yes	Yes	No	Yes	Yes	Yes	Yes	8	Low risk of bias
175	Hepatitis C virus-RNA, immunoglobulin M anti-HCV and risk factors in haemodialysis patients.	Dentico	1999	No	Yes	Yes	Yes	Yes	No	Yes	Yes	Unclear	Yes	7	Low risk of bias
176	Hepatitis C virus serotypes in haemodialysis patients in South-East Italy.	Dentico	2000	No	Yes	Yes	Yes	Yes	No	Yes	Yes	No	Yes	7	Low risk of bias
177	Hepatitis C virus in hemodialysis patients.	Dentico	1992	No	Yes	Yes	Yes	Yes	No	Yes	Yes	Unclear	Yes	7	Low risk of bias
178	Detection of antibodies to HCV in haemodialysis patients using two second generation ELISA tests.	Dentico	1993	No	Yes	Yes	Yes	Yes	No	Yes	Yes	Unclear	Yes	7	Low risk of bias
179	HCV third generation test in hemodialysis patients.	Dentico	1995	No	Yes	Yes	Yes	Yes	No	Yes	Yes	Unclear	Yes	7	Low risk of bias
180	Reduced antibody reactivity to hepatitis C virus antigens in hemodialysis patients coinfected with hepatitis B virus.	Devesa	1997	No	Yes	Yes	Yes	Yes	No	Yes	Yes	Yes	Yes	8	Low risk of bias
181	Risk factors of hepatitis C virus infection in patients on hemodialysis: a multivariate analysis based on a dialysis register in Central Italy.	Di lallo	1999	No	Yes	Yes	Yes	Yes	No	Yes	Yes	Yes	Yes	8	Low risk of bias

182	Hepatitis C virus antibodies in dialysis pediatric patients.	Di loreto	1992	No	Yes	Yes	Yes	Yes	No	Yes	Yes	Unclear	Yes	7	Low risk of bias
183	Confirmation of high prevalence of hepatitis C antibodies in hemodialysis patients by second generation immunoblot assay.	Di maggio	1992	No	Yes	Yes	Yes	Yes	No	Yes	Yes	Unclear	Yes	7	Low risk of bias
184	Epidemiology of hepatitis C virus among long-term dialysis patients: a 9-year study in an Italian region.	Di Napoli	2006	No	Yes	Yes	Yes	Yes	No	Yes	Yes	Yes	Yes	8	Low risk of bias
185	Further evidence for nosocomial spread of hepatitis C virus infection in hemodialysis units.	Dimković	1996	No	Yes	Yes	Yes	Yes	No	Yes	Yes	Yes	Yes	8	Low risk of bias
186	[Prevalence of hepatitis B and C viruses in a chronic hemodialysis center in Dakar].	Diouf	2000	No	Yes	Yes	Yes	Yes	No	Yes	Yes	Yes	Yes	8	Low risk of bias
187	Prevention of nosocomial transmission of hepatitis C infection in a hemodialysis unit. A prospective study	Djordjevic	2000	No	Yes	No	Yes	Yes	No	Unclear	Yes	Unclear	Yes	5	Moderate risk of bias
188	Hepatitis C virus infection in patients on peritoneal dialysis, hemodialysis and in dialysis staff members in south Serbia.	Djordjević	1996	No	Yes	Yes	Yes	Yes	No	Yes	Yes	No	Yes	7	Low risk of bias
189	[Prevalence and risk factors of hepatitis C virus infection in patients on hemodialysis: results of a Moroccan study].	Doblali	2014	No	Yes	Yes	Yes	Yes	No	Yes	Yes	Yes	Yes	8	Low risk of bias
190	[Seroprevalence of hepatitis C at the Military Hospital Mohammed V of Rabat].	Doblali	2014	No	Yes	No	Yes	Yes	No	Yes	Yes	Yes	Yes	7	Low risk of bias
191	Quantitation of hepatitis C viral RNA in sera of hemodialysis patients: gender-related differences in viral load.	DuBois	1994	No	Yes	No	Yes	Yes	No	Yes	Yes	Unclear	Yes	6	Moderate risk of bias
192	Hepatitis C virus in Vietnam: high prevalence of infection in dialysis and multi-transfused patients involving diverse and novel virus variants.	Dunford	2012	No	Yes	No	Yes	Yes	No	Yes	Yes	Yes	Yes	7	Low risk of bias
193	Hepatitis B and C virus infections among patients with end stage renal disease in a low-resourced hemodialysis center in Vietnam: a cross-sectional study.	Duong	2015	No	Yes	No	Yes	Yes	No	Yes	Yes	No	Yes	6	Moderate risk of bias
194	Screening haemodialysis patients for hepatitis C in Vietnam: The inconsistency between common hepatitis C virus serological and virological tests.	Duong	2019	No	Yes	No	Yes	Yes	No	Yes	Yes	Yes	Yes	7	Low risk of bias
195	Hepatitis C virus infection among chronic dialysis patients in the southeast of France. Provence-Alpes-Côte d'Azur Nephrologists Group.	Dussol	1995	No	Yes	No	Yes	Yes	No	Yes	Yes	Unclear	Yes	6	Moderate risk of bias
196	Detection of hepatitis C infection by polymerase chain reaction among hemodialysis patients.	Dussol	1993	No	Yes	No	Yes	Yes	No	Yes	Yes	Unclear	Yes	6	Moderate risk of bias
197	Hepatitis C virus infection among dialysis patients in United arab emirates.	El Shahat	1995	No	Yes	No	Yes	Yes	No	Yes	Yes	Yes	Yes	7	Low risk of bias
198	Hepatitis B and C Infections in Patients With Prolonged Hemodialysis Secondary to Chronic Renal Failure.	Elahi	2020	No	Yes	No	Yes	Yes	No	Yes	Yes	Yes	Yes	7	Low risk of bias
199	Hepatitis C virus infection in hemodialysis patients in Sudan: two centers' report.	El-Amin	2007	No	Yes	No	Yes	Yes	No	Yes	Yes	No	Yes	6	Moderate risk of bias
200	Diagnostic Performance of an Immunoassay for Simultaneous Detection of Hcv Core Antigen and Antibodies among Haemodialysis Patients.	El-Emshaty	2011	No	Yes	No	Yes	Yes	No	Yes	Yes	Unclear	Yes	6	Moderate risk of bias
201	Antibodies against hepatitis C virus (anti-HCV) in haemodialysis patients: association with hepatitis B serological markers.	Elisaf	1991	No	Yes	No	Yes	Yes	Unclear	Yes	Yes	Unclear	Yes	6	Moderate risk of bias
202	Prevalence and risk factors of hepatitis B and C viruses among haemodialysis patients in Gaza strip, Palestine.	El-kader	2010	No	Yes	No	Yes	Yes	Unclear	Yes	Yes	No	No	5	Moderate risk of bias
203	Hepatitis C virus infection in patients on maintenance dialysis in kuwait: epidemiological profile and efficacy of prophylaxis.	El-Reshaid	1995	No	Yes	No	Yes	Yes	Unclear	Yes	Yes	No	Yes	6	Moderate risk of bias
204	PREVALENCE OF ANTI-HEPATITIS-C VIRUS-ANTIBODIES AND HEPATITIS-C VIRUS VIREMIA IN CHRONIC-HEMODIALYSIS PATIENTS IN LIBYA.	Elzouki	1995	No	Yes	No	Yes	Yes	No	Yes	Yes	Unclear	Yes	6	Moderate risk of bias
205	HCV in hemodialysis patients in Benghazi, Libya.	El-Zouki	1993	No	Yes	No	Yes	Yes	No	Yes	Yes	Unclear	Yes	6	Moderate risk of bias
206	Marked reduction in the prevalence of hepatitis C virus infection in hemodialysis patients: causes and consequences.	Espinosa	2004	No	Yes	No	Yes	Yes	No	Yes	Yes	Yes	Yes	7	Low risk of bias
207	Risk of death and liver cirrhosis in anti-HCV positive long-term haemodialysis patients.	Espinosa	2001	No	Yes	No	Yes	Yes	No	Yes	Yes	Yes	Yes	7	Low risk of bias
208	Genotyping hepatitis C virus from hemodialysis patients in Central Brazil by line probe assay and sequence analysis.	Espírito-Santo	2007	No	Yes	No	Yes	Yes	No	Yes	Yes	No	Yes	6	Moderate risk of bias
209	De novo HCV infection among dialysis patients: a prospective study by HCV core antigen ELISA assay.	Fabrizi	2005	No	Yes	No	Yes	Yes	No	Yes	Yes	Yes	Yes	7	Low risk of bias
210	Hepatitis C virus infection and hepatic function in chronic hemodialysis patients.	Fabrizi	1992	No	Yes	No	Yes	Yes	No	Yes	Yes	Unclear	Yes	6	Moderate risk of bias
211	Influence of hepatitis C virus (HCV) viraemia upon serum aminotransferase activity in chronic dialysis patients.	Fabrizi	1997	No	Yes	No	Yes	Yes	No	Yes	Yes	Unclear	Yes	6	Moderate risk of bias
212	Hepatitis E virus infection in haemodialysis patients: a seroepidemiological survey.	Fabrizi	1997	No	Yes	No	Yes	Yes	No	Yes	Yes	No	No	5	Moderate risk of bias
213	Incidence of seroconversion for hepatitis C virus in chronic haemodialysis patients: a prospective study.	Fabrizi	1994	No	Yes	No	Yes	Yes	No	Yes	Yes	Yes	Yes	7	Low risk of bias
214	Virological characteristics of hepatitis C virus infection in chronic hemodialysis patients: a cross-sectional study.	Fabrizi	1995	No	Yes	No	Yes	Yes	No	Yes	Yes	Unclear	Yes	6	Moderate risk of bias
215	Molecular epidemiology of hepatitis C virus infection in dialysis patients.	Fabrizi	1997	No	Yes	No	Yes	Yes	No	Yes	Yes	No	Yes	6	Moderate risk of bias
216	Serologic survey for control of hepatitis C in haemodialysis patients: third-generation assays and analysis of costs.	Fabrizi	1997	No	Yes	No	Yes	Yes	No	Yes	Yes	No	Yes	6	Moderate risk of bias
217	Acquisition of hepatitis C virus in hemodialysis patients: a prospective study by branched DNA signal amplification assay.	Fabrizi	1998	No	Yes	No	Yes	Yes	No	Yes	Yes	Yes	Yes	7	Low risk of bias
218	Antibodies to hepatitis C virus (HCV) and transaminase concentration in chronic haemodialysis patients: a study with second-generation assays.	Fabrizi	1993	No	Yes	No	Yes	Yes	No	Yes	Yes	Unclear	Yes	6	Moderate risk of bias
219	Conflicting results of hepatitis C virus serological tests in haemodialysis patients.	Fabrizi	1993	No	Yes	No	Yes	Yes	No	Yes	Yes	Unclear	Yes	6	Moderate risk of bias

220	Low risk of sexual transmission of hepatitis C virus to spouses of infected hemodialysis patients.	Fadil-Romao	2006	No	Yes	No	Yes	Yes	No	Yes	Yes	Unclear	Yes	6	Moderate risk of bias
221	Prevalence of antibodies to hepatitis C virus in hemodialysis patients in Riyadh.	Fakunle	1991	No	Yes	No	Yes	Yes	No	Yes	Yes	Unclear	Yes	6	Moderate risk of bias
222	Hepatitis C virus RNA (HCV RNA) and viral types in dialysis patients in Dakar, Senegal.	Falasca	1999	No	Yes	No	Yes	Yes	No	Yes	Yes	Unclear	Yes	6	Moderate risk of bias
223	Hepatitis C and haemodialysis.	Faoagali	1990	No	Yes	No	Yes	Yes	No	Yes	Yes	Unclear	Yes	6	Moderate risk of bias
224	Hepatitis C, HCV genotypes and hepatic chronic renal failure on haemodialysis in plantation siderosis in patients with Brazil.	Farah	2007	No	Yes	No	Yes	Yes	No	Yes	Yes	Yes	Yes	7	Low risk of bias
225	Hepatic and extra-hepatic sequelae, and prevalence of viral hepatitis C infection estimated from routine data in at-risk groups.	Faustini	2010	No	Yes	No	Yes	Yes	No	Yes	Yes	Yes	Yes	7	Low risk of bias
226	Antibodies to hepatitis C virus in patients with chronic renal disease in Sri Lanka.	Fernando	2002	No	Yes	No	Yes	Yes	No	Yes	Yes	Yes	Yes	7	Low risk of bias
227	Hepatitis G virus infection in a haemodialysis unit: prevalence and clinical implications.	Forns	1997	No	Yes	No	Yes	Yes	No	Yes	Yes	Unclear	Yes	6	Moderate risk of bias
228	Incidence and risk factors of hepatitis C virus infection in a haemodialysis unit.	Forns	1997	No	Yes	No	Yes	Yes	No	Yes	Yes	Yes	Yes	7	Low risk of bias
229	High prevalence of TT virus (TTV) infection in patients on maintenance hemodialysis: frequent mixed infections with different genotypes and lack of evidence of associated liver disease.	Forns	1999	No	Yes	No	Yes	Yes	No	Yes	Yes	Yes	Yes	7	Low risk of bias
230	Hepatitis C in the health care setting. II. Seroprevalence among hemodialysis staff and patients in suburban New York City.	Forseter	1993	No	Yes	No	Yes	Yes	No	Yes	Yes	Unclear	Yes	6	Moderate risk of bias
231	Epidemiological and virological study of hepatitis c virus infection in hemodialysis	Foullous	2015	No	Yes	No	Yes	Yes	No	Yes	Yes	Unclear	Yes	6	Moderate risk of bias
232	Prevalence, genotypes and risk factors associated with hepatitis C virus infection in hemodialysis patients in Campo Grande, MS, Brazil.	Freitas	2008	No	Yes	No	Yes	Yes	Yes	Yes	Yes	Unclear	Yes	7	Low risk of bias
233	The prevalence of anti-HCV antibodies in hemodialysis patients.	Fujiyama	1991	No	Yes	No	Yes	Yes	No	Yes	Yes	Unclear	Yes	6	Moderate risk of bias
234	Changes in prevalence of anti-HCV antibodies associated with preventive measures among hemodialysis patients and dialysis staff.	Fujiyama	1995	No	Yes	No	Yes	Yes	No	Yes	Yes	Unclear	Yes	6	Moderate risk of bias
235	Prevalence of hepatitis C virus antibodies in hemodialysis patients and dialysis staff.	Fujiyama	1992	No	Yes	No	Yes	Yes	No	Yes	Yes	Unclear	Yes	6	Moderate risk of bias
236	Lower hepatitis G virus infection prevalence compared to hepatitis B and C virus infection prevalences.	Furusyo	2000	No	Yes	No	Yes	Yes	No	Yes	Yes	Yes	Yes	7	Low risk of bias
237	Acute hepatitis C among Japanese hemodialysis patients: a prospective 9-year study.	Furusyo	2001	No	Yes	No	Yes	Yes	No	Yes	Yes	Yes	Yes	7	Low risk of bias
238	Effect of isolation measures on the incidence and prevalence of hepatitis C virus infection in hernodialysis.	Gallego	2006	No	Yes	No	Yes	Yes	No	Yes	Yes	Yes	Yes	7	Low risk of bias
239	Hepatitis C in hemodialysis: the contribution of injection drug use.	Galperim	2010	No	Yes	No	Yes	Yes	No	Yes	Yes	Yes	Yes	7	Low risk of bias
240	[Antibodies against hepatitis C virus in patients with liver diseases and in risk subjects. Preliminary report].	Garassini	1990	No	Yes	No	Yes	Yes	No	Yes	Yes	Unclear	Yes	6	Moderate risk of bias
241	Relevance of investigating the presence of hepatitis C virus RNA in HCV antibody-negative hemodialysis patients.	García	2000	No	Yes	No	Yes	Yes	No	Yes	Yes	Yes	Yes	7	Low risk of bias
242	Comparison of the enzyme-linked immunosorbant assay III, recombinant immunoblot third generation assay, and polymerase chain reaction method in the detection of hepatitis C virus infection in haemodialysis patients.	Garinis	1999	No	Yes	No	Yes	Yes	No	Yes	Yes	Unclear	Yes	6	Moderate risk of bias
243	High prevalence of hepatitis G virus (HGV) infections in dialysis staff.	Gärtner	1999	No	Yes	No	Yes	Yes	No	Yes	Yes	Unclear	Yes	6	Moderate risk of bias
244	Epidemiology of hepatitis B and hepatitis C virus infections among hemodialysis patients in Khartoum, Sudan.	Gasim	2012	No	Yes	No	Yes	Yes	No	Yes	Yes	Unclear	Yes	6	Moderate risk of bias
245	HCV infection in hemodialyzed patients: incidence and correlation with dialytic age.	Giammaria	1992	No	Yes	No	Yes	Yes	No	Yes	Yes	Unclear	Yes	6	Moderate risk of bias
246	Non-A, non-B hepatitis and anti-HCV antibodies in dialysis patients.	Gilli	1990	No	Yes	No	Yes	Yes	Unclear	Yes	Yes	Unclear	Yes	6	Moderate risk of bias
247	Prevalence of antibodies to hepatitis C virus in patients on peritoneal dialysis--a multicenter study.	Gladziwa	1993	No	Yes	No	Yes	Yes	No	Yes	Yes	Unclear	Yes	6	Moderate risk of bias
248	Prevalence of antibodies to hepatitis C virus in hemodialysis patients and renal transplant recipients.	Gohar	1995	No	Yes	No	Yes	Yes	No	Yes	Yes	Unclear	Yes	6	Moderate risk of bias
249	[Anti-HCV seropositivity in dialysis patients].	Gomes	2006	No	Yes	No	Yes	Yes	Unclear	Yes	Yes	No	Yes	6	Moderate risk of bias
250	[Hepatitis C viral in patients with terminal chronic kidney failure. I. Prevalence].	González-Michaca	2000	No	Yes	No	Yes	Yes	No	Yes	Yes	Unclear	Yes	6	Moderate risk of bias
251	Prevalence and risk factors for hepatitis C virus infection in continuous ambulatory peritoneal dialysis patients.	Górriz	1996	No	Yes	Yes	Yes	Yes	Unclear	Yes	Yes	Unclear	Yes	7	Low risk of bias
252	Prevalence of hepatitis C virus antibodies in hemodialysis patients in the area of Milan.	Gubertini	1992	No	Yes	No	Yes	Yes	Unclear	Yes	Yes	Unclear	Yes	6	Moderate risk of bias
253	Prevalence of hepatitis C in patients on maintenance haemodialysis.	Gul	2003	No	Yes	No	Yes	Yes	No	Yes	Yes	Unclear	Yes	6	Moderate risk of bias
254	Prevalence of hepatitis C infection in hemodialysis patients: Report from 37 hemodialysis centers in Libya	Gusbi	2019	No	Yes	No	Yes	Yes	No	Yes	Yes	Unclear	Yes	6	Moderate risk of bias
255	Hepatitis C infection in hemodialysis units.	Ha	1990	No	Yes	No	Yes	Yes	No	Yes	Yes	Unclear	Yes	6	Moderate risk of bias

256	[Viral hepatitis C in chronic hemodialyzed patients in southern Tunisia. Prevalence and risk factors].	Hachicha	1995	No	Yes	No	Yes	Yes	No	Yes	Yes	Unclear	Yes	6	Moderate risk of bias
257	Hepatitis B virus subtypes and hepatitis C virus genotypes in patients with chronic liver disease or on maintenance hemodialysis in Indonesia	Hadiwandowo	1994	No	Yes	No	Yes	Yes	No	Yes	Yes	Unclear	Yes	6	Moderate risk of bias
258	Prevalence of hepatitis B and hepatitis C in blood donors and high risk groups in Hajjah, Yemen Republic.	Haidar	2002	No	Yes	No	Yes	Yes	No	Yes	Yes	No	Yes	6	Moderate risk of bias
259	National Epidemiological Study about Hepatitis C Virus Infection among Dialysis Patients.	Hajji	2021	Yes	Yes	No	Yes	Yes	No	Yes	Yes	No	Yes	7	Low risk of bias
260	Prospective virological follow-up of hepatitis C infection in a haemodialysis unit.	Halfon	1998	No	Yes	No	Yes	Yes	Unclear	Yes	Yes	Unclear	Yes	6	Moderate risk of bias
261	Hepatitis C virus transmission at an outpatient hemodialysis unit--New York, 2001-2008.	Hallack	2009	No	Yes	No	Yes	Yes	No	Yes	Yes	Yes	Yes	7	Low risk of bias
262	Hepatitis B, Hepatitis C, and Human Immune deficiency Virus Seroconversion Positivity Rates and Their Potential Risk Factors Among Patients on Maintenance Hemodialysis in Cameroon.	Halle	2016	No	Yes	No	Yes	Yes	Unclear	Yes	Yes	No	Yes	6	Moderate risk of bias
263	Occurrence of hepatitis B and C infection among hemodialyzed patients with chronic renal failure in Qazvin, Iran: A preliminary study	Hamissi	2011	No	Yes	No	Yes	Yes	No	Yes	Yes	No	Yes	6	Moderate risk of bias
264	Hepatitis B virus (HBV) and Hepatitis C virus (HCV) infections among hemodialysis patients at OMTH.	Hammad	2016	No	Yes	No	Yes	Yes	No	Yes	Yes	No	Yes	6	Moderate risk of bias
265	Hepatitis C virus infection in renal failure patients in the absence of anti-hepatitis C virus antibodies.	Hanuka	2002	No	Yes	No	Yes	Yes	No	Yes	Yes	Unclear	Yes	6	Moderate risk of bias
266	Antibody to hepatitis C virus increases with time on hemodialysis.	Hardy	1992	No	Yes	No	Yes	Yes	No	Yes	Yes	Unclear	Yes	6	Moderate risk of bias
267	Low prevalence of hepatitis C virus infection in hemodialysis units: effect of isolation?	Harmankaya	2002	No	Yes	No	Yes	Yes	No	Yes	Yes	Yes	Yes	7	Low risk of bias
268	Epidemiological Aspects of Hepatitis B and C and Human Immunodeficiency Viruses Among Hemodialysis Patients in Mazandaran Province, Iran.	Hasanjani Roushan	2016	No	Yes	No	Yes	Yes	No	Yes	Yes	Yes	Yes	7	Low risk of bias
269	Post-transfusion-transmitted hepatitis C virus infection: a study on thalassemia and hemodialysis patients in southeastern Iran.	Hassanshahi	2011	No	Yes	No	Yes	Yes	No	Yes	Yes	No	Yes	6	Moderate risk of bias
270	Prevalence of antibody to hepatitis C virus in hemodialysis patients.	Hayashi	1991	No	Yes	No	Yes	Yes	No	Yes	Yes	Unclear	Yes	6	Moderate risk of bias
271	Prevalence and role of hepatitis C viraemia in haemodialysis patients in Japan.	Hayashi	1994	No	Yes	No	Yes	Yes	No	Yes	Yes	Unclear	Yes	6	Moderate risk of bias
272	Seroepidemiology of hepatitis C virus infection in hemodialysis patients and the general population in Fukuoka and Okinawa, Japan.	Hayashi	1994	No	Yes	No	Yes	Yes	No	Yes	Yes	Unclear	Yes	6	Moderate risk of bias
273	Prevalence and risk factors of hepatitis C virus infection in haemodialysis patients: a multicentre study in 2796 patients.	Hinrichsen	2002	No	Yes	No	Yes	Yes	Unclear	Yes	Yes	No	Yes	6	Moderate risk of bias
274	Hepatitis C virus infection among dialysis patients in Tunisia: incidence and molecular evidence for nosocomial transmission.	Hmaied	2006	No	Yes	No	Yes	Yes	No	Yes	Yes	No	Yes	6	Moderate risk of bias
275	[HCV antibodies in hemodialyzed patients in Tunisia].	Hmida	1995	No	Yes	No	Yes	Yes	No	Yes	Yes	Unclear	Yes	6	Moderate risk of bias
276	Intrafamilial transmission of hepatitis C virus in hemodialysis patients.	Hou	1995	No	Yes	No	Yes	Yes	Unclear	Yes	Yes	Unclear	Yes	6	Moderate risk of bias
277	High prevalence of antibodies to hepatitis C virus in three haemodialysis centres in south-western Poland.	Hruby	1993	No	Yes	No	Yes	Yes	Unclear	Yes	Yes	Unclear	Yes	6	Moderate risk of bias
278	TT virus infection in patients on peritoneal dialysis in Taiwan.	Hsu	2007	No	Yes	No	Yes	Yes	Unclear	Yes	Yes	No	Yes	6	Moderate risk of bias
279	Hepatitis C markers in hemodialysis patients.	Huang	1993	No	Yes	No	Yes	Yes	No	Yes	Yes	Unclear	Yes	6	Moderate risk of bias
280	Hepatitis C virus--does it penetrate the haemodialysis membrane? PCR analysis of haemodialysis ultrafiltrate and whole blood.	Hubmann	1995	No	Yes	No	Yes	Yes	No	Yes	Yes	Unclear	Yes	6	Moderate risk of bias
281	High prevalence of and risk factors for hepatitis C in haemodialysis patients in Saudi Arabia: a need for new dialysis strategies.	Huraib	1995	No	Yes	No	Yes	Yes	No	Yes	Yes	Unclear	Yes	6	Moderate risk of bias
282	Hepatitis-C and it's seroconversion in end stage kidney disease patients on maintenance hemodialysis and factors affecting it.	Hussain	2019	No	Yes	No	Yes	Yes	No	Yes	Yes	Unclear	Yes	6	Moderate risk of bias
283	Methods used to reduce the prevalence of hepatitis C in a dialysis unit.	Hussein	2010	No	Yes	No	Yes	Yes	No	Yes	Yes	Yes	Yes	7	Low risk of bias
284	The impact of polymerase chain reaction assays for the detection of hepatitis C virus infection in a hemodialysis unit.	Hussein	2007	No	Yes	No	Yes	Yes	No	Yes	Yes	Yes	Yes	7	Low risk of bias
285	[The determination of the prevalence of antibodies to the hepatitis C virus (anti-HCV) and of the serum levels of C-reactive protein in chronic dialysis patients].	Iancu	1995	No	Yes	No	Yes	Yes	No	Yes	Yes	Unclear	Yes	6	Moderate risk of bias
286	[Hepatitis C virus: results of detection in several high risk groups in the X region of Chile].	Ibarra	1995	No	Yes	No	Yes	Yes	No	Yes	Yes	Unclear	Yes	6	Moderate risk of bias
287	Hepatitis C Virus Seroconversion Among Hemodialysis Patients and the Role of Hepatitis C Virus Positive Patient's Isolation in Benha, Egypt.	Ibrahim	2017	No	Yes	No	Yes	Yes	No	Yes	Yes	Unclear	Yes	6	Moderate risk of bias
288	Clinical significance of hepatitis G virus infection in patients on long-term haemodialysis.	Ideura	1997	No	Yes	No	Yes	Yes	No	Yes	Yes	Unclear	Yes	6	Moderate risk of bias
289	Superinfection of TT virus and hepatitis C virus among chronic haemodialysis patients.	Ikeuchi	1999	No	Yes	No	Yes	Yes	No	Yes	Yes	Unclear	Yes	6	Moderate risk of bias
290	[Hepatitis B virus markers and anti-HCV antibodies in hemodialyzed patients].	Illés	1992	No	Yes	No	Yes	Yes	No	Yes	Yes	Unclear	Yes	6	Moderate risk of bias
291	Comparison of anti-hepatitis C virus detection with ELISA assay and RIBA 4 in dialysis patients: our experience.	Innocenti	1992	No	Yes	No	Yes	Yes	No	Yes	Yes	Unclear	Yes	6	Moderate risk of bias

292	HCV incidence in a dialysis center: preliminary reports.	Ippolito	1992	No	Yes	No	Yes	Yes	No	Yes	Yes	Yes	Yes	7	Low risk of bias
293	Hepatitis C infection unrelated to blood transfusion in hemodialysis patients.	Irie	1994	No	Yes	No	Yes	Yes	No	Yes	Yes	No	Yes	6	Moderate risk of bias
294	Identification of hepatitis C virus seroconversion resulting from nosocomial transmission on a haemodialysis unit: implications for infection control and laboratory screening.	Irish	1999	No	Yes	No	Yes	Yes	No	Yes	Yes	Unclear	Yes	6	Moderate risk of bias
295	Torque teno virus infection in hemodialysis patients in North India.	Irshad	2010	No	Yes	No	Yes	Yes	No	Yes	Yes	Yes	Yes	7	Low risk of bias
296	HCV infection in Delhi, India.	Irshad	1998	No	Yes	No	Yes	Yes	No	Yes	Yes	Yes	Yes	7	Low risk of bias
297	Viral hepatitis in multiple blood transfused patients treated at a referral hospital of Delhi, India.	Irshad	2002	No	Yes	No	Yes	Yes	No	Unclear	Yes	Unclear	Yes	5	Moderate risk of bias
298	Epidemiology update for hepatitis C virus and hepatitis B virus in end-stage renal disease in France.	Isnard Bagnis	2017	Yes	Yes	Yes	Yes	Yes	No	Yes	Yes	Yes	Yes	9	Low risk of bias
299	Hepatitis C virus infection in hemodialysis patients: First-generation enzyme immunoassay.	Ivankovic	1994	No	Yes	No	Yes	Yes	No	Yes	Yes	Unclear	Yes	6	Moderate risk of bias
300	Patterns in the prevalence of hepatitis C virus infection at the start of hemodialysis in Japan.	Iwasa	2008	No	Yes	No	Yes	Yes	No	Yes	Yes	Yes	Yes	7	Low risk of bias
301	Occasional infection of hepatitis C virus occurring in haemodialysis units identified by serial monitoring of the virus infection.	Iwasaki	2000	No	Yes	No	Yes	Yes	No	Yes	Yes	Yes	Yes	7	Low risk of bias
302	Molecular evidence for nosocomial transmission of hepatitis C virus in a French hemodialysis unit.	Izopet	1999	No	Yes	No	Yes	Yes	No	Yes	Yes	Yes	Yes	7	Low risk of bias
303	Incidence of HCV infection in French hemodialysis units: a prospective study.	Izopet	2005	No	Yes	No	Yes	Yes	No	Yes	Yes	Yes	Yes	7	Low risk of bias
304	Prevalence and predisposing factors for hepatitis C virus in haemodialysis unit universiti kebangsaan malaysia medical centre.	Jaafar	2011	No	Yes	No	Yes	Yes	No	Yes	Yes	Yes	Yes	7	Low risk of bias
305	Hepatitis C in hemodialysis centers of golestan province, northeast of Iran (2005).	Jabbari	2008	No	Yes	No	Yes	Yes	No	Yes	Yes	Unclear	Yes	6	Moderate risk of bias
306	Incidence and risk factors for hepatitis C seroconversion in hemodialysis: A prospective study.	Jadoul	1993	No	Yes	No	Yes	Yes	No	Yes	Yes	Yes	Yes	7	Low risk of bias
307	Occult hepatitis C virus infection is more common than hepatitis B infection in maintenance hemodialysis patients.	Jain	2008	No	Yes	No	Yes	Yes	No	Yes	Yes	Yes	Yes	7	Low risk of bias
308	Prevalence of hepatitis viruses among chronic renal failure patients on hemodialysis in central India.	jaiswal	2002	No	Yes	No	Yes	Yes	No	Yes	Yes	Yes	Yes	7	Low risk of bias
309	A very high prevalence of hepatitis C virus infection among patients undergoing hemodialysis in Kosovo: a nationwide study.	Jakupi	2018	Yes	Yes	Yes	Yes	Yes	No	Yes	Yes	No	Yes	8	Low risk of bias
310	Prevalence of Hepatitis C Infection and its Genotypes in Suspected Hemodialysis Patients, Southwest of Iran.	Jamalidoust	2021	No	Yes	No	Yes	Yes	No	Yes	Yes	Yes	Yes	7	Low risk of bias
311	Hepatitis C and hepatitis B virus infection in hemodialysis patients and staff: a two year follow-up.	Jankovic	1994	No	Yes	No	Yes	Yes	No	Yes	Yes	Yes	Yes	7	Low risk of bias
312	Prevalence and associations of hepatitis C viremia in hemodialysis patients at a tertiary care hospital.	Jasuja	2009	No	Yes	No	Yes	Yes	No	Yes	Yes	Unclear	Yes	6	Moderate risk of bias
313	Prevalence and Risk Factors Associated with Hepatitis B and Hepatitis C Infections among Patients Undergoing Hemodialysis: A Single-Centre Study in Somalia.	jeele	2021	No	Yes	No	Yes	Yes	No	Yes	Yes	No	Yes	6	Moderate risk of bias
314	Hepatitis C infection in two urban hemodialysis units.	Jeffers	1990	No	Yes	No	Yes	Yes	No	Yes	Yes	No	Yes	6	Moderate risk of bias
315	Seropositivity to hepatitis C virus in Tunisian haemodialysis patients.	Jemni	1994	No	Yes	No	Yes	Yes	Unclear	Yes	Yes	Unclear	Yes	6	Moderate risk of bias
316	Hepatitis C Virus (HCV) Infection among Seronegative Patients undergoing Haemodialysis in a Remotely Located Tertiary Care Hospital of Northern India: Value of HCV-RNA and Genotypes.	Jindal	2015	No	Yes	No	Yes	Yes	No	Yes	Yes	Yes	Yes	7	Low risk of bias
317	HCV infection among healthy blood donors and risk groups in north India.	Jindal	2009	No	Yes	No	Yes	Yes	No	Yes	Yes	Yes	Yes	7	Low risk of bias
318	Hepatitis C infection in a pediatric dialysis population.	Jonas	1992	No	Yes	No	Yes	Yes	No	Yes	Yes	Unclear	Yes	6	Moderate risk of bias
319	Hepatitis C and hepatitis B seroprevalence and associated risk factors in hemodialysis patients in Guilan province, north of Iran: HCV and HBV seroprevalence in hemodialysis patients.	Joukar	2011	No	Yes	No	Yes	Yes	No	Yes	Yes	No	Yes	6	Moderate risk of bias
320	Occult hepatitis B infection in a hemodialysis population in Guilan Province, Northern Iran.	Joukar	2012	No	Yes	No	Yes	Yes	No	Yes	Yes	No	Yes	6	Moderate risk of bias
321	Prevalence of hepatitis B and C viruses infections among hemodialysis patients in Addis Ababa, Ethiopia.	Juhar	2018	No	Yes	No	Yes	Yes	No	Yes	Yes	No	Yes	6	Moderate risk of bias
322	[Anti-HCV antibodies among hemodialysed and kidney-transplanted patients].	Juszczyk	1994	No	Yes	No	Yes	Yes	No	Yes	Yes	Unclear	Yes	6	Moderate risk of bias
323	Prevalence and risk factors of hepatitis B and C viruses among hemodialysis patients in Isfahan, Iran.	KALANTARI	2014	No	Yes	Yes	Yes	Yes	No	Yes	Yes	Yes	Yes	8	Low risk of bias
324	Clinical characteristics and mortality in hepatitis C positive haemodialysis patients: a population based study.	Kalantar-Zadeh	2005	Yes	Yes	No	Yes	Yes	No	Yes	Yes	No	Yes	7	Low risk of bias
325	Circulation of an atypical hepatitis C virus (HCV) strain at a dialysis unit in northeast India.	kalita	2021	No	Yes	No	Yes	Yes	No	Yes	Yes	Yes	Yes	7	Low risk of bias
326	Laboratory Evaluation of Hepatitis C Virus Infection in Patients Undergoing Hemodialysis from North East India.	Kalita	2022	No	Yes	No	Yes	Yes	No	Yes	Yes	Unclear	Yes	6	Moderate risk of bias
327	Significance of hepatitis B, hepatitis C and GBV-C in ANCA-positive hemodialysis patients.	Kallinowski	1997	No	Yes	No	Yes	Yes	No	Yes	Yes	Unclear	Yes	6	Moderate risk of bias
328	Prevalence of antibodies to hepatitis C virus in hemodialysis patients.	Kallinowski	1991	No	Yes	No	Yes	Yes	No	Yes	Yes	Unclear	Yes	6	Moderate risk of bias
329	Seroprevalence occurrence of viral hepatitis and HIV among hemodialysis patients.	kamal	2018	No	Yes	No	Yes	Yes	No	Yes	Yes	Yes	Yes	7	Low risk of bias
330	Seroprevalence of human immunodeficiency virus, hepatitis B virus, and hepatitis C virus among	Kansay	2019	No	Yes	No	Yes	Yes	No	Yes	Yes	Yes	Yes	7	Low risk of bias

	hemodialysis patients in a Tertiary Care Teaching Hospital in a developing country														
331	Is dialysis environment more important than blood transfusion in transmission of hepatitis C virus during hemodialysis?	kapoor	1993	No	Yes	No	Yes	Yes	No	Yes	Yes	Unclear	Yes	6	Moderate risk of bias
332	Seroprevalence and risk factors of HCV in dialysis patients in a university hemodialysis, center of southeast Anatolia, Turkey.	kara	2001	No	Yes	No	Yes	Yes	No	Yes	Yes	Yes	Yes	7	Low risk of bias
333	Hepatitis C and G Virus Infection Prevalence Among Hemodialysis Patients and Associated Risk Factors in the Hormozgan Province of Southern Iran.	Kargar Kheirabad	2016	No	Yes	Yes	Yes	Yes	No	Yes	Yes	No	Yes	7	Low risk of bias
334	Hepatitis C Virus Among Hemodialysis Patients in Najran: Prevalence is More Among Multi-Center Visitors.	Kashem	2003	No	Yes	No	Yes	Yes	No	Yes	Yes	Unclear	Yes	6	Moderate risk of bias
335	Incidence and Risk Factors for Hepatitis C Virus and Hepatitis B Virus Seroconversion in End-Stage Renal Failure Patients on Maintenance Hemodialysis.	Kataruka	2020	No	Yes	No	Yes	Yes	No	Yes	Yes	No	Yes	6	Moderate risk of bias
336	Association of HCV core antigen seropositivity with long-term mortality in patients on regular hemodialysis.	Kato	2012	No	Yes	No	Yes	Yes	No	Yes	Yes	No	Yes	6	Moderate risk of bias
337	Quantitation and genotyping of hepatitis C virus RNA in sera of hemodialysis and AIDS patients.	Kessler	1996	No	Yes	No	Yes	Yes	No	Yes	Yes	Unclear	Yes	6	Moderate risk of bias
338	Risk factors for HCV infection in two haemodialysis units in The Netherlands.	Keur	1997	No	Yes	No	Yes	Yes	No	Yes	Yes	No	Yes	6	Moderate risk of bias
339	Evaluation of diagnostic value of ELISA method (EIA) & PCR in diagnosis of hepatitis C virus in hemodialysis patients.	Khadem-Ansari	2006	No	Yes	No	Yes	Yes	No	Yes	Yes	Unclear	Yes	6	Moderate risk of bias
340	High prevalence of hepatitis C virus infection and genotype distribution among general population, blood donors and risk groups.	Khaja	2006	No	Yes	No	Yes	Yes	No	Yes	Yes	Unclear	Yes	6	Moderate risk of bias
341	Prevalence of hepatitis B and C markers in patients on maintenance hemodialysis in Najran.	Khan	2001	No	Yes	No	Yes	Yes	No	Yes	Yes	No	Yes	6	Moderate risk of bias
342	Rising burden of Hepatitis C Virus in hemodialysis patients.	Khan	2011	No	Yes	Yes	Yes	Yes	No	Yes	Yes	No	Yes	7	Low risk of bias
343	Prevalence and risk factors for hepatitis C virus infection in hemodialysis patients in an Iraqi renal transplant center.	Khattab	2008	No	Yes	No	Yes	Yes	No	Yes	Yes	Yes	Yes	7	Low risk of bias
344	Presence and significance of transfusion-transmitted virus infection in Iranian patients on maintenance hemodialysis.	Kheradpezhouh	2013	No	Yes	No	Yes	Yes	No	Yes	Yes	No	Yes	6	Moderate risk of bias
345	Prevalence of HCV infections among hemodialysis patients in Al Gharbiyah Governorate, Egypt.	Khodir	2012	No	Yes	No	Yes	Yes	No	Yes	Yes	No	Yes	6	Moderate risk of bias
346	Risk factors for hepatitis C virus infection in patients on long-term hemodialysis.	Khokhar	2005	No	Yes	No	Yes	Yes	No	Yes	Yes	Yes	Yes	7	Low risk of bias
347	HCV-RNA Pcr Positivity In Hcv Antibody Negative Patients Undergoing Haemodialysis.	Kiani	2018	No	Yes	No	Yes	Yes	No	Yes	Yes	Unclear	Yes	6	Moderate risk of bias
348	Prevalence of hepatitis E virus infection in regular hemodialysis patients.	Kikuchi	2006	No	Yes	Yes	Yes	Yes	No	Yes	Yes	Unclear	Yes	7	Low risk of bias
349	Hepatitis C virus genotypes in chronic hemodialysis patients.	Kiliç	2000	No	Yes	No	Yes	Yes	No	Yes	Yes	Unclear	Yes	6	Moderate risk of bias
350	Hepatitis C in dialysis patients: relationship to blood transfusions, dialysis and liver disease.	Knudsen	1993	No	Yes	No	Yes	Yes	No	Yes	Yes	No	Yes	6	Moderate risk of bias
351	Eighteen-year follow-up cohort study on hepatitis B and C virus infections related long-term prognosis among hemodialysis patients in Hiroshima.	Ko	2020	No	Yes	No	Yes	Yes	No	Yes	Yes	Yes	Yes	7	Low risk of bias
352	Prospective follow-up study of hepatitis C virus infection in patients undergoing maintenance haemodialysis: comparison among haemodialysis units.	Kobayashi	1998	No	Yes	No	Yes	Yes	No	Yes	Yes	Yes	Yes	7	Low risk of bias
353	Detection of hepatitis B and C infection by polymerase chain reaction among hemodialysis patients.	Kocabaş	2002	No	Yes	No	Yes	Yes	No	Yes	Yes	Unclear	Yes	6	Moderate risk of bias
354	[Studies on hepatitis C virus infection in haemodialysis patients].	Koda	1992	No	Yes	No	Yes	Yes	No	Yes	Yes	Unclear	Yes	6	Moderate risk of bias
355	Hepatitis C antibodies in dialysis patients and patients with leukaemia.	Kolho	1993	No	Yes	No	Yes	Yes	No	Yes	Yes	No	Yes	6	Moderate risk of bias
356	Factors associated with the severity of pruritus in patients with terminal chronic kidney disease undergoing hemodialysis in Lima, Peru.	Kossuth-Cabrejos	2020	No	Yes	No	Yes	Yes	No	Unclear	Yes	No	Yes	5	Moderate risk of bias
357	Detection of hepatitis C virus RNA in hemodialysis patients.	Kuhns	1994	No	Yes	No	Yes	Yes	No	Yes	Yes	No	Yes	6	Moderate risk of bias
358	A cross-sectional study of dialysis practice-patterns in patients with chronic kidney disease on maintenance hemodialysis.	Kulkarni	2015	No	Yes	No	Yes	Yes	No	Yes	Yes	No	Yes	6	Moderate risk of bias
359	Hepatitis G virus infection in hemodialysis patients from urban Delhi.	Kumar	2005	No	Yes	No	Yes	Yes	No	Yes	Yes	Unclear	Yes	6	Moderate risk of bias
360	Hepatitis-C virus antibodies (anti HCV) in haemodialyzed vs non-dialyzed patients.	Kumar	1994	No	Yes	No	Yes	Yes	No	Yes	Yes	No	Yes	6	Moderate risk of bias
361	Prevalence and risk factors of Hepatitis C among maintenance hemodialysis patients at a tertiary-care hospital in Coimbatore, India.	Kumar	2011	No	Yes	No	Yes	Yes	No	Yes	Yes	Unclear	Yes	6	Moderate risk of bias
362	Differential effect of viral hepatitis infection on mortality among Korean maintenance dialysis patients: a prospective multicenter cohort study	Kwon	2015	No	Yes	No	Yes	Yes	No	Yes	Yes	Unclear	Yes	6	Moderate risk of bias
363	Evaluation of commercially available third-generation anti-hepatitis C virus enzyme-linked immunosorbent assay in patients on haemodialysis.	Lakshmi	2007	No	Yes	No	Yes	Yes	No	Yes	Yes	No	Yes	6	Moderate risk of bias
364	Nosocomal transmission of hepatitis C viruses (HCV) in a hemodialysis unit: Molecular evidence.	Lampe	2018	No	Yes	No	Yes	Yes	No	Yes	Yes	No	Yes	6	Moderate risk of bias
365	Molecular analysis and patterns of ALT and hepatitis C virus seroconversion in haemodialysis patients with acute hepatitis.	Lampe	2008	No	Yes	No	Yes	Yes	No	Yes	Yes	Unclear	Yes	6	Moderate risk of bias

366	Molecular epidemiology of a hepatitis C virus epidemic in a haemodialysis unit: outbreak investigation and infection outcome.	Lanini	2010	No	Yes	No	Yes	Yes	No	Yes	Yes	No	Yes	6	Moderate risk of bias
367	Significance of anti-E2 in the diagnosis of HCV infection in patients on maintenance hemodialysis: anti-E2 is frequently detected among anti-HCV antibody-negative patients.	Lee	1996	No	Yes	No	Yes	Yes	No	Yes	Yes	Unclear	Yes	6	Moderate risk of bias
368	Hepatitis C antibodies in patients on peritoneal dialysis: prevalence and risk factors.	Lee	1996	No	Yes	No	Yes	Yes	No	Yes	Yes	Unclear	Yes	6	Moderate risk of bias
369	Comparative study of hepatitis C virus antibody between hemodialysis and continuous ambulatory peritoneal dialysis patients.	Lee	1993	No	Yes	No	Yes	Yes	No	Yes	Yes	No	Yes	6	Moderate risk of bias
370	Seroepidemiology of hepatitis C virus infection in Taiwan.	Lee	1991	No	Yes	No	Yes	Yes	No	Yes	Yes	Unclear	Yes	6	Moderate risk of bias
371	Hepatitis C viral infection in a Chinese hemodialysis unit.	Li	2010	No	Yes	No	Yes	Yes	No	Yes	Yes	No	Yes	6	Moderate risk of bias
372	Hepatitis C virus core antigen in virological monitoring of dialysis patients.	Li Cavoli	2010	No	Yes	No	Yes	Yes	No	Yes	Yes	No	Yes	6	Moderate risk of bias
373	High incidence of hepatitis C virus infection in hemodialysis patients in Taiwan.	Lin	1993	No	Yes	No	Yes	Yes	No	Yes	Yes	Unclear	Yes	6	Moderate risk of bias
374	Prevalence of antibodies to hepatitis C virus in the hemodialysis unit.	Lin	1991	No	Yes	No	Yes	Yes	No	Yes	Yes	Unclear	Yes	6	Moderate risk of bias
375	Viral hepatitis C and B among dialysis patients at the Rabat University Hospital: prevalence and risk factors.	Lioussfi	2014	No	Yes	No	Yes	Yes	No	Yes	Yes	No	Yes	6	Moderate risk of bias
376	Profile and predictors of hepatitis and HIV infection in patients on hemodialysis of Quetta, Pakistan.	Lodhi	2019	No	Yes	No	Yes	Yes	No	Yes	Yes	No	Yes	6	Moderate risk of bias
377	Determination of the cut-off value of serum alanine aminotransferase in patients undergoing hemodialysis, to identify biochemical activity in patients with hepatitis C viremia.	Lopes	2006	No	Yes	Yes	Yes	Yes	No	Yes	Yes	No	Yes	7	Low risk of bias
378	Prevalence of hepatitis B, hepatitis C, GB virus C/hepatitis G and TT viruses in predialysis and hemodialysis patients.	López-Alcorocho	2001	No	Yes	Yes	Yes	Yes	No	Yes	Yes	Unclear	No	6	Moderate risk of bias
379	Prevalence of hepatitis C virus infection at three hemodialysis units in the western region of Puerto Rico.	López-Navedo	1999	No	Yes	Yes	Yes	Yes	No	Yes	Yes	No	Yes	7	Low risk of bias
380	[Hepatitis C virus infection among long-term hemodialysis patients].	Lu	1997	No	Yes	Yes	Yes	Yes	No	Yes	Yes	No	Yes	7	Low risk of bias
381	Hepatitis C virus infection in patients with chronic liver disease or chronic renal failure and blood donors in Thailand.	Luengrojanakul	1994	No	Yes	Yes	Yes	Yes	No	Yes	Yes	Unclear	Yes	7	Low risk of bias
382	Seroprevalence of human immunodeficiency virus, hepatitis B and C viruses among haemodialysis patients in two newly opened centres in Cameroon.	Luma	2017	No	Yes	Yes	Yes	Yes	No	Yes	Yes	No	Yes	7	Low risk of bias
383	Hepatitis B and C viral infections among hemodialysis patients attending 500 bedded Yangon specialty hospital, Myanmar	Lwin	2018	No	Yes	No	Yes	Yes	No	Yes	Yes	No	Yes	6	Moderate risk of bias
384	Prevalence of hepatitis C among haemodialysis patients in a tertiary care hospital in south India.	Madhavan	2020	No	Yes	No	Yes	Yes	No	Yes	Yes	No	No	5	Moderate risk of bias
385	Seronegative occult hepatitis C infection among hemodialysis patients: A prevalence study.	Mahmoudvand	2021	No	Yes	No	Yes	Yes	No	Yes	Yes	Yes	Yes	7	Low risk of bias
386	Prevalence and risk factors for hepatitis b and c among end-stage renal disease patients on hemodialysis in Gaborone, Botswana.	Mahupe	2021	No	Yes	No	Yes	Yes	No	Yes	Yes	No	Yes	6	Moderate risk of bias
387	Hepatitis C virus screening and clinical monitoring of biomarkers in patients undergoing hemodialysis.	Maia	2009	No	Yes	No	Yes	Yes	No	Yes	Yes	No	Yes	6	Moderate risk of bias
388	Hepatitis C prevalence studied by polymerase chain reaction and serological methods in haemodialysis patients in Mazandaran, Iran.	Makhlough	2008	No	Yes	Yes	Yes	Yes	No	Yes	Yes	No	Yes	7	Low risk of bias
389	Antibodies to hepatitis C virus (anti-HCV): prevalence in the same geographical area in dialysis patients, staff members, and blood donors.	Malaguti	1992	No	Yes	Yes	Yes	Yes	No	Yes	Yes	Unclear	No	6	Moderate risk of bias
390	Hepatitis B virus and hepatitis C virus co-infection in hemodialysis patients: A retrospective study from a tertiary care hospital of North India.	Malhotra	2016	No	Yes	Yes	Yes	Yes	No	Yes	Yes	Yes	Yes	8	Low risk of bias
391	Prevalence of hepatitis B and C infection in hemodialysis patients of Rasht (Center of Guilan Province, Northern part of Iran).	Mansour-Ghanael	2009	No	Yes	Yes	Yes	Yes	No	Yes	Yes	No	Yes	7	Low risk of bias
392	HEPATITIS VIRUS INFECTION IN END-STAGE KIDNEY DISEASE PATIENTS TREATED BY HAEMODIALYSIS IN ROMANIA AFTER 5 YEARS OF ACTIVE ANTIVIRAL THERAPY, REVISITED.	Marc	2022	No	Yes	No	Yes	Yes	No	Unclear	Yes	Unclear	Yes	5	Moderate risk of bias
393	Antibodies against hepatitis C virus in hemodialysis patients.	Mas	1996	No	Yes	No	Yes	Yes	No	Yes	Yes	Unclear	Yes	6	Moderate risk of bias
394	Sero Conversion of Viral Hepatitis among End Stage Renal Disease Patients on Hemodialysis in Kashmir: Results of a Prospective Study.	Masoodi	2019	No	Yes	No	Yes	Yes	No	Yes	Yes	Yes	Yes	7	Low risk of bias
395	Hepatitis C virus antibodies, viral RNA and genotypes in sera from patients on maintenance haemodialysis.	Masuko	1994	No	Yes	No	Yes	Yes	No	Yes	Yes	Unclear	Yes	6	Moderate risk of bias
396	Hepatitis C virus infection in renal dialysis patients in Glasgow.	McIntyre	1994	No	Yes	No	Yes	Yes	No	Yes	Yes	Unclear	Yes	6	Moderate risk of bias
397	[Prevalence and associated factors to hepatitis C in hemodialysis patients in Brazil].	Medeiros	2004	No	Yes	No	Yes	Yes	No	Yes	Yes	Unclear	Yes	6	Moderate risk of bias
398	Different kinetics of HBV and HCV during haemodialysis and absence of seronegative viral hepatitis in patients with end-stage renal disease.	Mederacke	2011	No	Yes	No	Yes	Yes	No	Yes	Yes	Unclear	Yes	6	Moderate risk of bias
399	Diagnostic utility of hepatitis C virus core antigen in hemodialysis patients.	Medhi	2008	No	Yes	No	Yes	Yes	No	Yes	Yes	Yes	Yes	7	Low risk of bias
400	Anti-hepatitis C virus positivity and clinical correlations in hemodialyzed patients.	Medici	1992	No	Yes	No	Yes	Yes	No	Yes	Yes	Unclear	Yes	6	Moderate risk of bias
401	Seroconversion to hepatitis C virus in dialysis patients: a retrospective and prospective study.	Medin	1993	No	Yes	No	Yes	Yes	No	Yes	Yes	Yes	Yes	7	Low risk of bias

402	[Hepatitis C serum prevalence in hemodialyzed patients].	Mello Lde	2007	No	Yes	No	Yes	Yes	Unclear	Yes	Yes	Unclear	Yes	6	Moderate risk of bias
403	[Risk factors for hepatitis C in hemodialysis and its impact on the waiting list for kidney transplantation].	Méndez Chacón	2005	No	Yes	No	Yes	Yes	Unclear	Yes	Yes	No	Yes	6	Moderate risk of bias
404	Prevalence of hepatitis C virus infection among hemodialysis patients at a tertiary-care hospital in Mexico City, Mexico.	Méndez-Sánchez	2004	No	Yes	No	Yes	Yes	No	Yes	Yes	Unclear	Yes	6	Moderate risk of bias
405	Prevalence and risk factors of hepatitis B and C among hemodialysis patients in Tunisia.	Mhalla	2018	No	Yes	No	Yes	Yes	No	Yes	Yes	Yes	Yes	7	Low risk of bias
406	Profile of hepatitis B virus, hepatitis C virus, hepatitis d virus and human immunodeficiency virus infections in hemodialysis patients of a tertiary care hospital in uttarakhand.	Mittal	2013	No	Yes	No	Yes	Yes	No	Yes	Yes	Yes	Yes	7	Low risk of bias
407	Hepatitis C in chronic renal failure patients.	Mitwalli	1992	No	Yes	No	Yes	Yes	No	Yes	Yes	Yes	Yes	7	Low risk of bias
408	Hepatitis G virus (HGV) infection in Saudi dialysis patients and healthy controls.	Mitwalli	2000	No	Yes	No	Yes	Yes	No	Yes	Yes	Yes	Yes	7	Low risk of bias
409	[Hepatitis C in hemodialysis patients].	Miyano	2001	No	Yes	No	Yes	Yes	Unclear	Yes	Yes	Unclear	Yes	6	Moderate risk of bias
410	[Infection of hepatitis C virus in patients with chronic renal failure undergoing hemodialysis therapy and staff members].	Miyasaka	1991	No	Yes	No	Yes	Yes	Unclear	Yes	Yes	Unclear	Yes	6	Moderate risk of bias
411	Genetic and serological evidence for multiple instances of unrecognized transmission of hepatitis C virus in hemodialysis units.	Mizuno	1998	No	Yes	No	Yes	Yes	No	Yes	Yes	Unclear	Yes	6	Moderate risk of bias
412	Molecular evaluation of hepatitis G virus and hepatitis C virus in patients with chronic renal failure in Iran.	Mohsenzadeh	2012	No	Yes	No	Yes	Yes	No	Yes	Yes	Unclear	Yes	6	Moderate risk of bias
413	Hepatitis C virus (HCV) Infection Rate among Seronegative Hemodialysis Patients Screened by Two Methods; HCV Core Antigen and Polymerase Chain Reaction.	Moini	2013	No	Yes	No	Yes	Yes	No	Yes	Yes	No	Yes	6	Moderate risk of bias
414	High prevalence of antibodies to hepatitis C virus in hemodialysis units using a second generation assay.	Mondelli	1992	No	Yes	No	Yes	Yes	No	Yes	Yes	Unclear	Yes	6	Moderate risk of bias
415	Abnormal alanine aminotransferase activity reflects exposure to hepatitis C virus in haemodialysis patients.	Mondelli	1991	No	Yes	No	Yes	Yes	No	Yes	Yes	Yes	Yes	7	Low risk of bias
416	Hepatitis C virus infection in hemodialysis patients in Maracaibo, Venezuela.	Monsalve-Castillo	2012	No	Yes	No	Yes	Yes	No	Yes	Yes	Yes	Yes	7	Low risk of bias
417	Epidemiology of hepatitis C virus infection in hemodialysis patients of Sicily.	Montalto	2008	No	Yes	No	Yes	Yes	No	Yes	Yes	Unclear	Yes	6	Moderate risk of bias
418	Impact of prophylactic measures on prevalence of anti HCV and viral genotypes in a dialysis unit [13].	Morales	1997	No	Yes	No	Yes	Yes	No	Yes	Yes	Yes	Yes	7	Low risk of bias
419	Prospective study of hepatitis C virus infection in hemodialysis patients by monthly analysis of HCV RNA and antibodies.	Moreira	2003	No	Yes	No	Yes	Yes	Unclear	Yes	Yes	Yes	Yes	7	Low risk of bias
420	Prevalence and characterization of hepatitis C virus in hemodialysis patients.	Morikawa	1999	No	Yes	No	Yes	Yes	No	Yes	Yes	Unclear	Yes	6	Moderate risk of bias
421	Epidemiology of hepatitis C in a population of hemodialysis patients.	Mosconi	1992	No	Yes	No	Yes	Yes	No	Yes	Yes	Unclear	Yes	6	Moderate risk of bias
422	[The prevalence of serum anti-hepatitis C virus antibodies in hemodialyzed patients].	Motta	1991	No	Yes	No	Yes	Yes	No	Yes	Yes	Unclear	Yes	6	Moderate risk of bias
423	Epidemiology of hepatitis C virus infection in ESRD patients in Khuzestan Province, Iran.	Mousavi	2012	No	Yes	No	Yes	Yes	No	Yes	Yes	Yes	Yes	7	Low risk of bias
424	Prevalence of HCV antibodies in haemodialysis population of Multan - Pakistan.	Muhammad	1997	No	Yes	No	Yes	Yes	Unclear	Yes	Yes	Unclear	Yes	6	Moderate risk of bias
425	Risk factors for dialysis-associated hepatitis C in Venezuela.	Muller	1992	No	Yes	No	Yes	Yes	No	Yes	Yes	Unclear	Yes	6	Moderate risk of bias
426	Profile of hepatitis B and hepatitis C virus infections in dialysis and renal transplant patients 1997-2001: CMCH Vellore	Murthy	2003	No	Yes	No	Yes	Yes	No	Yes	Yes	Yes	Yes	7	Low risk of bias
427	Incidence of antibodies to hepatitis C virus in patients undergoing chronic dialysis and CAPD.	Nakashima	1993	No	Yes	No	Yes	Yes	No	Yes	Yes	No	Yes	6	Moderate risk of bias
428	Hepatitis C and B virus infections in populations at low or high risk in Ho Chi Minh and Hanoi, Vietnam	Nakata	1994	No	Yes	No	Yes	Yes	No	Yes	Yes	No	Yes	6	Moderate risk of bias
429	Prognosis of anti-hepatitis C virus antibody-positive patients on regular hemodialysis therapy.	Nakayama	2000	No	Yes	No	Yes	Yes	No	Yes	Yes	Yes	Yes	7	Low risk of bias
430	Low prevalence of anti-hepatitis C virus antibodies in female hemodialysis patients without blood transfusion: a multicenter analysis.	Nakayama	1996	No	Yes	No	Yes	Yes	No	Yes	Yes	Yes	Yes	7	Low risk of bias
431	Prevalence of hepatitis b &c in patients of end stage renal disease (ESRD) on treatment of chronic/long-term hemodialysis.	Nasir	2019	No	Yes	No	Yes	Yes	No	Yes	Yes	Yes	Yes	7	Low risk of bias
432	Hepatitis C virus infection among patients on hemodialysis: a report from a single center in Iran.	Nemati	2009	No	Yes	No	Yes	Yes	Unclear	Yes	Yes	Unclear	Yes	6	Moderate risk of bias
433	ENVIRONMENTAL TRANSMISSION OF HEPATITIS-B AND HEPATITIS-C VIRUSES WITHIN THE HEMODIALYSIS UNIT.	Neto	1995	No	Yes	No	Yes	Yes	No	Yes	Yes	Yes	Yes	7	Low risk of bias
434	Prevalence of hepati[1]tis C virus infection according to the year of birth: identifcation of risk groups.	Neukam	2018	No	Yes	No	Yes	Yes	No	Yes	Yes	Yes	Yes	7	Low risk of bias
435	Outbreak of hemodialysis-associated non-A, non-B hepatitis and correlation with antibody to hepatitis C virus.	Niu	1992	No	Yes	No	Yes	Yes	Unclear	Yes	Yes	Yes	Yes	7	Low risk of bias
436	Multicenter study of hepatitis C virus infection in chronic hemodialysis patients and hemodialysis center staff members.	Niu	1993	No	Yes	Yes	Yes	Yes	No	Yes	Yes	Yes	Yes	8	Low risk of bias
437	Prevalence of hepatitis C virus antibody in patients on chronic hemodialysis.	Nomiyama	1998	No	Yes	No	Yes	Yes	Unclear	Yes	Yes	Unclear	Yes	6	Moderate risk of bias
438	Hepatitis C virus infection in hemodialysis patients in southern Sweden: epidemiological, clinical, and diagnostic aspects.	Nordenfelt	1993	No	Yes	No	Yes	Yes	No	Yes	Yes	Unclear	Yes	6	Moderate risk of bias

439	Seroprevalence of hepatitis C in patients with type 2 diabetes mellitus and non-diabetic on haemodialysis.	ocak	2006	No	Yes	No	Yes	Yes	No	Yes	Yes	Unclear	Yes	6	Moderate risk of bias
440	Hepatitis virus infection (HBV and HCV) in eleven Japanese hemodialysis units.	Oguchi	1992	No	Yes	No	Yes	Yes	Unclear	Yes	Yes	Unclear	Yes	6	Moderate risk of bias
441	Prevalence of anti-HCV in patients on long-term hemodialysis.	Oguchi	1990	No	Yes	No	Yes	Yes	Unclear	Yes	Yes	Unclear	Yes	6	Moderate risk of bias
442	Standardized prevalence ratios for chronic hepatitis C virus infection among adult Japanese hemodialysis patients.	Ohsawa	2010	No	Yes	No	Yes	Yes	No	Yes	Yes	Yes	Yes	7	Low risk of bias
443	Seropositivity for anti-HCV core antigen is independently associated with increased all[1]cause, cardiovascular, and liver disease-related mortality in haemodialysis patients.	Ohsawa	2010	No	Yes	No	Yes	Yes	No	Yes	Yes	Yes	Yes	7	Low risk of bias
444	Sero-prevalence of Hepatitis B and C infection among patients with end stage renal disease at haemodialysis initiation. African Journal of Medical and Health Sciences.	Okoye	2020	No	Yes	No	Yes	Yes	No	Yes	Yes	Yes	Yes	7	Low risk of bias
445	Epidemiological Survey of Patients With Hemodialysis Complicated by Hepatitis C in Japan.	Okubo	2019	No	Yes	No	Yes	Yes	No	Yes	Yes	Yes	Yes	7	Low risk of bias
446	Markers of hepatitis C infection among hemodialysis patients with acute and chronic infection: implications for infection control strategies in hemodialysis units.	Oliva	1995	No	Yes	No	Yes	Yes	Unclear	Yes	Yes	Unclear	Yes	6	Moderate risk of bias
447	Late seroconversion of C virus markers in hemodialysis patients.	Oliva	1993	No	Yes	No	Yes	Yes	Unclear	Yes	Yes	Yes	Yes	7	Low risk of bias
448	Seroprevalence of hepatitis C virus infection and evaluation of serum aminotransferase levels among haemodialysis patients in Izmir, Turkey.	Olut	2005	No	Yes	No	Yes	Yes	No	Yes	Yes	No	Yes	6	Moderate risk of bias
449	Liver enzymes and protein electrophoretic patterns in hemodialysis patients with antibodies against the hepatitis C virus.	Omar	1995	No	Yes	No	Yes	Yes	No	Yes	Yes	Unclear	Yes	6	Moderate risk of bias
450	A prospective study of the prevalence of hepatitis B and C virus co-infection among patients with chronic renal disease under hemodialysis.	Ono-nita	2004	No	Yes	No	Yes	Yes	No	Yes	Yes	Yes	Yes	7	Low risk of bias
451	Seroprevalence of hepatitis B and C in maintenance dialysis in a public hospital in a developing country.	Otedo	2003	No	Yes	No	Yes	Yes	No	Yes	Yes	No	Yes	6	Moderate risk of bias
452	Prevalence of antibodies to hepatitis C virus among hemodialysis patients in Damascus, Syria.	Othman	2001	No	Yes	No	Yes	Yes	No	Yes	Yes	No	Yes	6	Moderate risk of bias
453	Revelance of hepatitis C virus RNA detection, quantitation and genotypes in a hemodialysis unit.	Ouzan	1997	No	Yes	No	Yes	Yes	No	Yes	Yes	Unclear	Yes	6	Moderate risk of bias
454	[Anti HCV in hemodialyzed patients: reduction of the prevalence and association with epidemiological variables].	Padrone	1999	No	Yes	No	Yes	Yes	Unclear	Yes	Yes	Unclear	Yes	6	Moderate risk of bias
455	Prevalence of HCV, HBV and HIV infections in patients and staff of haemodialysis unit.	Palanisamy	2012	No	Yes	No	Yes	Yes	No	Yes	Yes	Unclear	Yes	6	Moderate risk of bias
456	Risk factors and clinical expression of HCV infection in hemodialysis patients.	Pauri	1992	No	Yes	No	Yes	Yes	No	Yes	Yes	Yes	Yes	7	Low risk of bias
457	Hepatitis C virus seropositivity and TNF superfamily receptors: sCD40, sFas--the new putative determinants of endothelial dysfunction in haemodialysis patients.	Pawlak	2010	No	Yes	No	Yes	Yes	No	Yes	Yes	Unclear	Yes	6	Moderate risk of bias
458	The prevalence of anti-HCV positivity in patients undergoing haemodialysis or with malignant disease.	Paydas	1994	No	Yes	No	Yes	Yes	No	Yes	Yes	Unclear	Yes	6	Moderate risk of bias
459	[Hepatitis C virus antibodies in persons on dialysis].	Pazdiora	1993	No	Yes	No	Yes	Yes	No	Yes	Yes	Unclear	Yes	6	Moderate risk of bias
460	[Viral hepatitis C--a problem in the treatment of children with renal insufficiency on hemodialysis].	Peco-Antić	1993	No	Yes	No	Yes	Yes	No	Yes	Yes	Yes	Yes	7	Low risk of bias
461	[Epidemiologic study of infection by hepatitis C virus in a hemodialysis unit].	Pena	2000	No	Yes	No	Yes	Yes	No	Yes	Yes	Unclear	Yes	6	Moderate risk of bias
462	Influence of hepatitis C virus infection on soluble cellular adhesion molecules in hemodialysis patients.	Peng	2005	No	Yes	No	Yes	Yes	No	Yes	Yes	Unclear	Yes	6	Moderate risk of bias
463	Seroprevalence of hepatitis B, hepatitis C and HIV infection among patients undergoing haemodialysis in Buenos Aires, Argentina	Pereson	2021	No	Yes	No	Yes	Yes	No	Yes	Yes	Yes	Yes	7	Low risk of bias
464	Prevalence of antihepatitis C antibodies in patients treated with continuous ambulatory peritoneal dialysis and hemodialysis.	Pérez-Fontán	1991	No	Yes	No	Yes	Yes	No	Yes	Yes	No	Yes	6	Moderate risk of bias
465	Prevalence of infected patients and understaffing have a role in hepatitis C virus transmission in dialysis.	Petrosillo	2001	No	Yes	No	Yes	Yes	No	Yes	Yes	Yes	Yes	7	Low risk of bias
466	Prevalence of human immunodeficiency virus, hepatitis B virus and hepatitis C virus among dialysis patients. The Italian Multicentric Study on Nosocomial and Occupational Risk of Blood-Borne Infections in Dialysis.	Petrosillo	1993	No	Yes	No	Yes	Yes	No	Yes	Yes	Unclear	Yes	6	Moderate risk of bias
467	The risks of occupational exposure and infection by human immunodeficiency virus, hepatitis B virus, and hepatitis C virus in the dialysis setting. Italian Multicenter Study on Nosocomial and Occupational Risk of Infections in Dialysis.	Petrosillo	1995	No	Yes	No	Yes	Yes	No	Yes	Yes	Yes	Yes	7	Low risk of bias
468	Anti-hepatitis C virus antibodies and hepatitis C virus viraemia in haemodialysis patients.	Picciotto	1993	No	Yes	No	Yes	Yes	No	Yes	Yes	Unclear	Yes	6	Moderate risk of bias
469	[Hepatitis C virus infection in patients on chronic hemodialysis].	Pljesa	1996	No	Yes	No	Yes	Yes	No	Yes	Yes	Unclear	Yes	6	Moderate risk of bias
470	Hepatitis C virus-related acute and chronic hepatitis in hemodialysis patients.	Pluvio	1992	No	Yes	No	Yes	Yes	No	Yes	Yes	Unclear	Yes	6	Moderate risk of bias
471	Serprevalence of hepatitis-C virus in blood donors and high risk individuals.	Poddar	2012	No	Yes	No	Yes	Yes	No	Yes	Yes	Yes	Yes	7	Low risk of bias
472	Prevalence of hepatitis B & C viruses among patients on hemodialysis in Lucknow, Uttar Pradesh.	Prakash	2014	No	Yes	No	Yes	Yes	No	Yes	Yes	Unclear	Yes	6	Moderate risk of bias
473	Comparison of third generation ELISA and conventional nested RT-PCR for detection of HCV among hemodialysis patients.	Prakash	2014	No	Yes	No	Yes	Yes	No	Yes	Yes	Unclear	Yes	6	Moderate risk of bias
474	Hepatitis E virus (HEV) infection in haemodialysis patients.	Psichogiou	1996	No	Yes	No	Yes	Yes	No	Yes	Yes	Unclear	Yes	6	Moderate risk of bias

475	High incidence of hepatitis C virus infection in hemodialysis patients in units with high prevalence.	Pujol	1996	No	Yes	No	Yes	Yes	No	Yes	Yes	Unclear	Yes	6	Moderate risk of bias
476	Prevalence of hepatitides in our hemodialyzed population.	Raccosta	1992	No	Yes	No	Yes	Yes	No	Yes	Yes	Unclear	Yes	6	Moderate risk of bias
477	Seroprevalence and risk factors for Hepatitis C virus among maintenance hemodialysis patients at a Tertiary Care Hospital in Puducherry, India.	Ragunathan	2022	No	Yes	No	Yes	Yes	No	Yes	Yes	Yes	Yes	7	Low risk of bias
478	Prevalence of Hepatitis B and Hepatitis C in Patients undergoing hemodialysis at a teaching hospital in Uttarakhand.	Raina	2022	No	Yes	No	Yes	Yes	No	Yes	Yes	No	Yes	6	Moderate risk of bias
479	Anti-HCV seropositivity among haemodialysis patients of Iranian origin.	Rais-Jalali	1999	No	Yes	No	Yes	Yes	No	Yes	Yes	Unclear	Yes	6	Moderate risk of bias
480	Serological pattern of anti-HBc alone infers occult hepatitis B virus infection in high-risk individuals in Iran.	Ramezani	2013	No	Yes	No	Yes	Yes	No	Yes	Yes	Yes	Yes	7	Low risk of bias
481	Improved detection of hepatitis C virus infection by transcription-mediated amplification technology in dialysis population.	Rao	2010	No	Yes	No	Yes	Yes	No	Yes	Yes	Unclear	Yes	6	Moderate risk of bias
482	Hepatitis C in several risk groups of Kosovo.	Rashiti-Bytyci	2022	No	Yes	No	Yes	Yes	Yes	Yes	Yes	No	Yes	7	Low risk of bias
483	Utility of HCV core antigen ELISA in the screening for hepatitis C virus infection in patients on hemodialysis.	Reddy	2006	No	Yes	No	Yes	Yes	No	Yes	Yes	Yes	Yes	7	Low risk of bias
484	Prevalence of HBV and HCV dual infection in patients on haemodialysis.	Reddy	2005	No	Yes	No	Yes	Yes	No	Yes	Yes	Yes	Yes	7	Low risk of bias
485	Hepatitis C infection and the risk of bacteremia in hemodialysis patients with tunneled vascular access catheters.	Reddy	2009	No	Yes	No	Yes	Yes	No	Yes	Yes	No	Yes	6	Moderate risk of bias
486	Cross-Sectional Study to Determine the Prevalence of Hepatitis B and C Virus Infection in High Risk Groups in the Northeast Region of Brazil.	Ribeiro	2017	No	Yes	No	Yes	Yes	No	Yes	Yes	No	Yes	6	Moderate risk of bias
487	HCV-RNA qualitative assay based on transcription mediated amplification improves the detection of hepatitis C virus infection in patients on hemodialysis: results from five hemodialysis units in central Greece.	Rigopoulou	2005	No	Yes	No	Yes	Yes	No	Yes	Yes	Unclear	Yes	6	Moderate risk of bias
488	Hepatitis B and C virus infection among hemodialysis patients in Yogyakarta, Indonesia: Prevalence and molecular evidence for nosocomial transmission.	Rinonce	2013	No	Yes	Yes	Yes	Yes	No	Yes	Yes	No	Yes	7	Low risk of bias
489	Detection of antibodies to hepatitis C virus in dialysis patients.	Rivanera	1993	No	Yes	No	Yes	Yes	No	Yes	Yes	Yes	Yes	7	Low risk of bias
490	Prevalence of TT virus infection in Italian-dialysis patients.	Rivanera	2009	No	Yes	No	Yes	Yes	No	Yes	Yes	Unclear	Yes	6	Moderate risk of bias
491	Prevalence of hepatitis C virus infection and genotypes in patient with chronic kidney disease undergoing hemodialysis.	Rodrigues de Freitas	2013	No	Yes	No	Yes	Yes	No	Yes	Yes	Yes	Yes	7	Low risk of bias
492	Hepatitis C virus infection in haemodialysis patients.	Roger	1991	No	Yes	No	Yes	Yes	No	Yes	Yes	Unclear	Yes	6	Moderate risk of bias
493	Health related quality of life in Iranian hemodialysis patients with viral hepatitis: changing epidemiology.	Rostami	2013	No	Yes	No	Yes	Yes	No	Yes	Yes	No	Yes	6	Moderate risk of bias
494	Hemodialysis as predisposing factor for viral hepatitis B and C.	Roşu	2015	No	Yes	No	Yes	Yes	No	Yes	Yes	Unclear	Yes	6	Moderate risk of bias
495	Prevalence and genotyping pattern of hepatitis C virus among patients on maintenance hemodialysis at five centers in Pune, India.	Roy	2019	No	Yes	No	Yes	Yes	No	Yes	Yes	Yes	Yes	7	Low risk of bias
496	Hepatitis C virus infection in hemodialyzed patients detected by first and second generation assays.	Ruffatti	1992	No	Yes	No	Yes	Yes	No	Yes	Yes	Yes	Yes	7	Low risk of bias
497	Serum alanine aminotransferase in hepatitis c screening of patients on hemodialysis.	Saab	2001	No	Yes	No	Yes	Yes	No	Yes	Yes	Yes	Yes	7	Low risk of bias
498	Effect of HCV infection on hematocrit and hemoglobin level in Egyptian hemodialysis patients.	Sabry	2009	No	Yes	No	Yes	Yes	No	Yes	Yes	Unclear	Yes	6	Moderate risk of bias
499	Hepatitis C virus infection in hemodialysis patients in jordan.	Said	1995	No	Yes	No	Yes	Yes	No	Yes	Yes	Unclear	Yes	6	Moderate risk of bias
500	Prevalence of hepatitis C virus infection among long-term hemodialysis patients: detection of hepatitis C virus RNA in plasma.	Sakamoto	1993	No	Yes	No	Yes	Yes	No	Yes	Yes	Unclear	Yes	6	Moderate risk of bias
501	Prevalence of hepatitis C virus infection among haemodialysis patients in West Java, Indonesia.	Saketi	2003	No	Yes	No	Yes	Yes	No	Yes	Yes	Unclear	Yes	6	Moderate risk of bias
502	Hepatitis C virus infection in French hemodialysis units: a multicenter study.	Salama	2000	No	Yes	No	Yes	Yes	No	Yes	Yes	Unclear	Yes	6	Moderate risk of bias
503	Hepatitis G virus exposure in dialysis patients and blood donors in Isfahan-Iran.	Salehi	2014	No	Yes	Yes	Yes	Yes	No	Yes	Yes	Unclear	Yes	7	Low risk of bias
504	Prevalence of antibodies to hepatitis C virus in HBsAg negative hemodialysis patients.	Salunkhe	1992	No	Yes	No	Yes	Yes	No	Yes	Yes	Unclear	Yes	6	Moderate risk of bias
505	Prevalence of hepatitis C virus antibody and related risk factors among hemodialysis patients in Markazi province (2004).	Samimi rad	2006	No	Yes	Unclear	Yes	Yes	Unclear	Yes	Yes	Unclear	Yes	6	Moderate risk of bias
506	Hepatitis C virus infection among multi-transfused patients and personnel in haemodialysis units in central Islamic Republic of Iran.	Samimi Rad	2012	No	Yes	No	Yes	Yes	No	Yes	Yes	No	Yes	6	Moderate risk of bias
507	Hepatitis C virus infection and HCV genotypes of hemodialysis patients.	Samimi Rad	2008	No	Yes	No	Yes	Yes	No	Yes	Yes	No	Yes	6	Moderate risk of bias
508	High prevalence of a rare hepatitis C virus in patients treated in the same hemodialysis unit: evidence for nosocomial transmission of HCV.	Sampietro	1995	No	Yes	No	Yes	Yes	No	Yes	Yes	Unclear	Yes	6	Moderate risk of bias
509	Hepatitis C virus antibodies in patients on hemodialysis.	San Miguel	1992	No	Yes	No	Yes	Yes	No	Yes	Yes	Unclear	Yes	6	Moderate risk of bias
510	Hepatitis C prevalence and risk factors in the northern Alberta dialysis population.	Sandhu	1999	No	Yes	No	Yes	Yes	No	Yes	Yes	Unclear	Yes	6	Moderate risk of bias
511	Hepatitis status and mortality in hemodialysis population.	Santoro	2009	No	Yes	No	Yes	Yes	No	Yes	Yes	Yes	Yes	7	Low risk of bias

512	Infection by the hepatitis C virus in chronic renal failure patients undergoing hemodialysis in Mato Grosso state, central Brazil: a cohort study.	Santos	2007	No	Yes	No	Yes	Yes	No	Yes	Yes	Yes	Yes	7	Low risk of bias
513	Prevalence of hepatitis B and hepatitis C in haemodialysis patients.	Santos	1998	No	Yes	No	Yes	Yes	No	Yes	Yes	No	Yes	6	Moderate risk of bias
514	Hepatitis C virus and hepatitis B virus infection in patients and staff of haemodialysis unit - A report from Chennai.	Saraswathy	2013	No	Yes	No	Yes	Yes	No	Yes	Yes	Unclear	Yes	6	Moderate risk of bias
515	Hepatitis C virus antibodies in dialysis patients in Tunisia: a single center study.	Sassi	2000	No	Yes	No	Yes	Yes	No	Yes	Yes	Unclear	Yes	6	Moderate risk of bias
516	Decreased prevalence and incidence of HCV markers in haemodialysis units: a multicentric French survey.	Sauné	2011	No	Yes	No	Yes	Yes	No	Yes	Yes	Unclear	Yes	6	Moderate risk of bias
517	A large nosocomial outbreak of hepatitis C virus infections at a hemodialysis center.	Savey	2005	No	Yes	No	Yes	Yes	No	Yes	Yes	Unclear	Yes	6	Moderate risk of bias
518	The impact of nurse understaffing on the transmission of hepatitis C virus in a hospital-based hemodialysis unit.	Saxena	2004	No	Yes	No	Yes	Yes	No	Yes	Yes	Yes	Yes	7	Low risk of bias
519	Prevalence of hepatitis C antibodies among hemodialysis patients in Al-hasa region of saudi arabia.	Saxena	2001	No	Yes	No	Yes	Yes	No	Yes	Yes	Unclear	Yes	6	Moderate risk of bias
520	The role the type of vascular access plays in the transmission of hepatitis C virus in a high prevalence hemodialysis unit.	Saxena	2002	No	Yes	No	Yes	Yes	No	Yes	Yes	Yes	Yes	7	Low risk of bias
521	Impact of dedicated space, dialysis equipment, and nursing staff on the transmission of hepatitis C virus in a hemodialysis unit of the middle east.	Saxena	2003	No	Yes	No	Yes	Yes	No	Yes	Yes	Yes	Yes	7	Low risk of bias
522	HCV infection in haemodialysis and CAPD patients.	Sayiner	1999	No	Yes	No	Yes	Yes	No	Yes	Yes	Unclear	Yes	6	Moderate risk of bias
523	Hepatitis B and C virus infection in the hemodialysis population from three romanian regions.	Schiller	2015	No	Yes	No	Yes	Yes	No	Yes	Yes	Unclear	Yes	6	Moderate risk of bias
524	Hepatitis C virus antibodies in haemodialysis patients.	Schlipköter	1990	No	Yes	No	Yes	Yes	No	Yes	Yes	Unclear	Yes	6	Moderate risk of bias
525	Prevalence of hepatitis C virus infections in dialysis patients and their contacts using a second generation enzymed-linked immunosorbent assay.	Schlipköter	1992	No	Yes	No	Yes	Yes	No	Yes	Yes	Unclear	Yes	6	Moderate risk of bias
526	Hepatitis C virus infections in dialysis centers in The Netherlands: a national survey by serological and molecular methods.	Schneeberger	1998	No	Yes	No	Yes	Yes	No	Yes	Yes	No	Yes	6	Moderate risk of bias
527	The prevalence and incidence of hepatitis C virus infections among dialysis patients in the Netherlands: a nationwide prospective study.	Schneeberger	2000	No	Yes	No	Yes	Yes	No	Yes	Yes	Yes	Yes	7	Low risk of bias
528	Prevalence of antibodies to hepatitis C virus in a Dutch group of haemodialysis patients related to risk factors.	Schneeberger	1993	No	Yes	No	Yes	Yes	No	Yes	Yes	No	Yes	6	Moderate risk of bias
529	Prolonged time until seroconversion among hemodialysis patients: the need for HCV PCR.	Schroeter	2005	No	Yes	No	Yes	Yes	No	Yes	Yes	Yes	Yes	7	Low risk of bias
530	GB virus C hepatitis G virus infection in hemodialysis patients: Determination of seroprevalence by a four-antigen recombinant immunoblot assay.	Schroter	1999	No	Yes	No	Yes	Yes	No	Yes	Yes	Unclear	Yes	6	Moderate risk of bias
531	High percentage of seronegative HCV infections in hemodialysis patients: the need for PCR.	Schröter	1997	No	Yes	No	Yes	Yes	No	Yes	Yes	Unclear	Yes	6	Moderate risk of bias
532	HCV in a group of chronic hemodialysis patients.	Scipioni	1992	No	Yes	No	Yes	Yes	No	Yes	Yes	Unclear	Yes	6	Moderate risk of bias
533	Adverse impact of hepatitis C virus infection on renal replacement therapy and renal transplant patients in Australia and New Zealand	Scott	2010	No	Yes	No	Yes	Yes	No	Yes	Yes	Yes	Yes	7	Low risk of bias
534	HCV infections in dialysis patients.	Scotto	1992	No	Yes	No	Yes	Yes	No	Yes	Yes	Unclear	Yes	6	Moderate risk of bias
535	Trends in hepatitis C infection among hemodialysis patients in Senegal: results of a decade of prevention.	Seck	2014	No	Yes	No	Yes	Yes	No	Yes	Yes	Unclear	Yes	6	Moderate risk of bias
536	Hepatitis C virus infections in dialysis units: prevalence of HCV-RNA and antibodies to HCV.	Seelig	1994	No	Yes	No	Yes	Yes	No	Yes	Yes	Unclear	Yes	6	Moderate risk of bias
537	[Prevalence of anti-HCV antibodies and seroconversion incidence in five haemodialysis units in Morocco].	Sekkat	2008	No	Yes	No	Yes	Yes	No	Yes	Yes	Yes	Yes	7	Low risk of bias
538	Prevalence of hepatitis C antibodies (HCV) in a dialysis population at one center.	Selgas	1992	No	Yes	No	Yes	Yes	No	Yes	Yes	Unclear	Yes	6	Moderate risk of bias
539	Prevalence of hepatitis C virus infection among hemodialysis patients in a single center in Yemen.	Selm	2010	No	Yes	No	Yes	Yes	No	Yes	Yes	Unclear	Yes	6	Moderate risk of bias
540	Molecular evidence for nosocomial spread of two different hepatitis C virus strains in one hemodialysis unit.	Seme	1997	No	Yes	No	Yes	Yes	No	Yes	Yes	Yes	Yes	7	Low risk of bias
541	High prevalence of hepatitis C virus infection in hemodialysis patients from one dialysis unit in Slovenia.	Seme	1995	No	Yes	No	Yes	Yes	No	Yes	Yes	Unclear	Yes	6	Moderate risk of bias
542	Hepatitis C virus in patients on regular hemodialysis in Beni-Suef Governorate, Egypt	Senosy	2016	No	Yes	No	Yes	Yes	No	Unclear	Unclear	No	Yes	4	Moderate risk of bias
543	Hepatitis C infection in hemodialysis patients: Protective against oxidative stress?	Sezer	2006	No	Yes	No	Yes	Yes	No	Yes	Yes	Unclear	Yes	6	Moderate risk of bias
544	Seroprevalence of Hepatitis B Virus and Hepatitis C Virus in Patients Undergoing Maintenance Hemodialysis.	Shabbir	2022	No	Yes	No	Yes	Yes	No	Yes	Yes	Unclear	Yes	6	Moderate risk of bias
545	Prevalence of Hepatitis C in Dialysis Patients in Khyber Teaching Hospital Peshawar.	Shah	2022	No	Yes	No	Yes	Yes	No	Yes	Yes	No	Yes	6	Moderate risk of bias
546	Prevalence of hepatitis C antibodies among hemodialysis patients in the Western province of saudi arabia.	Shaheen	1995	No	Yes	No	Yes	Yes	No	Yes	Yes	Unclear	Yes	6	Moderate risk of bias
547	Prevalence of HBV, HCV, and HIV Infections among Patients Undergoing Hemodialysis in Fasa, Iran: A Six-Year Follow-up Study.	Shamsdin	2022	No	Yes	No	Yes	Yes	Yes	Yes	Yes	Yes	Yes	8	Low risk of bias

548	Evaluation of immunogenicity of hepatitis B vaccine in hemodialysis patients at Mazandaran Heart Center, Iran.	Shamshirian	2017	No	Yes	No	Yes	Yes	No	Yes	Yes	Unclear	Yes	6	Moderate risk of bias
549	The role of hemodialysis machines dedication in reducing Hepatitis C transmission in the dialysis setting in Iran: a multicenter prospective interventional study.	Shamshirsaz	2004	No	Yes	No	Yes	Yes	No	Yes	Yes	Unclear	Yes	6	Moderate risk of bias
550	High prevalence of hepatitis G virus infection compared with hepatitis C virus infection in patients undergoing chronic hemodialysis.	Sheng	1998	No	Yes	No	Yes	Yes	No	Yes	Yes	Unclear	Yes	6	Moderate risk of bias
551	Prevalence of anti-HCV and HCV viremia in hemodialysis patients in Taiwan.	Sheu	1992	No	Yes	No	Yes	Yes	No	Yes	Yes	Unclear	Yes	6	Moderate risk of bias
552	Patient-care practices associated with an increased prevalence of hepatitis C virus infection among chronic hemodialysis patients.	Shimokura	2011	No	Yes	No	Yes	Yes	No	Yes	Yes	No	Yes	6	Moderate risk of bias
553	Infection with GB virus C and hepatitis C virus in drug addicts, patients on maintenance hemodialysis, or with chronic liver disease in Nepal.	Shrestha	1997	No	Yes	No	Yes	Yes	No	Yes	Yes	Unclear	Yes	6	Moderate risk of bias
554	Virological features of hepatitis C virus infection in hemodialysis patients.	Silini	1993	No	Yes	No	Yes	Yes	No	Yes	Yes	Unclear	Yes	6	Moderate risk of bias
555	Prevalence of hepatitis C virus (HCV) infection and HCV genotypes of hemodialysis patients in Salvador, Northeastern Brazil.	Silva	2006	No	Yes	No	Yes	Yes	No	Yes	Yes	No	Yes	6	Moderate risk of bias
556	A twelve year natural history of hepatitis C virus infection in hemodialyzed patients.	Simon	1994	No	Yes	No	Yes	Yes	No	Yes	Yes	Yes	Yes	7	Low risk of bias
557	Seroprevalence of hepatitis B and C viruses in patients with chronic kidney disease in the predialysis stage at a university hospital in Turkey.	Sit	2007	No	Yes	No	Yes	Yes	No	Yes	Yes	No	Yes	6	Moderate risk of bias
558	High prevalence of hepatitis C infection among patients receiving hemodialysis at an urban dialysis center.	Sivapalasingam	2002	No	Yes	No	Yes	Yes	No	Yes	Yes	No	Yes	6	Moderate risk of bias
559	Prevalence of anti-HCV positivity in hemodialysis patients.	Sivrel Arisoy	2000	No	Yes	No	Yes	Yes	No	Yes	Yes	Unclear	Yes	6	Moderate risk of bias
560	[Hepatitis B (HBV) and C (HCV) virus infections as an eventual cause of chronic hepatic damage in patients undergoing maintenance hemodialysis].	Slizien	1995	No	Yes	No	Yes	Yes	Unclear	Yes	Yes	Unclear	Yes	6	Moderate risk of bias
561	Differential prevalence of hepatitis C virus subtypes in healthy blood donors, patients on maintenance hemodialysis, and patients with hepatocellular carcinoma in Surabaya, Indonesia	Soetjipto	1996	No	Yes	No	Yes	Yes	No	Yes	Yes	Unclear	Yes	6	Moderate risk of bias
562	Risk factors of HCV seroconversion in hemodialysis patients in tabriz, iran.	Somi	2014	No	Yes	No	Yes	Yes	No	Yes	Yes	No	Yes	6	Moderate risk of bias
563	Hepatitis C virus genotypes in patients with end-stage renal disease in East Azerbaijan, Iran.	Somi	2008	No	Yes	No	Yes	Yes	No	Yes	Yes	No	Yes	6	Moderate risk of bias
564	A cost-identification analysis of screening and surveillance of hepatitis C infection in a prospective cohort of dialysis patients.	Somsouk	2008	No	Yes	No	Yes	Yes	No	Yes	Yes	Yes	Yes	7	Low risk of bias
565	Hepatitis C virus antibodies among risk groups in a South African area endemic for hepatitis B virus.	Soni	1993	No	Yes	No	Yes	Yes	No	Yes	Yes	No	Yes	6	Moderate risk of bias
566	The annual incidence of seroconversion of antibodies to the hepatitis C virus in the hemodialysis population in saudi arabia.	Souqiyyeh	1995	No	Yes	No	Yes	Yes	No	Yes	Yes	Yes	Yes	7	Low risk of bias
567	Hepatitis B and C in the hemodialysis unit of Tocantins, Brazil: serological and molecular profiles.	Souza	2003	No	Yes	No	Yes	Yes	No	Yes	Yes	No	Yes	6	Moderate risk of bias
568	Risk of death among chronic dialysis patients infected with hepatitis C virus	Stehman-Breen	1998	No	Yes	No	Yes	Yes	No	Yes	Yes	Yes	Yes	7	Low risk of bias
569	Prevalence and risk factors of hepatitis C and B virus infections in hemodialysis patients and their spouses: a multicenter study in Beijing, China.	Su	2013	No	Yes	Yes	Yes	Yes	No	Yes	Yes	No	Yes	7	Low risk of bias
570	Prevalence of hepatitis C virus infection in hemodialysis patients in Sudan.	Suliman	1995	No	Yes	No	Yes	Yes	No	Yes	Yes	No	Yes	6	Moderate risk of bias
571	[Occurrence of antibodies against hepatitis C virus (anti-HCV) in patients on long-term hemodialysis].	Sułowicz	1992	No	Yes	No	Yes	Yes	Unclear	Yes	Yes	Unclear	Yes	6	Moderate risk of bias
572	Incidence and patterns of hepatitis C virus seroconversion in a cohort of hemodialysis patients.	Sypsa	2005	No	Yes	No	Yes	Yes	No	Yes	Yes	Yes	Yes	7	Low risk of bias
573	An archived serum sample as a clue for identifying the primary source of a nosocomial hepatitis C virus outbreak in a haemodialysis unit.	Szücs	2014	No	Yes	No	Yes	Yes	No	Yes	Yes	No	Yes	6	Moderate risk of bias
574	Hepatitis C virus infection in chronic haemodialysis patients--relationship to blood transfusions and dialyser re-use.	Taal	2000	No	Yes	No	Yes	Yes	No	Yes	Yes	No	Yes	6	Moderate risk of bias
575	Prevalence of hepatitis C and B virus infections among hemodialysis patients in Karaj, Iran.	Tajbakhsh	2015	No	Yes	No	Yes	Yes	No	Yes	Yes	No	Yes	6	Moderate risk of bias
576	Prevalence of four blood-borne viruses (HBV, HCV, HTLV-I, HIV-1) among haemodialysis patients in Japan.	Tamura	1992	No	Yes	No	Yes	Yes	No	Yes	Yes	No	Yes	6	Moderate risk of bias
577	[Detection of TT virus (TTV) in Japanese hemodialysis (HD) patients].	Tanaka	1999	No	Yes	No	Yes	Yes	Unclear	Yes	Yes	Unclear	Yes	6	Moderate risk of bias
578	Hepatitis E virus infection in hemodialysis patients: a seroepidemiological survey in Iran.	Taremi	2005	No	Yes	No	Yes	Yes	No	Yes	Yes	No	Yes	6	Moderate risk of bias
579	Prevalence of hepatitis C virus infection in hemodialysis patients.	Taziki	2008	No	Yes	No	Yes	Yes	No	Yes	Yes	Yes	Yes	7	Low risk of bias
580	Hepatitis B and C in dialysis units in Kosova.	Telaku	2009	No	Yes	No	Yes	Yes	No	Yes	Yes	Yes	Yes	7	Low risk of bias
581	[Hepatitis C virus infection in patients treated with hemodialysis].	Teruel	1990	No	Yes	No	Yes	Yes	Unclear	Yes	Yes	Unclear	Yes	6	Moderate risk of bias
582	Viral hepatitis infections among dialysis patients: Thailand registry report.	Thanachartwet	2007	No	Yes	No	Yes	Yes	No	Yes	Yes	Yes	Yes	7	Low risk of bias
583	Occult hepatitis C virus infection during an outbreak in a hemodialysis unit in Thailand.	Thongsawat	2008	No	Yes	No	Yes	Yes	No	Yes	Yes	Unclear	Yes	6	Moderate risk of bias
584	The role of dedicated reuse machine for anti HCV positive patients in reducing the incidence of hepatitis C	Tjiang	2016	No	Yes	No	Yes	Yes	No	Yes	Yes	Yes	Yes	7	Low risk of bias

	infection in hemodialysis unit: A two year prospective study.														
585	High prevalence of hepatitis C virus infection in one dialysis center in Bulgaria.	Todorov	1998	No	Yes	No	Yes	Yes	No	Yes	Yes	Unclear	Yes	6	Moderate risk of bias
586	Risk factors and seroprevalence of hepatitis B and C infections among hemodialysis patients in Tehran	Toosi	2007	No	Yes	No	Yes	Yes	No	Unclear	Yes	Unclear	Yes	5	Moderate risk of bias
587	High frequency of antibodies to Hantaan virus and hepatitis C virus in chronic haemodialysis patients. Coincidence or cross-reaction?	Tsianos	1993	No	Yes	No	Yes	Yes	No	Yes	Yes	Unclear	Yes	6	Moderate risk of bias
588	[Prevalence of hepatitis C virus infection among chronic hemodialysis patients].	Tsuyuguchi	1994	No	Yes	No	Yes	Yes	Unclear	Yes	Yes	Unclear	Yes	6	Moderate risk of bias
589	Prevalence and incidence of hepatitis C virus in hemodialysis patients in British Columbia: Follow-up after a possible breach in hemodialysis machines.	Tu	2009	No	Yes	No	Yes	Yes	No	Yes	Yes	No	Yes	6	Moderate risk of bias
590	[Hepatitis E virus seropositivity in hemodialysis patients in Hatay province, Turkey].	Uçar	2009	No	Yes	No	Yes	Yes	Unclear	Yes	Yes	Unclear	Yes	6	Moderate risk of bias
591	Risk factors for hepatitis C virus sero-positivity among haemodialysis patients receiving care at kidney centre in a tertiary health facility in Maiduguri, Nigeria.	Ummate	2014	No	Yes	No	Yes	Yes	No	Yes	Yes	Unclear	Yes	6	Moderate risk of bias
592	Prevalence of Hepatitis C virus infection among haemodialysis patients in North-Eastern Nigeria.	Ummate	2013	No	Yes	No	Yes	Yes	No	Unclear	Yes	Unclear	Yes	5	Moderate risk of bias
593	HBV and HCV infections in relation to the chosen genetic features in hemodialyzed patients.	Urbanowicz	2000	No	Yes	No	Yes	Yes	Unclear	Yes	Yes	Unclear	Yes	6	Moderate risk of bias
594	Screening of relatives of anti-hepatitis-C virus positive hemodialysed patients (preliminary data).	Urso	1996	No	Yes	No	Yes	Yes	Unclear	Yes	Yes	Unclear	Yes	6	Moderate risk of bias
595	PREVALENCE AND RISK FACTORS OF HEPATITIS B AND C VIRUS INFECTIONS AMONG HEMODIALYSIS PATIENTS FROM PRIVATE HEMODIALYSIS UNITS IN SURABAYA, INDONESIA.	Utsumi	2016	No	Yes	No	Yes	Yes	Unclear	Yes	Yes	Yes	Yes	7	Low risk of bias
596	TT virus infection in hemodialysis patients.	Utsunomiya	1999	No	Yes	Yes	Yes	Yes	Unclear	Yes	Yes	No	Yes	7	Low risk of bias
597	The role of transfusion-transmitted virus in patients undergoing hemodialysis.	Valtuille	2002	No	Yes	No	Yes	Yes	Unclear	Yes	Yes	Unclear	Yes	6	Moderate risk of bias
598	Behavior of antibody profile against hepatitis C virus in patients on maintenance hemodialysis.	Vandelli	1992	No	Yes	No	Yes	Yes	Yes	Yes	Yes	Yes	Yes	8	Low risk of bias
599	High prevalence of hepatitis C infection among Brazilian hemodialysis patients in Rio de Janeiro: a one-year follow-up study.	Vanderborght	1995	No	Yes	No	Yes	Yes	Yes	Yes	Yes	Unclear	Yes	7	Low risk of bias
600	Prevalence of isolated hepatitis B core antibody and occult hepatitis B among adult patients initiating hemodialysis at the Philippine General Hospital (PGH) Dialysis Unit.	Velasquez	2011	No	Yes	No	Yes	Yes	No	Yes	Yes	Unclear	Yes	6	Moderate risk of bias
601	Detection of hepatitis C virus in patients with terminal renal disease undergoing dialysis in southern Brazil: prevalence, risk factors, genotypes, and viral load dynamics in hemodialysis patients.	Vidales-Braz	2015	No	Yes	No	Yes	Yes	Unclear	Yes	Yes	Yes	Yes	7	Low risk of bias
602	Seroprevalence of hepatitis C infection in patients undergoing haemodialysis in a tertiary care centre.	Vinayakumar	2020	No	Yes	No	Yes	Yes	Unclear	Yes	Yes	Unclear	Yes	6	Moderate risk of bias
603	Epidemiology of hepatitis C virus infection in dialysis units: first-versus second-generation assays.	Vitale	1993	No	Yes	No	Yes	Yes	Unclear	Yes	Yes	Unclear	Yes	6	Moderate risk of bias
604	Infections with hepatitis B and C viruses in patients on maintenance dialysis in Romania and in former communist countries: yellow spots on a blank map?	Vladutiu	2000	No	Yes	No	Yes	Yes	Unclear	Yes	Yes	Yes	Yes	7	Low risk of bias
605	A cross-sectional epidemiological study of HBV, HCV, HDV and HEV prevalence in the SubCarpathian and South-Eastern regions of Romania.	Voiculescu	2010	No	Yes	No	Yes	Yes	Yes	Yes	Yes	Yes	Yes	8	Low risk of bias
606	Follow-up study of hepatitis C virus infection in uremic patients on maintenance hemodialysis for 30 months.	Wang	2000	No	Yes	No	Yes	Yes	Unclear	Yes	Yes	Yes	Yes	7	Low risk of bias
607	Infection with GB virus C and hepatitis C virus in hemodialysis patients and blood donors in Beijing.	Wang	1997	No	Yes	No	Yes	Yes	Unclear	Yes	Yes	No	Yes	6	Moderate risk of bias
608	Mortality in hepatitis C-positive patients treated with peritoneal dialysis	Wang	2008	No	Yes	No	Yes	Yes	No	Yes	Yes	Yes	Yes	7	Low risk of bias
609	GB virus C and hepatitis C virus infections in hemodialysis patients in eight Japanese centers.	Watanabe	1997	No	Yes	No	Yes	Yes	Unclear	Yes	Yes	No	Yes	6	Moderate risk of bias
610	Seroprevalence of HCV, HAV, HBV, HDV, HCMV and HIV in high risk groups/Frankfurt a.M., Germany.	Weber	1995	No	Yes	No	Yes	Yes	Unclear	Yes	Yes	Yes	Yes	7	Low risk of bias
611	[Detection of serum HCV RNA and HBV DNA in patients on hemodialysis].	Wei	1996	No	Yes	No	Yes	Yes	Unclear	Yes	Yes	Unclear	Yes	6	Moderate risk of bias
612	Evolutionary seroepidemiology of viral hepatitis and the gap in hepatitis C care cascades among uraemic patients receiving haemodialysis in Taiwan-the Formosa-Like Group.	Wei	2021	No	Yes	No	Yes	Yes	Yes	Yes	Yes	Unclear	Yes	7	Low risk of bias
613	Prevalence of antibodies to hepatitis C virus (anti-HCV) in different populations in Taiwan.	Wu	1991	No	Yes	No	Yes	Yes	Unclear	Yes	Yes	Unclear	Yes	6	Moderate risk of bias
614	[Detection of hepatitis C virus antigen in hemodialysis patients].	Wu	2009	No	Yes	No	Yes	Yes	Unclear	Yes	Yes	Unclear	Yes	6	Moderate risk of bias
615	Seroprevalence of hepatitis B, C and D viral among hemodialysis patients in Tehran.	Yadegarynia	2017	No	Yes	No	Yes	Yes	Unclear	Yes	Yes	Unclear	Yes	6	Moderate risk of bias
616	Prevalence of occult hepatitis B and hepatitis C virus infections in Turkish hemodialysis patients.	Yakaryilmaz	2006	No	Yes	No	Yes	Yes	Unclear	Yes	Yes	Unclear	Yes	6	Moderate risk of bias
617	Long term survey of hepatitis C virus infection in hemodialysis units in Fukuoka, Japan.	Yamaji	1996	No	Yes	No	Yes	Yes	Unclear	Yes	Yes	Yes	Yes	7	Low risk of bias
618	Seroprevalence of antibodies to the hepatitis C virus in Singapore.	Yap	1991	No	Yes	No	Yes	Yes	Unclear	Yes	Yes	Unclear	Yes	6	Moderate risk of bias
619	High prevalence of hepatitis C virus antibody in patients with chronic renal failure at the start of hemodialysis therapy.	Yonemura	1996	No	Yes	No	Yes	Yes	Unclear	Yes	Yes	Unclear	Yes	6	Moderate risk of bias

620	Hepatitis C virus in chronic hemodialysis patients with non-A, non-B hepatitis.	Yoshida	1992	No	Yes	Yes	Yes	Yes	Unclear	Yes	Yes	Unclear	Yes	7	Low risk of bias
621	Antibodies against non-structural c100/3 and structural core antigen of hepatitis C virus (HCV) in hemodialysis patients.	Yoshida	1993	No	Yes	Yes	Yes	Yes	Unclear	Yes	Yes	No	Yes	7	Low risk of bias
622	Nosocomial Transmission of Hepatitis B Surface Antigen and Anti-Hepatitis C Virus among Hemodialysis Patients.	Yousif	2022	No	Yes	No	Yes	Yes	No	Yes	Yes	No	Yes	6	Moderate risk of bias
623	Hepatitis C virus and carpal tunnel syndrome in hemodialysis patients: a single center cross-sectional study.	Yu	2020	No	Yes	No	Yes	Yes	Unclear	Yes	Yes	No	Yes	6	Moderate risk of bias
624	Establishment of an outreach, grouping healthcare system to achieve microelimination of HCV for uremic patients in haemodialysis centres (ERASE-C).	Yu	2021	No	Yes	No	Yes	Yes	No	Unclear	Yes	Unclear	Yes	5	Moderate risk of bias
625	Quality control measures for lowering the seroconversion rate of hemodialysis patients with hepatitis B or C virus.	Yuan	2012	No	Yes	No	Yes	Yes	Unclear	Yes	Yes	Yes	Yes	7	Low risk of bias
626	Seroprevalence of Hepatitis Viruses B, C, D and HIV Infection Among Hemodialysis Patients in Kerman Province, South-East Iran.	Zahedi	2012	No	Yes	No	Yes	Yes	Yes	Yes	Yes	Unclear	Yes	7	Low risk of bias
627	Prevalence of seroconversion of hepatitis C virus among hemodialysis patients in Menoufia Governorate, Egypt.	Zahran	2014	No	Yes	No	Yes	Yes	Unclear	Yes	Yes	Unclear	Yes	6	Moderate risk of bias
628	[Low prevalence of hepatitis G infection in dialysis patients in Israel].	Zamir	1999	No	Yes	No	Yes	Yes	Unclear	Yes	Yes	Unclear	Yes	6	Moderate risk of bias
629	Hepatitis C virus seroconversion and genotype prevalence in patients and staff on chronic hemodialysis.	Zamir	1999	No	Yes	No	Yes	Yes	Unclear	Yes	Yes	Unclear	Yes	6	Moderate risk of bias
630	The prevalence of hepatitis C virus antibodies among hemodialysis patients.	Zeldis	1990	No	Yes	No	Yes	Yes	Unclear	Yes	Yes	Unclear	Yes	6	Moderate risk of bias
631	Phylogenetic analysis of hepatitis C virus isolates from hemodialysis patients.	Zeuzem	1996	No	Yes	No	Yes	Yes	Unclear	Yes	Yes	Unclear	Yes	6	Moderate risk of bias
632	Tumor Necrosis Factor-alpha Promoter Gene Polymorphisms Are Not Associated with Hepatitis C Virus Infection in Chinese Hemodialysis Patients.	Zhang	2011	No	Yes	No	Yes	Yes	Unclear	Yes	Yes	Unclear	Yes	6	Moderate risk of bias
633	Baseline data report of the China Dialysis Outcomes and Practice Patterns Study (DOPPS).	Zhao	2021	No	Yes	Yes	Yes	Yes	Unclear	Yes	Yes	Unclear	Yes	7	Low risk of bias
634	Prevalence of HCV infection in hemodialysis patients of South Khorasan in comparison with HBV, HDV, HTLV I/II, And HIV infection	Ziaee	2014	No	Yes	No	Yes	Yes	No	Yes	Yes	No	Yes	6	Moderate risk of bias
