# Supplementary material for: Global epidemiology of hepatitis C virus in dialysis patients: A systematic review and meta-analysis
Source: PLoS One. 2024 Feb 8;19(2):e0284169. doi: 10.1371/journal.pone.0284169 (PMC10852299; doi:10.1371/journal.pone.0284169)
Supplement: S8 Table — (PDF) [file pone.0284169.s009.pdf]

S8 Table: Subgroup analyses of prevalence of hepatitis C virus in hemodialysis patients

	**Prevalence. % (95%CI)**	**95% Prediction interval**	**N Studies**	**N Participants**	**H (95%CI)**	**I² (95%CI)**	**P heterogeneity**	**P difference subtypes**
**HCV prevalence in HD**								
**Study Design**								0.122
Case control	14.4 [6.7-24.1]	[0-66.3]	4	849	2.6 [1.7-4.1]	85.3 [63.7-94.1]	<0.001	
Cohort (Baseline data)	25.7 [21.2-30.4]	[0.8-67]	65	73805	13.4 [12.9-13.9]	99.4 [99.4-99.5]	<0.001	
Cross-sectional	24.2 [22.4-26]	[0-72.7]	597	317425	11.5 [11.3-11.6]	99.2 [99.2-99.3]	<0.001	
**Sampling**								0.893
Non probabilistic	24.3 [22.7-26]	[0-69.3]	601	288726	10.3 [10.2-10.5]	99.1 [99-99.1]	<0.001	
Probabilistic	23.9 [18.1-30.1]	[0-78.7]	66	103434	16 [15.5-16.6]	99.6 [99.6-99.6]	<0.001	
**Rural/Urban**								0.759
Rural	26.2 [20-32.9]	[1.6-65.1]	28	5418	5.2 [4.7-5.8]	96.4 [95.5-97]	<0.001	
Urban	25.1 [23.2-27]	[0.8-65.6]	388	161745	8.6 [8.4-8.8]	98.6 [98.6-98.7]	<0.001	
**Countries**								<0.001
Indonesia	63.6 [42.9-82]	[2-100]	8	970	6.3 [5.3-7.5]	97.5 [96.4-98.2]	<0.001	
Romania	55.3 [30.8-78.5]	[0-100]	7	2778	11.5 [10-13.1]	99.2 [99-99.4]	<0.001	
Egypt	55.2 [47.1-63.2]	[26.9-81.9]	9	7578	6 [5-7.1]	97.2 [96-98]	<0.001	
Kosovo	48.2 [41-55.5]	[0-100]	3	1386	2.5 [1.5-4.4]	84.4 [53.3-94.8]	0.002	
Poland	46.7 [37.9-55.5]	[19.4-75]	7	629	2.2 [1.5-3.1]	79.3 [57.5-89.9]	<0.001	
Saudi Arabia	44.2 [36-52.6]	[4.2-89.7]	36	7000	6.7 [6.2-7.3]	97.8 [97.4-98.1]	<0.001	
Yemen	43.7 [33.6-54]	[7.5-84.3]	4	443	1.9 [1.2-3.3]	73.7 [26.2-90.6]	0.01	
Morocco	41.9 [28.7-55.7]	[2.3-90]	11	2062	6.1 [5.2-7.1]	97.3 [96.3-98]	<0.001	
Senegal	40.9 [1.4-89.8]	[0-100]	3	143	5.1 [3.5-7.4]	96.1 [91.8-98.2]	<0.001	
Serbia	38.3 [25.4-52.1]	[2.9-83.7]	6	564	3 [2.2-4.2]	89.1 [78.9-94.4]	<0.001	
Pakistan	36.7 [30.1-43.5]	[11.3-66.9]	18	2723	3.5 [3-4.1]	91.8 [88.5-94.1]	<0.001	
Kuwait	36.1 [4.4-77.4]	[0-100]	3	1695	12.6 [10.2-15.5]	99.4 [99-99.6]	<0.001	
Portugal	33.7 [19.5-49.4]	[0-96.9]	4	1664	5.1 [3.7-7]	96.1 [92.8-97.9]	<0.001	
Taiwan	31.3 [23.1-40]	[4.7-67.6]	13	7317	6.5 [5.6-7.4]	97.6 [96.8-98.2]	<0.001	
Thailand	29.9 [0.1-80]	[0-100]	6	8916	41.6 [39.3-44]	99.9 [99.9-99.9]	<0.001	
Peru	28.8 [2.3-68.4]	[0-100]	3	422	8.3 [6.3-11]	98.6 [97.5-99.2]	<0.001	
Turkey	28.7 [20-38.2]	[0-78.3]	24	3746	5.9 [5.3-6.5]	97.1 [96.4-97.7]	<0.001	
Italy	28.6 [25.7-31.6]	[8.5-54.4]	74	37208	5.7 [5.3-6]	96.9 [96.5-97.2]	<0.001	
Japan	25.6 [21.7-29.7]	[5.3-54]	40	23531	6.9 [6.4-7.4]	97.9 [97.6-98.2]	<0.001	
Jordan	25.2 [12.9-39.9]	[0-80.4]	6	5939	10.3 [8.8-12]	99 [98.7-99.3]	<0.001	

	Prevalence. % (95%CI)	95% Prediction interval	N Studies	N Participants	H (95%CI)	I² (95%CI)	P heterogeneity	P difference subtypes
France	24.3 [15.8-33.9]	[0.1-68.5]	15	82320	14.4 [13.4-15.5]	99.5 [99.4-99.6]	<0.001	
Brazil	24 [19-29.4]	[1.2-61.9]	40	13870	7.1 [6.6-7.6]	98 [97.7-98.3]	<0.001	
Tunisia	23.3 [13.1-35.4]	[0-72.2]	10	16949	12.8 [11.6-14.1]	99.4 [99.3-99.5]	<0.001	
Venezuela	22.9 [2.3-54.3]	[0-100]	5	637	7.2 [5.8-9]	98.1 [97-98.8]	<0.001	
Libya	22.8 [13.6-33.5]	[0.2-64.7]	6	5752	7.9 [6.6-9.5]	98.4 [97.7-98.9]	<0.001	
Spain	22.5 [16.6-28.9]	[0.3-62.2]	30	17270	8.5 [7.9-9.2]	98.6 [98.4-98.8]	<0.001	
Argentina	22.1 [17.4-27.2]	[5.1-46.4]	4	2698	2.5 [1.6-4]	84 [59.7-93.6]	<0.001	
United States of America	20.5 [16.6-24.7]	[4.3-44.2]	25	27968	7.2 [6.6-7.9]	98.1 [97.7-98.4]	<0.001	
India	19.3 [14.9-24.1]	[0.1-56.6]	46	9359	5.5 [5.1-6]	96.7 [96.1-97.2]	<0.001	
China	18.2 [13.9-22.8]	[3.2-41]	18	14923	6.1 [5.4-6.8]	97.3 [96.6-97.9]	<0.001	
Greece	17.6 [9.5-27.6]	[0-57.6]	7	1755	4.8 [3.8-6]	95.6 [93-97.2]	<0.001	
Israel	16.7 [11.2-23]	[0-90.2]	3	453	1.5 [1-2.7]	53 [0-86.5]	0.119	
Vietnam	15.9 [7.4-26.7]	[0-74.1]	4	968	3.7 [2.5-5.3]	92.5 [84.1-96.5]	<0.001	
Sudan	13.3 [7.1-21]	[0-44.8]	6	1103	3.2 [2.3-4.4]	90.2 [81.5-94.9]	<0.001	
Iraq	12.6 [6.5-20.3]	[0-100]	3	759	2.7 [1.6-4.6]	86.4 [60.9-95.3]	0.001	
South Africa	11.3 [3.9-21.6]	[0-100]	3	317	2.5 [1.4-4.3]	83.9 [51.5-94.6]	0.002	
Germany	10.5 [6.9-14.8]	[0-34.4]	19	9285	5.7 [5.1-6.5]	97 [96.1-97.6]	<0.001	
Iran	10.4 [7.6-13.5]	[0-37.2]	46	19114	6.4 [6-6.9]	97.6 [97.2-97.9]	<0.001	
Sweden	10.4 [5.7-16.1]	[0-32.7]	6	843	2.3 [1.5-3.3]	80.6 [58-91]	<0.001	
Nigeria	9.4 [4.1-16.5]	[0-50.6]	4	631	2.4 [1.5-3.9]	83.3 [57.5-93.4]	<0.001	
Mexico	8.8 [5.9-12.2]	[0-45.5]	3	384	1.1 [1-3.4]	16.2 [0-91.3]	0.303	
United Kingdom	8.3 [3.9-14]	[0-35.2]	5	1042	2.9 [2-4.2]	88 [74.6-94.4]	<0.001	
Denmark	6.4 [3.4-10.1]	[0-75.5]	3	884	2 [1.1-3.7]	75.7 [19.9-92.6]	0.016	
South Korea	6.3 [3.8-9.4]	[0.2-18.7]	7	6052	3.7 [2.8-4.8]	92.7 [87.4-95.7]	<0.001	
Australia	5 [0.4-13.5]	[0-49]	6	21413	7.8 [6.4-9.4]	98.3 [97.6-98.9]	<0.001	
Soa	3.2 [1.2-6]	[NA-NA]	1	220	NA [NA-NA]	NA [NA-NA]	1	
Netherlands	3.1 [2.4-3.9]	[1-6.2]	4	5253	1.3 [1-2.2]	40.5 [0-79.9]	0.169	
**WHO Region**								<0.001
Eastern Mediterranean	27.9 [24.3-31.6]	[0-77.2]	165	72324	10.7 [10.4-10.9]	99.1 [99.1-99.2]	<0.001	
South-East Asia	25.3 [16.2-35.6]	[0-98.6]	64	19490	15 [14.5-15.5]	99.6 [99.5-99.6]	<0.001	
Europe	24.6 [21.9-27.4]	[0-71.1]	235	170654	11.8 [11.6-12.1]	99.3 [99.3-99.3]	<0.001	
America	21.2 [18.1-24.3]	[1.6-53.6]	84	47857	7.9 [7.5-8.2]	98.4 [98.2-98.5]	<0.001	
Western Pacific	20.9 [17.8-24.1]	[0.8-56.4]	94	77113	10.3 [9.9-10.7]	99.1 [99-99.1]	<0.001	
Africa	12.8 [8-18.5]	[0-42.3]	18	1988	3.3 [2.8-4]	91 [87.3-93.6]	<0.001	

	Prevalence. % (95%CI)	95% Prediction interval	N Studies	N Participants	H (95%CI)	I² (95%CI)	P heterogeneity	P difference subtypes
**UNSD Region**								<0.001
Eastern Europe	48.6 [35.2-62]	[1.7-97.4]	18	3868	7.8 [7.1-8.6]	98.4 [98-98.7]	<0.001	
Southeastern Asia	35.5 [16.5-57.2]	[0-100]	24	11424	20.6 [19.7-21.5]	99.8 [99.7-99.8]	<0.001	
Western Asia	34.9 [29.3-40.6]	[0.2-86]	88	22186	8.6 [8.2-9]	98.6 [98.5-98.8]	<0.001	
Northern Africa	32.9 [25.7-40.5]	[0.5-82.4]	42	33444	13.4 [12.8-14]	99.4 [99.4-99.5]	<0.001	
Southern Europe	27.8 [25.3-30.5]	[5.1-59.3]	131	60714	6.8 [6.5-7.1]	97.8 [97.7-98]	<0.001	
South America	23.3 [19-27.9]	[0.9-61.4]	55	18030	7 [6.6-7.5]	98 [97.7-98.2]	<0.001	
Eastern Asia	22.9 [19.9-26.1]	[2.9-53.9]	78	51823	8.2 [7.9-8.6]	98.5 [98.4-98.7]	<0.001	
Northern America	19.1 [15.5-22.9]	[4-41.5]	27	29590	7 [6.4-7.7]	98 [97.6-98.3]	<0.001	
West Africa	18.3 [9.2-29.5]	[0-61.9]	8	840	3.5 [2.7-4.5]	91.8 [86.2-95.1]	<0.001	
Southern Asia	17.6 [14.8-20.6]	[0-55.1]	113	31330	6.7 [6.4-7]	97.8 [97.6-97.9]	<0.001	
Western Europe	13.4 [10.4-16.8]	[0.2-40.2]	43	99656	10 [9.5-10.6]	99 [98.9-99.1]	<0.001	
Southern Africa	10.7 [2.9-22.4]	[0-65.9]	5	594	3.9 [2.8-5.3]	93.4 [87.5-96.5]	<0.001	
Northern Europe	9.1 [6-12.7]	[0.1-27.8]	17	3158	3.2 [2.6-3.8]	90 [85.6-93.1]	<0.001	
Central America	8.8 [5.9-12.2]	[0-45.5]	3	384	1.1 [1-3.4]	16.2 [0-91.3]	0.303	
Oceania	4.6 [1.6-8.8]	[0-24.7]	7	23863	7.2 [6-8.6]	98.1 [97.2-98.6]	<0.001	
Eastern Africa	2.7 [1.3-4.3]	[0.3-6.7]	4	573	1 [1-2.6]	0 [0-84.7]	0.503	
**Country income level**								0.477
High-income economies	24.4 [22.3-26.5]	[0.3-66.8]	340	265633	11.9 [11.7-12.1]	99.3 [99.3-99.3]	<0.001	
Low-income economies	24.6 [14.1-36.8]	[0-79]	15	2344	6.4 [5.6-7.2]	97.5 [96.8-98.1]	<0.001	
Lower-middle income economies	26.8 [22.7-31.1]	[0-75.1]	115	41440	9.1 [8.8-9.5]	98.8 [98.7-98.9]	<0.001	
Upper-middle-income economies	22.4 [19-26]	[0-77.2]	192	81225	11.9 [11.6-12.1]	99.3 [99.3-99.3]	<0.001	
**Type of dialysis**								0.001
Hemodialysis	25.5 [23.8-27.3]	[0-72.5]	616	368136	11.8 [11.7-12]	99.3 [99.3-99.3]	<0.001	
Peritoneal dialysis	10.4 [4.4-18.3]	[0-73.3]	37	5137	7.7 [7.2-8.3]	98.3 [98.1-98.5]	<0.001	
**HCV_diagnostic_method**								0.003
Indirect ELISA	25.2 [23.1-27.3]	[0-73.2]	445	217926	11.1 [10.9-11.3]	99.2 [99.2-99.2]	<0.001	
Enzyme immunoassay (EIA)	24.6 [16.2-34.1]	[1.8-61.2]	8	4539	5.7 [4.7-7]	97 [95.6-97.9]	<0.001	
Classical RT-PCR	24.2 [20-28.7]	[1.5-60.6]	56	8986	4.7 [4.4-5.1]	95.5 [94.7-96.2]	<0.001	
Immunochromatographic test	22.9 [7.3-43.4]	[0-95.1]	5	375	4 [2.9-5.5]	93.8 [88.5-96.7]	<0.001	
Real-time RT-PCR	18.6 [10.8-27.8]	[0-58.8]	11	1931	4.8 [4-5.8]	95.7 [93.9-97]	<0.001	
Immunoblot Assay	18.2 [6.1-34.6]	[0-84.6]	5	466	4 [2.9-5.5]	93.8 [88.3-96.7]	<0.001	
Rapid Diagnostic test	18.2 [7.4-32.5]	[0-75]	8	1434	6.1 [5-7.3]	97.3 [96.1-98.1]	<0.001	
Microparticle enzyme immunoassay	17.1 [8.4-27.9]	[0-71]	16	7701	10.6 [9.7-11.6]	99.1 [98.9-99.3]	<0.001	

	Prevalence. % (95%CI)	95% Prediction interval	N Studies	N Participants	H (95%CI)	I² (95%CI)	P heterogeneity	P difference subtypes
Chemiluminescent immunoassay	15.4 [11.3-20]	[0.5-43.9]	26	14682	6.9 [6.3-7.6]	97.9 [97.5-98.3]	<0.001	
Direct ELISA	9.4 [2.9-18.8]	[0-100]	3	503	2.9 [1.7-4.9]	88.2 [67.2-95.8]	<0.001	
**Target**								0.649
Anti-HCV	24.5 [22.8-26.2]	[0-69.7]	589	306314	10.8 [10.6-10.9]	99.1 [99.1-99.2]	<0.001	
Viral antigen	16.7 [3.4-37]	[0-94.6]	5	725	6.2 [4.8-7.8]	97.4 [95.7-98.4]	<0.001	
Viral RNA	23 [17.9-28.7]	[0-74.7]	71	84394	9.4 [9-9.8]	98.9 [98.8-99]	<0.001	
