# Supplementary material for: Global epidemiology of hepatitis C virus in dialysis patients: A systematic review and meta-analysis
Source: PLoS One. 2024 Feb 8;19(2):e0284169. doi: 10.1371/journal.pone.0284169 (PMC10852299; doi:10.1371/journal.pone.0284169)
Supplement: S1 Text — (PDF) [file pone.0284169.s010.pdf]

S1 text: List of included studies

1. Abacioglu, Y.H., et al., Molecular evidence of nosocomial transmission of hepatitis C virus in a haemodialysis unit. Eur J Clin Microbiol Infect Dis, 2000. 19(3): p. 182-6.

2. Abad, S., et al., Effectiveness of direct-acting antivirals in Hepatitis C virus infection in haemodialysis patients. Nefrologia, 2017. 37(2): p. 158-163.

3. Abb, J., Prevalence of hepatitis C virus antibodies in hospital personnel. Zentralbl Bakteriol, 1991. 274(4): p. 543-7.

4. Abboud, O., A. Rashid, and S. Al-Kaabi, Hepatitis C virus infection in hemodialysis patients in qatar. Saudi J Kidney Dis Transpl, 1995. 6(2): p. 151-3.

5. Abd Alrahman, S. and A. Gassoum, Prevalence of hepatitis c virus in haemodialysis patients, in Sudan. International Journal of Current Research, 2015. 7: p. 6.

6. Abdelaali, B., et al., Hepatitis C Viral Prevalence and Seroconversion in Moroccan Hemodialysis Units: Eight Year Follow Up. Journal of Medical Diagnostic Methods, 2013. 02(05).

7. Abdelnour, G.E., et al., Detection of anti-hepatitis C-virus antibodies and hepatitis C-virus RNA in Lebanese hemodialysis patients. Eur J Epidemiol, 1997. 13(8): p. 863-7.

8. Abdourakhmanov, D.T., et al., Epidemiological and clinical aspects of hepatitis C virus infection in the Russian Republic of Daghestan. Eur J Epidemiol, 1998. 14(6): p. 549-53.

9. Abdulkarim, A.S., et al., Hepatitis C virus genotypes and hepatitis G virus in hemodialysis patients from Syria: identification of two novel hepatitis C virus subtypes. Am J Trop Med Hyg, 1998. 59(4): p. 571-6.

10. Abe, T., S. Oomori, and W. Obara, Current Status of Hepatitis C Virus-Infected Maintenance Hemodialysis Patients in Japan. Ther Apher Dial, 2018. 22(1): p. 58-60.

11. Abou Rached, A., et al., Incidence and prevalence of hepatitis B and hepatitis C viruses in hemodialysis patients in Lebanon. World J Nephrol, 2016. 5(1): p. 101-7.

12. AbouSeif, K., et al., Association of conjunctival and corneal calcification with vascular calcification among hepatitis-C-seropositive hemodialysis patients. Saudi J Kidney Dis Transpl, 2016. 27(6): p. 1168-1181.

13. Abu-Aisha, H., et al., The effect of chemical and heat disinfection of the hemodialysis machines on the spread of hepatitis C virus infection: a prospective study. Saudi J Kidney Dis Transpl, 1995. 6(2): p. 174-8.

14. Açıkgöz, G., et al., Correlation of hepatitis C antibody levels in gingival crevicular fluid and saliva of hepatitis C seropositive hemodialysis patients. Int J Dent, 2009. 2009: p. 247121.

15. Afsar, B., et al., Quality of life in hemodialysis patients: hepatitis C virus infection makes sense. Int Urol Nephrol, 2009. 41(4): p. 1011-9.

16. Agarwal, S.K., et al., Hepatitis C virus infection in haemodialysis: the 'no-isolation' policy should not be generalized. Nephron Clin Pract, 2009. 111(2): p. c133-40.

17. Agarwal, S.K., S.C. Dash, and M. Irshad, Hepatitis C virus infection during haemodialysis in India. J Assoc Physicians India, 1999. 47(12): p. 1139-43.

18. Agarwal, S.K., M. Irshad, and S.C. Dash, HCV infection during renal replacement therapy: should we dialyze all HCV-positive patients on dedicated machines? Nephron, 1998. 79(4): p. 479-80.

19. Ahmad, M.S., et al., Prevalence of antibodies against the hepatitis C virus among voluntary blood donors at a makkah hospital. Saudi J Kidney Dis Transpl, 1995. 6(2): p. 122-4.

20. Ahmadi, S.M. and N. Raeessi, Assessing the prevalence of HBV and HCV infections in children under going hemodialysis and the related risk factors in a children's Medical Center: PS132. Porto Biomed J, 2017. 2(5): p. 236.

21. Ahmed, H., H. Rahman, and R. Harun Ur, Prevalence of hepatitis C virus in maintenance haemodialysis patients - A prospective study. Bangladesh Renal Journal, 2003. 22: p. 39-43.

22. Ahmetagić, S., et al., [Hepatitis C virus infection in hemodialysis patients in General Hospital Gracanica]. Med Arh, 2006. 60(5): p. 298-300.

23. Ahmetagić, S., et al., Hepatitis C infection in risk groups. Bosn J Basic Med Sci, 2006. 6(4): p. 13-7.

24. Akhmouch, I., et al., Seroprevalence and risk factors for hepatitis C. Hemodialysis International, 2009. 13(3): p. 408.

25. Akpolat, T., et al., Prevalence of anti-HCV among haemodialysis patients in Turkey: a multicentre study. Nephrol Dial Transplant, 1995. 10(4): p. 479-80.

26. al Meshari, K., et al., Hepatitis C virus infection in hemodialysis patients: comparison of two new hepatitis C antibody assays with a second-generation assay. J Am Soc Nephrol, 1995. 6(5): p. 1439-44.

27. al Nasser, M.N., et al., Seropositivity to hepatitis C virus in Saudi haemodialysis patients. Vox Sang, 1992. 62(2): p. 94-7.

28. Al Shohaib, S.S., et al., The prevalence of hepatitis C virus antibodies among hemodialysis patients in jeddah area, saudi arabia. Saudi J Kidney Dis Transpl, 1995. 6(2): p. 128-31.

29. Al Traif, I., et al., Anti-HCV positive hemodialysis patients: clinical, biochemical, histologic and virologic study and a proposed management scheme. Ann Saudi Med, 2000. 20(3-4): p. 307-9.

30. Al Zabadi, H., H. Rahal, and R. Fuqaha, Hepatitis B and C prevalence among hemodialysis patients in the West Bank hospitals, Palestine. BMC Infect Dis, 2016. 16: p. 41.

31. Alashek, W.A., C.W. McIntyre, and M.W. Taal, Hepatitis B and C infection in haemodialysis patients in Libya: prevalence, incidence and risk factors. BMC Infect Dis, 2012. 12: p. 265.

32. Alavian, S.M., et al., Prevalence of hepatitis C virus infection and related risk factors among Iranian haemodialysis patients. Nephrology (Carlton), 2003. 8(5): p. 256-60.

33. Albalate, M., et al., [Spanish extrahospitalary hemodialysis centers survey]. Nefrologia, 2007. 27(2): p. 175-83.

34. Albuquerque, A.C., et al., Prevalence and risk factors of hepatitis C virus infection in hemodialysis patients from one center in Recife, Brazil. Mem Inst Oswaldo Cruz, 2005. 100(5): p. 467-70.

35. al-Dhahry, S.H., et al., Prevalence of antibodies to hepatitis C virus among Omani patients with renal disease. Infection, 1993. 21(3): p. 164-7.

36. Alfurayh, O., et al., Hepatitis C virus infection in chronic haemodialysis patients, a clinicopathologic study. Nephrol Dial Transplant, 1992. 7(4): p. 327-32.

37. Al-Ghamdi, S.M. and A.S. Al-Harbi, Hepatitis C Virus Sero-status in Hemodialysis Patients Returning from Holiday: Another Risk Factor for HCV Transmission. Saudi J Kidney Dis Transpl, 2001. 12(1): p. 14-20.

38. Ali, M., H. Moftah, and S. Moftah, Hepatitis B Virus and Hepatitis C Virus in Hemodialysis Patients: A Prevalence Study from Dialysis Centers in El-Beyda and Almarj- Libya. Asian Journal of Biology, 2017. 2(2): p. 1-5.

39. Ali, N., et al., Prevalence and risk factors of hepatitis B and C viruses among haemodialysis patients: a multicentric study. Eur J Gastroenterol Hepatol, 2019. 31(1): p. 29-33.

40. Al-Jamal, M., et al., Hepatitis C virus (HCV) infection in hemodialysis patients in the south of Jordan. Saudi J Kidney Dis Transpl, 2009. 20(3): p. 488-92.

41. Aljarallah, B.M., Hepatitis B and C Viral Infections among Dialysis Patients and Related Factors of Dialysis Centres in Saudi Arabia. Journal of Clinical and Diagnostic Research, 2022. 16(1): p. OC28-OC31.

42. Al-Jiffri, A.M., et al., Hepatitis C virus infection among patients on hemodialysis in jeddah: a single center experience. Saudi J Kidney Dis Transpl, 2003. 14(1): p. 84-9.

43. Almawi, W.Y., et al., Seroprevalence of hepatitis C virus and hepatitis B virus among dialysis patients in Bahrain and Saudi Arabia. Transplant Proc, 2004. 36(6): p. 1824-6.

44. Almroth, G., et al., Monitoring hepatitis C infection in a major Swedish nephrology unit and molecular resolution of a new case of nosocomial transmission. J Med Virol, 2010. 82(2): p. 249-56.

45. Almroth, G., et al., Detection and prevention of hepatitis C in dialysis patients and renal transplant recipients. A long-term follow up (1989-January 1997). J Intern Med, 2002. 251(2): p. 119-28.

46. al-Mugeiren, M., et al., Seropositivity to hepatitis C virus (HCV) in Saudi children with chronic renal failure maintained on haemodialysis. Ann Trop Paediatr, 1992. 12(2): p. 217-9.

47. al-Mugeiren, M., et al., Hepatitis C virus infection in two groups of paediatric patients: one maintained on haemodialysis and the other on continuous ambulatory peritoneal dialysis. Ann Trop Paediatr, 1996. 16(4): p. 335-9.

48. Al-Muhanna, F.A., Hepatitis C virus infection among hemodialysis patients in the eastern region of saudi arabia. Saudi J Kidney Dis Transpl, 1995. 6(2): p. 125-7.

49. Al-Muramdy, W.H.K., Prevalence rate of hepatitis c virus (HCV) and hepatitis b virus (HBV) infection in iraqi patients on hemodialysis: Cross sectional study. Medico-Legal Update, 2020. 20(3): p. 661-666.

50. Alonso, M.C., et al., Antibodies to hepatitis C virus in patients on haemodialysis. Nephron, 1991. 57(2): p. 247.

51. Alsaran, K.A., et al., Effect of hepatitis C virus on hemoglobin and hematocrit levels in saudi hemodialysis patients. Ren Fail, 2009. 31(5): p. 349-54.

52. Altawalah, H., et al., Prevalence of blood borne viruses in the dialysis unit, mubarak Al-Kabeer hospital, kuwait. Kuwait Medical Journal, 2015. 47(1): p. 30-32.

53. Altay, M., et al., Human herpesvirus 6 infection in hemodialysis and peritoneal dialysis patients. Perit Dial Int, 2011. 31(3): p. 320-4.

54. Altindiş, M., et al., [Investigation of hemodialysis patients in terms of the presence of occult hepatitis B]. Mikrobiyol Bul, 2007. 41(2): p. 227-33.

55. Alve-Castillo, F.M., et al., Hepatitis C virus in populations at risk for infection. Revista Espanola De Enfermedades Digestivas, 2007. 99(6): p. 315-319.

56. Alves, R.V., et al., Architect hepatitis c virus (HCV) core antigen test: A HCV rna screening alternative in end-stage renal disease (ESRD) and hemodialysis patients? Nephrology Dialysis Transplantation, 2018. 33: p. i450.

57. al-Wakeel, J., et al., Liver disease in dialysis patients with antibodies to hepatitis C virus. Nephrol Dial Transplant, 1996. 11(11): p. 2265-8.

58. Aman, K., et al., Prevalence and associated factors of hepatitis C virus infection among renal disease patients on maintenance hemodialysis in three health centers in Aden, Yemen: a cross sectional study. Saudi J Kidney Dis Transpl, 2015. 26(2): p. 380-5.

59. Amar, Y., et al., Hepatitis C virus infection in a Moroccan hemodialysis unit: Prevalence and risk factors [1]. Gastroenterologie Clinique et Biologique, 2005. 29(6-7): p. 746-747.

60. Ambrozaitis, A., et al., Hepatitis C in Lithuania: incidence, prevalence, risk factors and viral genotypes. Clin Diagn Virol, 1995. 4(4): p. 273-84.

61. Ambühl, P.M., U. Binswanger, and E.L. Renner, [Epidemiology of chronic hepatitis B and C among dialysis patients in Switzerland]. Schweiz Med Wochenschr, 2000. 130(10): p. 341-8.

62. Amin Elzorkany, K.M. and A. Zahran, Hepatitis C virus status in hemodialysis patients in Menoufia Government, Egypt, five years apart: Do we have any improvement? Saudi J Kidney Dis Transpl, 2017. 28(5): p. 1126-1132.

63. Amiri, Z.M., A.J. Shakib, and M. Toorchi, Seroprevalence of hepatitis C and risk factors in haemodialysis patients in Guilan, Islamic Republic of Iran. East Mediterr Health J, 2005. 11(3): p. 372-6.

64. Amjad, U., et al., Association of anti-HCV sero-prevalence with blood transfusion and practice of haemodialysis from multiple centres in patients on maintenance haemodialysis. Pak J Med Sci, 2020. 36(2): p. 286-289.

65. Amorim, R.M., et al., Hepatitis C virus genotypes in hemodialysis patients in the Federal District, Brazil. Rev Inst Med Trop Sao Paulo, 2010. 52(1): p. 57-60.

66. Ansar, M.M. and A. Kooloobandi, Prevalence of hepatitis C virus infection in thalassemia and haemodialysis patients in north Iran-Rasht. J Viral Hepat, 2002. 9(5): p. 390-2.

67. Ansari, M.K. and M. Omrani, Evaluation of Diagnostic Value of Elisa Method (EIA) and PCR in Diagnosis of Hepatitis C Virus In Hemodialysis Patients. Hepatitis Monthly, 2006. 6(1): p. 19-23.

68. Antonio Quiroga, J., et al., Recombinant γ-interferon as adjuvant to hepatitis B vaccine in hemodialysis patients. Hepatology, 1990. 12(4 I): p. 661-663.

69. Anwar, K., et al., Prevalence of Hepatitis B and Hepatitis C Infection among Patients Undergoing Dialysis. Journal of Human Virology & Retrovirology, 2016. 3(3).

70. Arababadi, M.K., G. Hassanshahi, and H. Yousefi, HBV-DNA in hemodialysis patients infected by HCV. Saudi J Kidney Dis Transpl, 2009. 20(3): p. 398-401.

71. Arenas, M.D., et al., [Nosocomial transmission of the hepatitis C virus in hemodialysis: monitors, personnel, or both?]. Nefrologia, 2001. 21(5): p. 476-84.

72. Aripkhodjayeva, F., et al., Peculiarities of the Clinical Course of Chronic Viral Hepatitis C in the Background of End-Stage Chronic Renal Insufficiency. Exp Clin Transplant, 2020. 18(Suppl 1): p. 47-50.

73. Assadian, A., et al., Hemodialysis access surgery - is there an increased risk of acquiring hepatitis C virus compared to other elective vascular interventions? Vasa-Journal of Vascular Diseases, 2008. 37(1): p. 81-85.

74. Assarehzadegan, M.A., et al., Prevalence of hepatitis C and B infection and HC V genotypes among hemodialysis patients in Khuzestan province, southwest Iran. Saudi J Kidney Dis Transpl, 2009. 20(4): p. 681-4.

75. Aucella, F., et al., Effectiveness of universal precautions in limiting nosocomial transmission of hepatitis C virus in haemodialysis units. Int J Artif Organs, 1996. 19(7): p. 435-6.

76. Aucella, F., et al., Systematic monitor disinfection is effective in limiting HCV spread in hemodialysis. Blood Purif, 2000. 18(2): p. 110-4.

77. Ayed, K., et al., Hepatitis C virus infection in hemodialysis patients from Tunisia: national survey by serologic and molecular methods. Transplant Proc, 2003. 35(7): p. 2573-5.

78. Ayoola, E.A., et al., Prevalence and significance of antibodies to hepatitis C virus among Saudi haemodialysis patients. J Med Virol, 1991. 35(3): p. 155-9.

79. Baby, M., et al., [Prevalence and risk factors of hepatitis C virus infection in chronic hemodialysis patients at the University Hospital of Point G, Bamako, Mali]. Mali Med, 2011. 26(2): p. 12-5.

80. Bahakim, H., et al., Hepatitis C virus antibodies in high-risk Saudi groups. Vox Sang, 1991. 60(3): p. 162-4.

81. Baheti, R., R. Gehlot, and R. Baheti, Seroprevalence of Anti HCV Ab in Healthy Voluntary Blood Donors and in High Risk Individuals. J Indian Acad Clin Med, 2000. 1.

82. Baid-Agrawal, S., et al., Prevalence of occult hepatitis C infection in chronic hemodialysis and kidney transplant patients. J Hepatol, 2014. 60(5): p. 928-33.

83. Baldessar, M.Z., et al., Hepatitis C risk factor for patients submitted to dialysis. Braz J Infect Dis, 2007. 11(1): p. 12-5.

84. Bao, K., et al., Prevalence of HCV Infection Among Hemodialysis Patients in Lanzhou of Northwestern China. Infect Drug Resist, 2022. 15: p. 5609-5617.

85. Barbosa, J.R., et al., Performance of point of care assays for hepatitis B and C viruses in chronic kidney disease patients. Journal of Clinical Pathology, 2018. 71(10): p. 879-884.

86. Barril, G., et al., Prevalence of hepatitis C virus in dialysis patients in Spain. Nephrology Dialysis Transplantation, 1995. 10(SUPPL. 6): p. 78-80.

87. Basaras, M., et al., Detection of HGV in serum and peripheral blood mononuclear cells of maintenance haemodialysis patients. J Hosp Infect, 1999. 42(2): p. 155-9.

88. Bastiani, M.F., G.G. Baiocco, and S.C. Wagner, Prevalence of hepatitis C in patients with renal disease undergoing hemodialysis treatment. Jornal Brasileiro de Patologia e Medicina Laboratorial, 2014. 50(5).

89. Batchoun, R.G., M.A. Al-Najdawi, and S. Al-Taamary, Anti-ENA antibody profile in hepatitis C patients undergoing hemodialysis. Saudi J Kidney Dis Transpl, 2011. 22(4): p. 682-8.

90. Batieha, A., et al., Epidemiology and cost of haemodialysis in Jordan. Eastern Mediterranean health journal = La revue de sante de la Mediterranee orientale = al-Majallah al-sihhiyah li-sharq al-mutawassit, 2007. 13(3): p. 654-63.

91. Bdour, S., Hepatitis C virus infection in Jordanian haemodialysis units: serological diagnosis and genotyping. J Med Microbiol, 2002. 51(8): p. 700-704.

92. Beccari, M., et al., HGV infection in dialysis patients. Nephrol Dial Transplant, 1996. 11(11): p. 2370-1.

93. Ben Othman, S., et al., [High prevalence and incidence of hepatitis C virus infections among dialysis patients in the East-Centre of Tunisia]. Pathol Biol (Paris), 2004. 52(6): p. 323-7.

94. Benghanem, M., et al., Virological markers profile in hemodialysis patients. Hemodialysis International, 2009. 13(3): p. 409.

95. Bernieh, B., et al., Prevalence of hepatitis C virus antibodies in hemodialysis patients in madinah Al munawarah. Saudi J Kidney Dis Transpl, 1995. 6(2): p. 132-5.

96. Besso, L., et al., Prevalence of HCV antibodies in a uraemic population undergoing maintenance dialysis therapy and in the staff members of the dialysis unit. Nephron, 1992. 61(3): p. 304-6.

97. Bhaumik, P. and K. Debnath, Prevalence of Hepatitis B and C among HemodialysisPatients of Tripura, India. Euroasian Journal of Hepato-Gastroenterology, 2012. 2(1): p. 10-13.

98. Bin Selm, S.A., The seroprevalence of anti-HCV in high-risk dialysis patients. Saudi J Kidney Dis Transpl, 2011. 22(4): p. 806-7.

99. Bin Shabbir, U., et al., Seroprevalence of Hepatitis B Virus and Hepatitis C Virus in Patients Undergoing Maintenance Hemodialysis. Cureus Journal of Medical Science, 2022. 14(5).

100. Boero, R., et al., HCV viremia in hemodialysis patients: detection by a DNA enzyme immunoassay for amplified HCV sequences. Ren Fail, 1995. 17(5): p. 565-73.

101. Bonfm Ferreira, T.M., et al., Does infection by the hepatitis C virus decrease the response of immunization against the hepatitis B virus in individuals undergoing dialysis? J Bras Nefrol, 2017. 39(2): p. 141-145.

102. Borzecki, A., et al., Occurrence of infectious diseases in dialysed patients. Ann Univ Mariae Curie Sklodowska Med, 2004. 59(1): p. 351-5.

103. Bosevska, G., et al., Screening for hepatitis B, C and HIV infection among patients on haemodialysis (cross sectional analysis among patients from two dialysis units in the period January to July 2005). Prilozi, 2009. 30(2): p. 159-74.

104. Boulaajaj, K., et al., [Prevalence of hepatitis C, hepatitis B and HIV infection among haemodialysis patients in Ibn-Rochd university hospital, Casablanca]. Nephrol Ther, 2005. 1(5): p. 274-84.

105. Boysen, T., et al., Presence and significance of TT virus in Danish patients on maintenance hemodialysis. Scand J Urol Nephrol, 2003. 37(3): p. 259-64.

106. Bracchi, O., et al., Hepatitis C: reality of a renal unit. Nephron, 1992. 61(3): p. 369-70.

107. Broumand, B., et al., Prevalence of hepatitis C infection and its risk factors in hemodialysis patients in tehran: preliminary report from "the effect of dialysis unit isolation on the incidence of hepatitis C in dialysis patients" project. Saudi J Kidney Dis Transpl, 2002. 13(4): p. 467-72.

108. Brugnano, R., et al., Antibodies against hepatitis C virus in hemodialysis patients in the central Italian region of Umbria: evaluation of some risk factors. Nephron, 1992. 61(3): p. 263-5.

109. Budihusodo, U., et al., Seroepidemiology of HBV and HCV infection in Jakarta, Indonesia. Gastroenterol Jpn, 1991. 26 Suppl 3: p. 196-201.

110. Bukh, J., et al., High prevalence of hepatitis C virus (HCV) RNA in dialysis patients: failure of commercially available antibody tests to identify a significant number of patients with HCV infection. Copenhagen Dialysis HCV Study Group. The Journal of infectious diseases, 1993. 168(6): p. 1343-8.

111. Busek, S.U., et al., Hepatitis C and hepatitis B virus infection in different hemodialysis units in Belo Horizonte, Minas Gerais, Brazil. Mem Inst Oswaldo Cruz, 2002. 97(6): p. 775-8.

112. Butt, N., et al., Treatment Outcomes for Patients Undergoing Hemodialysis with Chronic Hepatitis C on the Sofosbuvir and Daclatasvir Regimen. Cureus, 2019. 11(9): p. e5702.

113. Calabrese, G., et al., Transmission of anti-HCV within the household of haemodialysis patients. Lancet, 1991. 338(8780): p. 1466.

114. Campo, N., et al., TT virus infection in haemodialysis patients. Nephrol Dial Transplant, 2000. 15(11): p. 1823-6.

115. Cantù, P., et al., Prevalence of antibodies against hepatitis C virus in a dialysis unit. Nephron, 1992. 61(3): p. 337-8.

116. Capşa, D., et al., HCV seroprevalence in dialysis patients, their relatives and medical staff. Rev Roum Virol, 1991. 42(3-4): p. 171-5.

117. Caramelo, C., et al., Undiagnosed hepatitis C virus infection in hemodialysis patients: value of HCV RNA and liver enzyme levels. Kidney Int, 1996. 50(6): p. 2027-31.

118. Carneiro, M.A., et al., Hepatitis C prevalence and risk factors in hemodialysis patients in Central Brazil: a survey by polymerase chain reaction and serological methods. Mem Inst Oswaldo Cruz, 2001. 96(6): p. 765-9.

119. Carneiro, M.A., et al., Molecular and epidemiological study on nosocomial transmission of HCV in hemodialysis patients in Brazil. J Med Virol, 2007. 79(9): p. 1325-33.

120. Carrera, F., et al., Persistence of antibodies to hepatitis C virus in a chronic hemodialysis population. Nephron, 1994. 68(1): p. 38-40.

121. Carrera, F., et al., Prevalence of non-A non-B hepatitis and anti-HCV antibodies in a Portuguese dialysis population. Nephrol Dial Transplant, 1992. 7(9): p. 913-6.

122. Carvalho, M., et al., High Prevalence of Hepatitis C Virus Infection in Chronic Hemodialysis Patients. Braz J Infect Dis, 1999. 3(4): p. 144-148.

123. Cassidy, M.J., et al., The prevalence of antibodies to hepatitis C virus at two haemodialysis units in South Africa. S Afr Med J, 1995. 85(10): p. 996-8.

124. Castelnovo, C., et al., Comparison of three different tests for assessment of hepatitis C virus in dialysis patients. Perit Dial Int, 1995. 15(6): p. 241-5.

125. Catalani, C., et al., Prevalence of HBV, HDV, HCV infection and alleged risk factors in the Pistoia (Italy) haemodialysis population. Italian Journal of Allergy and Clinical Immunology, 2008. 18(1): p. 22-29.

126. Cendoroglo Neto, M., et al., Environmental transmission of hepatitis B and hepatitis C viruses within the hemodialysis unit. Artif Organs, 1995. 19(3): p. 251-5.

127. Chadha, M.S., et al., Prevalence of hepatitis B and C virus infections among haemodialysis patients in Pune (western India). Vox Sang, 1993. 64(2): p. 127-8.

128. Chan, T.M., A.S. Lok, and I.K. Cheng, Hepatitis C infection among dialysis patients: a comparison between patients on maintenance haemodialysis and continuous ambulatory peritoneal dialysis. Nephrol Dial Transplant, 1991. 6(12): p. 944-7.

129. Chang, J.M., et al., Discrepancy between serological and virological analysis of viral hepatitis in hemodialysis patients. Int J Med Sci, 2014. 11(5): p. 436-41.

130. Chanpong, G.F., et al., Hepatitis C among child transfusion and adult renal dialysis patients in Indonesia. Am J Trop Med Hyg, 2002. 66(3): p. 317-20.

131. Chattopadhyay, S., et al., Prevalence of transfusion-transmitted virus infection in patients on maintenance hemodialysis from New Delhi, India. Hemodial Int, 2005. 9(4): p. 362-6.

132. Chaudhary, R. and T. Mo, Antibody to hepatitis C virus in risk groups in Canada. Can J Infect Dis, 1992. 3(1): p. 27-9.

133. Chawla, N.S., et al., Hepatitis B and C Virus infections associated with renal replacement therapy in patients with end stage renal disease in a tertiary care hospital in India - prevalence, risk factors and outcome. Indian Journal of Nephrology, 2005. 15(4): p. 205.

134. Chen, J.J., et al., Occult hepatitis B and C in hemodialysis patients in a hyper-endemic area in southern Taiwan. Hepatology International, 2012. 6(1): p. 93-94.

135. Chen, K.S., et al., Superinfection with hepatitis C virus in hemodialysis patients with hepatitis B surface antigenemia: its prevalence and clinical significance in Taiwan. Nephron, 1996. 73(2): p. 158-64.

136. Chevaliez, S., et al., Full-length sequence analysis of a new subtype of hepatitis C virus genotype 1 starting its spread in a hemodialysis unit. Journal of Hepatology, 2009. 50: p. S149.

137. Chishti, S.M.I., A.M. Khan, and F. Bashir, Serological Monitoring Of HCV Marker In Hemodialysis Patients From Tertiary Care Hospitals Of Karachi. Med. Forum, 2015. 26(3): p. 6.

138. Chiu, Y.L., et al., Association of uraemic pruritus with inflammation and hepatitis infection in haemodialysis patients. Nephrol Dial Transplant, 2008. 23(11): p. 3685-9.

139. Chizoba, O.O. and A.A. Chibuogwu, Duration of dialysis increases risk of hepatitis C virus infections among hemodialysis patients in Anambra state, Nigeria. Universa Medicina, 2018. 37(3): p. 173-180.

140. Choi, H.Y., et al., Hepatitis B and C status according to dialysis modality in a Korean Single Center. Hemodialysis International, 2009. 13(3): p. 435.

141. Chong, V.H. and H.S. Zinna, Hepatitis C virus infection and haemodialysis: experience of a district general hospital in Brunei Darussalam. Singapore Med J, 2008. 49(11): p. 916-20.

142. Chopra, G.S., et al., Hepatitis C Virus Infection in Haemodialysis Patients: "Wolf in Sheep's Clothing". Med J Armed Forces India, 2005. 61(3): p. 241-4.

143. Chuaypen, N., et al., Prevalence and genotype distribution of hepatitis C virus within hemodialysis units in Thailand: role of HCV core antigen in the assessment of viremia. BMC Infect Dis, 2022. 22(1): p. 79.

144. Cocco, M., et al., Is hepatitis G virus a real risk for haemodialysis patients? Edtna erca j, 1998. 24(3): p. 36-7.

145. Colombo, P., et al., Prevalence of hepatitis C infection in a hemodialysis unit. Nephron, 1992. 61(3): p. 326-7.

146. Conlon, P.J., et al., Lower prevalence of anti-hepatitis C antibody in dialysis and renal transplant patients in Ireland. Ir J Med Sci, 1993. 162(4): p. 145-7.

147. Constantine, N.T., et al., Successful use of two rapid HCV assays in a high prevalence Romanian population. J Clin Lab Anal, 1994. 8(5): p. 332-4.

148. Conway, M., et al., Prevalence of antibodies to hepatitis C in dialysis patients and transplant recipients with possible routes of transmission. Nephrol Dial Transplant, 1992. 7(12): p. 1226-9.

149. Corcoran, G.D., et al., Hepatitis C virus infection in haemodialysis patients: a clinical and virological study. J Infect, 1994. 28(3): p. 279-85.

150. Cordeiro, V.M., et al., Decline in hepatitis B and C prevalence among hemodialysis patients in Tocantins, Northern Brazil. Rev Inst Med Trop Sao Paulo, 2018. 60: p. e36.

151. Couroucé, A.M., et al., Hepatitis C virus (HCV) infection in haemodialysed patients: HCV-RNA and anti-HCV antibodies (third-generation assays). Nephrol Dial Transplant, 1995. 10(2): p. 234-9.

152. Covic, A., et al., Hepatitis virus infection in haemodialysis patients from Moldavia. Nephrol Dial Transplant, 1999. 14(1): p. 40-5.

153. Cristina, G., et al., A survey of hepatitis C virus infection in haemodialysis patients over a 7-year follow-up. Nephrol Dial Transplant, 1997. 12(10): p. 2208-10.

154. Da Porto, A., et al., Hepatitis C virus in dialysis units: a multicenter study. Nephron, 1992. 61(3): p. 309-10.

155. Da Silva Cardoso, M., et al., Prevalence of HCV-RNA-positive patients in a dialysis unit in Germany. Nephron, 1994. 68(4): p. 517-8.

156. da Silva, N.M., et al., Evidence of association between hepatitis C virus genotype 2b and nosocomial transmissions in hemodialysis centers from southern Brazil. Virol J, 2013. 10: p. 167.

157. Dadgaran, S.A., Prevalence and Risk Factors of Hepatitis C Virus Among Hemodialysis Patients. Journal of Guilan University of Medical Sciences, 2005. 14(55): p. 76-86.

158. Dağlar, D., et al., [Investigation of hepatitis B and hepatitis C virus infections by serological and molecular methods in hemodialysis patients]. Mikrobiyol Bul, 2014. 48(1): p. 143-50.

159. Dai, C.Y., et al., SEN virus infection among patients on maintenance hemodialysis in southern Taiwan. J Infect, 2005. 51(2): p. 110-5.

160. Dai, C.Y., et al., Epidemiology and clinical significance of chronic hepatitis-related viruses infection in hemodialysis patients from Taiwan. Nephron, 2002. 90(2): p. 148-53.

161. Dalekos, G.N., et al., Absence of HCV viraemia in anti-HCV-negative haemodialysis patients. Nephrol Dial Transplant, 1998. 13(7): p. 1804-6.

162. Darrudi, A., et al., Prevalence of hepatitis B and C virus infections and immunity among hemodialysis patients in the Mazandaran province, Northern Iran. J Family Med Prim Care, 2022. 11(5): p. 1785-1788.

163. Datta, S., N. Goel, and C. Wattal, Utility of routine real time quantitative PCR monitoring of HCV infection in haemodialysis patients. Indian J Med Microbiol, 2015. 33 Suppl: p. 106-11.

164. Dattolo, P., et al., [Natural history of HCV infection and risk of death in a cohort of patients on long-term hemodialysis]. G Ital Nefrol, 2006. 23(6): p. 585-90.

165. Davies, J., et al., Blood-borne viruses in the haemodialysis-dependent population attending Top End Northern Territory facilities 2000-2009. Nephrology (Carlton), 2012. 17(5): p. 501-7.

166. Daw, M.A., et al., Prevalence of hepatitis C virus antibodies among different populations of relative and attributable risk. Saudi Med J, 2002. 23(11): p. 1356-60.

167. de Lamballerie, X., et al., Nosocomial transmission of hepatitis C virus in haemodialysis patients. J Med Virol, 1996. 49(4): p. 296-302.

168. de Los Ríos, R., et al., [Prevalence of anti hepatitis C antibodies in patients with chronic renal failure receiving conservative therapy]. Rev Gastroenterol Peru, 2006. 26(3): p. 265-70.

169. de Medina, M., et al., Prevalence of hepatitis C and G virus infection in chronic hemodialysis patients. Am J Kidney Dis, 1998. 31(2): p. 224-6.

170. de Medina, M., et al., Quantitative detection of hepatitis C virus RNA in patients undergoing hemodialysis. Asaio j, 1997. 43(1): p. 19-22.

171. de Medina, M., et al., Improved detection of antibodies to hepatitis C virus in dialysis patients using a second-generation enzyme immunoassay. Am J Kidney Dis, 1992. 20(6): p. 589-91.

172. de Paula Farah, K., et al., Hepatitis C, HCV genotypes and hepatic siderosis in patients with chronic renal failure on haemodialysis in Brazil. Nephrol Dial Transplant, 2007. 22(7): p. 2027-31.

173. Deep, A., et al., Prevalence of viral hepatitis (Hep-B and Hep-C) infection in advanced renal failure patients: a tertiary care centre study. Journal of Clinical and Experimental Hepatology, 2022. 12(Supplement 2): p. S89-S90.

174. Delarocque-Astagneau, E., et al., Outbreak of hepatitis C virus infection in a hemodialysis unit: potential transmission by the hemodialysis machine? Infect Control Hosp Epidemiol, 2002. 23(6): p. 328-34.

175. Dentico, P., et al., Prevalence and incidence of hepatitis C virus (HCV) in hemodialysis patients: study of risk factors. Clin Nephrol, 1992. 38(1): p. 49-52.

176. Dentico, P., et al., Hepatitis C virus-RNA, immunoglobulin M anti-HCV and risk factors in haemodialysis patients. Microbios, 1999. 99(392): p. 55-62.

177. Dentico, P., et al., Hepatitis C virus serotypes in haemodialysis patients in South-East Italy. Scand J Infect Dis, 2000. 32(2): p. 143-6.

178. Dentico, P., et al., Hepatitis C virus in hemodialysis patients. Nephron, 1992. 61(3): p. 307-8.

179. Dentico, P., et al., Detection of antibodies to HCV in haemodialysis patients using two second generation ELISA tests. Ital J Gastroenterol, 1993. 25(1): p. 19-22.

180. Dentico, P., et al., HCV third generation test in hemodialysis patients. Ital J Gastroenterol, 1995. 27(6): p. 300-2.

181. Devesa, M., et al., Reduced antibody reactivity to hepatitis C virus antigens in hemodialysis patients coinfected with hepatitis B virus. Clin Diagn Lab Immunol, 1997. 4(6): p. 639-42.

182. Di Lallo, D., et al., Risk factors of hepatitis C virus infection in patients on hemodialysis: a multivariate analysis based on a dialysis register in Central Italy. Eur J Epidemiol, 1999. 15(1): p. 11-4.

183. Di Loreto, M., et al., Hepatitis C virus antibodies in dialysis pediatric patients. Nephron, 1992. 61(3): p. 365-6.

184. Di Maggio, A., et al., Confirmation of high prevalence of hepatitis C antibodies in hemodialysis patients by second generation immunoblot assay. Nephron, 1992. 61(3): p. 347-9.

185. Di Napoli, A., et al., Epidemiology of hepatitis C virus among long-term dialysis patients: a 9-year study in an Italian region. Am J Kidney Dis, 2006. 48(4): p. 629-37.

186. Dimković, N., et al., Further evidence for nosocomial spread of hepatitis C virus infection in hemodialysis units. Nephron, 1996. 74(2): p. 488.

187. Diouf, M.L., et al., [Prevalence of hepatitis B and C viruses in a chronic hemodialysis center in Dakar]. Dakar Med, 2000. 45(1): p. 1-4.

188. Djordjević, V., et al., Hepatitis C virus infection in patients on peritoneal dialysis, hemodialysis and in dialysis staff members in south Serbia. Nephron, 1996. 72(4): p. 720.

189. Djordjević, V., et al., Prevention of nosocomial transmission of hepatitis C infection in a hemodialysis unit. A prospective study. Int J Artif Organs, 2000. 23(3): p. 181-8.

190. Doblali, T., et al., [Prevalence and risk factors of hepatitis C virus infection in patients on hemodialysis: results of a Moroccan study]. Med Sante Trop, 2014. 24(4): p. 375-8.

191. Doblali, T. and R. Hadef, [Seroprevalence of hepatitis C at the Military Hospital Mohammed V of Rabat]. Pan Afr Med J, 2014. 19: p. 182.

192. DuBois, D.B., et al., Quantitation of hepatitis C viral RNA in sera of hemodialysis patients: gender-related differences in viral load. Am J Kidney Dis, 1994. 24(5): p. 795-801.

193. Dunford, L., et al., Hepatitis C virus in Vietnam: high prevalence of infection in dialysis and multi-transfused patients involving diverse and novel virus variants. PLoS One, 2012. 7(8): p. e41266.

194. Duong, C.M., D.P. Olszyna, and M.L. McLaws, Hepatitis B and C virus infections among patients with end stage renal disease in a low-resourced hemodialysis center in Vietnam: a cross-sectional study. BMC Public Health, 2015. 15: p. 192.

195. Duong, M.C. and M.L. McLaws, Screening haemodialysis patients for hepatitis C in Vietnam: The inconsistency between common hepatitis C virus serological and virological tests. J Viral Hepat, 2019. 26(1): p. 25-29.

196. Dussol, B., et al., Hepatitis C virus infection among chronic dialysis patients in the southeast of France. Provence-Alpes-Côte d'Azur Nephrologists Group. Nephrol Dial Transplant, 1995. 10(4): p. 477-8.

197. Dussol, B., et al., Detection of hepatitis C infection by polymerase chain reaction among hemodialysis patients. Am J Kidney Dis, 1993. 22(4): p. 574-80.

198. El Shahat, Y.I., et al., Hepatitis C virus infection among dialysis patients in United arab emirates. Saudi J Kidney Dis Transpl, 1995. 6(2): p. 157-62.

199. Elahi, W., et al., Hepatitis B and C Infections in Patients With Prolonged Hemodialysis Secondary to Chronic Renal Failure. Cureus, 2020. 12(10): p. e10905.

200. El-Amin, H.H., et al., Hepatitis C virus infection in hemodialysis patients in Sudan: two centers' report. Saudi J Kidney Dis Transpl, 2007. 18(1): p. 101-6.

201. El-Emshaty, W.M., et al., Diagnostic Performance of an Immunoassay for Simultaneous Detection of Hcv Core Antigen and Antibodies among Haemodialysis Patients. Braz J Microbiol, 2011. 42(1): p. 303-9.

202. Elisaf, M., et al., Antibodies against hepatitis C virus (anti-HCV) in haemodialysis patients: association with hepatitis B serological markers. Nephrol Dial Transplant, 1991. 6(7): p. 476-9.

203. El-kader, Y.E.-O.A., A.A. Elmanama, and B.M. Ayesh, Prevalence and risk factors of hepatitis B and C viruses among haemodialysis patients in Gaza strip, Palestine. Virol J, 2010. 7: p. 210.

204. El-Reshaid, K., et al., Hepatitis C virus infection in patients on maintenance dialysis in kuwait: epidemiological profile and efficacy of prophylaxis. Saudi J Kidney Dis Transpl, 1995. 6(2): p. 144-50.

205. Elzouki, A.N.Y., et al., PREVALENCE OF ANTI-HEPATITIS-C VIRUS-ANTIBODIES AND HEPATITIS-C VIRUS VIREMIA IN CHRONIC-HEMODIALYSIS PATIENTS IN LIBYA. Nephrology Dialysis Transplantation, 1995. 10(4): p. 475-476.

206. El-Zouki, A.Y., A.B. Bendard, and M.S. Sharif, HCV in hemodialysis patients in Benghazi, Libya. Ann Saudi Med, 1993. 13(2): p. 203.

207. Espinosa, M., et al., Risk of death and liver cirrhosis in anti-HCV-positive long-term haemodialysis patients. Nephrol Dial Transplant, 2001. 16(8): p. 1669-74.

208. Espinosa, M., et al., Marked reduction in the prevalence of hepatitis C virus infection in hemodialysis patients: causes and consequences. Am J Kidney Dis, 2004. 43(4): p. 685-9.

209. Espírito-Santo, M.P., et al., Genotyping hepatitis C virus from hemodialysis patients in Central Brazil by line probe assay and sequence analysis. Braz J Med Biol Res, 2007. 40(4): p. 545-50.

210. Fabrizi, F., et al., De novo HCV infection among dialysis patients: a prospective study by HCV core antigen ELISA assay. Aliment Pharmacol Ther, 2005. 21(7): p. 861-9.

211. Fabrizi, F., et al., Hepatitis C virus infection and hepatic function in chronic hemodialysis patients. Nephron, 1992. 61(1): p. 119.

212. Fabrizi, F., et al., Influence of hepatitis C virus (HCV) viraemia upon serum aminotransferase activity in chronic dialysis patients. Nephrol Dial Transplant, 1997. 12(7): p. 1394-8.

213. Fabrizi, F., et al., Hepatitis E virus infection in haemodialysis patients: a seroepidemiological survey. Nephrol Dial Transplant, 1997. 12(1): p. 133-6.

214. Fabrizi, F., et al., Incidence of seroconversion for hepatitis C virus in chronic haemodialysis patients: a prospective study. Nephrol Dial Transplant, 1994. 9(11): p. 1611-5.

215. Fabrizi, F., et al., Virological characteristics of hepatitis C virus infection in chronic hemodialysis patients: a cross-sectional study. Clin Nephrol, 1995. 44(1): p. 49-55.

216. Fabrizi, F., et al., Molecular epidemiology of hepatitis C virus infection in dialysis patients. Nephron, 1997. 77(2): p. 190-6.

217. Fabrizi, F., et al., Serologic survey for control of hepatitis C in haemodialysis patients: third-generation assays and analysis of costs. Nephrol Dial Transplant, 1997. 12(2): p. 298-303.

218. Fabrizi, F., et al., Acquisition of hepatitis C virus in hemodialysis patients: a prospective study by branched DNA signal amplification assay. Am J Kidney Dis, 1998. 31(4): p. 647-54.

219. Fabrizi, F., et al., Antibodies to hepatitis C virus (HCV) and transaminase concentration in chronic haemodialysis patients: a study with second-generation assays. Nephrol Dial Transplant, 1993. 8(8): p. 744-7.

220. Fabrizi, F., et al., Conflicting results of hepatitis C virus serological tests in haemodialysis patients. Nephron, 1993. 64(4): p. 652.

221. Fadil-Romao, M.A., L.N. Pomar, and J.E. Romao, Low risk of sexual transmission of hepatitis C virus to spouses of infected hemodialysis patients. Dialysis & Transplantation, 2006. 35(4): p. 252-+.

222. Fakunle, Y.M., et al., Prevalence of antibodies to hepatitis C virus in hemodialysis patients in Riyadh. Ann Saudi Med, 1991. 11(5): p. 504-6.

223. Falasca, E., et al., Hepatitis C virus RNA (HCV RNA) and viral types in dialysis patients in Dakar, Senegal. Clinical microbiology and infection : the official publication of the European Society of Clinical Microbiology and Infectious Diseases, 1999. 5(4): p. 230-232.

224. Faoagali, J.L. and S.J. Fleming, Hepatitis C and haemodialysis. Med J Aust, 1990. 153(5): p. 301-2.

225. Farah, K.d.P., et al., Hepatitis C, HCV genotypes and hepatic chronic renal failure on haemodialysis in plantation siderosis in patients with Brazil. Nephrology Dialysis Transplantation, 2007. 22(7): p. 2027-2031.

226. Faustini, A., et al., Hepatic and extra-hepatic sequelae, and prevalence of viral hepatitis C infection estimated from routine data in at-risk groups. BMC Infect Dis, 2010. 10: p. 97.

227. Fernando, S., et al., Antibodies to hepatitis C virus in patients with chronic renal disease in Sri Lanka. Transplant Proc, 2002. 34(8): p. 3087-90.

228. Forns, X., et al., Hepatitis G virus infection in a haemodialysis unit: prevalence and clinical implications. Nephrol Dial Transplant, 1997. 12(5): p. 956-60.

229. Forns, X., et al., Incidence and risk factors of hepatitis C virus infection in a haemodialysis unit. Nephrol Dial Transplant, 1997. 12(4): p. 736-40.

230. Forns, X., et al., High prevalence of TT virus (TTV) infection in patients on maintenance hemodialysis: frequent mixed infections with different genotypes and lack of evidence of associated liver disease. J Med Virol, 1999. 59(3): p. 313-7.

231. Forseter, G., et al., Hepatitis C in the health care setting. II. Seroprevalence among hemodialysis staff and patients in suburban New York City. Am J Infect Control, 1993. 21(1): p. 5-8.

232. Foullous, A., et al., Epidemiological and Virological Study of Hepatitis C Virus Infection in Hemodialysis (Case of Six Centers) in Morocco. Journal of Biology, Agriculture and Healthcare, 2015. ISSN 2224-3208 (Paper) ISSN 2225-093X (Online): p. 99.

233. Freitas, S.Z., et al., Prevalence, genotypes and risk factors associated with hepatitis C virus infection in hemodialysis patients in Campo Grande, MS, Brazil. Mem Inst Oswaldo Cruz, 2008. 103(4): p. 405-8.

234. Fujiyama, S., et al., The prevalence of anti-HCV antibodies in hemodialysis patients. Gastroenterol Jpn, 1991. 26 Suppl 3: p. 206-8.

235. Fujiyama, S., et al., Changes in prevalence of anti-HCV antibodies associated with preventive measures among hemodialysis patients and dialysis staff. Hepatogastroenterology, 1995. 42(2): p. 162-5.

236. Fujiyama, S., et al., Prevalence of hepatitis C virus antibodies in hemodialysis patients and dialysis staff. Hepatogastroenterology, 1992. 39(2): p. 161-5.

237. Furusyo, N., et al., Lower hepatitis G virus infection prevalence compared to hepatitis B and C virus infection prevalences. Dig Dis Sci, 2000. 45(1): p. 188-95.

238. Furusyo, N., et al., Acute hepatitis C among Japanese hemodialysis patients: a prospective 9-year study. Am J Gastroenterol, 2001. 96(5): p. 1592-600.

239. Gallego, E., et al., Effect of isolation measures on the incidence and prevalence of hepatitis C virus infection in hernodialysis. Nephron Clinical Practice, 2006. 104(1): p. C1-C6.

240. Galperim, B., et al., Hepatitis C in hemodialysis: the contribution of injection drug use. Braz J Infect Dis, 2010. 14(4): p. 422-6.

241. Garassini, M.A., et al., [Antibodies against hepatitis C virus in patients with liver diseases and in risk subjects. Preliminary report]. G e n, 1990. 44(4): p. 343-8.

242. García, F., et al., Relevance of investigating the presence of hepatitis C virus RNA in HCV antibody-negative hemodialysis patients. Am J Nephrol, 2000. 20(2): p. 166-7.

243. Garinis, G., et al., Comparison of the enzyme-linked immunosorbant assay III, recombinant immunoblot third generation assay, and polymerase chain reaction method in the detection of hepatitis C virus infection in haemodialysis patients. J Clin Lab Anal, 1999. 13(3): p. 122-5.

244. Gärtner, B.C., et al., High prevalence of hepatitis G virus (HGV) infections in dialysis staff. Nephrol Dial Transplant, 1999. 14(2): p. 406-8.

245. Gasim, G.I., et al., Epidemiology of hepatitis B and hepatitis C virus infections among hemodialysis patients in Khartoum, Sudan. J Med Virol, 2012. 84(1): p. 52-5.

246. Giammaria, U., et al., HCV infection in hemodialyzed patients: incidence and correlation with dialytic age. Nephron, 1992. 61(3): p. 335-6.

247. Gilli, P., et al., Non-A, non-B hepatitis and anti-HCV antibodies in dialysis patients. Int J Artif Organs, 1990. 13(11): p. 737-41.

248. Gladziwa, U., et al., Prevalence of antibodies to hepatitis C virus in patients on peritoneal dialysis--a multicenter study. Clin Nephrol, 1993. 40(1): p. 46-52.

249. Gohar, S.A., et al., Prevalence of antibodies to hepatitis C virus in hemodialysis patients and renal transplant recipients. J Egypt Public Health Assoc, 1995. 70(5-6): p. 465-84.

250. Gomes, M., et al., [Anti-HCV seropositivity in dialysis patients]. Rev Saude Publica, 2006. 40(5): p. 931-4.

251. González-Michaca, L., A. Mercado, and G. Gamba, [Hepatitis C viral in patients with terminal chronic kidney failure. I. Prevalence]. Rev Invest Clin, 2000. 52(3): p. 246-54.

252. Górriz, J.L., et al., Prevalence and risk factors for hepatitis C virus infection in continuous ambulatory peritoneal dialysis patients. Nephrol Dial Transplant, 1996. 11(6): p. 1109-12.

253. Gubertini, G., et al., Prevalence of hepatitis C virus antibodies in hemodialysis patients in the area of Milan. Nephron, 1992. 61(3): p. 271-2.

254. Gul, A. and F. Iqbal, Prevalence of hepatitis C in patients on maintenance haemodialysis. J Coll Physicians Surg Pak, 2003. 13(1): p. 15-8.

255. Gusbi, E., et al., Prevalence of hepatitis C infection in hemodialysis patients: Report from 37 hemodialysis centers in Libya. Libyan Journal of Medical Sciences, 2019. 3(2): p. 42.

256. Ha, S.K., et al., Hepatitis C infection in hemodialysis units. Korean J Intern Med, 1990. 5(2): p. 83-6.

257. Hachicha, J., et al., [Viral hepatitis C in chronic hemodialyzed patients in southern Tunisia. Prevalence and risk factors]. Ann Med Interne (Paris), 1995. 146(5): p. 295-8.

258. Hadiwandowo, S., et al., Hepatitis B virus subtypes and hepatitis C virus genotypes in patients with chronic liver disease or on maintenance hemodialysis in Indonesia. J Med Virol, 1994. 43(2): p. 182-6.

259. Haidar, N.A., Prevalence of hepatitis B and hepatitis C in blood donors and high risk groups in Hajjah, Yemen Republic. Saudi Med J, 2002. 23(9): p. 1090-4.

260. Hajji, M., et al., National Epidemiological Study about Hepatitis C Virus Infection among Dialysis Patients. Saudi Journal of Kidney Diseases and Transplantation, 2021. 32(6): p. 1715-1721.

261. Halfon, P., et al., Prospective virological follow-up of hepatitis C infection in a haemodialysis unit. J Viral Hepat, 1998. 5(2): p. 115-21.

262. Hallack, Hepatitis C virus transmission at an outpatient hemodialysis unit--New York, 2001-2008. MMWR Morb Mortal Wkly Rep, 2009. 58(8): p. 189-94.

263. Halle, M.P., et al., Hepatitis B, Hepatitis C, and Human Immune deficiency Virus Seroconversion Positivity Rates and Their Potential Risk Factors Among Patients on Maintenance Hemodialysis in Cameroon. Iran J Kidney Dis, 2016. 10(5): p. 304-309.

264. Hamissi, J. and H. Hamissi, Occurrence of hepatitis B and C infection among hemodialyzed patients with chronic renal failure in Qazvin, Iran: A preliminary study. Public Health, 2011. 3(1): p. 9.

265. Hammad, D.M.A., HEPATITIS B VIRUS (HBV) AND HEPATITIS C VIRUS (HCV) INFECTIONS AMONG HEMODIALYSIS PATIENTS AT OMTH. European Journal of Pharmaceutical and Medical Research, 2016: p. 4.

266. Hanuka, N., et al., Hepatitis C virus infection in renal failure patients in the absence of anti-hepatitis C virus antibodies. J Viral Hepat, 2002. 9(2): p. 141-5.

267. Hardy, N.M., et al., Antibody to hepatitis C virus increases with time on hemodialysis. Clin Nephrol, 1992. 38(1): p. 44-8.

268. Harmankaya, O., et al., Low prevalence of hepatitis C virus infection in hemodialysis units: effect of isolation? Ren Fail, 2002. 24(5): p. 639-44.

269. Hasanjani Roushan, M.R., et al., Epidemiological Aspects of Hepatitis B and C and Human Immunodeficiency Viruses Among Hemodialysis Patients in Mazandaran Province, Iran. Nephrourol Mon, 2016. 8(3): p. e37878.

270. Hassanshahi, G., et al., Post-transfusion-transmitted hepatitis C virus infection: a study on thalassemia and hemodialysis patients in southeastern Iran. Arch Virol, 2011. 156(7): p. 1111-5.

271. Hayashi, J., et al., Prevalence of antibody to hepatitis C virus in hemodialysis patients. Am J Epidemiol, 1991. 134(6): p. 651-7.

272. Hayashi, J., et al., Prevalence and role of hepatitis C viraemia in haemodialysis patients in Japan. J Infect, 1994. 28(3): p. 271-7.

273. Hayashi, J., et al., Seroepidemiology of hepatitis C virus infection in hemodialysis patients and the general population in Fukuoka and Okinawa, Japan. J Gastroenterol, 1994. 29(3): p. 276-81.

274. Hinrichsen, H., et al., Prevalence and risk factors of hepatitis C virus infection in haemodialysis patients: a multicentre study in 2796 patients. Gut, 2002. 51(3): p. 429-33.

275. Hmaied, F., et al., Hepatitis C virus infection among dialysis patients in Tunisia: incidence and molecular evidence for nosocomial transmission. J Med Virol, 2006. 78(2): p. 185-91.

276. Hmida, S., et al., [HCV antibodies in hemodialyzed patients in Tunisia]. Pathol Biol (Paris), 1995. 43(7): p. 581-3.

277. Hou, C.H., et al., Intrafamilial transmission of hepatitis C virus in hemodialysis patients. J Med Virol, 1995. 45(4): p. 381-5.

278. Hruby, Z., et al., High prevalence of antibodies to hepatitis C virus in three haemodialysis centres in south-western Poland. Nephrol Dial Transplant, 1993. 8(8): p. 740-3.

279. Hsu, B.G., et al., TT virus infection in patients on peritoneal dialysis in Taiwan. Ren Fail, 2007. 29(5): p. 553-7.

280. Huang, C.S., et al., Hepatitis C markers in hemodialysis patients. J Clin Microbiol, 1993. 31(7): p. 1764-9.

281. Hubmann, R., et al., Hepatitis C virus--does it penetrate the haemodialysis membrane? PCR analysis of haemodialysis ultrafiltrate and whole blood. Nephrol Dial Transplant, 1995. 10(4): p. 541-2.

282. Huraib, S., et al., High prevalence of and risk factors for hepatitis C in haemodialysis patients in Saudi Arabia: a need for new dialysis strategies. Nephrol Dial Transplant, 1995. 10(4): p. 470-4.

283. Hussain, Y., et al., Hepatitis-C and it's seroconversion in end stage kidney disease patients on maintenance hemodialysis and factors affecting it. Pak J Med Sci, 2019. 35(1): p. 66-70.

284. Hussein, M.M. and J.M. Mooij, Methods used to reduce the prevalence of hepatitis C in a dialysis unit. Saudi J Kidney Dis Transpl, 2010. 21(5): p. 909-13.

285. Hussein, M.M., et al., The impact of polymerase chain reaction assays for the detection of hepatitis C virus infection in a hemodialysis unit. Saudi J Kidney Dis Transpl, 2007. 18(1): p. 107-13.

286. Iancu, L., et al., [The determination of the prevalence of antibodies to the hepatitis C virus (anti-HCV) and of the serum levels of C-reactive protein in chronic dialysis patients]. Rev Med Chir Soc Med Nat Iasi, 1995. 99(1-2): p. 129-33.

287. Ibarra, H., et al., [Hepatitis C virus: results of detection in several high risk groups in the X region of Chile]. Rev Med Chil, 1995. 123(4): p. 439-44.

288. Ibrahim, M.E.-T. and M.A. Elawady, Hepatitis C Virus Seroconversion Among Hemodialysis Patients and the Role of Hepatitis C Virus Positive Patient's Isolation in Benha, Egypt. Clinical Medicine Research, 2017. 6(2): p. 31.

289. Ideura, T., et al., Clinical significance of hepatitis G virus infection in patients on long-term haemodialysis. J Gastroenterol Hepatol, 1997. 12(11): p. 762-5.

290. Ikeuchi, T., et al., Superinfection of TT virus and hepatitis C virus among chronic haemodialysis patients. J Gastroenterol Hepatol, 1999. 14(8): p. 796-800.

291. Illés, M., L. Szontágh, and G. Gál, [Hepatitis B virus markers and anti-HCV antibodies in hemodialyzed patients]. Orv Hetil, 1992. 133 Suppl 1: p. 27-30.

292. Innocenti, M., et al., Comparison of anti-hepatitis C virus detection with ELISA assay and RIBA 4 in dialysis patients: our experience. Nephron, 1992. 61(3): p. 315.

293. Ippolito, E., et al., HCV incidence in a dialysis center: preliminary reports. Nephron, 1992. 61(3): p. 375-6.

294. Irie, Y., et al., Hepatitis C infection unrelated to blood transfusion in hemodialysis patients. J Hepatol, 1994. 20(4): p. 557-9.

295. Irish, D.N., et al., Identification of hepatitis C virus seroconversion resulting from nosocomial transmission on a haemodialysis unit: implications for infection control and laboratory screening. J Med Virol, 1999. 59(2): p. 135-40.

296. Irshad, M. and S. Kumar Agarwal, HCV infection in Delhi, India. Hepatology Research, 1998. 11(2): p. 129-132.

297. Seck, S.M., et al., Trends in hepatitis C infection among hemodialysis patients in Senegal: results of a decade of prevention. Saudi J Kidney Dis Transpl, 2014. 25(6): p. 1341-5.

298. Seelig, R., et al., Hepatitis C virus infections in dialysis units: prevalence of HCV-RNA and antibodies to HCV. Ann Med, 1994. 26(1): p. 45-52.

299. Sekkat, S., et al., [Prevalence of anti-HCV antibodies and seroconversion incidence in five haemodialysis units in Morocco]. Nephrol Ther, 2008. 4(2): p. 105-10.

300. Selgas, R., et al., Prevalence of hepatitis C antibodies (HCV) in a dialysis population at one center. Perit Dial Int, 1992. 12(1): p. 28-30.

301. Selm, S.B., Prevalence of hepatitis C virus infection among hemodialysis patients in a single center in Yemen. Saudi J Kidney Dis Transpl, 2010. 21(6): p. 1165-8.

302. Seme, K., et al., Molecular evidence for nosocomial spread of two different hepatitis C virus strains in one hemodialysis unit. Nephron, 1997. 77(3): p. 273-8.

303. Seme, K., et al., High prevalence of hepatitis C virus infection in hemodialysis patients from one dialysis unit in Slovenia. Nephron, 1995. 71(1): p. 99-100.

304. Senosy, S.A. and E.M. El Shabrawy, Hepatitis C virus in patients on regular hemodialysis in Beni-Suef Governorate, Egypt. J Egypt Public Health Assoc, 2016. 91(2): p. 86-9.

305. Sezer, S., et al., Hepatitis C infection in hemodialysis patients: Protective against oxidative stress? Transplant Proc, 2006. 38(2): p. 406-10.

306. Shabbir, U.B., et al., Seroprevalence of Hepatitis B Virus and Hepatitis C Virus in Patients Undergoing Maintenance Hemodialysis. Cureus, 2022. 14(5): p. e24794.

307. Shah, N., et al., Prevalence of Hepatitis C in Dialysis Patients in Khyber Teaching Hospital Peshawar. Pakistan Journal of Medical and Health Sciences, 2022. 16(1): p. 904-906.

308. Shaheen, F.A., et al., Prevalence of hepatitis C antibodies among hemodialysis patients in the Western province of saudi arabia. Saudi J Kidney Dis Transpl, 1995. 6(2): p. 136-9.

309. Shamsdin, S.A., et al., Prevalence of HBV, HCV, and HIV Infections among Patients Undergoing Hemodialysis in Fasa, Iran: A Six-Year Follow-up Study. Middle East Journal of Digestive Diseases, 2022. 14(3): p. 317-322.

310. Shamshirian, A., et al., Evaluation of immunogenicity of hepatitis B vaccine in hemodialysis patients at Mazandaran Heart Center, Iran. Biomedical and Pharmacology Journal, 2017. 10(2): p. 557-562.

311. Shamshirsaz, A.A., et al., The role of hemodialysis machines dedication in reducing Hepatitis C transmission in the dialysis setting in Iran: a multicenter prospective interventional study. BMC Nephrol, 2004. 5: p. 13.

312. Sheng, L., et al., High prevalence of hepatitis G virus infection compared with hepatitis C virus infection in patients undergoing chronic hemodialysis. Am J Kidney Dis, 1998. 31(2): p. 218-23.

313. Sheu, J.C., et al., Prevalence of anti-HCV and HCV viremia in hemodialysis patients in Taiwan. J Med Virol, 1992. 37(2): p. 108-12.

314. Shimokura, G., et al., Patient-care practices associated with an increased prevalence of hepatitis C virus infection among chronic hemodialysis patients. Infect Control Hosp Epidemiol, 2011. 32(5): p. 415-24.

315. Shrestha, S.M., et al., Infection with GB virus C and hepatitis C virus in drug addicts, patients on maintenance hemodialysis, or with chronic liver disease in Nepal. J Med Virol, 1997. 53(2): p. 157-61.

316. Silini, E., et al., Virological features of hepatitis C virus infection in hemodialysis patients. J Clin Microbiol, 1993. 31(11): p. 2913-7.

317. Silva, L.K., et al., Prevalence of hepatitis C virus (HCV) infection and HCV genotypes of hemodialysis patients in Salvador, Northeastern Brazil. Braz J Med Biol Res, 2006. 39(5): p. 595-602.

318. Simon, N., et al., A twelve year natural history of hepatitis C virus infection in hemodialyzed patients. Kidney Int, 1994. 46(2): p. 504-11.

319. Sit, D., et al., Seroprevalence of hepatitis B and C viruses in patients with chronic kidney disease in the predialysis stage at a university hospital in Turkey. Intervirology, 2007. 50(2): p. 133-7.

320. Sivapalasingam, S., et al., High prevalence of hepatitis C infection among patients receiving hemodialysis at an urban dialysis center. Infect Control Hosp Epidemiol, 2002. 23(6): p. 319-24.

321. Sivrel Arisoy, A., et al., Prevalence of anti-HCV positivity in hemodialysis patients. Nephron, 2000. 85(4): p. 363.

322. Slizien, W., et al., [Hepatitis B (HBV) and C (HCV) virus infections as an eventual cause of chronic hepatic damage in patients undergoing maintenance hemodialysis]. Przegl Lek, 1995. 52(2): p. 47-50.

323. Soetjipto, et al., Differential prevalence of hepatitis C virus subtypes in healthy blood donors, patients on maintenance hemodialysis, and patients with hepatocellular carcinoma in Surabaya, Indonesia. J Clin Microbiol, 1996. 34(12): p. 2875-80.

324. Somi, M.H., et al., Risk factors of HCV seroconversion in hemodialysis patients in tabriz, iran. Hepat Mon, 2014. 14(6): p. e17417.

325. Somi, M.H., et al., Hepatitis C virus genotypes in patients with end-stage renal disease in East Azerbaijan, Iran. Saudi J Kidney Dis Transpl, 2008: p. 5.

326. Somsouk, M., et al., A cost-identification analysis of screening and surveillance of hepatitis C infection in a prospective cohort of dialysis patients. Dig Dis Sci, 2008. 53(4): p. 1093-9.

327. Soni, P.N., et al., Hepatitis C virus antibodies among risk groups in a South African area endemic for hepatitis B virus. Journal of medical virology, 1993. 40(1): p. 65-8.

328. Souqiyyeh, M.Z., et al., The annual incidence of seroconversion of antibodies to the hepatitis C virus in the hemodialysis population in saudi arabia. Saudi J Kidney Dis Transpl, 1995. 6(2): p. 167-73.

329. Souza, K.P., et al., Hepatitis B and C in the hemodialysis unit of Tocantins, Brazil: serological and molecular profiles. Mem Inst Oswaldo Cruz, 2003. 98(5): p. 599-603.

330. Stehman-Breen, C., et al., Risk of death among chronic dialysis patients infected with hepatitis C virus. American Journal of Kidney Diseases, 1998. 32(4): p. 629-634.

331. Su, Y., et al., Prevalence and risk factors of hepatitis C and B virus infections in hemodialysis patients and their spouses: a multicenter study in Beijing, China. J Med Virol, 2013. 85(3): p. 425-32.

332. Suliman, S.M., et al., Prevalence of hepatitis C virus infection in hemodialysis patients in Sudan. Saudi J Kidney Dis Transpl, 1995. 6(2): p. 154-6.

333. Sułowicz, W., et al., [Occurrence of antibodies against hepatitis C virus (anti-HCV) in patients on long-term hemodialysis]. Przegl Lek, 1992. 49(1-2): p. 67-8.

334. Sypsa, V., et al., Incidence and patterns of hepatitis C virus seroconversion in a cohort of hemodialysis patients. Am J Kidney Dis, 2005. 45(2): p. 334-43.

335. Szücs, M., et al., An archived serum sample as a clue for identifying the primary source of a nosocomial hepatitis C virus outbreak in a haemodialysis unit. Arch Virol, 2014. 159(9): p. 2207-12.

336. Taal, M.W. and R. van Zyl-Smit, Hepatitis C virus infection in chronic haemodialysis patients--relationship to blood transfusions and dialyser re-use. S Afr Med J, 2000. 90(6): p. 621-5.

337. Tajbakhsh, R., Prevalence of hepatitis C and B virus infections among hemodialysis patients in Karaj, Iran. Saudi J Kidney Dis Transpl, 2015. 26(4): p. 792-6.

338. Tamura, I., et al., Prevalence of four blood-borne viruses (HBV, HCV, HTLV-I, HIV-1) among haemodialysis patients in Japan. J Med Virol, 1992. 36(4): p. 271-3.

339. Tanaka, H., M. Miyano, and S. Yukawa, [Detection of TT virus (TTV) in Japanese hemodialysis (HD) patients]. Nihon Rinsho, 1999. 57(6): p. 1410-2.

340. Taremi, M., et al., Hepatitis E virus infection in hemodialysis patients: a seroepidemiological survey in Iran. BMC Infect Dis, 2005. 5: p. 36.

341. Taziki, O. and F. Espahbodi, Prevalence of hepatitis C virus infection in hemodialysis patients. Saudi J Kidney Dis Transpl, 2008. 19(3): p. 475-8.

342. Telaku, S., et al., Hepatitis B and C in dialysis units in Kosova. Virol J, 2009. 6: p. 72.

343. Teruel, J.L., et al., [Hepatitis C virus infection in patients treated with hemodialysis]. Med Clin (Barc), 1990. 95(3): p. 81-3.

344. Thanachartwet, V., et al., Viral hepatitis infections among dialysis patients: Thailand registry report. Nephrology (Carlton), 2007. 12(4): p. 399-405.

345. Thongsawat, S., et al., Occult hepatitis C virus infection during an outbreak in a hemodialysis unit in Thailand. J Med Virol, 2008. 80(5): p. 808-15.

346. Tjiang, M.M., et al., The role of dedicated reuse machine for anti HCV positive patients in reducing the incidence of hepatitis C infection in hemodialysis unit: A two year prospective study. Nephrology Dialysis Transplantation, 2016. 31: p. i547.

347. Todorov, V., et al., High prevalence of hepatitis C virus infection in one dialysis center in Bulgaria. Nephron, 1998. 79(2): p. 222-3.

348. Toosi, M.N., et al., Risk Factors and Seroprevalence of Hepatitis B and C Infections among Hemodialysis Patients in Tehran. Iranian Journal of Pathology, 2007. 2(4): p. 181-186.

349. Tsianos, E.V., et al., High frequency of antibodies to Hantaan virus and hepatitis C virus in chronic haemodialysis patients. Coincidence or cross-reaction? J Intern Med, 1993. 234(6): p. 607-10.

350. Tsuyuguchi, M., [Prevalence of hepatitis C virus infection among chronic hemodialysis patients]. Hokkaido Igaku Zasshi, 1994. 69(5): p. 1178-88.

351. Tu, A.W., et al., Prevalence and incidence of hepatitis C virus in hemodialysis patients in British Columbia: Follow-up after a possible breach in hemodialysis machines. Can J Infect Dis Med Microbiol, 2009. 20(2): p. e19-23.

352. Uçar, E., et al., [Hepatitis E virus seropositivity in hemodialysis patients in Hatay province, Turkey]. Mikrobiyol Bul, 2009. 43(2): p. 299-302.

353. Ummate, I., et al., Risk factors for hepatitis C virus sero-positivity among haemodialysis patients receiving care at kidney centre in a tertiary health facility in Maiduguri, Nigeria. Pan Afr Med J, 2014. 19: p. 305.

354. Ummate, I., et al., Prevalence of Hepatitis C Virus Infection Among Haemodialysis Patients in North-Eastern Nigeria. Tropical Journal of Nephrology, 2013: p. 5.

355. Urbanowicz, W., M. Wawrzynowicz-Syczewska, and A. Boroń-Kaczmarska, HBV and HCV infections in relation to the chosen genetic features in hemodialyzed patients. Nephron, 2000. 85(4): p. 357.

356. Urso, S., et al., Screening of relatives of anti-hepatitis-C virus positive hemodialysed patients (preliminary data). Archives of gerontology and geriatrics, 1996. 22 Suppl 1: p. 335-8.

357. Utsumi, T., et al., PREVALENCE AND RISK FACTORS OF HEPATITIS B AND C VIRUS INFECTIONS AMONG HEMODIALYSIS PATIENTS FROM PRIVATE HEMODIALYSIS UNITS IN SURABAYA, INDONESIA. Southeast Asian J Trop Med Public Health, 2016. 47(5): p. 927-34.

358. Utsunomiya, S., et al., TT virus infection in hemodialysis patients. Am J Gastroenterol, 1999. 94(12): p. 3567-70.

359. Valtuille, R., et al., The role of transfusion-transmitted virus in patients undergoing hemodialysis. J Clin Gastroenterol, 2002. 34(1): p. 86-8.

360. Vandelli, L., et al., Behavior of antibody profile against hepatitis C virus in patients on maintenance hemodialysis. Nephron, 1992. 61(3): p. 260-2.

361. Vanderborght, B.O., et al., High prevalence of hepatitis C infection among Brazilian hemodialysis patients in Rio de Janeiro: a one-year follow-up study. Rev Inst Med Trop Sao Paulo, 1995. 37(1): p. 75-9.

362. Velasquez, M.E.N., et al., Prevalence of isolated hepatitis B core antibody and occult hepatitis B among adult patients initiating hemodialysis at the Philippine General Hospital (PGH) Dialysis Unit. Journal of Gastroenterology and Hepatology, 2011. 26: p. 141.

363. Vidales-Braz, B.M., et al., Detection of hepatitis C virus in patients with terminal renal disease undergoing dialysis in southern Brazil: prevalence, risk factors, genotypes, and viral load dynamics in hemodialysis patients. Virol J, 2015. 12: p. 8.

364. Vinayakumar, A.N. and R. John, Seroprevalence of hepatitis C infection in patients undergoing haemodialysis in a tertiary care centre. Journal of Patient Safety and Infection Control, 2020. 8(2): p. 48-53.

365. Vitale, C., et al., Epidemiology of hepatitis C virus infection in dialysis units: first-versus second-generation assays. Nephron, 1993. 64(2): p. 315-6.

366. Vladutiu, D.S., et al., Infections with hepatitis B and C viruses in patients on maintenance dialysis in Romania and in former communist countries: yellow spots on a blank map? J Viral Hepat, 2000. 7(4): p. 313-9.

367. Voiculescu, M., et al., A cross-sectional epidemiological study of HBV, HCV, HDV and HEV prevalence in the SubCarpathian and South-Eastern regions of Romania. J Gastrointestin Liver Dis, 2010. 19(1): p. 43-8.

368. Wang, N.S., et al., Follow-up study of hepatitis C virus infection in uremic patients on maintenance hemodialysis for 30 months. World J Gastroenterol, 2000. 6(6): p. 888-892.

369. Wang, S.M., et al., Mortality in hepatitis C-positive patients treated with peritoneal dialysis. Perit Dial Int, 2008. 28(2): p. 183-7.

370. Wang, Y., et al., Infection with GB virus C and hepatitis C virus in hemodialysis patients and blood donors in Beijing. J Med Virol, 1997. 52(1): p. 26-30.

371. Watanabe, T., et al., GB virus C and hepatitis C virus infections in hemodialysis patients in eight Japanese centers. Nephron, 1997. 76(2): p. 171-5.

372. Weber, B., et al., Seroprevalence of HCV, HAV, HBV, HDV, HCMV and HIV in high risk groups/Frankfurt a.M., Germany. Zentralbl Bakteriol, 1995. 282(1): p. 102-12.

373. Wei, L., M. Wang, and Q. Zhang, [Detection of serum HCV RNA and HBV DNA in patients on hemodialysis]. Zhonghua Nei Ke Za Zhi, 1996. 35(11): p. 753-5.

374. Wei, Y.J., et al., Evolutionary seroepidemiology of viral hepatitis and the gap in hepatitis C care cascades among uraemic patients receiving haemodialysis in Taiwan-the Formosa-Like Group. J Viral Hepat, 2021. 28(5): p. 719-727.

375. Wu, J.S., et al., Prevalence of antibodies to hepatitis C virus (anti-HCV) in different populations in Taiwan. Zhonghua Min Guo Wei Sheng Wu Ji Mian Yi Xue Za Zhi, 1991. 24(1): p. 55-60.

376. Wu, R.H., [Detection of hepatitis C virus antigen in hemodialysis patients]. Zhonghua Shi Yan He Lin Chuang Bing Du Xue Za Zhi, 2009. 23(3): p. 232-4.

377. Yadegarynia, D., et al., Seroprevalence of hepatitis B, C and D viral among hemodialysis patients in Tehran. Iran J Microbiol, 2017. 9(3): p. 195-199.

378. Yakaryilmaz, F., et al., Prevalence of occult hepatitis B and hepatitis C virus infections in Turkish hemodialysis patients. Ren Fail, 2006. 28(8): p. 729-35.

379. Yamaji, K., et al., Long term survey of hepatitis C virus infection in hemodialysis units in Fukuoka, Japan. J Epidemiol, 1996. 6(4): p. 166-71.

380. Yap, I., et al., Seroprevalence of antibodies to the hepatitis C virus in Singapore. Southeast Asian J Trop Med Public Health, 1991. 22(4): p. 581-5.

381. Yonemura, K., et al., High prevalence of hepatitis C virus antibody in patients with chronic renal failure at the start of hemodialysis therapy. Nephron, 1996. 73(3): p. 484-5.

382. Yoshida, C.F., et al., Hepatitis C virus in chronic hemodialysis patients with non-A, non-B hepatitis. Nephron, 1992. 60(2): p. 150-3.

383. Yoshida, C.F., et al., Antibodies against non-structural c100/3 and structural core antigen of hepatitis C virus (HCV) in hemodialysis patients. Rev Inst Med Trop Sao Paulo, 1993. 35(4): p. 315-21.

384. Yousif, M.A., et al., Nosocomial Transmission of Hepatitis B Surface Antigen and Anti-Hepatitis C Virus among Hemodialysis Patients. Archives of Pharmacy Practice, 2022. 13(3): p. 5-10.

385. Yu, L., et al., Hepatitis C virus and carpal tunnel syndrome in hemodialysis patients: a single center cross-sectional study. Ren Fail, 2020. 42(1): p. 1076-1082.

386. Yu, M.L., et al., Establishment of an outreach, grouping healthcare system to achieve microelimination of HCV for uremic patients in haemodialysis centres (ERASE-C). Gut, 2021. 70(12): p. 2349-2358.

387. Yuan, J., et al., Quality control measures for lowering the seroconversion rate of hemodialysis patients with hepatitis B or C virus. Hepatobiliary Pancreat Dis Int, 2012. 11(3): p. 302-6.

388. Zahedi, M.J., et al., Seroprevalence of Hepatitis Viruses B, C, D and HIV Infection Among Hemodialysis Patients in Kerman Province, South-East Iran. Hepat Mon, 2012. 12(5): p. 339-43.

389. Zahran, A.M., Prevalence of seroconversion of hepatitis C virus among hemodialysis patients in Menoufia Governorate, Egypt. Arab J Nephrol Transplant, 2014. 7(2): p. 133-5.

390. Zamir, D., et al., [Low prevalence of hepatitis G infection in dialysis patients in Israel]. Harefuah, 1999. 137(9): p. 361-3, 432.

391. Zamir, D., et al., Hepatitis C virus seroconversion and genotype prevalence in patients and staff on chronic hemodialysis. J Clin Gastroenterol, 1999. 28(1): p. 23-8.

392. Zeldis, J.B., et al., The prevalence of hepatitis C virus antibodies among hemodialysis patients. Ann Intern Med, 1990. 112(12): p. 958-60.

393. Zeuzem, S., et al., Phylogenetic analysis of hepatitis C virus isolates from hemodialysis patients. Kidney Int, 1996. 49(3): p. 896-902.

394. Zhang, X., et al., Tumor Necrosis Factor-alpha Promoter Gene Polymorphisms Are Not Associated with Hepatitis C Virus Infection in Chinese Hemodialysis Patients. Renal Failure, 2011. 33(6): p. 593-599.

395. Zhao, X., et al., Baseline data report of the China Dialysis Outcomes and Practice Patterns Study (DOPPS). Sci Rep, 2021. 11(1): p. 873.

396. Ziaee, M., R. Azizee, and M.H. Namaei, Prevalence of HCV Infection in Hemodialysis Patients of South Khorasan in Comparison With HBV, HDV, HTLV I/II, And HIV Infection. Bangladesh Journal of Medical Science, 2013. 13(1): p. 36-39.

397. Irshad, M., et al., Torque teno virus infection in hemodialysis patients in North India. Int Urol Nephrol, 2010. 42(4): p. 1077-83.

398. Irshad, M., et al., Viral hepatitis in multiple blood transfused patients treated at a referral hospital of Delhi, India. International Medical Journal, 2002. 9: p. 57-60.

399. Isnard Bagnis, C., et al., Epidemiology update for hepatitis C virus and hepatitis B virus in end-stage renal disease in France. Liver Int, 2017. 37(6): p. 820-826.

400. Ivankovic, Z., S. Stipanic, and B. Krstonosic, Hepatitis C virus infection in hemodialysis patients: First-generation enzyme immunoassay. Croatian Medical Journal, 1994. 35(3): p. 149-153.

401. Iwasa, Y., et al., Patterns in the prevalence of hepatitis C virus infection at the start of hemodialysis in Japan. Clin Exp Nephrol, 2008. 12(1): p. 53-7.

402. Iwasaki, Y., et al., Occasional infection of hepatitis C virus occurring in haemodialysis units identified by serial monitoring of the virus infection. J Hosp Infect, 2000. 45(1): p. 54-61.

403. Izopet, J., et al., Molecular evidence for nosocomial transmission of hepatitis C virus in a French hemodialysis unit. J Med Virol, 1999. 58(2): p. 139-44.

404. Izopet, J., et al., Incidence of HCV infection in French hemodialysis units: a prospective study. J Med Virol, 2005. 77(1): p. 70-6.

405. Jaafar, R., et al., Prevalence and predisposing factors for hepatitis C virus in haemodialysis unit universiti kebangsaan malaysia medical centre. BMC Proceedings, 2011. 5.

406. Jabbari, A., S. Besharat, and B. Khodabakshi, Hepatitis C in hemodialysis centers of golestan province, northeast of Iran (2005). Hepatitis Monthly, 2008. 8(1): p. 61-65.

407. Jadoul, M., C. Cornu, and C. van Ypersele de Strihou, Incidence and risk factors for hepatitis C seroconversion in hemodialysis: a prospective study. The UCL Collaborative Group. Kidney Int, 1993. 44(6): p. 1322-6.

408. Jain, P. and S. Nijhawan, Occult hepatitis C virus infection is more common than hepatitis B infection in maintenance hemodialysis patients. World J Gastroenterol, 2008. 14(14): p. 2288-9.

409. Jaiswal, S.B., et al., Prevalence of hepatitis viruses among chronic renal failure patients on hemodialysis in central India. Dialysis & Transplantation, 2002. 31(4): p. 234-+.

410. Jakupi, X., et al., A very high prevalence of hepatitis C virus infection among patients undergoing hemodialysis in Kosovo: a nationwide study. BMC Nephrol, 2018. 19(1): p. 304.

411. Jamalidoust, M., M. Eskandari, and M. Ziyaeyan, Prevalence of Hepatitis C Infection and its Genotypes in Suspected Hemodialysis Patients, Southwest of Iran. Jundishapur Journal of Microbiology, 2021. 14(11).

412. Jankovic, N., et al., Hepatitis C and hepatitis B virus infection in hemodialysis patients and staff: a two year follow-up. Int J Artif Organs, 1994. 17(3): p. 137-40.

413. Jasuja, S., et al., Prevalence and associations of hepatitis C viremia in hemodialysis patients at a tertiary care hospital. Indian J Nephrol, 2009. 19(2): p. 62-7.

414. Jeele, M.O.O., et al., Prevalence and Risk Factors Associated with Hepatitis B and Hepatitis C Infections among Patients Undergoing Hemodialysis: A Single-Centre Study in Somalia. Int J Nephrol, 2021. 2021: p. 1555775.

415. Jeffers, L.J., et al., Hepatitis C infection in two urban hemodialysis units. Kidney Int, 1990. 38(2): p. 320-2.

416. Jemni, S., et al., Seropositivity to hepatitis C virus in Tunisian haemodialysis patients. Nouv Rev Fr Hematol, 1994. 36(5): p. 349-51.

417. Jindal, N., et al., HCV infection among healthy blood donors and risk groups in north India. Libyan J Med, 2009. 4(1): p. 17.

418. Jindal, N., et al., Hepatitis C Virus (HCV) Infection among Seronegative Patients undergoing Haemodialysis in a Remotely Located Tertiary Care Hospital of Northern India: Value of HCV-RNA and Genotypes. J Clin Diagn Res, 2015. 9(12): p. Dc10-2.

419. Jonas, M.M., et al., Hepatitis C infection in a pediatric dialysis population. Pediatrics, 1992. 89(4 Pt 2): p. 707-9.

420. Joukar, F., et al., Hepatitis C and hepatitis B seroprevalence and associated risk factors in hemodialysis patients in Guilan province, north of Iran: HCV and HBV seroprevalence in hemodialysis patients. Hepat Mon, 2011. 11(3): p. 178-81.

421. Joukar, F., et al., Occult hepatitis B infection in a hemodialysis population in Guilan province, northern Iran. Hemodial Int, 2012. 16(2): p. 294-7.

422. Juhar, S., et al., Prevalence of hepatitis B and C viruses infections among hemodialysis patients in Addis Ababa, Ethiopia. Journal of Interventional Nephrology, 2018. 1(1): p. 8-14.

423. Juszczyk, J., et al., [Anti-HCV antibodies among hemodialysed and kidney-transplanted patients]. Przegl Epidemiol, 1994. 48(1-2): p. 3-9.

424. Kalantari, H., et al., Prevalence and risk factors of hepatitis B and C viruses among hemodialysis patients in Isfahan, Iran. Adv Biomed Res, 2014. 3: p. 73.

425. Kalantar-Zadeh, K., C.J. McAllister, and L.G. Miller, Clinical characteristics and mortality in hepatitis C-positive haemodialysis patients: a population based study. Nephrol Dial Transplant, 2005. 20(8): p. 1662-9.

426. Kalita, D., S. Deka, and K. Chamuah, Circulation of an atypical hepatitis C virus (HCV) strain at a dialysis unit in northeast India. Microbiologyopen, 2021. 10(1): p. e1147.

427. Kalita, D., et al., Laboratory Evaluation of Hepatitis C Virus Infection in Patients Undergoing Hemodialysis from North East India. J Clin Exp Hepatol, 2022. 12(2): p. 475-482.

428. Kallinowski, B., et al., Significance of hepatitis B, hepatitis C and GBV-C in ANCA-positive hemodialysis patients. Nephron, 1997. 77(3): p. 357-8.

429. Kallinowski, B., et al., Prevalence of antibodies to hepatitis C virus in hemodialysis patients. Nephron, 1991. 59(2): p. 236-8.

430. Kamal, I.M.A. and B.M. Mahdi, Seroprevalence occurrence of viral hepatitis and HIV among hemodialysis patients. Ann Med Surg (Lond), 2018. 29: p. 1-4.

431. Kansay, S., J. Sekhon, and S. Rana, Seroprevalence of human immunodeficiency virus, hepatitis B virus, and hepatitis C virus among hemodialysis patients in a Tertiary Care Teaching Hospital in a developing country. Indian J Sex Transm Dis AIDS, 2019. 40(2): p. 120-125.

432. Kapoor, M., et al., Is dialysis environment more important than blood transfusion in transmission of hepatitis C virus during hemodialysis? Vox Sang, 1993. 65(4): p. 331.

433. Kara, I.H., et al., Seroprevalence and risk factors of HCV in dialysis patients in a university hemodialysis, center of southeast Anatolia, Turkey. Dialysis & Transplantation, 2001. 30(11): p. 748-+.

434. Kargar Kheirabad, A., et al., Hepatitis C and G Virus Infection Prevalence Among Hemodialysis Patients and Associated Risk Factors in the Hormozgan Province of Southern Iran. Hepat Mon, 2016. 16(10): p. e40375.

435. Kashem, A., et al., Hepatitis C Virus Among Hemodialysis Patients in Najran: Prevalence is More Among Multi-Center Visitors. Saudi J Kidney Dis Transpl, 2003. 14(2): p. 206-11.

436. Kataruka, M., et al., Incidence and Risk Factors for Hepatitis C Virus and Hepatitis B Virus Seroconversion in End-Stage Renal Failure Patients on Maintenance Hemodialysis. J Clin Exp Hepatol, 2020. 10(4): p. 316-321.

437. Kato, A., et al., Association of HCV core antigen seropositivity with long-term mortality in patients on regular hemodialysis. Nephron Extra, 2012. 2(1): p. 76-86.

438. Kessler, H.H., et al., Quantitation and genotyping of hepatitis C virus RNA in sera of hemodialysis and AIDS patients. Clin Diagn Virol, 1996. 5(1): p. 73-8.

439. Keur, I., et al., Risk factors for HCV infection in two haemodialysis units in The Netherlands. Neth J Med, 1997. 50(3): p. 97-101.

440. Khaja, M.N., et al., High prevalence of hepatitis C virus infection and genotype distribution among general population, blood donors and risk groups. Infect Genet Evol, 2006. 6(3): p. 198-204.

441. Khan, L.A. and S.A. Khan, Prevalence of hepatitis B and C markers in patients on maintenance hemodialysis in Najran. Saudi Med J, 2001. 22(7): p. 641-2.

442. Khan, S., et al., Rising burden of Hepatitis C Virus in hemodialysis patients. Virol J, 2011. 8: p. 438.

443. Khattab, O.S., Prevalence and risk factors for hepatitis C virus infection in hemodialysis patients in an Iraqi renal transplant center. Saudi J Kidney Dis Transpl, 2008. 19(1): p. 110-5.

444. Kheradpezhouh, M., et al., Presence and significance of transfusion-transmitted virus infection in Iranian patients on maintenance hemodialysis. J Microbiol Immunol Infect, 2007. 40(2): p. 106-11.

445. Khodir, S.A., et al., Prevalence of HCV infections among hemodialysis patients in Al Gharbiyah Governorate, Egypt. Arab J Nephrol Transplant, 2012. 5(3): p. 145-7.

446. Khokhar, N., et al., Risk factors for hepatitis C virus infection in patients on long-term hemodialysis. J Coll Physicians Surg Pak, 2005. 15(6): p. 326-8.

447. Kiani, I.G., et al., HCV-RNA Pcr Positivity In Hcv Antibody Negative Patients Undergoing Haemodialysis. J Ayub Med Coll Abbottabad, 2018. 30(3): p. 397-400.

448. Kikuchi, K., et al., Prevalence of hepatitis E virus infection in regular hemodialysis patients. Ther Apher Dial, 2006. 10(2): p. 193-7.

449. Kiliç, H., et al., Hepatitis C virus genotypes in chronic hemodialysis patients. Nephron, 2000. 84(4): p. 379-80.

450. Knudsen, F., et al., Hepatitis C in dialysis patients: relationship to blood transfusions, dialysis and liver disease. Kidney Int, 1993. 43(6): p. 1353-6.

451. Ko, K., et al., Eighteen-year follow-up cohort study on hepatitis B and C virus infections related long-term prognosis among hemodialysis patients in Hiroshima. J Med Virol, 2020.

452. Kobayashi, M., et al., Prospective follow-up study of hepatitis C virus infection in patients undergoing maintenance haemodialysis: comparison among haemodialysis units. J Gastroenterol Hepatol, 1998. 13(6): p. 604-9.

453. Kocabaş, E., et al., Detection of hepatitis B and C infection by polymerase chain reaction among hemodialysis patients. Nephron, 2002. 91(1): p. 178-80.

454. Koda, T., et al., [Studies on hepatitis C virus infection in haemodialysis patients]. Kansenshogaku Zasshi, 1992. 66(1): p. 66-9.

455. Kolho, E., et al., Hepatitis C antibodies in dialysis patients and patients with leukaemia. J Med Virol, 1993. 40(4): p. 318-21.

456. Kossuth-Cabrejos, S., A.M. Gavino-Gutiérrez, and W. Silva-Caso, Factors associated with the severity of pruritus in patients with terminal chronic kidney disease undergoing hemodialysis in Lima, Peru. Dermatol Reports, 2020. 12(1): p. 8310.

457. Kuhns, M., et al., Detection of hepatitis C virus RNA in hemodialysis patients. J Am Soc Nephrol, 1994. 4(7): p. 1491-7.

458. Kulkarni, M.J., et al., A cross-sectional study of dialysis practice-patterns in patients with chronic kidney disease on maintenance hemodialysis. Saudi J Kidney Dis Transpl, 2015. 26(5): p. 1050-6.

459. Kumar, D., et al., Hepatitis G virus infection in hemodialysis patients from urban Delhi. Ren Fail, 2005. 27(1): p. 87-93.

460. Kumar, H., et al., Hepatitis-C virus antibodies (anti HCV) in haemodialyzed vs non-dialyzed patients. J Pak Med Assoc, 1994. 44(2): p. 28-30.

461. Kumar, P., et al., Prevalence and risk factors of Hepatitis C among maintenance hemodialysis patients at a tertiary-care hospital in Coimbatore, India. Journal of Clinical and Diagnostic Research, 2011. 5: p. 725-728.

462. Kwon, E., et al., Differential Effect of Viral Hepatitis Infection on Mortality among Korean Maintenance Dialysis Patients: A Prospective Multicenter Cohort Study. PLoS One, 2015. 10(8): p. e0135476.

463. Lakshmi, V., A.K. Reddy, and K.V. Dakshinamurty, Evaluation of commercially available third-generation anti-hepatitis C virus enzyme-linked immunosorbent assay in patients on haemodialysis. Indian J Med Microbiol, 2007. 25(2): p. 140-2.

464. Lampe, E., et al., Nosocomal transmission of hepatitis C viruses (HCV) in a hemodialysis unit: Molecular evidence. Journal of Viral Hepatitis, 2018. 25: p. 143-144.

465. Lampe, E., et al., Molecular analysis and patterns of ALT and hepatitis C virus seroconversion in haemodialysis patients with acute hepatitis. Nephrology (Carlton), 2008. 13(3): p. 186-92.

466. Lanini, S., et al., Molecular epidemiology of a hepatitis C virus epidemic in a haemodialysis unit: outbreak investigation and infection outcome. BMC Infect Dis, 2010. 10: p. 257.

467. Lee, D.S., et al., Significance of anti-E2 in the diagnosis of HCV infection in patients on maintenance hemodialysis: anti-E2 is frequently detected among anti-HCV antibody-negative patients. J Am Soc Nephrol, 1996. 7(11): p. 2409-13.

468. Lee, G.S., et al., Hepatitis C antibodies in patients on peritoneal dialysis: prevalence and risk factors. Perit Dial Int, 1996. 16 Suppl 1: p. S424-8.

469. Lee, H.Y., et al., Comparative study of hepatitis C virus antibody between hemodialysis and continuous ambulatory peritoneal dialysis patients. Yonsei Med J, 1993. 34(4): p. 371-80.

470. Lee, S.D., et al., Seroepidemiology of hepatitis C virus infection in Taiwan. Hepatology, 1991. 13(5): p. 830-3.

471. Li Cavoli, G., et al., Hepatitis C virus core antigen in virological monitoring of dialysis patients. NDT Plus, 2010. 3: p. iii247.

472. Li, H. and S.X. Wang, Hepatitis C viral infection in a Chinese hemodialysis unit. Chin Med J (Engl), 2010. 123(24): p. 3574-7.

473. Lin, D.Y., et al., High incidence of hepatitis C virus infection in hemodialysis patients in Taiwan. Am J Kidney Dis, 1993. 21(3): p. 288-91.

474. Lin, H.H., et al., Prevalence of antibodies to hepatitis C virus in the hemodialysis unit. Am J Nephrol, 1991. 11(3): p. 192-4.

475. Lioussfi, Z., et al., Viral hepatitis C and B among dialysis patients at the Rabat University Hospital: prevalence and risk factors. Saudi J Kidney Dis Transpl, 2014. 25(3): p. 672-9.

476. Lodhi, A., et al., Profile and predictors of hepatitis and HIV infection in patients on hemodialysis of Quetta, Pakistan. Drug Discov Ther, 2019. 13(5): p. 274-279.

477. Lopes, E.P., et al., Determination of the cut-off value of serum alanine aminotransferase in patients undergoing hemodialysis, to identify biochemical activity in patients with hepatitis C viremia. J Clin Virol, 2006. 35(3): p. 298-302.

478. López-Alcorocho, J.M., et al., Prevalence of hepatitis B, hepatitis C, GB virus C/hepatitis G and TT viruses in predialysis and hemodialysis patients. J Med Virol, 2001. 63(2): p. 103-7.

479. López-Navedo, P.J., et al., Prevalence of hepatitis C virus infection at three hemodialysis units in the western region of Puerto Rico. Bol Asoc Med P R, 1999. 91(7-12): p. 100-2.

480. Lu, A., A. Wang, and R. Sheng, [Hepatitis C virus infection among long-term hemodialysis patients]. Zhonghua Nei Ke Za Zhi, 1997. 36(6): p. 402-5.

481. Luengrojanakul, P., et al., Hepatitis C virus infection in patients with chronic liver disease or chronic renal failure and blood donors in Thailand. J Med Virol, 1994. 44(3): p. 287-92.

482. Luma, H.N., et al., Seroprevalence of human immunodeficiency virus, hepatitis B and C viruses among haemodialysis patients in two newly opened centres in Cameroon. Pan Afr Med J, 2017. 27: p. 235.

483. Lwin, A.A., et al., Hepatitis B and C Viral Infections among Hemodialysis Patients Attending 500 Bedded Yangon Specialty Hospital, Myanmar. Myanmar Health Sciences Research Journal, 2018. 30(3).

484. Madhavan, A., et al., Prevalence of hepatitis C among haemodialysis patients in a tertiary care hospital in south India. Iran J Microbiol, 2020. 12(6): p. 644-649.

485. Mahmoudvand, S., et al., Seronegative occult hepatitis C infection among hemodialysis patients: A prevalence study. Ther Apher Dial, 2021. 25(2): p. 218-224.

486. Mahupe, P., et al., Prevalence and risk factors for hepatitis b and c among end-stage renal disease patients on hemodialysis in Gaborone, Botswana. Niger J Clin Pract, 2021. 24(1): p. 81-88.

487. Maia, L.P., et al., Hepatitis C virus screening and clinical monitoring of biomarkers in patients undergoing hemodialysis. J Med Virol, 2009. 81(7): p. 1220-31.

488. Makhlough, A., M. Jamshidi, and M.R. Mahdavi, Hepatitis C prevalence studied by polymerase chain reaction and serological methods in haemodialysis patients in Mazandaran, Iran. Singapore Med J, 2008. 49(11): p. 921-3.

489. Malaguti, M., et al., Antibodies to hepatitis C virus (anti-HCV): prevalence in the same geographical area in dialysis patients, staff members, and blood donors. Nephron, 1992. 61(3): p. 346.

490. Malhotra, R., et al., Hepatitis B virus and hepatitis C virus co-infection in hemodialysis patients: A retrospective study from a tertiary care hospital of North India. J Nat Sci Biol Med, 2016. 7(1): p. 72-4.

491. Mansour-Ghanael, F., et al., Prevalence of hepatitis B and C infection in hemodialysis patients of Rasht (Center of Guilan Province, Northern part of Iran). Hepatitis Monthly, 2009. 9(1): p. 45-49.

492. Marc, L., et al., HEPATITIS VIRUS INFECTION IN END-STAGE KIDNEY DISEASE PATIENTS TREATED BY HAEMODIALYSIS IN ROMANIA AFTER 5 YEARS OF ACTIVE ANTIVIRAL THERAPY, REVISITED. Nephrology Dialysis Transplantation, 2022. 37(SUPPL 3): p. i599.

493. Mas, R., et al., Antibodies against hepatitis C virus in hemodialysis patients. Turkish Journal of Gastroenterology, 1996. 7(4): p. 293-294.

494. Masoodi, I., et al., Sero Conversion of Viral Hepatitis among End Stage Renal Disease Patients on Hemodialysis in Kashmir: Results of a Prospective Study. Open Access Maced J Med Sci, 2019. 7(4): p. 587-593.

495. Masuko, K., et al., Hepatitis C virus antibodies, viral RNA and genotypes in sera from patients on maintenance haemodialysis. J Viral Hepat, 1994. 1(1): p. 65-71.

496. McIntyre, P.G., et al., Hepatitis C virus infection in renal dialysis patients in Glasgow. Nephrol Dial Transplant, 1994. 9(3): p. 291-5.

497. Medeiros, M.T., et al., [Prevalence and associated factors to hepatitis C in hemodialysis patients in Brazil]. Rev Saude Publica, 2004. 38(2): p. 187-93.

498. Mederacke, I., et al., Different kinetics of HBV and HCV during haemodialysis and absence of seronegative viral hepatitis in patients with end-stage renal disease. Nephrol Dial Transplant, 2011. 26(8): p. 2648-56.

499. Medhi, S., et al., Diagnostic utility of hepatitis C virus core antigen in hemodialysis patients (vol 41, pg 447, 2008). Clinical Biochemistry, 2008. 41(18): p. 1493-1493.

500. Medici, G., G.C. Depetri, and M. Mileti, Anti-hepatitis C virus positivity and clinical correlations in hemodialyzed patients. Nephron, 1992. 61(3): p. 363-4.

501. Medin, C., et al., Seroconversion to hepatitis C virus in dialysis patients: a retrospective and prospective study. Nephron, 1993. 65(1): p. 40-5.

502. Mello Lde, A., et al., [Hepatitis C serum prevalence in hemodialyzed patients]. Rev Soc Bras Med Trop, 2007. 40(3): p. 290-4.

503. Méndez Chacón, P., A. Vidalón, and H. Vildosola, [Risk factors for hepatitis C in hemodialysis and its impact on the waiting list for kidney transplantation]. Rev Gastroenterol Peru, 2005. 25(1): p. 12-8.

504. Méndez-Sánchez, N., et al., Prevalence of hepatitis C virus infection among hemodialysis patients at a tertiary-care hospital in Mexico City, Mexico. J Clin Microbiol, 2004. 42(9): p. 4321-2.

505. Mhalla, S., et al., Prevalence and risk factors of hepatitis B and C among hemodialysis patients in Tunisia. Med Mal Infect, 2018. 48(3): p. 175-179.

506. Mittal, G., et al., Profile of hepatitis B virus, hepatitis C virus, hepatitis d virus and human immunodeficiency virus infections in hemodialysis patients of a tertiary care hospital in uttarakhand. J Clin Exp Hepatol, 2013. 3(1): p. 24-8.

507. Mitwalli, A., et al., Hepatitis C in chronic renal failure patients. Am J Nephrol, 1992. 12(5): p. 288-91.

508. Mitwalli, A.H., et al., Hepatitis G virus (HGV) infection in Saudi dialysis patients and healthy controls. Saudi J Gastroenterol, 2000. 6(2): p. 79-83.

509. Miyano, M., H. Tanaka, and S. Ishitani, [Hepatitis C in hemodialysis patients]. Nihon Rinsho, 2001. 59(7): p. 1289-93.

510. Miyasaka, M., [Infection of hepatitis C virus in patients with chronic renal failure undergoing hemodialysis therapy and staff members]. Nihon Jinzo Gakkai Shi, 1991. 33(10): p. 989-99.

511. Mizuno, M., et al., Genetic and serological evidence for multiple instances of unrecognized transmission of hepatitis C virus in hemodialysis units. J Clin Microbiol, 1998. 36(10): p. 2926-31.

512. Mohsenzadeh, M., et al., Molecular evaluation of hepatitis G virus and hepatitis C virus in patients with chronic renal failure in Iran. African Journal of Microbiology Research, 2012. 6: p. 6257-6261.

513. Moini, M., et al., Hepatitis C virus (HCV) Infection Rate among Seronegative Hemodialysis Patients Screened by Two Methods; HCV Core Antigen and Polymerase Chain Reaction. Hepat Mon, 2013. 13(6): p. e9147.

514. Mondelli, M.U., et al., High prevalence of antibodies to hepatitis C virus in hemodialysis units using a second generation assay. Nephron, 1992. 61(3): p. 350-1.

515. Mondelli, M.U., et al., Abnormal alanine aminotransferase activity reflects exposure to hepatitis C virus in haemodialysis patients. Nephrol Dial Transplant, 1991. 6(7): p. 480-3.

516. Monsalve-Castillo, F., et al., Hepatitis C virus infection in hemodialysis patients in Maracaibo, Venezuela. Rev Inst Med Trop Sao Paulo, 2012. 54(1): p. 53-5.

517. Montalto, G., et al., Epidemiology of hepatitis C virus infection in hemodialysis patients of Sicily. Int J Artif Organs, 2008. 31(8): p. 745-6.

518. Morales, J., et al., Impact of prophylactic measures on prevalence of anti HCV and viral genotypes in a dialysis unit. Nephrol Dial Transplant, 1997. 12(12): p. 2805-7.

519. Moreira, R., et al., Prospective study of hepatitis C virus infection in hemodialysis patients by monthly analysis of HCV RNA and antibodies. Can J Microbiol, 2003. 49(8): p. 503-7.

520. Morikawa, T., et al., Prevalence and characterization of hepatitis C virus in hemodialysis patients. Intern Med, 1999. 38(8): p. 626-31.

521. Mosconi, G., et al., Epidemiology of hepatitis C in a population of hemodialysis patients. Nephron, 1992. 61(3): p. 298-9.

522. Motta, M., et al., [The prevalence of serum anti-hepatitis C virus antibodies in hemodialyzed patients]. Ann Ital Med Int, 1991. 6(4): p. 375-8.

523. Mousavi, S.S.B., et al., Epidemiology of Hepatitis C Virus Infection in ESRD Patients in Khuzestan Province, Iran. Shiraz E-Medical Journal, 2012. 13(3): p. 135-140.

524. Muhammad, A., N. Noor A, and S. M. S, Prevalence of HCV antibodies in haemodialysis population of Multan - Pakistan. 1997: p. 15-8.

525. Muller, G.Y., et al., Risk factors for dialysis-associated hepatitis C in Venezuela. Kidney Int, 1992. 41(4): p. 1055-8.

526. Murthy, K.K., et al., Profile of hepatitis B and hepatitis C virus infections in dialysis and renal transplant patients 1997-2001; CMCH vellore. Indian Journal of Nephrology, 2003. 13(1): p. 24.

527. Nakashima, F., et al., Incidence of antibodies to hepatitis C virus in patients undergoing chronic dialysis and CAPD. Kurume Med J, 1993. 40(4): p. 249-53.

528. Nakata, S., et al., Hepatitis C and B virus infections in populations at low or high risk in Ho Chi Minh and Hanoi, Vietnam. J Gastroenterol Hepatol, 1994. 9(4): p. 416-9.

529. Nakayama, E., et al., Prognosis of anti-hepatitis C virus antibody-positive patients on regular hemodialysis therapy. J Am Soc Nephrol, 2000. 11(10): p. 1896-1902.

530. Nakayama, E., et al., Low prevalence of anti-hepatitis C virus antibodies in female hemodialysis patients without blood transfusion: a multicenter analysis. J Med Virol, 1996. 48(3): p. 284-8.

531. Nasir, M.B., et al., Prevalence of hepatitis b &c in patients of end stage renal disease (ESRD) on treatment of chronic/long-term hemodialysis. Pakistan Journal of Medical and Health Sciences, 2019. 13(4): p. 1027-1029.

532. Nemati, E., et al., Hepatitis C virus infection among patients on hemodialysis: a report from a single center in Iran. Saudi J Kidney Dis Transpl, 2009. 20(1): p. 147-53.

533. Neto, M.C., et al., ENVIRONMENTAL TRANSMISSION OF HEPATITIS-B AND HEPATITIS-C VIRUSES WITHIN THE HEMODIALYSIS UNIT. Artificial Organs, 1995. 19(3): p. 251-255.

534. Neukam, K., et al., Prevalence of hepatitis C virus infection according to the year of birth: identification of risk groups. European Journal of Clinical Microbiology and Infectious Diseases, 2018. 37(2): p. 247-254.

535. Niu, M.T., et al., Outbreak of hemodialysis-associated non-A, non-B hepatitis and correlation with antibody to hepatitis C virus. Am J Kidney Dis, 1992. 19(4): p. 345-52.

536. Niu, M.T., P.J. Coleman, and M.J. Alter, Multicenter study of hepatitis C virus infection in chronic hemodialysis patients and hemodialysis center staff members. Am J Kidney Dis, 1993. 22(4): p. 568-73.

537. Nomiyama, K., et al., Prevalence of hepatitis C virus antibody in patients on chronic hemodialysis. Fukuoka Igaku Zasshi, 1998. 89(8): p. 232-7.

538. Nordenfelt, E., et al., Hepatitis C virus infection in hemodialysis patients in southern Sweden: epidemiological, clinical, and diagnostic aspects. J Med Virol, 1993. 40(4): p. 266-70.

539. Ocak, S., et al., Seroprevalence of hepatitis C in patients with type 2 diabetes mellitus and non-diabetic on haemodialysis. Int J Clin Pract, 2006. 60(6): p. 670-4.

540. Oguchi, H., et al., Hepatitis virus infection (HBV and HCV) in eleven Japanese hemodialysis units. Clin Nephrol, 1992. 38(1): p. 36-43.

541. Oguchi, H., et al., Prevalence of anti-HCV in patients on long-term hemodialysis. Nihon Jinzo Gakkai Shi, 1990. 32(3): p. 313-7.

542. Ohsawa, M., et al., Standardized prevalence ratios for chronic hepatitis C virus infection among adult Japanese hemodialysis patients. J Epidemiol, 2010. 20(1): p. 30-9.

543. Ohsawa, M., et al., Seropositivity for anti-HCV core antigen is independently associated with increased all-cause, cardiovascular, and liver disease-related mortality in hemodialysis patients. J Epidemiol, 2011. 21(6): p. 491-9.

544. Okoye, O.C., Sero-prevalence of Hepatitis B and C infection among patients with end stage renal disease at haemodialysis initiation. African Journal of Medical and Health Sciences, 2020. 19(1): p. 1-6.

545. Okubo, T., et al., Epidemiological Survey of Patients With Hemodialysis Complicated by Hepatitis C in Japan. Ther Apher Dial, 2019. 23(1): p. 44-48.

546. Oliva, J.A., et al., Markers of hepatitis C infection among hemodialysis patients with acute and chronic infection: implications for infection control strategies in hemodialysis units. Int J Artif Organs, 1995. 18(2): p. 73-7.

547. Oliva, J.A., et al., Late seroconversion of C virus markers in hemodialysis patients. Kidney Int Suppl, 1993. 41: p. S153-6.

548. Olut, A.I., F. Ozsakarya, and M. Dilek, Seroprevalence of hepatitis C virus infection and evaluation of serum aminotransferase levels among haemodialysis patients in Izmir, Turkey. J Int Med Res, 2005. 33(6): p. 641-6.

549. Omar, M.N., M.A. Tashkandy, and A.H. El Tonsy, Liver enzymes and protein electrophoretic patterns in hemodialysis patients with antibodies against the hepatitis C virus. Saudi J Kidney Dis Transpl, 1995. 6(2): p. 163-6.

550. Ono-Nita, S.K., et al., A prospective study of the prevalence of hepatitis B and C virus co-infection among patients with chronic renal disease under hemodialysis. J Hepatol, 2004. 40(4): p. 715-6.

551. Otedo, A.E., et al., Seroprevalence of hepatitis B and C in maintenance dialysis in a public hospital in a developing country. S Afr Med J, 2003. 93(5): p. 380-4.

552. Othman, B. and F. Monem, Prevalence of antibodies to hepatitis C virus among hemodialysis patients in Damascus, Syria. Infection, 2001. 29(5): p. 262-5.

553. Ouzan, D., et al., Revelance of hepatitis C virus RNA detection, quantitation and genotypes in a hemodialysis unit. European Journal of Internal Medicine, 1997. 8(3): p. 177-181.

554. Padrone, M., et al., [Anti HCV in hemodialyzed patients: reduction of the prevalence and association with epidemiological variables]. Acta Gastroenterol Latinoam, 1999. 29(3): p. 91-4.

555. Palanisamy, S., et al., Prevalence of HCV, HBV and HIV infections in patients and staff of haemodialysis unit. BMC Infectious Diseases, 2012. 12.

556. Pauri, P., et al., Risk factors and clinical expression of HCV infection in hemodialysis patients. Nephron, 1992. 61(3): p. 313-4.

557. Pawlak, K., M. Mysliwiec, and D. Pawlak, Hepatitis C virus seropositivity and TNF superfamily receptors: sCD40, sFas--the new putative determinants of endothelial dysfunction in haemodialysis patients. Thromb Res, 2010. 126(5): p. 393-8.

558. Paydas, S., et al., The prevalence of anti-HCV positivity in patients undergoing haemodialysis or with malignant disease. Br J Clin Pract, 1994. 48(1): p. 25-6.

559. Pazdiora, P., et al., [Hepatitis C virus antibodies in persons on dialysis]. Cas Lek Cesk, 1993. 132(8): p. 243-5.

560. Peco-Antić, A., et al., [Viral hepatitis C--a problem in the treatment of children with renal insufficiency on hemodialysis]. Srp Arh Celok Lek, 1993. 121(3-7): p. 81-3.

561. Pena, M.J., et al., [Epidemiologic study of infection by hepatitis C virus in a hemodialysis unit]. Enferm Infecc Microbiol Clin, 2000. 18(10): p. 496-9.

562. Peng, Y.S., et al., Influence of hepatitis C virus infection on soluble cellular adhesion molecules in hemodialysis patients. Blood Purif, 2005. 23(2): p. 106-12.

563. Pereson, M.J., et al., Seroprevalence of hepatitis B, hepatitis C and HIV infection among patients undergoing haemodialysis in Buenos Aires, Argentina. J Med Microbiol, 2021. 70(1).

564. Pérez-Fontán, M., et al., Prevalence of antihepatitis C antibodies in patients treated with continuous ambulatory peritoneal dialysis and hemodialysis. Nephron, 1991. 58(3): p. 381-2.

565. Petrosillo, N., et al., Prevalence of infected patients and understaffing have a role in hepatitis C virus transmission in dialysis. Am J Kidney Dis, 2001. 37(5): p. 1004-10.

566. Petrosillo, N., V. Puro, and G. Ippolito, Prevalence of human immunodeficiency virus, hepatitis B virus and hepatitis C virus among dialysis patients. The Italian Multicentric Study on Nosocomial and Occupational Risk of Blood-Borne Infections in Dialysis. Nephron, 1993. 64(4): p. 636-9.

567. Petrosillo, N., et al., The risks of occupational exposure and infection by human immunodeficiency virus, hepatitis B virus, and hepatitis C virus in the dialysis setting. Italian Multicenter Study on Nosocomial and Occupational Risk of Infections in Dialysis. Am J Infect Control, 1995. 23(5): p. 278-85.

568. Picciotto, A., et al., Anti-hepatitis C virus antibodies and hepatitis C virus viraemia in haemodialysis patients. Nephrol Dial Transplant, 1993. 8(10): p. 1115-7.

569. Pljesa, S., et al., [Hepatitis C virus infection in patients on chronic hemodialysis]. Srp Arh Celok Lek, 1996. 124 Suppl 1: p. 120-3.

570. Pluvio, M., et al., Hepatitis C virus-related acute and chronic hepatitis in hemodialysis patients. Nephron, 1992. 61(3): p. 322-3.

571. Poddar, N., et al., Seroprevalence of hepatitis-C virus in blood donors and high risk individuals. . Journal of Evolution of Medical and Dental Sciences, 2012. 1(6): p. 959-965.

572. Prakash, S., et al., Prevalence of hepatitis B & C viruses among patients on hemodialysis in Lucknow, Uttar Pradesh. Clinical Epidemiology and Global Health, 2014. 2(1): p. 19-23.

573. Prakash, S., et al., Comparison of third generation ELISA and conventional nested RT-PCR for detection of HCV among hemodialysis patients. Journal of Applied Pharmaceutical Science, 2014. 4(8): p. 018-022.

574. Psichogiou, M., et al., Hepatitis E virus (HEV) infection in haemodialysis patients. Nephrology Dialysis Transplantation, 1996. 11(6): p. 1093-1095.

575. Pujol, F.H., et al., High incidence of hepatitis C virus infection in hemodialysis patients in units with high prevalence. J Clin Microbiol, 1996. 34(7): p. 1633-6.

576. Raccosta, G., et al., Prevalence of hepatitides in our hemodialyzed population. Nephron, 1992. 61(3): p. 300-1.

577. Ragunathan, L., et al., Seroprevalence and risk factors for Hepatitis C virus among maintenance hemodialysis patients at a Tertiary Care Hospital in Puducherry, India. Biomedical and Biotechnology Research Journal, 2022. 6(1): p. 122-125.

578. Raina, D., N. Rawat, and A.K. Pandita, Prevalence of Hepatitis B and Hepatitis C in Patients undergoing hemodialysis at a teaching hospital in Uttarakhand. J Family Med Prim Care, 2022. 11(4): p. 1348-1353.

579. Rais-Jalali, G. and P. Khajehdehi, Anti-HCV seropositivity among haemodialysis patients of Iranian origin. Nephrol Dial Transplant, 1999. 14(8): p. 2055-6.

580. Ramezani, A., et al., Serological pattern of anti-HBc alone infers occult hepatitis B virus infection in high-risk individuals in Iran. The Journal of Infection in Developing Countries, 2010. 4(10): p. 658-661.

581. Rao, V., et al., Improved detection of hepatitis C virus infection by transcription-mediated amplification technology in dialysis population. Ren Fail, 2010. 32(6): p. 721-6.

582. Rashiti-Bytyci, A., N. Ramadani, and P. Rashiti, Hepatitis C in several risk groups of Kosovo. J Infect Dev Ctries, 2022. 16(1): p. 173-178.

583. Reddy, A.K., K.V. Dakshinamurty, and V. Lakshmi, Utility of HCV core antigen ELISA in the screening for hepatitis C virus infection in patients on hemodialysis. Indian J Med Microbiol, 2006. 24(1): p. 55-7.

584. Reddy, G.A., et al., Prevalence of HBV and HCV dual infection in patients on haemodialysis. Indian J Med Microbiol, 2005. 23(1): p. 41-3.

585. Reddy, S., et al., Hepatitis C infection and the risk of bacteremia in hemodialysis patients with tunneled vascular access catheters. South Med J, 2009. 102(4): p. 374-7.

586. Ribeiro, B.J., et al., Cross-Sectional Study to Determine the Prevalence of Hepatitis B and C Virus Infection in High Risk Groups in the Northeast Region of Brazil. International Journal of Environmental Research and Public Health, 2017. 14(7): p. 793.

587. Rigopoulou, E.I., et al., HCV-RNA qualitative assay based on transcription mediated amplification improves the detection of hepatitis C virus infection in patients on hemodialysis: results from five hemodialysis units in central Greece. J Clin Virol, 2005. 34(1): p. 81-5.

588. Rinonce, H.T., et al., Hepatitis B and C virus infection among hemodialysis patients in Yogyakarta, Indonesia: Prevalence and molecular evidence for nosocomial transmission. J Med Virol, 2013. 85(8): p. 1348-61.

589. Rivanera, D., et al., Detection of antibodies to hepatitis C virus in dialysis patients. Eur J Epidemiol, 1993. 9(1): p. 55-8.

590. Rivanera, D., et al., Prevalence of TT virus infection in Italian dialysis patients. Pathol Biol (Paris), 2009. 57(1): p. 97-100.

591. Rodrigues de Freitas, M.d.J., et al., Prevalence of hepatitis C virus infection and genotypes in patient with chronic kidney disease undergoing hemodialysis: HCV in Hemodialysis Patients. Journal of Medical Virology, 2013. 85(10): p. 1741-1745.

592. Roger, S.D., et al., Hepatitis C virus infection in haemodialysis patients. Aust N Z J Med, 1991. 21(1): p. 22-4.

593. Rostami, Z., et al., Health related quality of life in Iranian hemodialysis patients with viral hepatitis: changing epidemiology. Hepat Mon, 2013. 13(6): p. e9611.

594. Roşu, A.F., et al., Hemodialysis as predisposing factor for viral hepatitis B and C. Acta Microbiologica Hellenica, 2015. 60(3): p. 198.

595. Roy, P., et al., Prevalence and genotyping pattern of hepatitis C virus among patients on maintenance hemodialysis at five centers in Pune, India. Med J Armed Forces India, 2019. 75(1): p. 74-80.

596. Ruffatti, A., et al., Hepatitis C virus infection in hemodialyzed patients detected by first and second generation assays. Nephron, 1992. 61(3): p. 344-5.

597. Saab, S., et al., Serum alanine aminotransferase in hepatitis c screening of patients on hemodialysis. Am J Kidney Dis, 2001. 37(2): p. 308-15.

598. Sabry, A.A., et al., Effect of HCV infection on hematocrit and hemoglobin level in Egyptian hemodialysis patients. Int Urol Nephrol, 2009. 41(1): p. 189-93.

599. Said, R.A., et al., Hepatitis C virus infection in hemodialysis patients in jordan. Saudi J Kidney Dis Transpl, 1995. 6(2): p. 140-3.

600. Sakamoto, N., et al., Prevalence of hepatitis C virus infection among long-term hemodialysis patients: detection of hepatitis C virus RNA in plasma. J Med Virol, 1993. 39(1): p. 11-5.

601. Saketi, J.R., et al., Prevalence of hepatitis C virus infection among haemodialysis patients in West Java, Indonesia. Adv Exp Med Biol, 2003. 531: p. 201-9.

602. Salama, G., et al., Hepatitis C virus infection in French hemodialysis units: a multicenter study. J Med Virol, 2000. 61(1): p. 44-51.

603. Salehi, M., et al., Hepatitis G virus exposure in dialysis patients and blood donors in Isfahan-Iran. Int J Prev Med, 2014. 5(Suppl 3): p. S219-22.

604. Salunkhe, P.N., et al., Prevalence of antibodies to hepatitis C virus in HBsAg negative hemodialysis patients. Indian J Gastroenterol, 1992. 11(4): p. 164-5.

605. Samimi rad, K., et al., Prevalence of Hepatitis C virus antibody and related risk factors among hemodialysis patients in Markazi province (2004). Journal of Arak University of Medical Sciences, 2006. 9(1): p. 23-33.

606. Samimi-rad, K. and M. Hosseini, Hepatitis C Virus Infection and HCV Genotypes of Hemodialysis Patients. Iranian J Publ Health, 2008. 37: p. 7.

607. Samimi-Rad, K., et al., Hepatitis C virus infection among multi-transfused patients and personnel in haemodialysis units in central Islamic Republic of Iran. Eastern Mediterranean Health Journal, 2012. 18(3): p. 227-235.

608. Sampietro, M., et al., High prevalence of a rare hepatitis C virus in patients treated in the same hemodialysis unit: evidence for nosocomial transmission of HCV. Kidney Int, 1995. 47(3): p. 911-7.

609. San Miguel, G., et al., Hepatitis C virus antibodies in patients on hemodialysis. Infect Control Hosp Epidemiol, 1992. 13(5): p. 254, 256.

610. Sandhu, J., et al., Hepatitis C prevalence and risk factors in the northern Alberta dialysis population. Am J Epidemiol, 1999. 150(1): p. 58-66.

611. Santoro, D., et al., Hepatitis status and mortality in hemodialysis population. Ren Fail, 2009. 31(1): p. 6-12.

612. Santos, M.A. and F.J. Souto, Infection by the hepatitis C virus in chronic renal failure patients undergoing hemodialysis in Mato Grosso state, central Brazil: a cohort study. BMC Public Health, 2007. 7: p. 32.

613. Santos, M.G., et al., Prevalence of hepatitis B and hepatitis C in haemodialysis patients. Nephrology, 1998. 4(1-2): p. 101-104.

614. Saraswathy, M.P., et al., Hepatitis C virus and hepatitis B virus infection in patients and staff of haemodialysis unit - A report from Chennai. Journal of Pure and Applied Microbiology, 2013. 7(2): p. 1427-1430.

615. Sassi, F., et al., Hepatitis C virus antibodies in dialysis patients in Tunisia: a single center study. Saudi J Kidney Dis Transpl, 2000. 11(2): p. 218-22.

616. Sauné, K., et al., Decreased prevalence and incidence of HCV markers in haemodialysis units: a multicentric French survey. Nephrol Dial Transplant, 2011. 26(7): p. 2309-16.

617. Savey, A., et al., A large nosocomial outbreak of hepatitis C virus infections at a hemodialysis center. Infect Control Hosp Epidemiol, 2005. 26(9): p. 752-60.

618. Saxena, A.K. and B.R. Panhotra, The impact of nurse understaffing on the transmission of hepatitis C virus in a hospital-based hemodialysis unit. Med Princ Pract, 2004. 13(3): p. 129-35.

619. Saxena, A.K., et al., Prevalence of hepatitis C antibodies among hemodialysis patients in Al-hasa region of saudi arabia. Saudi J Kidney Dis Transpl, 2001. 12(4): p. 562-5.

620. Saxena, A.K., B.R. Panhotra, and D.S. Sundaram, The role the type of vascular access plays in the transmission of hepatitis C virus in a high prevalence hemodialysis unit. J Vasc Access, 2002. 3(4): p. 158-63.

621. Saxena, A.K., et al., Impact of dedicated space, dialysis equipment, and nursing staff on the transmission of hepatitis C virus in a hemodialysis unit of the middle east. Am J Infect Control, 2003. 31(1): p. 26-33.

622. Sayiner, A.A., et al., HCV infection in haemodialysis and CAPD patients. Nephrol Dial Transplant, 1999. 14(1): p. 256-7.

623. Schiller, A., et al., Hepatitis B and C virus infection in the hemodialysis population from three romanian regions. Nephron, 2015. 129(3): p. 202-8.

624. Schlipköter, U., Hepatitis C virus antibodies in haemodialysis patients. Lancet, 1990. 335(8702): p. 1409-10.

625. Schlipköter, U., et al., Prevalence of hepatitis C virus infections in dialysis patients and their contacts using a second generation enzymed-linked immunosorbent assay. Med Microbiol Immunol, 1992. 181(3): p. 173-80.

626. Schneeberger, P.M., et al., Hepatitis C virus infections in dialysis centers in The Netherlands: a national survey by serological and molecular methods. J Clin Microbiol, 1998. 36(6): p. 1711-5.

627. Schneeberger, P.M., et al., The prevalence and incidence of hepatitis C virus infections among dialysis patients in the Netherlands: a nationwide prospective study. J Infect Dis, 2000. 182(5): p. 1291-9.

628. Schneeberger, P.M., J. Vos, and W.C. van Dijk, Prevalence of antibodies to hepatitis C virus in a Dutch group of haemodialysis patients related to risk factors. J Hosp Infect, 1993. 25(4): p. 265-70.

629. Schroeter, M., et al., Prolonged time until seroconversion among hemodialysis patients: the need for HCV PCR. Intervirology, 2005. 48(4): p. 213-5.

630. Schroter, M., et al., GB virus C hepatitis G virus infection in hemodialysis patients: Determination of seroprevalence by a four-antigen recombinant immunoblot assay. Journal of Medical Virology, 1999. 57(3): p. 230-234.

631. Schröter, M., et al., High percentage of seronegative HCV infections in hemodialysis patients: the need for PCR. Intervirology, 1997. 40(4): p. 277-8.

632. Scipioni, F., et al., HCV in a group of chronic hemodialysis patients. Nephron, 1992. 61(3): p. 362.

633. Scott, D.R., et al., Adverse impact of hepatitis C virus infection on renal replacement therapy and renal transplant patients in Australia and New Zealand. Transplantation, 2010. 90(11): p. 1165-71.

634. Scotto, G., et al., HCV infections in dialysis patients. Nephron, 1992. 61(3): p. 320-1.
