# Supplementary material for: Global epidemiology of hepatitis C virus in dialysis patients: A systematic review and meta-analysis
Source: PLoS One. 2024 Feb 8;19(2):e0284169. doi: 10.1371/journal.pone.0284169 (PMC10852299; doi:10.1371/journal.pone.0284169)
Supplement: S3 Fig — (PDF) [file pone.0284169.s013.pdf]

S3 Fig: Funnel chart for publications of the hepatitis C virus global prevalence in dialysis patients.

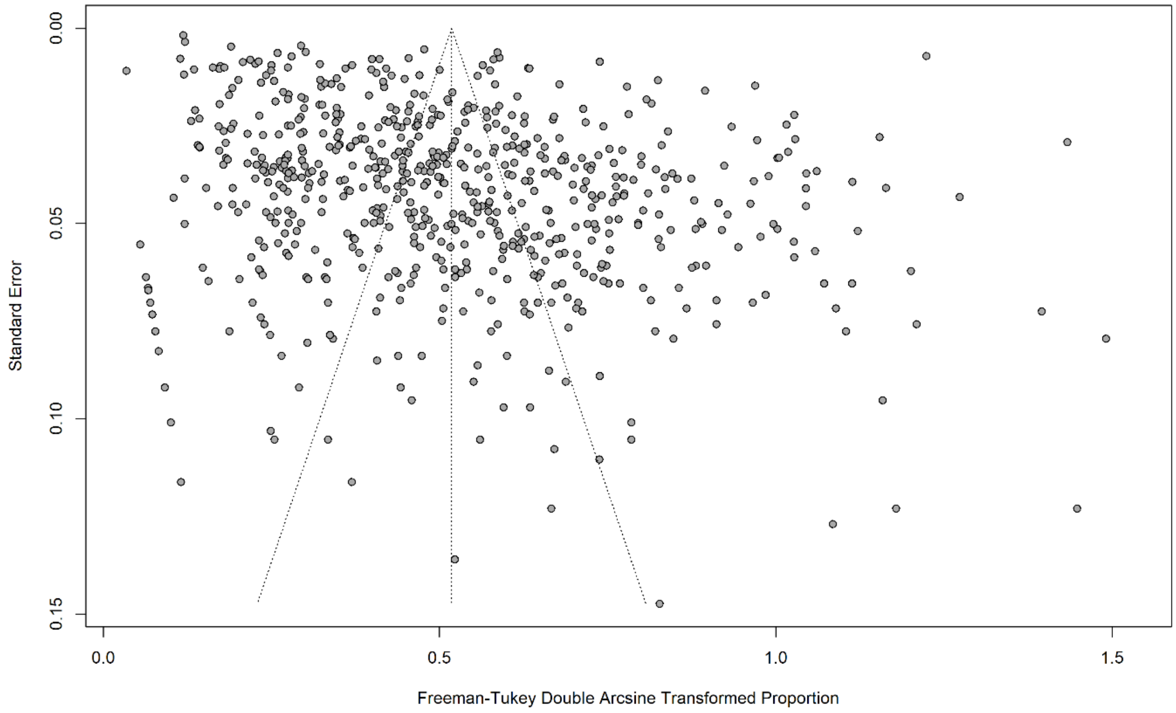

P Egger test< 0.001
